# Enantioselective Copper-Catalyzed Alkynylation of Quinolones Using Chiral P,N Ligands

**DOI:** 10.1021/acs.joc.3c01944

**Published:** 2023-11-15

**Authors:** Dáiríne M. Morgan, Cian M. Reid, Patrick J. Guiry

**Affiliations:** † Centre for Synthesis and Chemical Biology, Synthesis and Solid State Pharmaceutical Centre, School of Chemistry, University College Dublin, Belfield, Dublin 4, Ireland; ‡ Centre for Synthesis and Chemical Biology, School of Chemistry, University College Dublin, Belfield, Dublin 4, Ireland

## Abstract

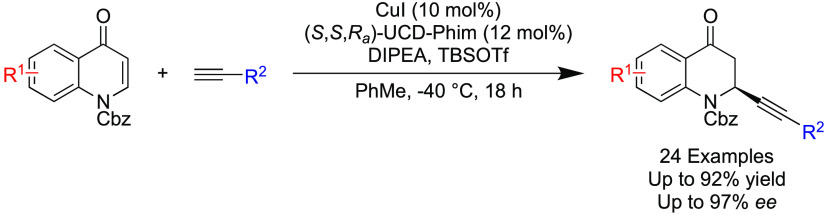

Herein we report a catalytic enantioselective alkynylation of quinolones. In this reaction, quinolones are silylated to form a quinolinium ion which then undergoes an enantioselective attack by a copper acetylide, templated by (*S*,*S*,*R*_a_)-UCD-Phim. This gives alkynylated products (24 examples) in yields of up to 92% and enantioselectivities of up to 97%. This methodology has been applied to the synthesis of two natural products, (+)-cuspareine and (+)-galipinine.

Quinolones and their derivatives are seen in the structures of many drug products and drug candidates and are well-known as broad spectrum bactericidal agents.^1−4^ Examples of quinolone-based drug products include the following (Figure 1): Ciprofloxacin, a broad-spectrum antibacterial agent used to treat various conditions caused by both Gram-positive and Gram-negative bacteria.^1^ Bradykinin B2 antagonists such as martinellic acid, which can elicit effects such as reduction of swelling, vasoconstriction, and reduced vascular permeability.^5^ The Hancock alkaloids, which are extracted from *Galipea officinalis* trunk bark, found in Venezuela, and were used in the past as part of nontraditional treatments for fever and dysentery.^6^

**Figure 1:**
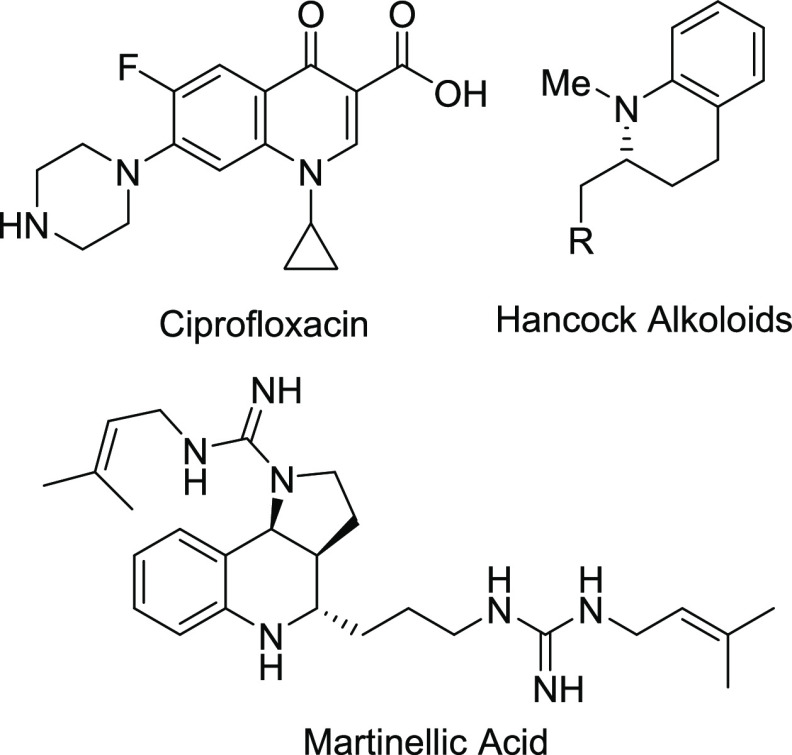
Relevant quinolone derivatives.

The Reissert reaction was originally reported in 1905 and involved the addition of potassium cyanide to quinoline in the presence of benzoyl chloride.^7^ This reaction was later adapted by Agawa and Yamaguchi to allow alkynes to be used.^8,9^ The asymmetric alkynylation of quinolones is therefore an important methodology to develop due to the potential of dihydroquinolone derivatives in medicinal chemistry.

The first enantioselective version of the reaction was carried out by Arndsten on both pyridines and quinolines using a derivative of the P,N ligand Quinap (Scheme 1a).^10,11^ Subsequently, Watson used a chiral copper acetylide in the enantioselective alkynylation of benzopyranyl oxocarbenium ions.^12^ Aponick later applied the axially chiral P,N ligand StackPhos (phosphino-imidazole) for quinoline alkynylation and expanded the methodology to include chromones as substrates through the use of silyl enol ethers (Scheme 1b).^13,14^ Mattson showed that quaternary stereocenters could be generated using copper complexes of bisoxazoline ligands.^15,16^ In 2022 Harutyunyan applied a similar methodology to the alkynylation of quinolones, mainly developing the racemic reaction although they were able to synthesize a small number of products in good to moderate enantioselectivities using the Ph-BPE ligand.^17^ During the preparation of this manuscript, Mattson published an additional account of the enantioselective alkynylation of quinolones using copper–bisoxazoline complexes, although long reaction times of 96 h were necessary to achieve good yields and enantioselectivities in this case (Scheme 1c).^18^

**Scheme 1:**
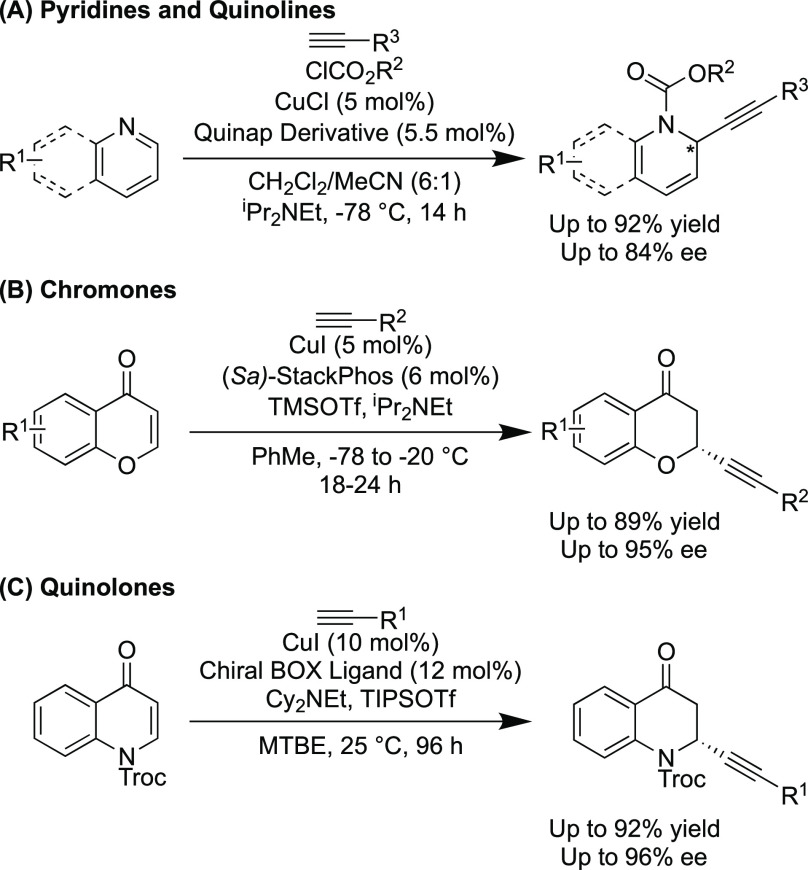
Asymmetric Alkynylation of Heterocycles

While success has been achieved with metal complexes of diphosphine and diamine ligands, the phosphinamine (P,N) class of chiral ligands, which we and others have extensively investigated, offer a range of ligands possessing central, planar, and axial chiral elements whose copper complexes warrant further investigation in this alkynylation process.^19−22^ Herein we report the results of our efforts to develop an enantioselective copper-catalyzed alkynylation of quinolones using chiral P,N ligands.

We began our optimization by testing a range of “Phim” (phosphino-imidazoline) type ligands **L1**–**L5**^23−28^ in the alkynylation reaction between Cbz-protected quinolone **1a** and phenylacetylene **2a** in the presence of CuI, DIPEA, and TBSOTf in toluene at room temperature (Table 1 and Figure 2).

**Table 1:**
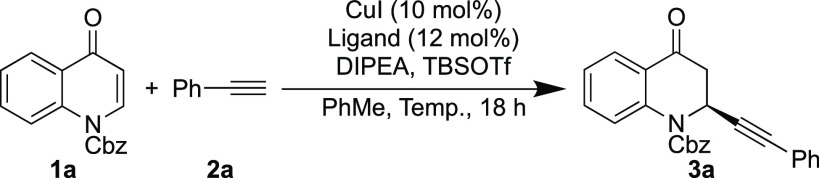
Optimization of Enantioselective Copper-Catalyzed Alkynylation^a^

entry	ligand	temp (°C)	yield (%)^b^	ee (%)^c^
1.	**L1**	20	57	4
2.	**L2**	20	49	15
3.	**L3**	20	54	0
4.	**L4**	20	65	34
5.	**L5**	20	69	17
6.	**L6**	20	30	7
7.^d^	**L4**	20	90	11
8.	**L4**	0	47	50
9.	**L4**	–20	32	54
10.	**L7**	–20	62	70
11.^e^	**L7**	–20	70	75
12.^e^	**L7**	–40	60	84

a Reaction conditions: quinolone (0.1 mmol), CuI (10 mol %), ligand (12 mol %), toluene (1 mL), alkyne (1.3 equiv), DIPEA (1.6 equiv), TBSOTf (1.2 equiv).

b Isolated yields after purification.

c Enantiomeric excess determined by separation on chiral UHPLC.

d TMSOTf used in place of TBSOTf.

e TBSOTf added at −78 °C.

All the “Phim” ligands that were tested in the reaction gave moderate yields and low to moderate levels of enantioselectivity (entries 1–5). PHOX type ligands are used widely in enantioselective catalysis,^29^ so (*S*)-^t^Bu-PHOX (**L6**) was also tested, but this afforded both a reduced yield and enantioselectivity compared to the Phim ligands (entry 6). Phim ligand **L4** had afforded the best result and so was taken forward for further testing. The Lewis acid was changed from TBSOTf to the sterically smaller TMSOTf, and this resulted in an increase in yield to 90% but a much reduced enantioselectivity of 11% ee (entry 7). Next the temperature was lowered in 20 °C increments with results showing an increase in enantioselectivity at reduced temperatures; unfortunately, this was also accompanied by a significant reduction in the yield obtained (entries 8 and 9).

**Figure 2:**
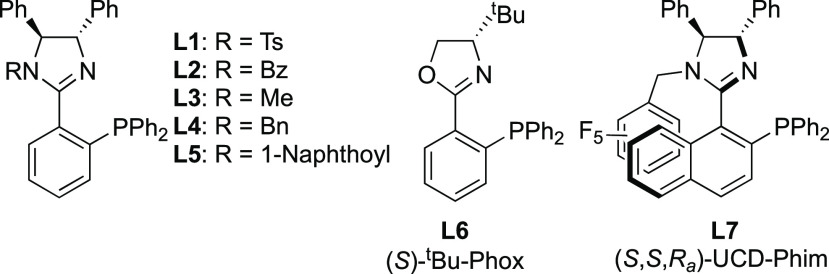
Ligands **L1**–**L7** tested.

We then tested (*S*,*S*,*R*_a_)-UCD-Phim (**L7**), a ligand that we had previously found to display a high degree of enantiocontrol in copper-catalyzed A^3^ coupling reactions.^30,31^ This resulted in a significant increase in both yield and enantioselectivity (entry 10). Further improvements were seen when the TBSOTf was added at −78 °C followed by warming of the reaction to −20 °C (entry 11). Optimal conditions were obtained when the temperature was reduced to −40 °C, giving a yield of 60% and an ee of 84% (entry 12). Further variation of the copper salt, base, solvent, reactant/reagent equivalents, concentration, and reaction time led to reductions in both yield and enantioselectivities (see SI for details).

The optimal reaction conditions were successfully applied to a range of aromatic alkynes with substitution being tolerated in the ortho, meta, and para positions (**3b**–**d**), with the best result being with the *m*-tolyl alkyne (**3c**), giving a yield of 92% and an ee of 97% (Scheme 2). It is supposed that the steric clash between the phenyl group on the imidazoline ring of the ligand and the methyl group on the phenyl acetylene leads to an increased ee. Both electron-withdrawing and electron-donating groups on the aromatic ring were well tolerated (**3e**–**h**) with the aryl halides acting as potential handles for further functionalization. Two precursors for Hancock alkaloids also gave moderate yields and good enantioselectivities (**3i** and **3j**). A bulky anthracenyl substituent could be used in the reaction while still maintaining a moderate ee (**3k**). A high enantioselectivity could also be obtained with the thiophene-containing alkyne (**3l**). Nonaromatic alkynes resulted in reduced yields and enantioselectivities (**3m**–**p**) apart from TMS-acetylene and 1-pentyne (**3m** and **3p**) which gave ee’s of 77% and 73%, respectively, and were similar to many of the aryl alkynes tested. Poorer results for nonaromatic alkynes showed a similar trend to what is commonly seen in the literature.^32^

**Scheme 2:**
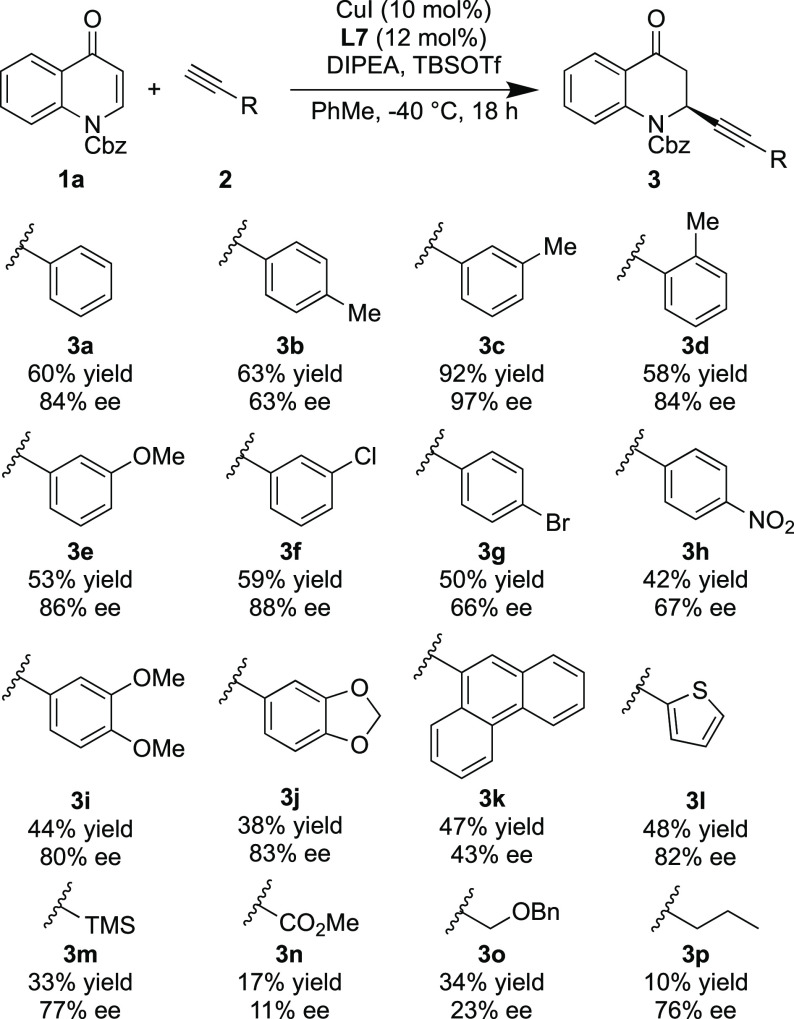
Scope of Alkynes Reaction conditions: quinolone (0.2 mmol), CuI (10 mol %), (*S*,*S*,*R*_a_)-UCD-Phim (12 mol %), toluene (2 mL), alkyne (1.3 equiv), DIPEA (1.6 equiv), TBSOTf (1.2 equiv).

A range of quinolones (**1b**–**i**) with varying substitution were next tested using the optimal reaction conditions employing *m*-tolylacetylene **2c** as the alkyne (Scheme 3). Both electron-donating and electron-withdrawing groups were well tolerated at the quinolone 6-position (**3q**–**u**). Disubstitution at the 5,7-position (**3v**) led to a moderate yield but a good enantioselectivity of 84% ee. Surprisingly, the benzodioxole group was not well tolerated, and a significantly reduced yield of 15% and enantioselectivity of 28% ee were obtained for this product (**3w**). As had previously been noted by Harutyunyan,^17^ substitution at the quinolone 8-position is not tolerated in the reaction and no conversion to product **3x** was observed, which is likely due to steric factors. A protected 4(1*H*)-pyridinone was also tested as a substrate but again no conversion to product **3y** was observed. In all cases with reduced yields, recovered starting material was obtained with little to no decomposition observed.

**Scheme 3:**
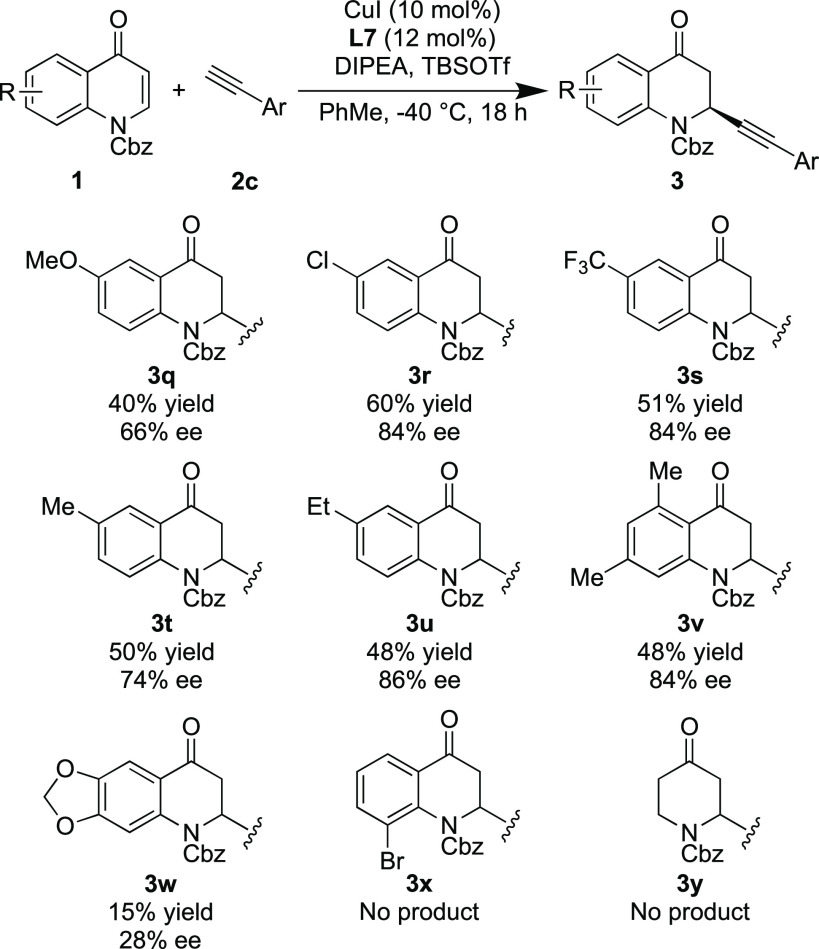
Scope of Quinolones Reaction conditions: quinolone (0.2 mmol), CuI (10 mol %), (*S*,*S*,*R*_a_)-UCD-Phim (12 mol %), toluene (2 mL), *m*-tolylacetylene (1.3 equiv), DIPEA (1.6 equiv), TBSOTf (1.2 equiv).

With a broad substrate scope established, it was necessary to assign the absolute configuration of the products obtained, and this was done through synthesis of a natural product. To demonstrate the robustness of the reaction conditions, the reaction was carried out on a 1 mmol scale using quinolone **1a** and 4-ethynyl-1,2-dimethoxybenzene **2i**. A yield of 56% and ee of 70% for **3i** was observed (Scheme 4). These results compared favorably with those which had previously been obtained in our substrate scope, showing that the reaction is amenable to scale up. This alkynylated product was then recrystallized to an ee of 93% and taken forward to a global reduction/deprotection using 10% Pd/C, followed by methylation to afford the natural product (+)-cuspareine **4**. The optical rotation of our synthetic cuspareine was obtained and compared to the literature to unambiguously assign the configuration and by inference those of the preferred enantiomer in the remainder of alkynated products.^6^ The same synthetic procedure was carried out using unsubstituted quinolone **1a** and 5-ethynylbenzo[*d*][1,3]dioxole **2j** on a 0.5 mmol scale with comparable results being obtained with a yield of 43% and an ee of 83% observed for **3j**, which was transformed in two further steps to the natural product (+)-galipinine **5**.

**Scheme 4:**
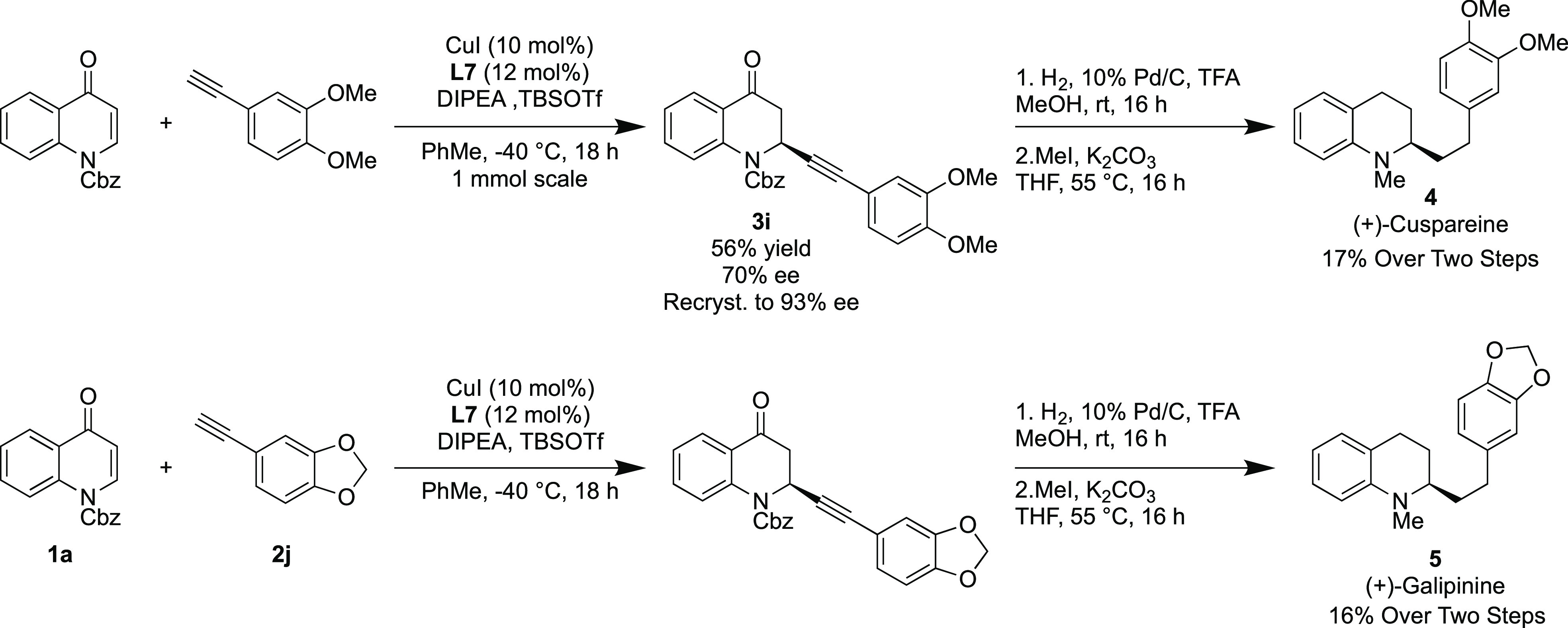
A 1 mmol Scale Synthesis of Natural Products

When ethyl 3-methyl-4-oxoquinoline-1(4*H*)-carboxylate (**7**) was subjected to the standard reaction conditions, no formation of the silyl enol ether was observed, most likely due to the increased steric congestion present at the reaction center. Thankfully, when the sterically smaller TMSOTf was used, the silyl enol ether intermediate was formed, enabling formation of the alkynylated product **8** in poor enantioselectivity but as a single diastereomer (Scheme 5).

**Scheme 5:**
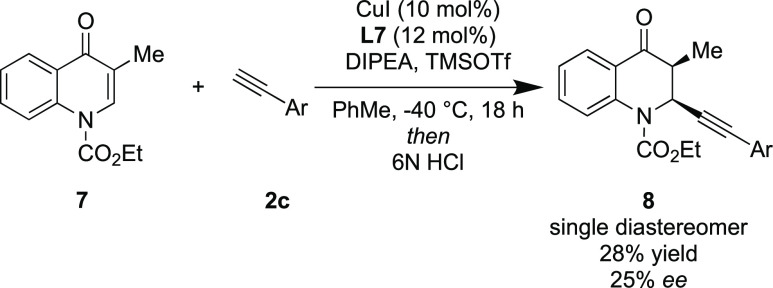
Synthesis of a Single Diastereomer

In conclusion, the P,N ligand, (*S*,*S*,*R*_a_)-UCD-Phim, has successfully been applied in the enantioselective alkynylation of quinolones (24 examples), affording a maximum yield of 92% and levels of enantioselectivity of up to 97% ee. The reaction was tolerant of alkynes and quinolones possessing a broad range of electron-withdrawing and -donating groups. The applicability of the reaction has further been demonstrated through its amenability to scale up and its use in the synthesis of two natural products, (+)-cuspareine **4** and (+)-galipinine **5**.

## Experimental Section

### General Information

^1^H NMR spectroscopy: ^1^H and ^13^C NMR spectra were obtained using Varian VNMRS 400, 500, and 600 MHz spectrometers at room temperature. Proton and carbon chemical shifts are quoted in ppm. ^1^H NMR spectra were recorded using an internal deuterium lock for the residual protons in CDCl_3_ (δ 7.26). ^13^C NMR spectra were recorded using an internal deuterium lock in CDCl_3_ (δ 77.0). Assignments were determined either on the basis of unambiguous chemical shift or coupling patterns, COSY, HSQC, and/or NOESY experiments. Peak multiplicities are defined as s = singlet, d = doublet, t = triplet, q = quartet, m = multiplet, br = broad; coupling constants (*J*) are reported to the nearest 0.1 Hz. Infrared spectroscopy: Infrared spectra were recorded on a Varian 3100 FT-IR spectrometer with the sample being prepared as a thin film on a diamond ATR module. Absorption maxima (ν_max_) are quoted in wavenumbers (cm^–1^). Ultra high performance liquid chromatrography: UHPLC was performed on an Shizmadzu UHPLC using a Chiralcel-OD, AD, or OJ column. Mass spectrometry: High-resolution mass spectra (HRM*S*) were recorded using a Waters Micromass LCT time-of-flight mass spectrometer. Optical rotation: Optical rotation measurements were recorded using a Schmidt-Haensch Unipol L2000 polarimeter at 589 nm and are quoted in units of deg cm^3^ dm^–1^ g^–1^. Reagents, solvents and techniques: Reagents were purchased from Sigma-Aldrich, Fischer, Acros, or Fluorochem and used without further purification unless otherwise stated. Dry tetrahydrofuran obtained from a Puresol Grubbs system unless otherwise stated. Toluene was dried over 3 Å moleclualr sieves. When appropriate, reactions were performed under a nitrogen atmosphere with oven-dried glassware. Oxygen-free nitrogen was supplied by BOC gases and used without further drying. Column chromatography was performed with Merck Kieselgel 60 F254 (230–400 mesh) silica gel. Thin-layer chromatography was performed on aluminum sheets precoated plates with silica gel 60 F254 or aluminum oxide 60 F254. The plates were visualized with ultraviolet fluorescence. Solvent was removed from solutions using a Büchi rotary evaporator with an integrated vacuum pump.

### General Procedure C for Asymmetric Alkynylation of Quinolones

A flame-dried, nitrogen backfilled 10 mL Schlenk tube was charged with CuI (0.0019 g, 0.020 mmol, 0.10 equiv) and (*S*,*S*,*R*_a_)-UCD-Phim (0.0171 g, 0.024 mmol, 0.12 equiv). Toluene (1 mL, 0.2 M) was added to the vessel and stirred at room temperature for 30 min. DIPEA (0.056 mL, 0.32 mmol, 1.60 equiv), the alkyne (1.3 equiv, 0.26 mmol), and the quinolone substrate (1 equiv, 0.20 mmol) were added sequentially. The remaining toluene (1 mL, combined 0.1 M) was added. The reaction was cooled to −78 °C, and the TBSOTf (0.042 mL, 0.24 mmol, 1.2 equiv) was added dropwise. The reaction mixture was warmed to −40 °C and stirred for 18 h. The reaction was brought to room temperature, 6 N HCl (2 mL) was added, and the mixture was stirred for 2 h. The aqueous layer was extracted with CH_2_Cl_2_ (3 × 5 mL). The organics were dried over sodium sulfate and filtered. The organics were removed in vacuo. The crude residue was purified by flash column chromatography (0–20% EtOAc in c-Hex) to afford the pure dihydroquinolone.

#### Benzyl (*S*)-4-Oxo-2-(phenylethynyl)-3,4-dihydroquinoline-1(2*H*)-carboxylate (**3a**)^17^

Synthesized as per general procedure C using benzyl 4-oxoquinoline-1(4*H*)-carboxylate **1a** and phenyl acetylene. The product was isolated as a white solid (46 mg, 60%, 84% ee). ^1^H NMR (400 MHz, CDCl_3_) δ 8.05 (dd, *J* = 7.8, 1.7 Hz, 1H), 7.85 (d, *J* = 8.4 Hz, 1H), 7.54 (ddd, *J* = 8.6, 7.3, 1.8 Hz, 1H), 7.46–7.34 (m, 5H), 7.28–7.12 (m, 6H), 6.08 (dd, *J* = 5.5, 2.1 Hz, 1H), 5.39 (d, *J* = 12.2 Hz, 1H), 5.30 (d, *J* = 12.2 Hz, 1H), 3.14 (dd, *J* = 17.2, 5.5 Hz, 1H), 2.96 (dd, *J* = 17.1, 2.1 Hz, 1H). ^13^C{^1^H} NMR (101 MHz, CDCl_3_) δ 192.1, 153.2, 141.2, 135.6, 134.6, 131.9, 128.9, 128.8, 128.7, 128.4, 127.2, 125.0, 124.7, 124.3, 121.9, 85.6, 85.0, 68.8, 47.4, 44.7. [α]^20^_D_ −8.28 (*c* = 1.00, CHCl_3_).

#### Benzyl (*S*)-4-Oxo-2-(*p*-tolylethynyl)-3,4-dihydroquinoline-1(2*H*)-carboxylate (**3b**)^17^

Synthesized as per general procedure C using benzyl 4-oxoquinoline-1(4*H*)-carboxylate **1a** and 3-methylphenylacetylene. The product was isolated as a white solid (50 mg, 63%, 63% ee). ^1^H NMR (500 MHz, CDCl_3_) δ 8.05 (dd, *J* = 7.9, 1.7 Hz, 1H), 7.86 (d, *J* = 8.4 Hz, 1H), 7.54 (ddd, *J* = 8.6, 7.3, 1.7 Hz, 1H), 7.49–7.33 (m, 5H), 7.22 (ddd, *J* = 8.0, 7.3, 1.1 Hz, 1H), 7.08 (d, *J* = 8.2 Hz, 2H), 7.01 (dd, *J* = 8.5, 0.8 Hz, 2H), 6.08 (dd, *J* = 5.5, 2.1 Hz, 1H), 5.39 (d, *J* = 12.2 Hz, 1H), 5.34–5.16 (m, 1H), 3.14 (dd, *J* = 17.1, 5.5 Hz, 1H), 2.96 (dd, *J* = 17.1, 2.1 Hz, 1H), 2.29 (s, 3H). ^13^C{^1^H} NMR (101 MHz, CDCl_3_) δ 192.2, 153.2, 141.2, 139.0, 135.6, 134.6, 131.8, 129.0, 128.9, 128.7, 128.4, 127.2, 125.0, 124.7, 124.3, 118.9, 85.1, 84.8, 68.7, 47.4, 44.8, 21.6. [α]^20^_D_ −55.42 (*c* = 1.00, CHCl_3_).

#### Benzyl (*S*)-4-Oxo-2-(*m*-tolylethynyl)-3,4-dihydroquinoline-1(2*H*)-carboxylate (**3c**)^17^

Synthesized as per general procedure C using benzyl 4-oxoquinoline-1(4*H*)-carboxylate **1a** and 3-methylphenylacetylene. The product was isolated as a white solid (72 mg, 92%, 97% ee). ^1^H NMR (400 MHz, CDCl_3_) δ 8.05 (dd, *J* = 7.9, 1.7 Hz, 1H), 7.85 (d, *J* = 8.4 Hz, 1H), 7.54 (ddd, *J* = 8.6, 7.2, 1.7 Hz, 1H), 7.47–7.30 (m, 5H), 7.22 (ddd, *J* = 8.2, 7.3, 1.1 Hz, 1H), 7.13–7.03 (m, 2H), 7.02–6.96 (m, 2H), 6.07 (dd, *J* = 5.5, 2.1 Hz, 1H), 5.39 (d, *J* = 12.2 Hz, 1H), 5.30 (d, *J* = 12.2 Hz, 1H), 3.14 (dd, *J* = 17.1, 5.5 Hz, 1H), 2.95 (dd, *J* = 17.1, 2.1 Hz, 1H), 2.24 (s, 3H). ^13^C{^1^H} NMR (101 MHz, CDCl_3_) δ 192.1, 153.2, 141.2, 138.0, 135.6, 134.6, 132.5, 129.7, 129.0, 128.9, 128.7, 128.4, 128.2, 127.2, 125.0, 124.7, 124.3, 121.7, 85.2, 68.8, 47.4, 44.7, 21.2. [α]^20^_D_ −48.87 (*c* = 1.00, CHCl_3_).

#### Benzyl (*S*)-4-Oxo-2-(*o*-tolylethynyl)-3,4-dihydroquinoline-1(2*H*)-carboxylate (**3d**)^17^

Synthesized as per general procedure C using benzyl 4-oxoquinoline-1(4*H*)-carboxylate **1a** and 2-methylphenylacetylene. The product was isolated as a white solid (44 mg, 58%, 84% ee). ^1^H NMR (400 MHz, CDCl_3_) δ 8.05 (dd, *J* = 7.6, 1.7 Hz, 1H) 7.85 (d, *J* = 8.6 Hz, 1H), 7.54 (ddd, *J* = 8.6, 7.6, 5.5 Hz, 1H), 7.45–7.34 (m, 5H), 7.24–7.11 (m, 3H), 7.08–7.00 (m, 2H), 6.10 (dd, *J* = 5.5, 2.1 Hz, 1H), 5.39 (d, *J* = 12.2 Hz, 1H), 5.30 (d, *J* = 12.2 Hz, 1H), 3.16 (dd, 17.1, 5.4 Hz, 1H), 2.97 (dd, *J* = 17.1, 2.1 Hz), 1.94 (s, 3H). ^13^C{^1^H} NMR (101 MHz, CDCl_3_) δ 192.1, 153.1, 141.3, 140.7, 135.6, 134.5, 131.9, 129.4, 128.9, 128.8, 128.7, 128.4, 127.2, 125.5, 125.2, 124. 7, 121.6, 89.5, 84.0, 77.4, 68.8, 47.6, 45.0, 20.2. [α]^20^_D_ −63.12 (*c* = 1.00, CHCl_3_).

#### Benzyl (*S*)-2-((3-Methoxyphenyl)ethynyl)-4-oxo-3,4-dihydroquinoline-1(2*H*)-carboxylate (**3e**)^17^

Synthesized as per general procedure C using benzyl 4-oxoquinoline-1(4*H*)-carboxylate **1a** and 3-ethynlanisole. The product was isolated as a white solid (43 mg, 53%, 86% ee). ^1^H NMR (400 MHz, CDCl_3_) δ 8.04 (dd, *J* = 7.8, 1.7 Hz, 1H), 7.85 (d, *J* = 8.4 Hz, 1H), 7.58–7.49 (m, 1H), 7.47–7.32 (m, 5H), 7.21 (ddd, *J* = 8.2, 7.3, 1.1 Hz, 1H), 7.14–7.06 (m, 1H), 6.84–6.73 (m, 2H), 6.69 (dd, *J* = 2.7, 1.4 Hz, 1H), 6.07 (dd, *J* = 5.6, 2.1 Hz, 1H), 5.38 (d, *J* = 12.2 Hz, 1H), 5.33–5.26 (m, 1H), 3.72 (s, 3H), 3.14 (dd, *J* = 17.1, 5.6 Hz, 1H), 2.96 (dd, *J* = 17.2, 2.1 Hz, 1H). ^13^C{^1^H} NMR (101 MHz, CDCl_3_) δ 192.0, 159.3, 153.2, 141.2, 135.6, 134.6, 129.4, 128.9, 128.7, 128.4, 127.2, 125.0, 124.7, 124.5, 124.3, 122.9, 116.8, 115.4, 85.4, 84.9, 68.8, 55.4, 47.4, 44.7. [α]^20^_D_ −147.63 (*c* = 1.00, CHCl_3_).

#### Benzyl (*S*)-2-((3-Chlorophenyl)ethynyl)-4-oxo-3,4-dihydroquinoline-1(2*H*)-carboxylate (**3f**)^17^

Synthesized as per general procedure C using benzyl 4-oxoquinoline-1(4*H*)-carboxylate **1a** and 3-chlorophenylacetylene. The product was isolated as a white solid (49 mg, 59% yield, 88% ee). ^1^H NMR (400 MHz, CDCl_3_) δ 8.05 (dd, *J* = 7.9, 1.7 Hz, 1H), 7.85 (d, *J* = 8.4 Hz, 1H), 7.55 (ddd, *J* = 8.7, 7.3, 1.8 Hz, 1H), 7.47–7.33 (m, 5H), 7.25–7.20 (m, 2H), 7.18–7.10 (m, 2H), 7.05 (dt, *J* = 7.7, 1.4 Hz, 1H), 6.08 (dd, *J* = 5.6, 2.1 Hz, 1H), 5.39 (d, *J* = 12.2 Hz, 1H), 5.30 (d, *J* = 12.2 Hz, 1H), 3.14 (dd, *J* = 17.2, 5.6 Hz, 1H), 2.95 (dd, *J* = 17.2, 2.1 Hz, 1H). ^13^C{^1^H} NMR (101 MHz, CDCl_3_) δ 191.9, 153.1, 141.1, 135.5, 134.7, 134.2, 131.8, 130.1, 129.5, 129.1, 128.9, 128.7, 128.4, 127.3, 124.8, 124.2, 123.6, 86.8, 83.5, 68.9, 47.3, 44.5. [α]^20^_D_ −126.35 (*c* = 1.00, CHCl_3_).

#### Benzyl (*S*)-2-((4-Bromophenyl)ethynyl)-4-oxo-3,4-dihydroquinoline-1(2*H*)-carboxylate (**3g**)

Synthesized as per general procedure C using benzyl 4-oxoquinoline-1(4*H*)-carboxylate **1a** and 1-bromo-4-ethynylbenzene. The product was isolated as a white solid (46 mg, 50%, 76% ee). ^1^H NMR (400 MHz, CDCl_3_) δ 8.04 (dd, *J* = 7.7, 1.7 Hz, 1H), 7.84 (d, *J* = 8.5 Hz, 1H), 7.54 (ddd, *J* = 8.6, 7.2, 1.7 Hz, 1H), 7.45–7.31 (m, 7H), 7.22(dt, *J* = 8.0, 7.3, 1.1 Hz, 1H), 7.03 (dt, *J* = 8.5, 2.2 Hz, 1H), 6.06 (dd, *J* = 5.5, 2.1 Hz, 1H), 5.38 (d, *J* = 12.2 Hz, 1H), 5.29 (d, *J* = 12.2, 1H), 3.14 (dd, *J* = 17.1, 5.6 Hz, 1H) 2.94 (17.1, 2.1 Hz, 1H). ^13^C{^1^H} NMR (101 MHz, CDCl_3_) δ 191.9, 153.1, 141.1, 135.5, 134.7, 133.4, 131.6, 128.9, 128.7, 128.4, 127.3, 124.9, 124.8, 124.2, 123.2, 120.8, 86.8, 83.9, 68.8, 47.4, 44.5, 31.1, 29.8. HRMS (ESI) [M + H]^+^ calcd 460.0542 for [C_25_H_19_BrNO_3_]^(+)^ found 460.0541. IR ν(cm^–1^): 3069, 3037, 2925, 2852, 1687, 1517, 1299, 1217, 851, 748, 696. Mp 161–163 °C. [α]^20^_D_ −74.02 (*c* = 1.00, CHCl_3_).

#### Benzyl (*S*)-2-((4-Nitrophenyl)ethynyl)-4-oxo-3,4-dihydroquinoline-1(2*H*)-carboxylate (**3h**)

Synthesized as per general procedure C using benzyl 4-oxoquinoline-1(4*H*)-carboxylate **1a** and 1-ethynyl-4-nitrobenzene. The product was isolated as a white solid (36 mg, 42%, 67% ee). ^1^H NMR (400 MHz, CDCl_3_) δ 8.11–8.02 (m, 3H), 7.85 (d, *J* = 8.4 Hz, 1H), 7.56 (ddd, *J* = 8.4, 7.3, 1.7 Hz, 1H), 7.48–7.34 (m, 5H), 7.34–7.29 (m, 2H), 7.25–7.21 (m, 1H), 6.13 (dd, *J* = 5.6, 2.1 Hz, 1H), 5.39 (d, *J* = 12.1 Hz, 1H), 5.30 (d, *J* = 12.2 Hz, 1H), 3.18 (dd, *J* = 17.2, 5.7 Hz, 1H), 2.97 (dd, *J* = 17.2, 2.1 Hz, 1H). ^13^C{^1^H} NMR (101 MHz, CDCl_3_) δ 191.6, 153.1, 147.5, 141.0, 135.4, 134.8, 132.8, 128.9, 128.8, 128.6, 128.5, 127.4, 125.0, 124.7, 124.2, 123.6, 90.9, 83.0, 69.0, 47.4, 44.3. HRMS (ESI) [M + H]^+^ calc. 427.1288 for [C_25_H_19_N_2_O_5_]^(+)^ found 427.1288. IR ν(cm^–1^): 3069, 3037, 2925, 2852, 1687, 1517, 1299, 1217, 851, 748, 696. Mp 130–133 °C. [α]20D −32.89 (*c* = 1.00, CHCl_3_).

#### Benzyl (*S*)-2-((3,4-Dimethoxyphenyl)ethynyl)-4-oxo-3,4-dihydroquinoline-1(2*H*)-carboxylate (**3i**)^17^

Synthesized as per general procedure C using benzyl 4-oxoquinoline-1(4*H*)-carboxylate **1a** and 4-ethynyl-1,2-dimethoxybenzene. The product was isolated as a yellow solid (39 mg, 44%, 80% ee). ^1^H NMR (400 MHz, CDCl_3_) δ 8.05 (dd, *J* = 7.8, 1.7 Hz, 1H), 7.85 (d, *J* = 8.4 Hz, 1H), 7.58–7.49 (m, 1H), 7.47–7.31 (m, 5H), 7.21 (ddd, *J* = 8.2, 7.3, 1.1 Hz, 1H), 6.79 (dd, *J* = 8.3, 1.9 Hz, 1H), 6.71–6.64 (m, 2H), 6.07 (dd, *J* = 5.5, 2.1 Hz, 1H), 5.38 (d, *J* = 12.2 Hz, 1H), 5.29 (d, *J* = 12.2 Hz, 1H), 3.82 (s, 3H), 3.78 (s, 3H), 3.14 (dd, *J* = 17.1, 5.6 Hz, 1H), 2.95 (dd, *J* = 17.1, 2.1 Hz, 1H). ^13^C{^1^H} NMR (101 MHz, CDCl_3_) δ 192.2, 153.2, 149.9, 148.6, 141.2, 135.6, 134.6, 128.9, 128.7, 128.4, 127.2, 125.4, 124.9, 124.6, 124.3, 114.5, 114.0, 110.9, 85.1, 84.1, 68.8, 55.99, 55.98, 47.5, 44.8. [α]^20^_D_ −69.91 (*c* = 1.00, CHCl_3_).

#### Benzyl (*S*)-2-(Benzo[*d*][1,3]dioxol-5-ylethynyl)-4-oxo-3,4-dihydroquinoline-1(2*H*)-carboxylate (**3j**)

Synthesized as per general procedure C using benzyl 4-oxoquinoline-1(4*H*)-carboxylate **1a** and 5-ethynylbenzo[*d*][1,3]dioxole. The product was isolated as a yellow solid (32 mg, 38%, 83% ee). ^1^H NMR (400 MHz, CDCl_3_) δ 8.04 (ddd, *J* = 7.8, 1.7, 0.5 Hz, 1H), 7.84 (d, *J* = 8.4 Hz, 1H), 7.53 (ddd, *J* = 8.4, 7.2, 1.7 Hz, 1H), 7.46–7.32 (m, 5H), 7.21 (ddd, *J* = 7.8, 7.2, 1.1 Hz, 1H), 6.70 (dd, *J* = 8.0, 1.6 Hz, 1H), 6.63 (dd, *J* = 8.1, 0.5 Hz, 1H), 6.61–6.59 (m, 1H), 6.04 (dd, *J* = 5.5, 2.1 Hz, 1H), 5.91 (s, 2H), 5.38 (d, *J* = 12.2 Hz, 1H), 5.29 (d, *J* = 12.2 Hz, 1H), 3.12 (dd, *J* = 17.1, 5.5 Hz, 1H), 2.93 (dd, *J* = 17.1, 2.1 Hz, 1H); ^13^C{^1^H} NMR (101 MHz, CDCl_3_) δ 192.1, 153.2, 148.3, 147.4, 141.2, 135.6, 134.6, 128.9, 128.7, 128.4, 127.2, 126.7, 125.0, 124.7, 124.3, 115.1, 111.9, 108.4, 101.4, 84.9, 83.9, 68.8, 47.4, 44.8; HRMS (ESI) [M + H]^+^ calc. 426.1335 for [C_26_H_20_NO_5_]^(+)^ found 426.1336; IR ν(cm^–1^): 3067, 3039, 2991, 2901, 2233, 1690, 1597, 1597, 1477, 1213, 1034, 758, 733; Mp 149–152 °C; [α]^20^_D_ −51.78 (*c* = 1.00, CHCl_3_).

#### Benzyl (*S*)-4-Oxo-2-(phenanthren-9-ylethynyl)-3,4-dihydroquinoline-1(2*H*)-carboxylate (**3k**)

Synthesized as per general procedure C using benzyl 4-oxoquinoline-1(4*H*)-carboxylate **1a** and 9-ethynylphenanthrene. The product was isolated as a white solid (45 mg, 47%, 72% ee). ^1^H NMR (400 MHz, CDCl_3_) δ 8.48 (dd, *J* = 8.2, 3.7 Hz, 2H), 8.14 (dd, *J* = 7.9, 1.7 Hz, 1H), 7.93 (d, *J* = 8.4 Hz, 1H), 7.78 (s, 1H), 7.76 (dd, *J* = 7.9, 1.4 Hz, 1H), 7.66–7.52 (m, 5H), 7.49–7.35 (m, 6H), 7.31 (dd, *J* = 8.3, 7.4, 1.0 Hz, 1H), 6.24 (dd, *J* = 5.5, 2.1 Hz, 1H), 5.42 (d, *J* = 12.2 Hz, 1H), 5.33 (d, *J* = 12.2 Hz, 1H), 3.26 (dd, *J* = 17.2, 5.5 Hz, 1H) 3.10 (dd, *J* = 17.2, 2.2 Hz, 1H). ^13^C{^1^H} NMR (101 MHz, CDCl_3_) δ 192.0, 153.0, 141.3, 135.4, 134.6, 132.2, 130.80, 130.78, 130.3, 129.9, 128.7, 128.6, 128.5, 128.3, 127.7, 127.3, 126.94, 126.91, 126.3, 125.2, 124.8, 124.4, 122.6, 122.5, 118.1, 90.0, 83.3, 68.7, 47.7, 44.9. HRMS (ESI) [M + H]^+^ calc. 482.1750 for [C_33_H_24_NO_3_]^(+)^ found 482.1750. IR ν(cm^–1^): 3072, 3034, 2961, 2922, 2852, 2224, 1715, 1691, 1595, 1477, 1455, 1389, 1296, 1215, 757. Mp 179–181 °C; [α]^20^_D_ −71.51 (*c* = 1.00, CHCl_3_).

#### Benzyl (*S*)-4-Oxo-2-(thiophen-2-ylethynyl)-3,4-dihydroquinoline-1(2*H*)-carboxylate (**3l**)

Synthesized as per general procedure C using benzyl 4-oxoquinoline-1(4*H*)-carboxylate **1a** and 2-ethynylthiophene. The product was isolated as a yellow solid (37 mg, 48%, 82% ee). ^1^H NMR (400 MHz, CDCl_3_) δ 8.04 (dd, *J* = 7.9, 1.7 Hz, 1H), 7.85 (d, *J* = 8.4 Hz, 1H), 7.54 (ddd, *J* = 8.5, 7.3, 1.7 Hz, 1H), 7.46–7.33 (m, 5H), 7.25–7.14 (m, 2H), 7.01 (dd, *J* = 3.7, 1.2 Hz, 1H), 6.86 (dd, *J* = 5.1, 3.6 Hz, 1H), 6.10 (dd, *J* = 5.6, 2.1 Hz, 1H), 5.38 (d, *J* = 12.2 Hz, 1H), 5.29 (d, *J* = 12.2 Hz, 1H), 3.14 (dd, *J* = 17.2, 5.7 Hz, 1H), 2.95 (dd, *J* = 17.2, 2.1 Hz, 1H). ^13^C{^1^H} NMR (101 MHz, CDCl_3_) δ 191.9, 153.1, 141.1, 135.6, 134.7, 133.0, 128.9, 128.7, 128.4, 127.7, 127.3, 127.0, 124.83, 124.77, 124.3, 121.7, 89.5, 78.2, 68.8, 47.6, 44.5. HRMS (ESI) [M + H]^+^ calc. 388.1001 for [C_23_H_18_NO_3_S]^(+)^ found 388.1001. IR ν(cm^–1^): 3063, 3038, 2922, 2854, 2226, 1716, 1693, 1596, 1298, 1265, 1221, 995, 696. Mp 120–124 °C. [α]^20^_D_ −28.58 (*c* = 1.00, CHCl_3_).

#### Benzyl (*S*)-4-Oxo-2-((trimethylsilyl)ethynyl)-3,4-dihydroquinoline-1(2*H*)-carboxylate (**3m**)

Synthesized as per general procedure C using benzyl 4-oxoquinoline-1(4*H*)-carboxylate **1a** and TMS-acetylene. The product was isolated as a white solid (25 mg, 33%, 77% ee). ^1^H NMR (400 MHz, CDCl_3_) δ 8.01 (ddd, *J* = 7.8, 1.7, 0.5 Hz, 1H), 7.79 (d, *J* = 8.4 Hz, 1H), 7.57–7.48 (m, 1H), 7.45–7.31 (m, 5H), 7.21 (ddd, *J* = 7.8, 7.3, 1.1 Hz, 1H), 5.81 (dd, *J* = 5.5, 2.1 Hz, 1H), 5.35 (d, *J* = 12.3 Hz, 1H), 5.26 (d, *J* = 12.3 Hz, 1H), 3.04 (dd, *J* = 17.2, 5.6 Hz, 1H), 2.85 (dd, *J* = 17.2, 2.1 Hz, 1H), −0.05 (s, 10H). ^13^C{^1^H} NMR (101 MHz, CDCl_3_) δ 192.1, 153.1, 141.2, 135.6, 134.4, 128.9, 128.7, 128.4, 127.1, 125.2, 124.7, 124.4, 101.9, 90.7, 68.7, 47.4, 44.8, −0.4. HRMS (ESI) [M + H]^+^ calc. 378.1519 for [C_22_H_24_NO_3_Si]^(+)^ found 378.1521; IR ν(cm^–1^): 3071, 3036, 2953, 2924, 2854, 2166, 1693, 1601, 1480, 1392, 1011, 840, 758, 735, 692, 645. Mp 87–90 °C. [α]^20^_D_ −28.30 (*c* = 1.00, CHCl_3_).

#### Benzyl (*S*)-2-(3-Methoxy-3-oxoprop-1-yn-1-yl)-4-oxo-3,4-dihydroquinoline-1(2*H*)-carboxylate (**3n**)^17^

Synthesized as per general procedure C using benzyl 4-oxoquinoline-1(4*H*)-carboxylate **1a** and methyl propiolate. The product was isolated as a white solid (12 mg, 17%, 11% ee). ^1^H NMR (400 MHz, CDCl_3_) δ 8.03 (dd, *J* = 7.9, 1.7 Hz, 1H), 7.81 (d, *J* = 8.4 Hz, 1H), 7.60–7.51 (m, 1H), 7.43–7.33 (m, 5H), 7.28–7.20 (m, 1H), 6.03 (dd, *J* = 6.0, 2.0 Hz, 1H), 5.36 (d, *J* = 12.2 Hz, 1H), 5.26 (d, *J* = 12.1 Hz, 1H), 3.66 (s, 3H), 3.12 (dd, *J* = 17.4, 5.9 Hz, 1H), 2.91 (dd, *J* = 17.4, 2.0 Hz, 1H). ^13^C{^1^H} NMR (101 MHz, CDCl_3_) δ 190.7, 153.1, 152.9, 140.8, 135.2, 135.0, 128.93, 128.86, 128.5, 127.6, 125.2, 124.5, 124.3, 83.5, 75.6, 69.2, 53.0, 46.5, 43.4. [α]^20^_D_ + 1.37 (*c* = 1.00, CHCl_3_).

#### Benzyl (*S*)-2-(3-(Benzyloxy)prop-1-yn-1-yl)-4-oxo-3,4-dihydroquinoline-1(2*H*)-carboxylate (**3o**)

Synthesized as per general procedure C using benzyl 4-oxoquinoline-1(4*H*)-carboxylate **1a** and ((prop-2-yn-1-yloxy)methyl)benzene. The product was isolated as a pale white oil (29 mg, 34%, 23% ee). ^1^H NMR (400 MHz, CDCl_3_) δ 8.07–8.01 (m, 1H), 7.84 (d, *J* = 8.4 Hz, 1H), 7.58–7.49 (m, 1H), 7.43–7.33 (m, 6H), 7.29–7.27 (m, 2H), 7.20 (ddd, *J* = 8.2, 7.3, 1.1 Hz, 1H), 7.15–7.08 (m, 2H), 5.92 (dq, *J* = 5.7, 1.9 Hz, 1H), 5.37 (d, *J* = 12.3 Hz, 1H), 5.27 (d, *J* = 12.2 Hz, 1H), 4.23 (d, *J* = 1.8 Hz, 2H), 3.97 (d, *J* = 1.8 Hz, 2H), 3.09 (dd, *J* = 17.1, 5.6 Hz, 1H), 2.88 (dd, *J* = 17.1, 2.1 Hz, 1H). ^13^C{^1^H} NMR (101 MHz, CDCl_3_) δ 191.9, 153.1, 141.2, 137.1, 135.5, 134.7, 128.9, 128.7, 128.7, 128.5, 128.4, 128.33, 128.31, 128.2, 128.0, 127.3, 124.9, 124.8, 124.3, 83.2, 80.9, 71.1, 68.8, 56.9, 46.9, 44.6. HRMS (ESI) [M + H]^+^ calc. 426.1699 for [C_27_H_24_NO_4_]^(+)^ found 426.1699. IR ν(cm^–1^): 3063, 3032, 2920, 2852, 1687, 1600, 1478, 1458, 1299, 1217, 1023, 696. [α]^20^_D_ −66.32 (*c* = 1.00, CHCl_3_).

#### Benzyl (*S*)-4-Oxo-2-(pent-1-yn-1-yl)-3,4-dihydroquinoline-1(2*H*)-carboxylate (**3p**)

Synthesized as per general procedure C using benzyl 4-oxoquinoline-1(4*H*)-carboxylate **1a** and ((prop-2-yn-1-yloxy)methyl)benzene. The product was isolated as a pale yellow oil (7 mg, 10%, 73% ee). ^1^H NMR (400 MHz, CDCl_3_) δ 8.00 (dd, *J* = 7.8, 1.7 Hz, 1H), 7.80 (d, *J* = 8.4 Hz, 1H), 7.50 (ddd, *J* = 8.7, 7.2, 1.7 Hz, 1H), 7.42–7.29 (m, 5H), 7.17 (t, *J* = 7.7 Hz, 1H), 5.83–5.76 (m, 1H), 5.33 (d, *J* = 12.3, 1H), 5.24 (d, *J* = 12.3, 1H), 3.01 (dd, *J* = 17.0, 5.4 Hz, 1H), 2.80 (dd, *J* = 17.0, 2.2 Hz, 1H), 1.93 (td, *J* = 7.1, 2.1, 2H), 1.25 (sext, *J* = 7.1 Hz, 2H), 0.67 (t, *J* = 7.1 Hz, 3H). ^13^C{^1^H} NMR (101 MHz, CDCl_3_) δ 192.3, 153.0, 141.1, 135.5, 128.7, 128.5, 128.2, 126.9, 124.9, 124.3, 124.1, 85.7, 76.7, 76.6, 68.4, 46.9, 44.9, 21.6, 20.3, 12.9. HRMS (ESI) [M + H]^+^ calc. 348.1593 for [C_22_H_22_NO_3_]^(+)^ found. 348.1594. IR ν(cm^–1^): 3068, 3034, 2962, 2933, 2904, 2872, 1695, 1690, 1601, 1479, 1387, 1301, 1223, 768, 735. [α]^20^_D_ −19.91 (*c* = 1.00, CHCl_3_).

#### Benzyl (*S*)-6-Methoxy-4-oxo-2-(*m*-tolylethynyl)-3,4-dihydroquinoline-1(2*H*)-carboxylate (**3q**)^17^

Synthesized as per general procedure C using benzyl 6-methoxy-4-oxoquinoline-1(4*H*)-carboxylate **1b** and 3-methylphenylacetylene. The product was isolated as a white solid (34 mg, 40% yield, 66% ee). ^1^H NMR (400 MHz, CDCl_3_) δ 7.74 (d, *J* = 9.1 Hz, 1H), 7.49 (d, *J* = 3.2 Hz, 1H), 7.44–7.33 (m, 5H), 7.17–6.95 (m, 5H), 6.06 (dd, *J* = 5.6, 2.0 Hz, 1H), 5.34 (d, *J* = 12.4 Hz, 1H), 5.27 (d, *J* = 12.3 Hz, 1H), 3.85 (s, 3H), 3.12 (dd, *J* = 17.2, 5.5 Hz, 1H), 2.94 (dd, *J* = 17.2, 2.1 Hz, 1H), 2.24 (s, 3H). ^13^C{^1^H} NMR (101 MHz, CDCl_3_) δ 192.2, 156.5, 153.2, 138.0, 135.7, 134.9, 132.5, 129.7, 129.0, 128.9, 128.7, 128.4, 128.2, 126.0, 125.8, 122.6, 121.8, 108.8, 85.3, 85.0, 68.7, 55.8, 47.4, 44.8, 21.2. [α]^20^_D_ −21.46 (*c* = 1.00, CHCl_3_).

#### Benzyl (*S*)-6-Chloro-4-oxo-2-(*m*-tolylethynyl)-3,4-dihydroquinoline-1(2*H*)-carboxylate (**3r**)

Synthesized as per general procedure C using benzyl 6-chloro-4-oxoquinoline-1(4*H*)-carboxylate **1c** and 3-methylphenylacetylene. The product was isolated as a white solid (52 mg, 60%, 84% ee). ^1^H NMR (400 MHz, CDCl_3_) δ 8.00 (d, *J* = 2.6 Hz, 1H), 7.85 (d, *J* = 9.0 Hz, 1H), 7.47 (dd, *J* = 9.0, 2.7 Hz, 1H), 7.45–7.35 (m, 5H), 7.14–7.06 (m, 2H), 7.04–6.99 (m, 2H), 6.07 (dd, *J* = 5.4, 2.1 Hz, 1H), 5.38 (d, *J* = 12.2 Hz, 1H), 5.30 (d, *J* = 12.2 Hz, 1H), 3.11 (dd, *J* = 17.2, 5.5 Hz, 1H), 2.96 (dd, *J* = 17.1, 2.2 Hz, 1H), 2.25 (s, 3H). ^13^C{^1^H} NMR (101 MHz, CDCl_3_) δ 190.9, 153.0, 139.7, 138.1, 135.4, 134.4, 132.5, 130.5, 129.9, 128.9, 128.8, 128.5, 128.3, 126.8, 125.9, 125.8, 121.5, 85.5, 84.6, 69.0, 47.3, 44.5, 21.2. HRMS (ESI) [M + H]^+^ calc. 430.1204 for [C_26_H_21_ClNO_3_]^(+)^; found 430.1190. IR ν(cm^–1^) 3034, 2916, 1855, 2231, 1717, 1695, 1596, 1211, 1021, 691, 673. Mp 95–100 °C. [α]^20^_D_ −82.36 (*c* = 1.00, CHCl_3_).

#### Benzyl (*S*)- 4-oOxo-2-(*m*-tolylethynyl)-6-(trifluoromethyl)-3,4-dihydroquinoline-1(2*H*)-carboxylate (**3s**)^17^

Synthesized as per general procedure C using benzyl 4-oxo-6-(trifluoromethyl)quinoline-1(4*H*)-carboxylate **1d** and 3-methylphenylacetylene. The product was isolated as a white solid (47 mg, 51%, 84% ee). ^1^H NMR (400 MHz, CDCl_3_) δ 8.26 (d, *J* = 2.3 Hz, 1H), 8.00 (d, *J* = 8.8 Hz, 1H), 7.68 (dd, *J* = 8.8, 2.3 Hz, 1H), 7.41–7.28 (m, 5H), 7.07–6.98 (m, 2H), 6.96–6.89 (m, 2H), 6.02 (dd, *J* = 5.4, 2.2 Hz, 1H), 5.33 (d, *J* = 12.1 Hz, 1H), 5.25 (d, *J* = 12.1 Hz, 1H), 3.07 (dd, *J* = 17.1, 5.4 Hz, 1H), 2.94 (dd, *J* = 17.1, 2,2 Hz, 1H), 2.17 (s, 3H). ^13^C{^1^H} NMR (101 MHz, CDCl_3_) δ 190.8, 152.9, 143.9, 138.2, 135.2, 132.5, 131.0 (q, *J* = 3.6 Hz), 130.0, 129.02, 129.96, 128.9, 128.6, 128.3, 126.9, 124.83, 124.79, 124.5 (d, *J* = 27.6 Hz), 121.3, 85.7, 84.3, 69.2, 47.4, 44.4, 21.2. [α]^20^_D_ −58.71 (*c* = 1.00, CHCl_3_).

#### Benzyl (*S*)-6-Methyl-4-oxo-2-(*m*-tolylethynyl)-3,4-dihydroquinoline-1(2*H*)-carboxylate (**3t**)^17^

Synthesized as per general procedure C using benzyl 6-methyl-4-oxoquinoline-1(4*H*)-carboxylate **1e** and 3-methylphenylacetylene. The product was isolated as a white solid (41 mg, 50%, 74% ee). ^1^H NMR (400 MHz, CDCl_3_) δ 7.86–7.81 (m, 1H), 7.73 (d, *J* = 8.5 Hz, 1H), 7.51–7.30 (m, 6H), 7.13–6.96 (m, 4H), 6.06 (dd, *J* = 5.6, 2.1 Hz, 1H), 5.38 (d, *J* = 12.2 Hz, 1H), 5.28 (d, *J* = 12.2 Hz, 1H), 3.12 (dd, *J* = 17.1, 5.5 Hz, 1H), 2.93 (dd, *J* = 17.1, 2.1 Hz, 1H), 2.35 (s, 3H), 2.24 (s, 3H). ^13^C{^1^H} NMR (101 MHz, CDCl_3_) δ 192.4, 153.2, 138.9, 138.0, 135.7, 135.5, 1345, 132.5, 129.7, 129.0, 128.9, 128.7, 128.4, 128.2, 127.2, 124.7, 124.2, 121.8, 85.3, 85.0, 68.7, 47.4, 44.8, 21.2, 20.8. [α]^20^_D_ −58.71 (*c* = 1.00, CHCl_3_).

#### Benzyl (*S*)-6-Ethyl-4-oxo-2-(*m*-tolylethynyl)-3,4-dihydroquinoline-1(2*H*)-carboxylate (**3u**)

Synthesized as per general procedure C using benzyl 6-ethyl-4-oxoquinoline-1(4*H*)-carboxylate **1f** and 3-methylphenylacetylene. The product was isolated as a white solid (41 mg, 48%, 86% ee). ^1^H NMR (400 MHz, CDCl_3_) δ 7.85 (d, *J* = 2.3 Hz, 1H), 7.74 (d, *J* = 8.6 Hz, 1H), 7.45–7.32 (m, 6H), 7.12–6.94 (m, 4H), 6.04 (dd, *J* = 5.6, 2.1 Hz, 1H), 5.37 (d, *J* = 12.2 Hz, 1H), 5.26 (d, *J* = 12.3 Hz, 1H), 3.10 (dd, *J* = 17.1, 5.6 Hz, 1H), 2.92 (dd, *J* = 17.1, 2.2 Hz, 1H), 2.65 (q, *J* = 7.6 Hz, 2H), 2.22 (s, 3H), 1.30–1.15 (m, 3H). ^13^C{^1^H} NMR (101 MHz, CDCl_3_) δ 192.4, 153.2, 140.7, 139.0, 138.0, 135.7, 134.5, 132.5, 129.6, 129.0, 128.8, 128.6, 128.4, 128.2, 126.0, 124.8, 124.2, 121.8, 85.4, 85.0, 68.7, 47.4, 44.8, 28.2, 21.2, 15.3. HRMS (ESI) [M + H]^+^ calc. 424.1906 for [C_28_H_26_NO_3_]^(+)^ found 424.1908. IR ν(cm^–1^): 2965, 2926, 2873, 2227, 1715, 1686, 1492, 1391, 1368, 1220, 1024, 756, 661. Mp 80–83 °C. [α]20D −76.02 (*c* = 1.00, CHCl_3_).

#### Benzyl (*S*)-5,7-Dimethyl-4-oxo-2-(*m*-tolylethynyl)-3,4-dihydroquinoline-1(2*H*)-carboxylate (**3v**)^17^

Synthesized as per general procedure C using benzyl 5,7-dimethyl-4-oxoquinoline-1(4*H*)-carboxylate **1g** and 3-methylphenylacetylene. The product was isolated as a white solid (40 mg, 48%, 84% ee). ^1^H NMR (400 MHz, CDCl_3_) δ 7.45–7.30 (m, 6H), 7.13–7.04 (m, 2H), 7.04–6.95 (m, 2H), 6.85 (s, 1H), 5.98 (dd, *J* = 5.9, 1.8 Hz, 1H), 5.39 (d, *J* = 12.3 Hz, 1H), 5.22 (d, *J* = 12.3 Hz, 1H), 3.11 (dd, *J* = 17.4, 6.0 Hz, 1H), 2.89 (dd, *J* = 17.4, 1.8 Hz, 1H), 2.64 (s, 3H), 2.32 (s, 3H), 2.25 (s, 2H). ^13^C{^1^H} NMR (101 MHz, CDCl_3_) δ 193.6, 153.3, 143.8, 142.1, 141.6, 138.0, 135.8, 132.5, 130.2, 129.6, 129.0, 128.8, 128.6, 128.3, 128.2, 123.9, 122.1, 122.0, 85.7, 84.8, 68.6, 46.9, 46.3, 23.3, 21.9, 21.3. [α]^20^_D_ −134.78 (*c* = 1.00, CHCl_3_).

#### Benzyl (*S*)-8-Oxo-6-(*m*-tolylethynyl)-7,8-dihydro-[1,3]dioxolo[4,5-*g*]quinoline-5(6*H*)-carboxylate (**3w**)^17^

Synthesized as per general procedure C using benzyl 8-oxo-[1,3]dioxolo[4,5-*g*]quinoline-5(8*H*)-carboxylate **1h** and 3-methylphenylacetylene. The product was isolated as a white solid (13 mg, 15%, 28% ee). ^1^H NMR (400 MHz, CDCl_3_) δ 7.45–7.28 (m, 7H), 7.16–7.01 (m, 4H), 6.06–5.99 (m, 3H), 5.36 (d, *J* = 12.3 Hz, 1H), 5.28 (d, *J* = 12.2 Hz, 1H), 3.06 (dd, *J* = 17.1, 5.6 Hz, 1H), 2.87 (dd, *J* = 17.1, 2.0 Hz, 1H), 2.26 (s, 3H). ^13^C{^1^H} NMR (101 MHz, CDCl_3_) δ 190.3, 152.9, 152.8, 145.2, 138.1, 137.9, 135.4, 132.4, 129.5, 128.9, 128.8, 128.6, 128.3, 128.1, 121.6, 119.8, 105.1, 105.0, 102.1, 85.0, 84.7, 68.6, 47.7, 44.0, 21.3. [α]^20^_D_ −100.90 (*c* = 1.00, CHCl_3_).

#### (+)-Cuspareine (**4**)^6^

Step 1: Synthesized as per general procedure **C** on a 1 mmol scale using benzyl 4-oxoquinoline-1(4*H*)-carboxylate **1a** and 4-ethynyl-1,2-dimethoxybenzene. The product was isolated as a yellow solid (0.254 g, 56%, 77% ee). This product was then recrystallized using hot ethanol (94% ee). Step 2: Pd/C 10% (0.1064 g, 0.1 mmol 0.5 equiv) was added to a flame-dried Schlenk flask, followed by a solution of **3i** (0.0883 g, 0.2 mmol) in MeOH (5 mL) and TFA (0.2 mL). The tube was put under a hydrogen atmosphere (1 atm). The reaction was stirred at room temperature for 16 h. The reaction was filtered through Celite and the filtrate evaporated to remove the solvent. The colorless liquid was carried forward to the next step without further purification. Step 3: The above crude product was dissolved in THF (5 mL) and K_2_CO_3_ (0.0829 g, 0.6 mmol, 3 equiv), and the reaction was stirred for 10 min. To this was added MeI (0.1419 g, 1.0 mmol, 5 equiv). The reaction was heated to reflux and stirred for 16 h. The reaction was quenched with water (5 mL) and extracted with CH_2_Cl_2_ (3 × 5 mL). The combined organic layers were washed with brine (5 mL), dried with MgSO_4_, and concentrated. Purification by column chromatography (c-Hex–EtOAc 20:1) afforded the product as a colorless oil (0.0053 g, 0.02 mmol, 17%). ^1^H NMR (400 MHz, CDCl_3_) δ 7.08 (t, *J* = 7.9, 6.8 Hz, 1H), 6.98 (d, *J* = 7.5 Hz, 1H), 6.79 (d, *J* = 8.0 Hz, 1H), 6.75–6.69 (m, 2H), 6.59 (t, *J* = 7.3 Hz, 1H), 6.53 (d, *J* = 8.2 Hz, 1H), 3.87 (s, 3H), 3.86 (s, 3H), 2.92 (s, 3H), 2.87–2.79 (m, 1H), 2.74–2.63 (m, 2H), 2.58–2.48 (m, 1H), 1.98–1.87 (m, 3H), 1.77–1.68 (m, 1H). ^13^C{^1^H} NMR (101 MHz, CDCl_3_) δ 149.0, 147.4, 145.4, 134.8, 131.0, 128.8, 127.3, 121.9, 120.2, 115.5, 111.7, 111.4, 110.8, 58.6, 56.1, 56.0, 38.3, 33.2, 32.1, 24.5, 23.7. [α]^20^_D_ +26.95 (*c* = 1.00, CHCl_3_).

#### (+)-Galipinine (**5**)^6^

Step 1: Pd/C 10% (0.1064 g, 0.1 mmol 0.5 equiv) was added to a flame-dried Schlenk flask, followed by a solution of 3j (0.0883 g, 0.2 mmol) in MeOH (5 mL) and TFA (0.2 mL). The tube was put under a hydrogen atmosphere (1 atm). The reaction was stirred at room temperature for 16 h. The reaction was filtered through Celite and the filtrate evaporated to remove the solvent. The colorless liquid was carried forward to the next step without further purification. Step 2: The above crude product was dissolved in THF (5 mL) and K_2_CO_3_ (0.0829 g, 0.6 mmol, 3 equiv), and the reaction was stirred for 10 min. To this was added MeI (0.1419 g, 1.0 mmol, 5 equiv). The reaction was heated to reflux and stirred for 16 h. The reaction was quenched with water (5 mL) and extracted with CH_2_Cl_2_ (3 × 5 mL). The combined organic layers were washed with brine (5 mL), dried with MgSO_4_, and concentrated. Purification by column chromatography (c-Hex–EtOAc 20:1) afforded the product as a pale yellow oil. (0.010 g, 16%). ^1^H NMR (400 MHz, CDCl_3_) δ 7.07 (t, *J* = 7.8 Hz, 1H), 6.97 (d, *J* = 7.3 Hz, 1H), 6.72 (d, *J* = 7.9 Hz, 1H), 6.68 (d, *J* = 1.7 Hz, 1H), 6.63 (dd, *J* = 7.9, 1.7 Hz, 1H), 6.59 (td, *J* = 7.3, 1.1 Hz, 1H), 6.54–6.51 (m, 1H), 5.92 (s, 2H), 2.90 (s, 3H), 2.89–2.78 (m, 1H), 2.72–2.60 (m, 2H), 2.54–2.46 (m, 1H), 1.98–1.83 (m, 3H), 1.74–1.65 (m, 2H). ^13^C{^1^H} NMR (101 MHz, CDCl_3_) δ 147.8, 145.8, 145.5, 136.0, 128.8, 127.3, 121.9, 121.1, 115.6, 110.8, 108.9, 108.3, 100.9, 58.4, 38.2, 33.3, 32.2, 24.5, 23.7. [α]^20^_D_ +18.08 (*c* = 1.00, CHCl_3_).

#### Benzyl (2*S*,3*S*)-3-Methyl-4-oxo-2-(m-tolylethynyl)-3,4-dihydroquinoline-1(2*H*)-carboxylate (**8**)

Synthesized as per general procedure C using Ethyl 3-methyl-4-oxoquinoline-1(4*H*)-carboxylate **7** and 3-methylphenylacetylene. The product was isolated as a pale yellow oil (13 mg, 18%, 25% ee). ^1^H NMR (400 MHz, CDCl_3_) δ 8.04 (dd, *J* = 7.8, 1.7 Hz, 1H), 7.86 (d, *J* = 8.4 Hz, 1H), 7.53 (ddd, 8.4, 7.8, 1.7 Hz), 7.22–7.16 (m, 1H), 7.11–7.03 (m, 2H), 7.01–6.96 (m, 2H), 5.85 (d, *J* = 4.9 Hz, 1H), 4.37 (qq, *J* = 10.7, 7.0 Hz, 2H), 3.08 (qd, *J* = 6.8, 4.9 Hz, 1H), 2.24 (s, 3H), 1.42 (d, *J* = 6.8 Hz, 3H), 1.40 (t, *J* = 7.1 Hz, 3H). ^13^C{^1^H} NMR (101 MHz, CDCl_3_) δ 195.0, 153.4, 141.5, 138.0, 134.2, 132.5, 129.6, 129.1, 128.2, 127.3, 124.6, 124.4, 124.0, 121.8, 86.3, 83.4, 63.2, 53.1, 47.0, 21.2, 14.7, 11.8. HRMS (ESI) [M + H]^+^ calcd 348.1593 for [C_22_H_22_NO_3_]^(+)^ found 348.1594. IR ν (cm^–1^): 3065, 3032, 2987, 2956, 2926, 1716, 1691, 1478, 1456, 1262, 1217, 1005, 819, 758, 694. [α]^20^_D_ +29.05 (*c* = 1.00, CHCl_3_).

## Data Availability

The data underlying this study are available in the published article and its Supporting Information.

## Supporting Information Available

Full experimental procedures, characterization data, NMR spectra, and chiral HPLC/SFC traces for all novel compounds reported (PDF)
