# Supplementary material for: Enantioselective Copper-Catalyzed Alkynylation of Quinolones Using Chiral P,N Ligands
Source: J Org Chem. 2023 Nov 15;89(3):1993–2000. doi: 10.1021/acs.joc.3c01944 (PMC10845117; doi:10.1021/acs.joc.3c01944)

Enantioselective Copper Catalysed Alkynylation of Quinolones Using P,N Ligands

Dáiríne M. Morgan, Cian M. Reid, Patrick J. Guiry*

Centre for Synthesis and Chemical Biology, School of Chemistry, University College Dublin, Belfield, Dublin 4, Ireland.

Supporting Information

Table of Contents

1. General Experimental Considerations........ S2
2. Optimisation of enantioselective reaction conditions........ S3
3. Synthesis and characterisation of quinolones. S6
4. Synthesis and characterisation of alkynes........ S12
5. Chromatograms of 4-oxo-2-alkynyl dihydroquinolines........ S14
6. References........ S45
7. NMR Spectra........ S47

1. General Experimental Considerations

1H-NMR Spectroscopy: ^{1}H and $^{13C\{1H\}}$ NMR spectra were obtained using Varian VNMRS 400, 500 and 600 MHz spectrometers at room temperature. Proton and carbon chemical shifts are quoted in ppm. ^{1}H NMR spectra were recorded using an internal deuterium lock for the residual protons in $CDCl_3$ (δ 7.26). $^{13C\{1H\}}$ NMR spectra were recorded using an internal deuterium lock in $CDCl_3$ (δ 77.0). Assignments were determined either on the basis of unambiguous chemical shift or coupling patterns, COSY, HSQC and/or NOESY experiments. Peak multiplicities are defined as: s = singlet, d = doublet, t = triplet, q = quartet, m = multiplet, br. = broad; coupling constants (J) are reported to the nearest 0.1 Hz.

Infrared Spectroscopy: Infrared spectra were recorded on a Varian 3100 FT-IR spectrometer with the sample being prepared as a thin film on a diamond ATR module. Absorption maxima ($\nu_{max)}$ are quoted in wavenumbers (cm-^{1}).

Ultra High Performance Liquid Chromatrography: UHPLC was performed on an Shizmadzu UHPLC using a Chiralcel-OD, AD, or OJ column.

Mass Spectrometry: High-resolution mass spectra (HRMS) were recorded using a Waters Micromass LCT time-of-flight mass spectrometer.

Optical Rotation: Optical rotation measurements were recorded using a Schmidt-Haensch Unipol L2000 polarimeter at 589 nm and are quoted in units of deg cm^3 dm^{-1} g^{-1}.

Reagents, Solvents and Techniques: Reagents were purchased from Sigma-Aldrich, Fischer, Acros or Fluorochem and used without further purification unless otherwise state. Dry tetrahydrofuran obtained from a Puresol Grubbs system unless otherwise stated. Toluene was dried over 3 Å moleclualr sieves. When appropriate, reactions were performed under a nitrogen atmosphere with oven dried glassware. Oxygen free nitrogen was supplied by BOC gases and used without further drying. Column chromatography was performed with Merck Kieselgel 60 F254 (230-400 mesh) silica gel. Thin-layer chromatography was performed on aluminium sheets pre-coated plates with silica gel 60 F254, or aluminium oxide 60 F254. The plates were realised with ultraviolet fluorescence. Solvent was removed from solutions using a Büchi rotary evaporator with an integrated vacuum pump.

2. Optimisation of enantioselective reaction conditions

In all cases for the optimisation general procedure **C** was used (*vide infra*).

Optimisation of reaction ligand

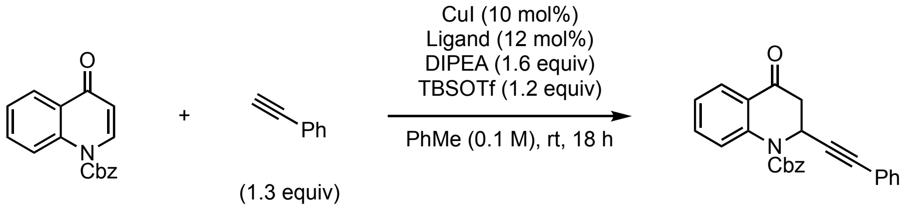

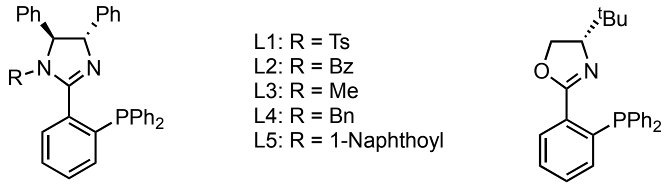

Table S1: Initial Ligand Screen

Entry	Ligand	Yield (%)	*ee* (%)
**1**	L1	57	4
**2**	L2	49	15
**3**	L3	54	0
**4**	L4	65	34
**5**	L5	69	17
**6**	(*S*)-*t*Bu-PHOX	30	7

Optimisation of copper salt

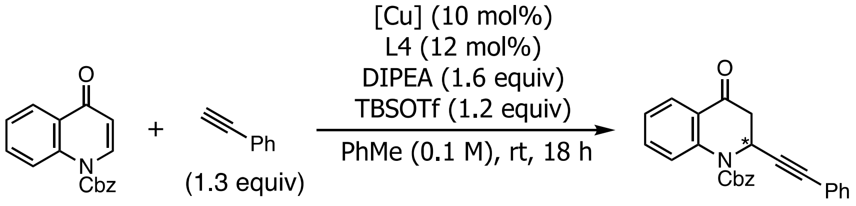

Table S2: Copper Salt Screen

Entry	Copper Salt	Yield (%)	*ee* (%)
**1**	CuI	65	34
**2**	CuBr	35	21
**3**	CuCl	17	0
**4**	CuOAc	21	0

**5**	$Cu(MeCN)_4BF_4$	21	5
**6**	Cu(OTf)	9	0
**7**	$Cu(OTf)_2$	16	17

Optimisation of activator, solvent, and base

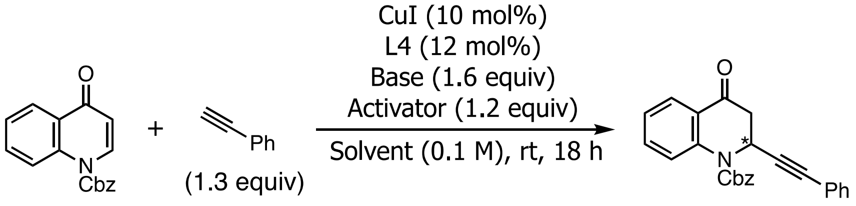

Table S3: Activator, Solvent and Base Screen

Entry	Activator	Solvent	Base	Yield (%)	*ee* (%)
**1**	TBSOTf	PhMe	DIPEA	65	34
**2**	TMSOTf	PhMe	DIPEA	90	11
**3**	TBSOTf	THF	DIPEA	52	14
**4**	TBSOTf	Methanol	DIPEA	0	n/a
**5**	TBSOTf	Acetonitrile	DIPEA	9	0
**6**	TBSOTf	PhMe	TEA	Trace	n/a
**7**	TBSOTf	PhMe	Cs_2CO_3	0	n/a
**8**	TBSOTf	PhMe	Cy_2NMe	90	26
**9**	TBSOTf	PhMe	KOAc	0	n/a
**10**	TBSOTf	PhMe	DBU	0	n/a

Optimisation of reaction temperature

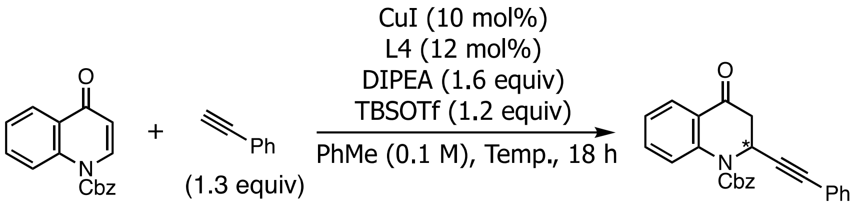

Table S4: Temperature Screen

Entry	Temp. (°C)	Yield (%)	*ee* (%)
**1**	rt	65	*34*
**2**	0	47	50
**3**	-20	32	54
**4**	-40	18	65
**5**[a]	-20	58	74

[a]Using (*S*,*S*,*R*$_a$)-UCD-Phim as a ligand

Optimisation of reagents and reactant equivalents

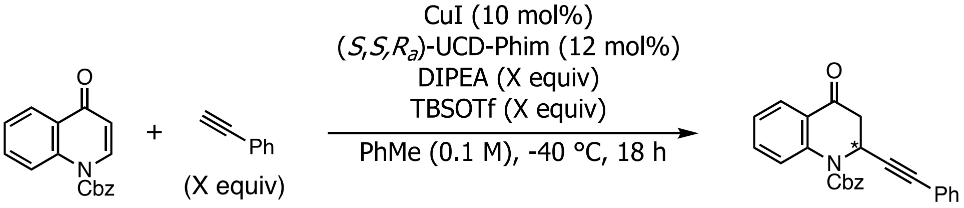

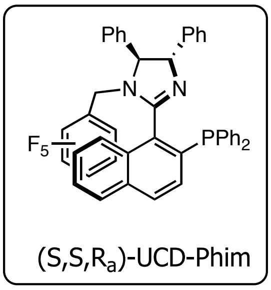

Table S5: Reagents' and Reactants' Equivalents Screen[a]

Entry	TBSOTf Equiv	Alkyne Equiv	Base Equiv	Yield (%)	*ee* (%)
**1**	1.2	1.3	1.6	58	74
**2**	1	1.3	1.6	34	75
**3**	1.4	1.3	1.6	49	78
**4**	1.2	1	1.6	24	80
**5**	1.2	1.3	1	42	78

[a]TBSOTf added to reaction at -78 °C

Optimisation of reaction concentration and reaction time

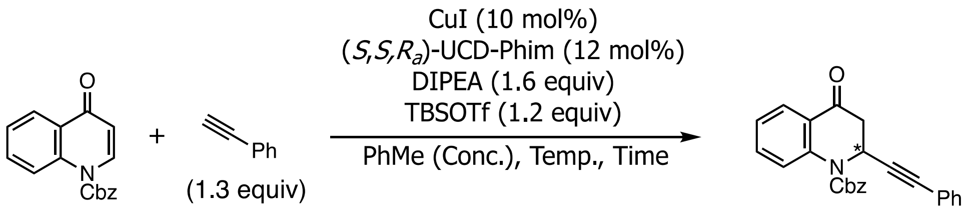

Table S6: Concentration and Time Screen

Entry	Conc. (M)	Time (h)	Temp. (°C)	Yield (%)	*ee* (%)
**1**	0.1	18	-20	58	74
**2**	0.2	18	-20	35	56
**3**	0.05	18	-20	35	63
**4**	0.1	24	-20	59	60
**5**	0.1	48	-20	54	63
**6**[a]	0.1	18	-20	70	75
**7**[a]	**0.1**	**18**	**-40**	**60**	**84**

[a]TBSOTf added to reaction at -78 °C

3. Synthesis and characterisation of quinolones.

General Procedure (B) for synthesis of protected 4(H)-quinolones. *Step 1:* A flame-dried, nitrogen-backfilled Schlenk flask was charged with a stir bar and Meldrum's acid (1.5 equiv). Trimethyl orthoformate (0.25 M) was added and the reaction was heated to 115 °C and stirred for 2 h using a heating mantle. The reaction was cooled to room temperature and the corresponding aniline was added (1 equiv). The reaction was heated to 115 °C for 2 h. The reaction was cooled to room temperature and the solvent was rotevaped., the solid was washed with MeOH (10 mL) and filtered. Without further purification, the crude product was added to a round bottom flask with a stir bar. Diphenyl ether (0.44 M) was added, and the reaction was heated at 250 °C for 1.5 h using a heating mantle. The solution was cooled to room temperature and cyclohexane (100 mL) was added. The precipitated product was filtered and washed with cyclohexane (50 mL) and the solid dried and subjected to the next reaction without further purification.

Step 2: The crude 4(H)-quinolones were taken forward to the next step without further purification. A flame-dried round bottom flask was charged with a stir bar, evacuated, and backfilled with nitrogen. Sodium hydride (2 equiv, 60 wt% in mineral oil) was added with tetrahydrofuran (1/2, total molarity: 0.081 M). The 4(H)-quinolones (1 equiv) in tetrahydrofuran (1/2, total molarity: 0.081 M) was added. The reaction was heated to 55 °C in an oil bath and stirred for 15 minutes. The reaction was cooled to room temperature and the acyl chloride (1 equiv) was added. The reaction was allowed stir at room temperature for 16 h. The reaction was cooled to 0 °C and quenched using water (10 mL). The organics were removed *in vacuo*. The aqueous phase was extracted using ethyl acetate (3 x 10 mL) and the combined organic phases were dried over sodium sulfate, filtered and concentrated *in vacuo* to afford the crude product. Purification was *via* flash column chromatography (c-Hex - EtOAc 5:1 to c-Hex – EtOAc 1:1) to afford the desired quinolone.

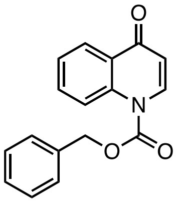

Benzyl 4-oxoquinoline-1(4H)-carboxylate (1a).[1] Synthesised as per general procedure **B** using aniline and benzyl chloroformate protecting group. The product was isolated as a yellow solid (0.96 g, 32 % yield). $\mathbf{R_f}$ = 0.5 (c-Hex – EtOAc 1:1).

^{1}H NMR (400 MHz, $CDCl_3$) δ 8.70 – 8.66 (m, 1H), 8.37 (dd, J = 8.3, 1.7 Hz, 2H), 7.67 (ddd, J = 8.9, 7.1, 1.8 Hz, 1H), 7.52 – 7.37 (m, 7H), 6.26 (d, J = 8.6 Hz, 1H), 5.47 (s, 2H).

^{13}C{^{1}H} NMR (101 MHz, $CDCl_3$) δ 179.1, 151.5, 138.6, 138.4, 134.1, 133.1, 129.5, 129.2, 129.0, 126.7, 125.7, 120.1, 112.7, 70.7.

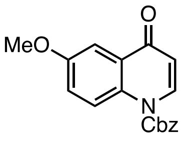

Benzyl 6-methoxy-4-oxoquinoline-1(4H)-carboxylate (1b).[1] Synthesised as per general procedure **B** using 4-methoxyaniline and benzyl chloroformate protecting group. The product was isolated as a yellow solid (0.127 g, 18%). $\mathbf{R_f}$ = 0.3 (c-Hex – EtOAc 1:1).

^{1}H NMR (500 MHz, $CDCl_3$) δ 8.63 (d, J = 9.6 Hz, 1H), 8.35 (d, J = 8.5 Hz, 1H), 7.76 (d, J = 3.2 Hz, 1H), 7.50 – 7.37 (m, 5H), 7.30 – 7.22 (m, 1H), 6.24 (d, J = 8.5 Hz, 1H), 5.45 (s, 2H), 3.90 (s, 3H).

$^{13}C\{^1H\}$ NMR (101 MHz, $CDCl_3$) δ 178.78, 157.18, 151.4, 137.8, 134.1, 132.8, 129.4, 129.1, 129.0, 128.1, 122.7, 121.9, 111.8, 106.3, 70.6, 55.8.

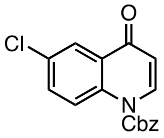

Benzyl 6-chloro-4-oxoquinoline-1(4H)-carboxylate (1c).[2] Synthesised as per general procedure **B** using 4-chlorolaniline and benzyl chloroformate protecting group. The product was isolated as a yellow solid (0.793 g, 55%). **R_f** = 0.54 (c-Hex – EtOAc 1:1).

^1H NMR (400 MHz, $CDCl_3$) δ 8.67 (d, J = 9.4 Hz, 1H), 8.36 (d, J = 8.6 Hz, 1H), 8.32 (d, J = 2.7 Hz, 1H), 7.59 (dd, J = 9.4, 2.7 Hz, 1H), 7.52 – 7.38 (m, 5H), 6.25 (d, J = 8.6 Hz, 1H), 5.47 (s, 2H).

$^{13}C\{^1H\}$ NMR (101 MHz, $CDCl_3$) δ 177.8, 151.2, 138.5, 137.0, 133.9, 133.1, 131.9, 129.5, 129.1, 129.1, 128.7, 127.9, 127.1, 126.1, 122.1, 112.7, 70.9.

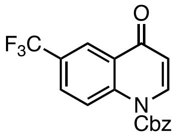

Benzyl 4-oxo-6-(trifluoromethyl)quinoline-1(4H)-carboxylate (1d).[1] Synthesised as per general procedure **B** using 4-Trifluoromethyllaniline and benzyl chloroformate protecting group. The product was isolated as a yellow solid (0.249 g, 15%). **R_f** = 0.45 (c-Hex – EtOAc 1:1).

^1H NMR (400 MHz, $CDCl_3$) δ 8.83 (d, J = 9.2 Hz, 1H), 8.66 (s, 1H), 8.40 (d, J = 8.6 Hz, 1H), 7.87 (dd, J = 9.2, 2.4 Hz, 1H), 7.51 – 7.29 (m, 5H), 6.31 (d, J = 8.6 Hz, 1H), 5.49 (s, 2H).

$^{13}C\{^1H\}$ NMR (101 MHz, $CDCl_3$) δ 178.0, 151.2, 140.6, 138.9, 133.7, 129.7, 129.2, 129.2, 129.2, 128.7, 127.8, 127.1, 126.6, 124.6, 121.2, 113.2, 71.2.

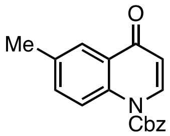

Benzyl 6-methyl-4-oxoquinoline-1(4H)-carboxylate (1e).[1] Synthesised as per general procedure **B** using 4-methylaniline and benzyl chloroformate protecting group. The product was isolated as a yellow solid (0.348 g, 26%). **R_f** = 0.27 (c-Hex – EtOAc 1:1).

^1H NMR (400 MHz, $CDCl_3$) δ 8.56 (d, J = 8.9 Hz, 1H), 8.34 (d, J = 8.5 Hz, 1H), 8.15 (d, J = 2.3 Hz, 1H), 7.52 – 7.32 (m, 6H), 6.20 (m, 1H), 5.45 (s, 2H), 2.44 (s, 3H).

$^{13}C\{^1H\}$ **NMR** (101 MHz, $CDCl_3$) δ 179.2, 151.4, 138.1, 136.5, 135.6, 134.3, 134.1, 129.4, 129.1, 128.9, 126.5, 126.2, 112.0, 112.5, 70.5, 20.8.

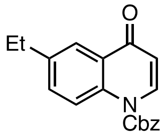

Benzyl 6-ethyl-4-oxoquinoline-1(4H)-carboxylate (1f). Synthesised as per general procedure **B** using 4-ethylaniline and benzyl chloroformate protecting group. The product was isolated as a yellow solid (0.224 g, 16%). $\mathbf{R_f}$ = 0.45 (c-Hex – EtOAc 1:1).

^{1}**H NMR** (400 MHz, $CDCl_3$) δ 8.59 (d, J = 9.0 Hz, 1H), 8.35 (d, J = 8.5 Hz, 1H), 8.21 – 8.16 (m, 1H), 7.53 – 7.39 (m, 5H), 6.24 (d, J = 8.5 Hz, 1H), 5.46 (s, 2H), 2.76 (q, J = 7.6 Hz, 2H), 1.28 (t, J = 7.6 Hz, 3H).

$^{13}C\{^1H\}$ **NMR** (101 MHz, $CDCl_3$) δ 179.3, 151.5, 141.9, 138.1, 136.7, 134.1, 133.3, 129.4, 129.1, 129.0, 126.7, 125.0, 120.1, 112.5, 70.6, 28.3, 15.4.

HRMS (ESI) $[M+H]^+$ calc. 308.1280 for $[C_{19}H_{18}NO_3]^{(+)}$ found 308.1281.

IR ν(cm^{-1}): 2961, 2929, 2871, 1747, 1639, 1603, 1483, 1193, 1012, 821, 751, 698.

MP 63-65 °C.

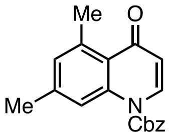

Benzyl 5,7-dimethyl-4-oxoquinoline-1(4H)-carboxylate (1g).[1] Synthesised as per general procedure **B** using 3,5-dimethylaniline and benzyl chloroformate protecting group. The product was isolated as a yellow oil (0.560 g, 42%). $\mathbf{R_f}$ = 0.65 (c-Hex – EtOAc 1:1).

^{1}**H NMR** (400 MHz, $CDCl_3$) δ 8.21 – 8.12 (m, 2H), 7.51 – 7.32 (m, 5H), 7.00 (dt, J = 1.6, 0.8 Hz, 1H), 6.11 (d, J = 8.5 Hz, 1H), 5.44 (s, 2H), 2.83 (s, 3H), 2.40 (s, 3H).

$^{13}C\{^1H\}$ **NMR** (101 MHz, $CDCl_3$) δ 181.3, 151.9, 142.3, 141.1, 140.3, 136.4, 134.3, 130.3, 129.3, 129.1, 128.9, 123.3, 118.3, 114.3, 70.4, 24.0, 22.1.

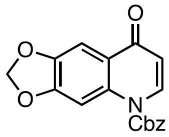

Benzyl 8-oxo-[1,3]dioxolo[4,5-g]quinoline-5(8H)-carboxylate (1h).[1] Synthesised as per general procedure **B** using 3,4-(Methylenedioxy)aniline and benzyl chloroformate protecting group. The product was isolated as a yellow solid (0.110 g, 6%). $\mathbf{R_f}$ = 0.30 (c-Hex – EtOAc 1:1).

^{1}H NMR (400 MHz, $CDCl_3$) δ 8.28 (d, J = 8.5 Hz, 1H), 8.22 (s, 1H), 7.72 (s, 1H), 7.52 – 7.38 (m, 5H), 6.20 (d, J = 8.5 Hz, 1H), 6.09 (s, 2H), 5.45 (s, 2H).

$^{13}C\{^1H\}$ NMR (101 MHz, $CDCl_3$) δ 177.9, 152.5, 151.5, 146.3, 137.3, 135.4, 134.0, 129.5, 129.1, 129.0, 122.9, 112.2, 104.0, 102.4, 100.3, 70.7.

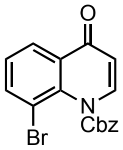

Benzyl 8-bromo-4-oxoquinoline-1(4H)-carboxylate (1i). Synthesised as per general procedure **B** using 2-bromoaniline and benzyl chloroformate protecting group. The product was isolated as a yellow solid (0.19 g, 12%). **R_f** = 0.32 (c-Hex – EtOAc 1:1).

^{1}H NMR (400 MHz, $CDCl_3$) δ 9.06 (d, J = 5.0 Hz, 1H), 8.07 (ddd, J = 20.4, 8.0, 1.3 Hz, 2H), 7.56 (d, J = 5.0 Hz, 1H), 7.49 – 7.35 (m, 6H), 5.36 (s, 2H).

$^{13}C\{^1H\}$ NMR (101 MHz, $CDCl_3$) δ 154.7, 152.0, 146.8, 141.0, 134.3, 129.3, 129.0, 128.9, 128.7, 127.8, 124.8, 121.3, 112.4, 71.4.

IR ν(cm^{-1}): 3139, 3063, 3040, 2960, 2925, 1755, 1725, 1595, 1490, 1378, 1218, 1204, 757, 700.

HRMS (ESI) $[M+H]^+$ calc. for 223.9711 $[C_9H_8BrNO]^{(+)}$ found 223.9705. The mass of **1i** could not be identified from using HRMS instead, the protonated fragment that occurred after loss of the Cbz group was identified.

MP 103-106 °C.

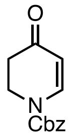

Benzyl 4-oxo-3,4-dihydropyridine-1(2H)-carboxylate (6).[3] A solution of 4-methoxypyridine (0.47 mL, 4.58 mmol, 1 eq.) was added to methanol (9.4 mL). The reaction was cooled to -78 °C. Sodium borohydride (0.19 g, 5.04 mmol, 1.1 eq.) was added and the reaction was stirred for 15 min. Benzyl chloroformate (0.72 mL, 5.04 mmol, 1.1 eq.) was added dropwise over 20 min. The reaction was stirred for 1 h. The reaction was warmed to -60 °C and quenched with water (10 mL). The reaction was warmed to room temperature. The aqueous phase was extracted using ethyl acetate (3 x 20 mL) and the combined organic phases were washed with water (2 x 20 mL) and brine (20 mL). The organic phase was dried using magnesium sulfate. The organic phase was gravity filtered and was concentrated under vacuum to afford the crude product a yellow oil. Purification by column chromatography (c-Hex - EtOAc 4:1 to c-Hex – EtOAc 1:1) afforded the product a white solid **R_f** = 0.17 (c-Hex – EtOAc 4:1). (0.63 g, 59% yield).

^{1}H NMR (400 MHz, $CDCl_3$) δ 7.85 (br s, 1H), 7.43 – 7.32 (m, 5H), 5.34 (d, J = 8.3 Hz, 1H), 5.26 (s, 2H), 4.08 – 4.01 (m, 2H), 2.59 – 2.51 (m, 2H).

^{13}C{^{1}H} NMR (101 MHz, $CDCl_3$) δ 193.4, 143.4, 135.0, 128.9, 128.8, 128.6, 107.9, 69.2, 42.7, 35.8.

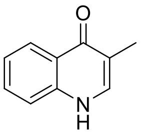

3-methylquinolin-4(1H)-one.[4] *Step 1:* 2-aminobenzonitrile (0.5 g, 4.23 mmol, 1 eq.) was dissolved in THF (14 mL, 0.3 M). The reaction was cooled to 0 °C. Ethylmagnesium bromide (3.0 M in diethyl ether) (4.23 mL, 12.69 mmol, 3 eq.) was added dropwise to the reaction mixture, formation of bright yellow precipitate observed. The reaction was left stirring at room temperature for 6 h. The reaction was quenched by addition of 2 M HCl, formation of white precipitate. 2M NaOH was added to basify the reaction. The aqueous phase was extracted with diethyl ether (3 x 20 mL). The organic phase was dried using magnesium sulfate. The organic phase was gravity filtered and was concentrated under vacuum to afford the crude product a yellow oil. The crude product was taken forward to the following step without further purification.

Step 2: 1-(2-aminophenyl)propan-1-one (0.32 g, 2.15 mmol, 1 eq.) was added to methylformate (5.96 mL, 0.36 M). NaH (60% in mineral oil) (0.18 g, 4.53 mmol, 2.11 eq.) was added and the reaction was heated to 40 °C. The reaction was left stir overnight. The reaction was cooled to 0 °C followed by the addition of water (10 mL) and ethanol (10 mL). the aqueous layer was washed ethyl acetate (3 x 25 mL). The organic phase was dried using magnesium sulfate. The organic phase was gravity filtered and was concentrated under vacuum to afford the crude product a yellow oil. Purification by column chromatography (c-Hex - EtOAc 1:1 to c-Hex – EtOAc 1:3) afforded the product a white solid (0.26 g, 39% yield over 2 steps).

^{1}H NMR (400 MHz, DMSO-D_6) δ 11.61 (s, 1H), 8.14 – 8.06 (m, 1H), 7.87 (dq, J = 5.9, 0.9 Hz, 1H), 7.59 (ddd, J = 8.4, 6.9, 1.5 Hz, 1H), 7.49 (ddd, J = 8.4, 1.2, 0.7 Hz, 1H), 7.27 (ddd, J = 8.1, 6.9, 1.2 Hz, 1H), 1.98 (d, J = 1.0 Hz, 3H).

^{13}C{^{1}H} NMR (101 MHz, DMSO-D_6) δ 176.7, 139.8, 136.7, 131.0, 124.9, 124.09, 122.5, 118.0, 116.5, 13.5.

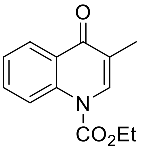

Ethyl 3-methyl-4-oxoquinoline-1(4H)-carboxylate (7). Synthesised as per general procedure **B** using 3-methylquinolin-4(1H)-one and ethyl chloroformate protecting group (1.63 mmol scale). The product was isolated as a yellow solid (0.135 g, 31%).

^{1}H NMR (400 MHz, $CDCl_3$) δ 8.63 (ddd, J = 8.9, 1.0, 0.5 Hz, 1H), 8.39 (ddd, J = 8.0, 1.8, 0.5 Hz, 1H), 8.29 (q, J = 1.1 Hz, 1H), 7.63 (ddd, J = 8.9, 7.0, 1.8 Hz, 1H), 7.41 (ddd, J = 8.0, 7.1, 1.0 Hz, 1H), 4.52 (q, J = 7.1 Hz, 2H), 2.12 (d, 3H), 1.49 (t, J = 7.1 Hz, 3H).

^{13}C{^{1}H} NMR (101 MHz, $CDCl_3$) δ 179.5, 151.8, 138.5, 135.3, 132.5, 126.7, 125.5, 125.2, 120.4, 119.9, 65.0, 14.4, 14.0.

4. Synthesis and characterisation of alkynes

General Procedure (A) for synthesis of aryl alkynes: *Step 1:* A flame-dried, nitrogen-backfilled Schlenk tube was charged with a stir bar, $Pd(PPh_3)_4$ (5 mol%), CuI (10 mol%) and the aryl bromide (1 equiv) if it was a solid. THF (1.0 M) and triethylamine (1.0 M) were added to the reaction mixture. TMS-acetylene (1.2 equiv) was added to the reaction mixture with the Schlenk tap closed. The aryl bromide (1 equiv) was then added if it was a liquid. The reaction mixture was then heated to 70 °C and stirred for 14 hours. The reaction was allowed to cool to room temperature and then passed through a plug of silica using pentane as an eluent. The organics were concentrated *in vacuo* The crude residue was subjected to the next reaction without further purification.

Step 2: The crude residue is dissolved in dry methanol (0.5 M) and transferred to a flame-dried, nitrogen-backfilled flask containing potassium carbonate (4.0 equiv). The reaction mixture was stirred at room temperature for 3 hours. The reaction was diluted with water (50 mL) and extracted with EtOAc (3x50 mL). The organic layer was washed with brine and dried over sodium sulfate. The organics were concentrated *in vacuo* and the crude residue was subjected to flash column chromatography (c-hex/EtOAc) to afford the desired aryl alkyne.

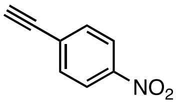

1-ethynyl-4-nitrobenzene (2h).[5] Synthesised as per general procedure **A** from 1-bromo-4-nitrobenzene. The product was isolated as a yellow solid (0.0895 g, 0.61 mmol, 78%). **R$_f$** = 0.5 (c-Hex – EtOAc 4:1).

^{1}H NMR (400 MHz, $CDCl_3$) δ 8.18 (dt, J = 8.9, 2.2 Hz, 1H), 7.63 (dt, J = 8.9, 2.2 Hz), 3.34 (s, 1H).

^{13}C{^{1}H} NMR (101 MHz, $CDCl_3$) δ 147.7, 133.1, 129.1, 123.7, 82.5, 81.8.

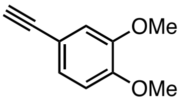

4-ethynyl-1,2-dimethoxybenzene (2i).[6] Synthesised as per general procedure **A** from 4-bromo-1,2-dimethoxybenzene. The product was isolated as a yellow solid (0.5500 g, 3.39 mmol, 90%). **R$_f$** = 0.4 (c-Hex – EtOAc 4:1).

^{1}H NMR (400 MHz, $CDCl_3$) δ 7.08 (dd, J = 8.3, 1.8 Hz, 1H), 6.97 (d, J = 1.8 Hz, 1 H), 6.78 (d, J = 8.3, 1H). 3.86 (s, 3H), 3.85 (s, 3H), 2.98 (s, 1H)

^{13}C{^{1}H} NMR (101 MHz, $CDCl_3$) δ 149.8, 148.6, 125.5, 114.7, 114.2, 111.0, 83.7, 75.6, 55.9 (2 x OCH_3).

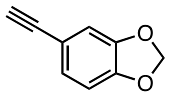

3,4-methylenedioxyphenylethyne (2j).[7] Synthesised as per general procedure **A** from 1,2-(methylenedioxy)-4-bromobenzene. The product was isolated as a yellow oil **R_f** = 0.76 (0.4010 g, 2.74 mmol, 68%).

^{1}H NMR (400 MHz, $CDCl_3$) δ 7.02 (101 MHz, $CDCl_3$) δ 7.02 (dd, J = 8.0, 1.6 Hz, 1H), 6.93 (d, J = 1.6 Hz, 1H), 6.75 (dd, J = 8.0, 0.5 Hz, 1H), 5.97 (d, J = 0.5 Hz, 1H), 2.97 (s, 1H).

^{13}C{^{1}H} NMR (101 MHz, $CDCl_3$) δ 148.4, 147.5, 127.0, 115.4, 112.2, 108.6, 101.5, 83.7, 75.7.

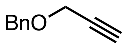

((prop-2-yn-1-yloxy)methyl)benzene (2o).[8] Propragyl alcohol (0.21 mL, 3.57 mmol, 1 eq.) in THF (1 mL) was added dropwise to a suspension of NaH (0.143 g, 3.57 mmol, 1 eq.) in THF (8 mL) at 0 °C. The reaction was allowed stir at room temperature for 1 h. Benzylbromide (0.47 mL, 3.93 mmol, 1.1 eq.) in THF (2 mL) was added dropwise to the mixture. The reaction was allowed stir overnight. The reaction was cooled to 0 °C and quenched with water (10 mL). The THF was removed by rotary evaporation. The aqueous layer was washed with EtOAc (3 x 20 mL). The organic layers were combined, dried $MgSO_4$, filtered and concentrated to afford the crude product as a yellow oil. Purification by column chromatography (c-Hex - EtOAc 4:1 to c-Hex – EtOAc 1:1) afforded the product a yellow oil (0.34 g, 65% yield).

^{1}H NMR (400 MHz, $CDCl_3$) δ 7.40 – 7.29 (m, 5H), 4.63 (s, 2H), 4.19 (d, J = 2.4 Hz, 2H), 2.48 (t, J = 2.4 Hz, 1H).

^{13}C{^{1}H} NMR (101 MHz, $CDCl_3$) δ 137.4, 128.6, 128.2, 128.0, 79.8, 74.7, 71.7, 57.2.

5. Chromatograms of 4-oxo-2-alkynyl dihydroquinolines

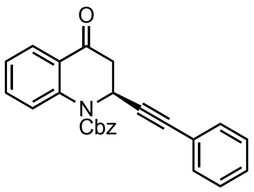

Benzyl (*S*)-4-oxo-2-(phenylethynyl)-3,4-dihydroquinoline-1(2H)-carboxylate (3a).[1]

UPLC analysis (CHIRALPAK OD, 90% Heptane and 10% IPA, 1 mL/min) t_R 8.70 min (Major) and t_R = 9.99 min (Minor)

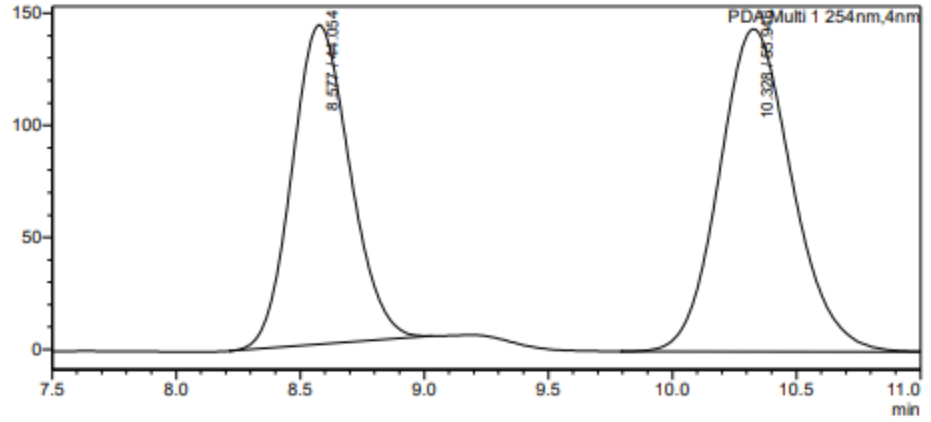

PDA Ch1 254nm

Peak#	Ret. Time	Area	Height	Name	Area%
1	8.577	2309794	142595		44.054
2	10.328	2933353	143978		55.946
Total		5243147	286573		100.000

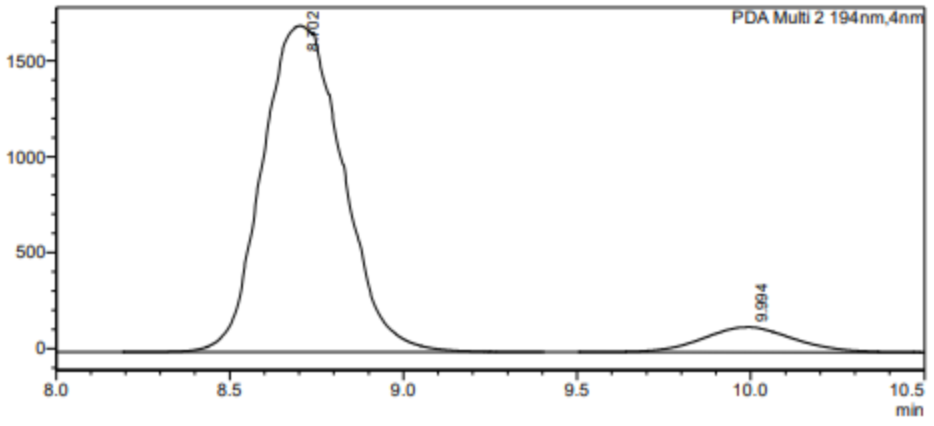

PDA Ch2 194nm

Peak#	Ret. Time	Area	Height	Conc.	Unit	Mark	Area%
1	8.702	27367951	1697558	0.000			91.973
2	9.994	2388644	130678	0.000			8.027
Total		29756594	1828236				100.000

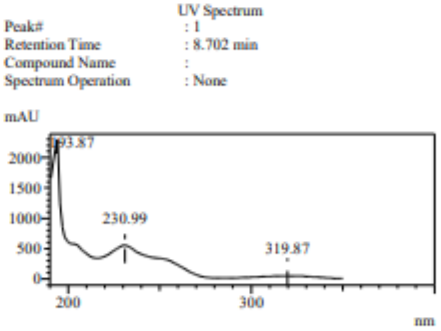

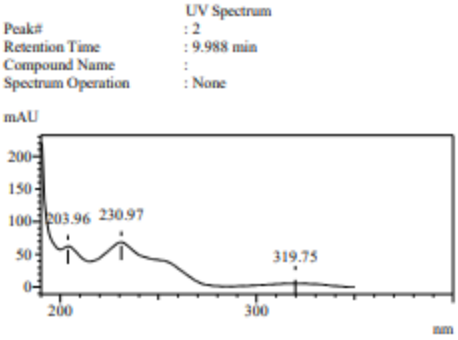

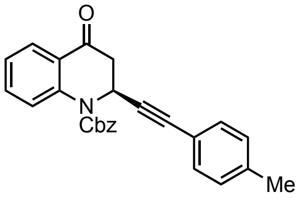

Benzyl (*S*)-4-oxo-2-(*p*-tolylethynyl)-3,4-dihydroquinoline-1(2H)-carboxylate (3b).[1]

UPLC analysis (CHIRALPAK OD, 90% Heptane and 10% IPA, 1 mL/min) t_R 8.48 min (Major) and t_R = 9.85 min (Minor)

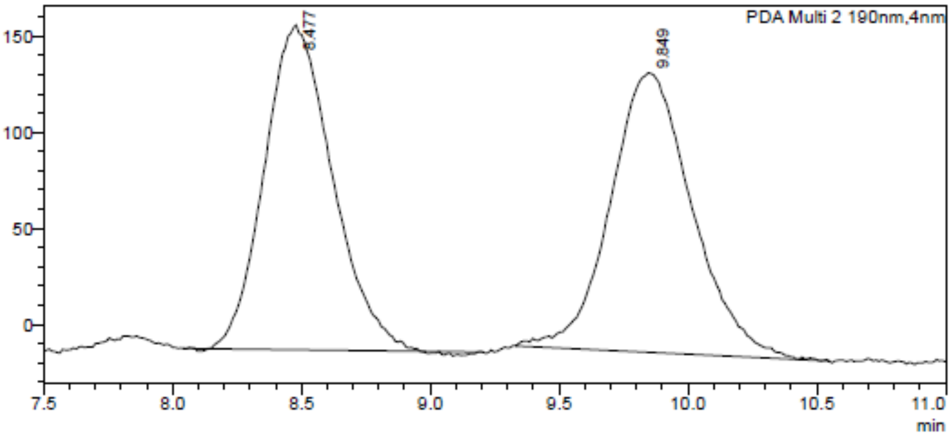

PDA Ch2 190nm

Peak#	Ret. Time	Area	Height	Conc.	Unit	Mark	Area%
1	8.477	3099250	168787	0.000		M	49.139
2	9.849	3207833	145359	0.000		M	50.861
Total		6307083	314146				100.000

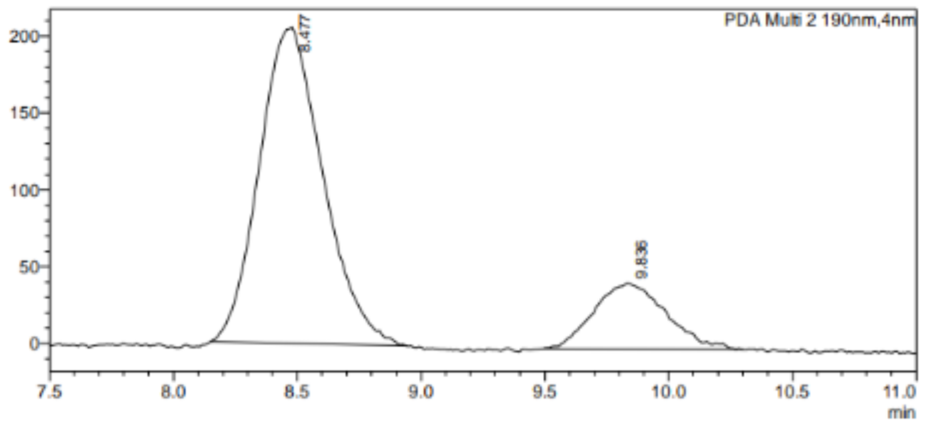

PDA Ch2 190nm

Peak#	Ret. Time	Area	Height	Unit	Mark	Name	Area%
1	8.477	3686253	205315		M		81.066
2	9.836	860973	42642		M		18.934
Total		4547226	247957				100.000

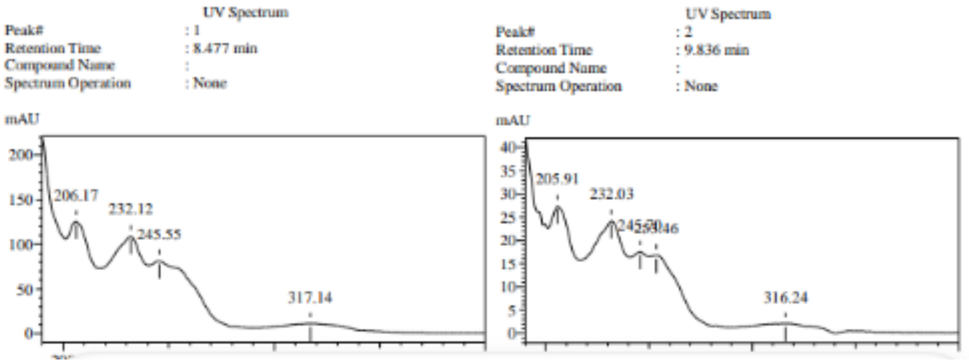

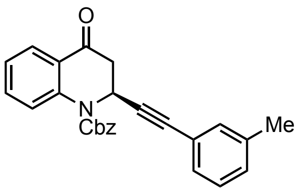

Benzyl (*S*)-4-oxo-2-(*m*-tolylethynyl)-3,4-dihydroquinoline-1(2H)-carboxylate (3c).[1]

UPLC analysis (CHIRALPAK OD, 90% Heptane and 10% IPA, 1 mL/min) t_R 9.74 min (Major) and t_R = 11.55 min (Minor).

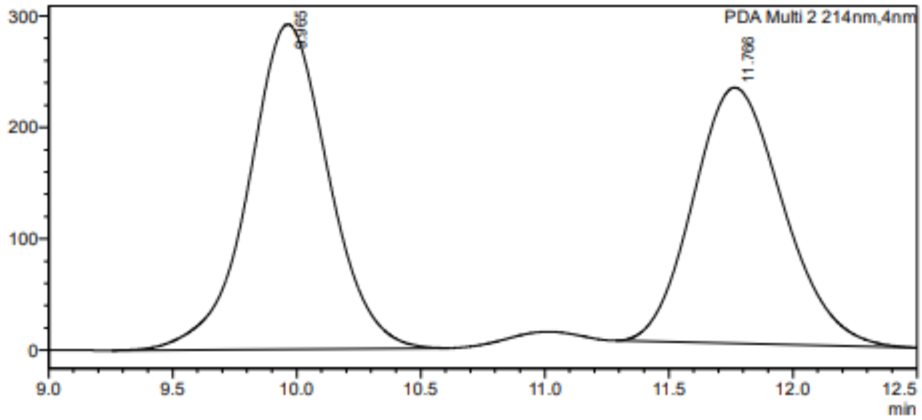

PDA Ch2 214nm

Peak#	Ret. Time	Area	Height	Conc.	Unit	Mark	Area%
1	9.965	6527163	291703	0.000			52.992
2	11.766	5790070	229653	0.000		S	47.008
Total		12317232	521355				100.000

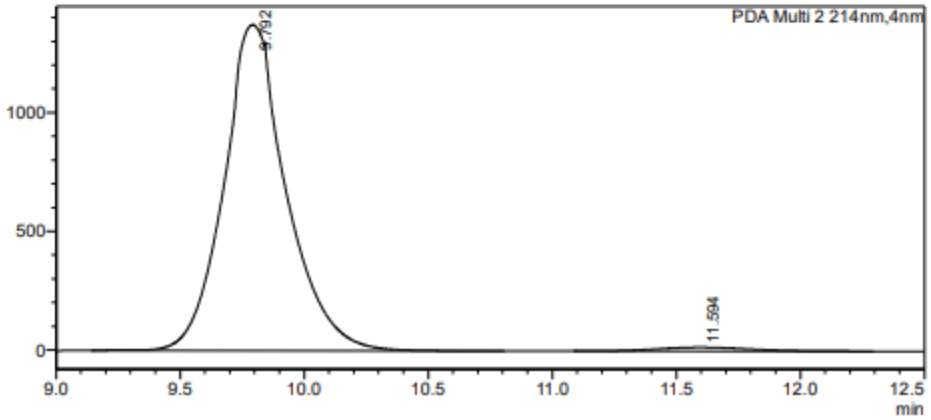

PDA Ch2 214nm

Peak#	Ret. Time	Area	Height	Conc.	Area%
1	9.792	24025254	1371120	0.000	98.267
2	11.594	423586	16269	0.000	1.733
Total		24448840	1387389		100.000

UV Spectrum

Peak# : 1
Retention Time : 9.792 min
Compound Name :
Spectrum Operation : None

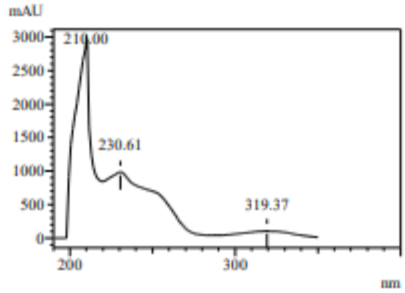

UV Spectrum

Peak# : 2
Retention Time : 11.594 min
Compound Name :
Spectrum Operation : None

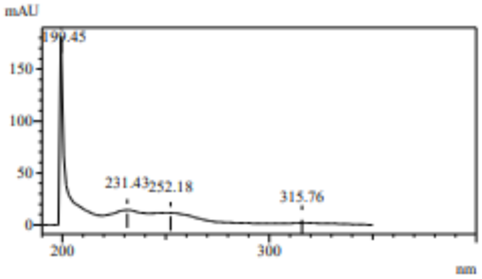

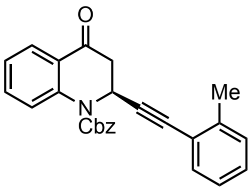

Benzyl (*S*)-4-oxo-2-(*o*-tolylethynyl)-3,4-dihydroquinoline-1(2H)-carboxylate (3d).[1]

UPLC analysis (CHIRALPAK OD, 90% Heptane and 10% IPA, 1 mL/min) t_R 8.25 min (Major) and t_R = 8.97 min (Minor)

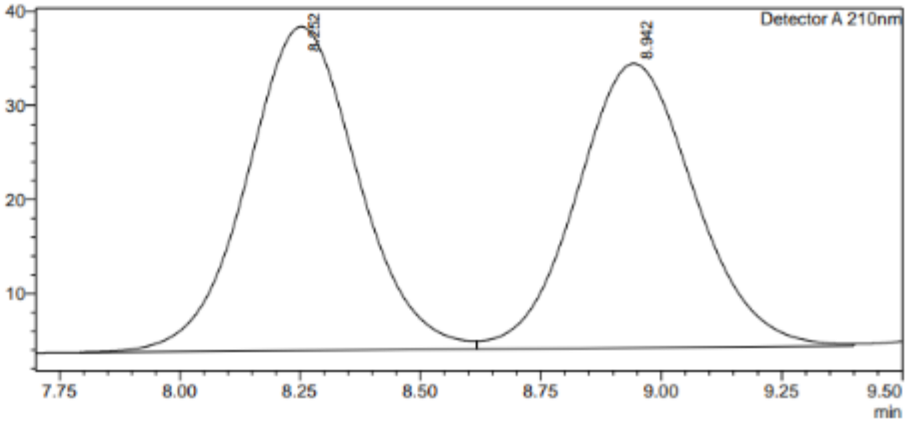

Peak#	Ret. Time	Area	Height	Unit	Mark	Name	Area%
1	8.252	552637	34341				51.859
2	8.942	513022	30150		V		48.141
Total		1065660	64491				100.000

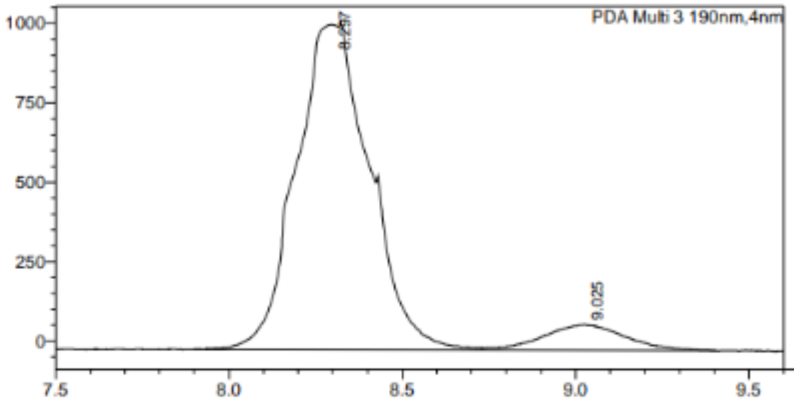

PDA Ch3 190nm

Peak#	Ret. Time	Area	Height	Unit	Mark	Name	Area%
1	8.297	15202031	1022877		M		91.933
2	9.025	1334036	80717		V M		8.067
Total		16536067	1103594				100.000

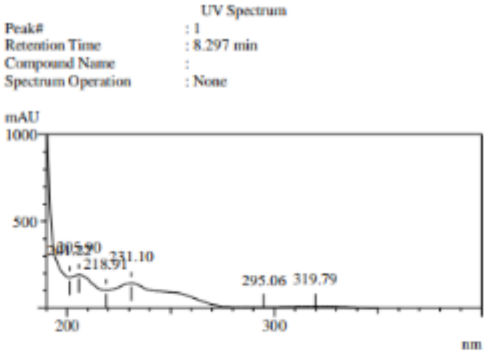

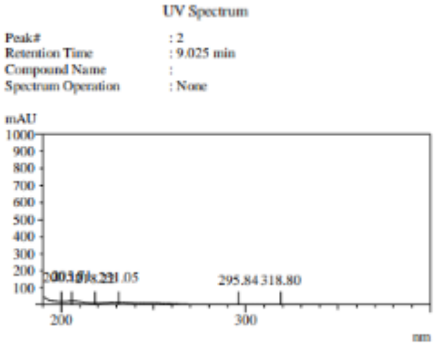

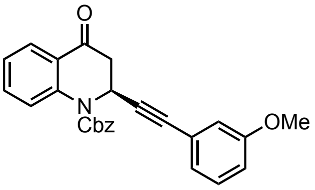

Benzyl (*S*)-2-((3-methoxyphenyl)ethynyl)-4-oxo-3,4-dihydroquinoline-1(2H)-carboxylate (3e).[1]

UPLC analysis (CHIRALPAK OD, 90% Heptane and 10% IPA, 1 mL/min) t_R 10.68 min (Major) and t_R = 12.3 min (Minor)

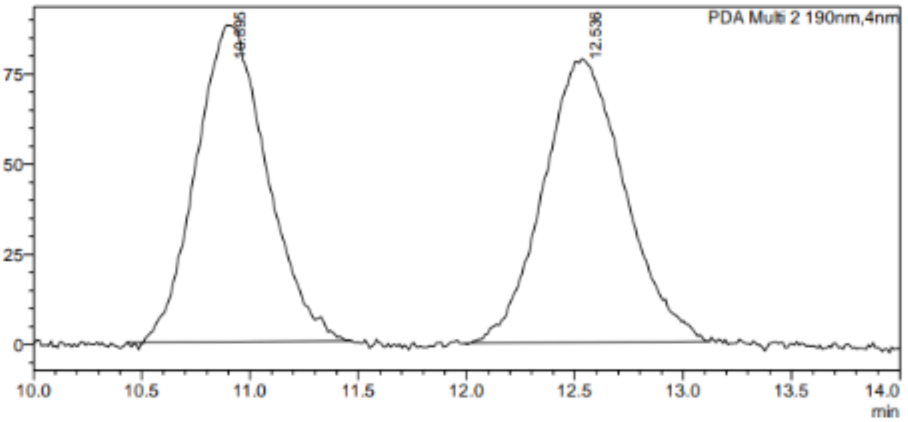

PDA Ch2 190nm

Peak#	Ret. Time	Area	Height	Unit	Mark	Name	Area%
1	10.895	2000670	87696		M		49.331
2	12.536	2054973	78490		M		50.669
Total		4055643	166186				100.000

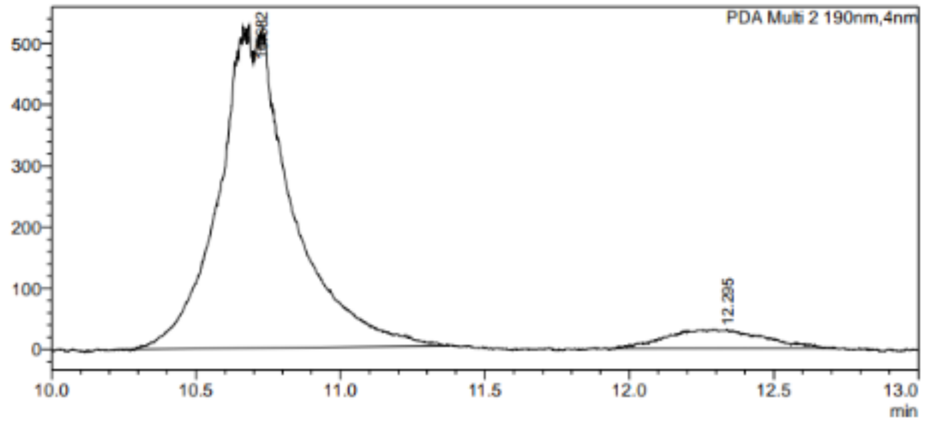

PDA Ch2 190nm

Peak#	Ret. Time	Area	Height	Unit	Mark	Name	Area%
1	10.682	9265467	516047		M		92.873
2	12.295	711005	30303		M		7.127
Total		9976472	546350				100.000

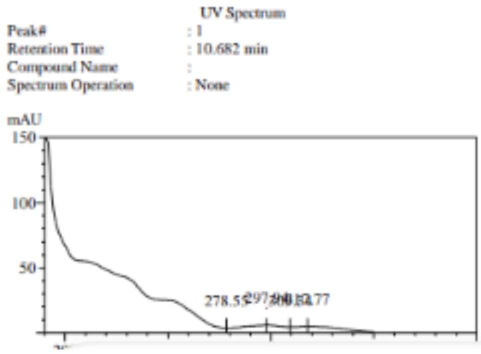

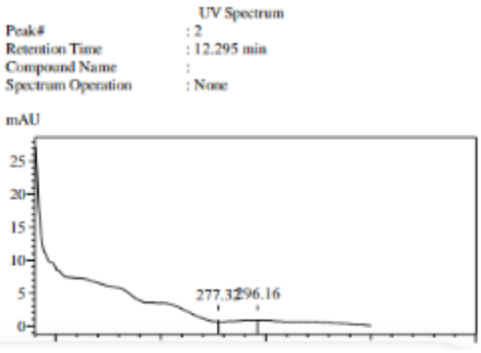

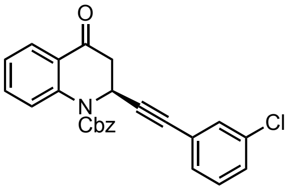

Benzyl (*S*)-2-((3-chlorophenyl)ethynyl)-4-oxo-3,4-dihydroquinoline-1(2H)-carboxylate (3f).[1]

UPLC analysis (CHIRALPAK ID, 90% Heptane and 10% isopropanol, 1 mL/min) t_R 9.04 min (Major) and t_R = 11.06 min (Minor)

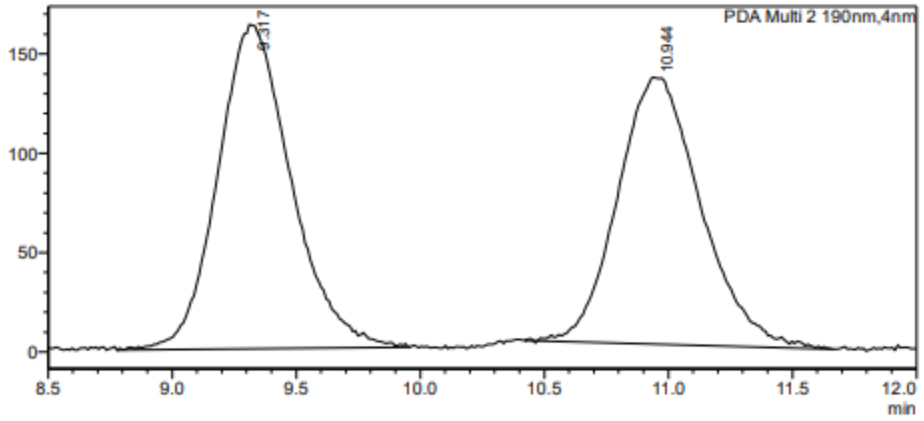

PDA Ch2 190nm

Peak#	Ret. Time	Area	Height	Conc.	Unit	Mark	Area%
1	9.317	3346817	162975	0.000			51.611
2	10.944	3137820	134295	0.000			48.389
Total		6484636	297270				100.000

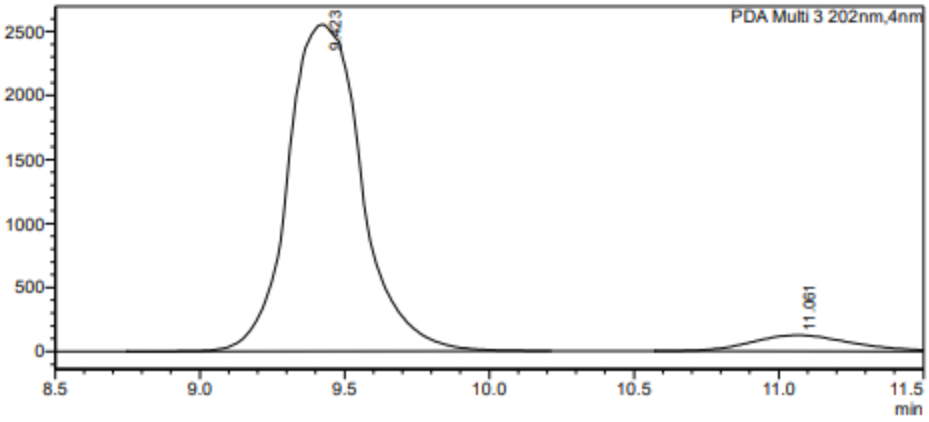

PDA Ch3 202nm

Peak#	Ret. Time	Area	Height	Conc.	Unit	Mark	Area%
1	9.423	45242367	2549793	0.000		M	93.762
2	11.061	3009982	124320	0.000		M	6.238
Total		48252350	2674113				100.000

UV Spectrum

Peak# : 1
Retention Time : 9.605 min
Compound Name :
Spectrum Operation : None

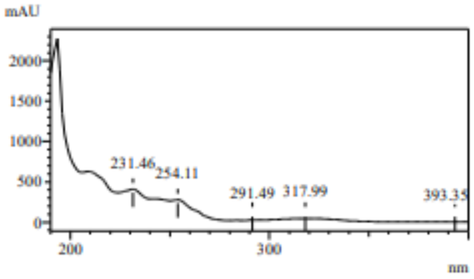

UV Spectrum

Peak# : 2
Retention Time : 11.061 min
Compound Name :
Spectrum Operation : None

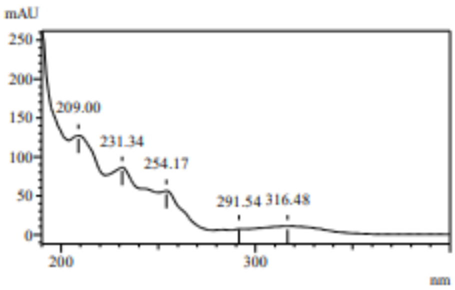

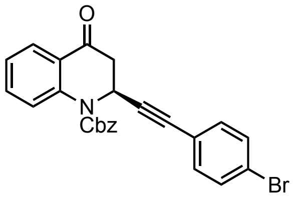

Benzyl (*S*)-2-((4-bromophenyl)ethynyl)-4-oxo-3,4-dihydroquinoline-1(2H)-carboxylate (3g).

UPLC analysis (CHIRALPAK OD, 90% Heptane and 10% IPA, 1 mL/min) t_R 10.00 min (Major) and t_R = 11.19 min (Minor).

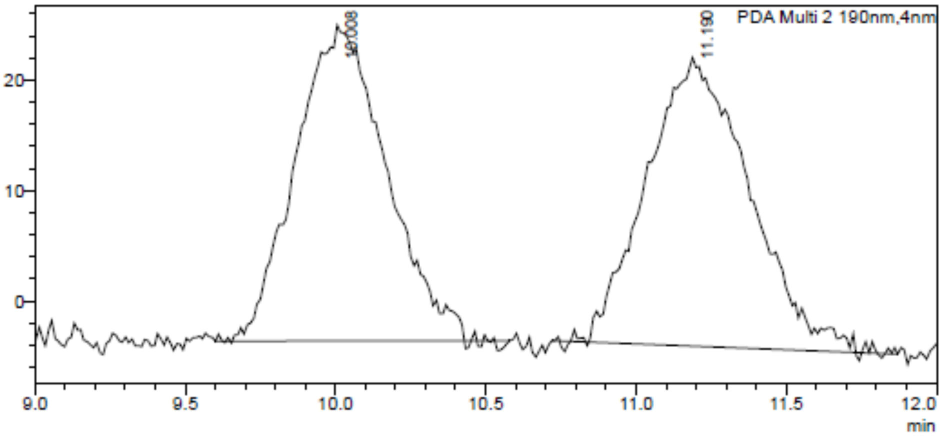

PDA Ch2 190nm

Peak#	Ret. Time	Area	Height	Conc.	Unit	Mark	Area%
1	10.008	599000	28569	0.000		M	49.044
2	11.190	622346	26111	0.000		M	50.956
Total		1221346	54680				100.000

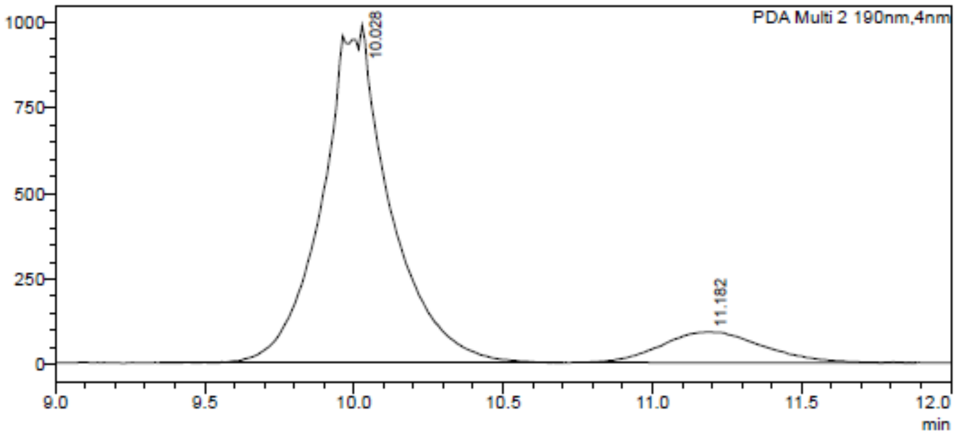

PDA Ch2 190nm

Peak#	Ret. Time	Area	Height	Conc.	Unit	Mark	Area%
1	10.028	15718360	985773	0.000		M	87.913
2	11.182	2161163	90084	0.000		M	12.087
Total		17879523	1075858				100.000

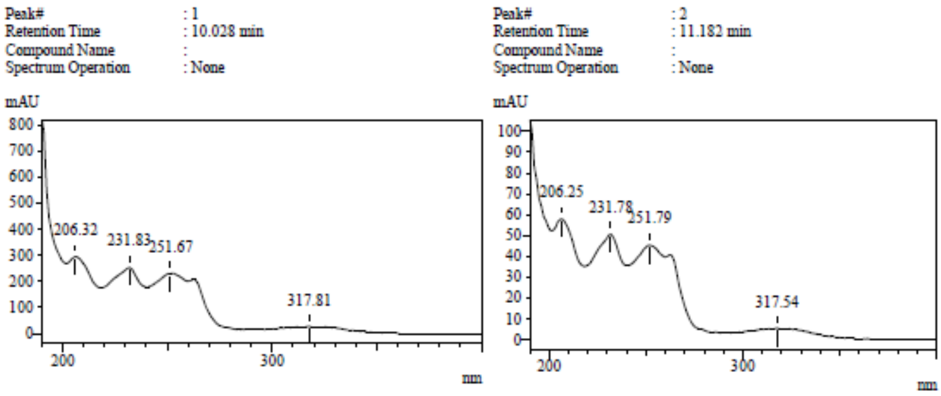

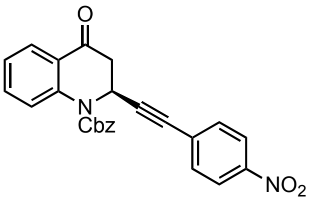

Benzyl (*S*)-2-((4-nitrophenyl)ethynyl)-4-oxo-3,4-dihydroquinoline-1(2H)-carboxylate (3h).

UPLC analysis (CHIRACEL AD, 90% heptane and 10% isopropanol, 1mL/min) t_R = 21.86 min (Major) and t_R = 27.16 min (Minor).

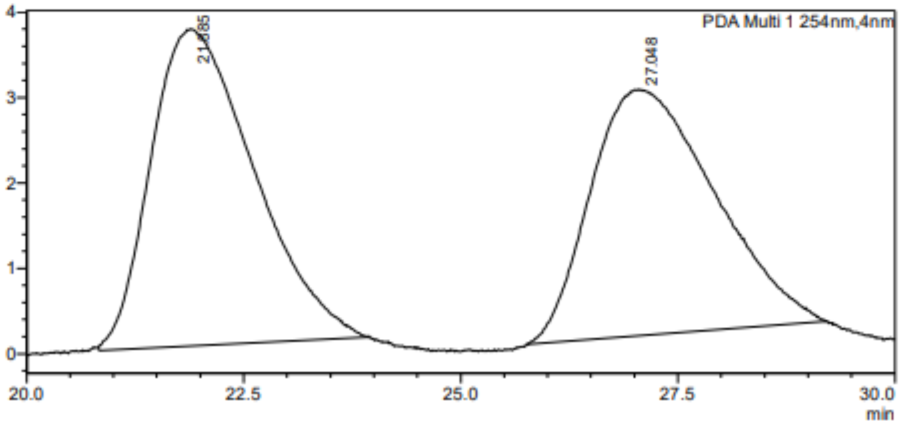

PDA Ch1 254nm

Peak#	Ret. Time	Area	Height	Conc.	Unit	Mark	Area%
1	21.885	306491	3709	0.000		V	51.776
2	27.048	285468	2884	0.000			48.224
Total		591959	6593				100.000

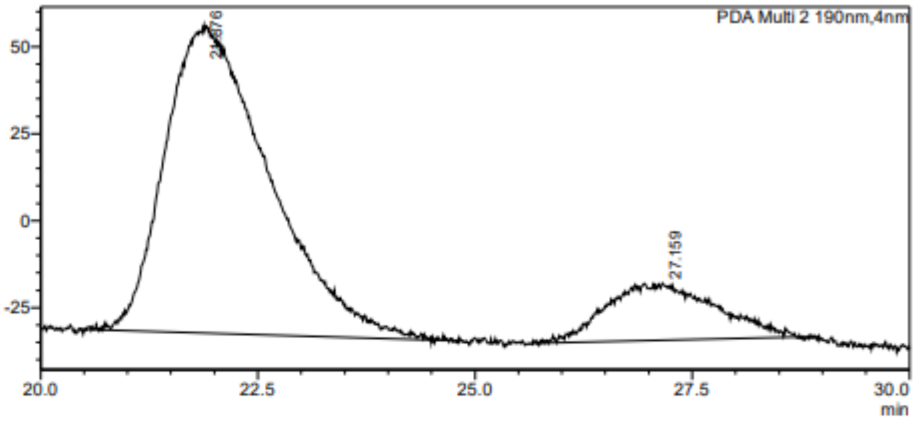

PDA Ch2 190nm

Peak#	Ret. Time	Area	Height	Conc.	Unit	Mark	Area%
1	21.876	7367854	88438	0.000		M	83.727
2	27.159	1431949	16387	0.000		M	16.273
Total		8799804	104825				100.000

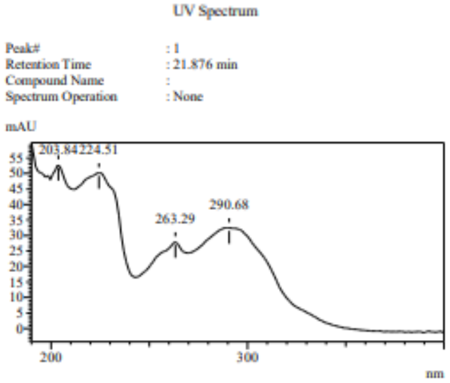

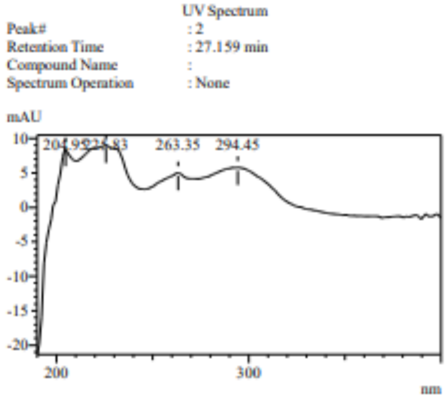

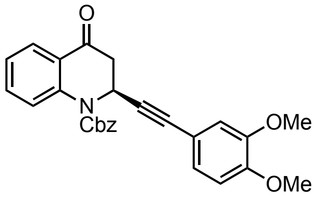

Benzyl (*S*)-2-((3,4-dimethoxyphenyl)ethynyl)-4-oxo-3,4-dihydroquinoline-1(2H)-carboxylate (3i).[1]

UPLC analysis (CHIRACEL AD, 90% heptane and 10% isopropanol, 1mL/min) t_R = 16.6 min (Major) and t_R = 19.9 min (Minor).

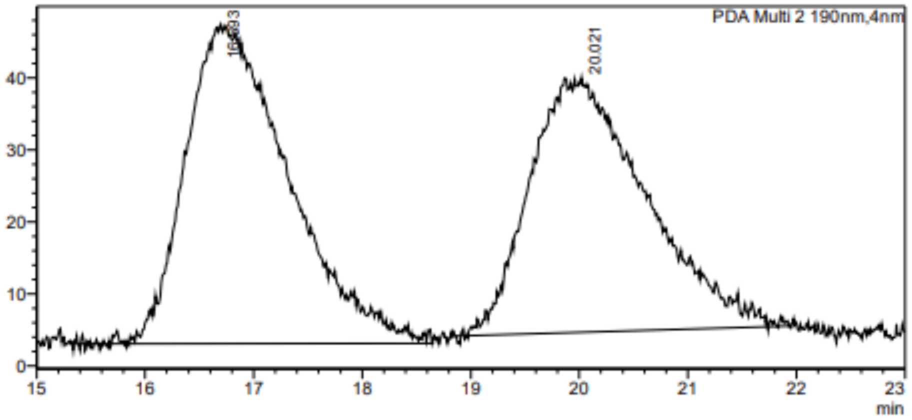

PDA Ch2 190nm

Peak#	Ret. Time	Area	Height	Conc.	Unit	Mark	Area%
1	16.693	2923407	44279	0.000		M	52.640
2	20.021	2630164	35345	0.000		M	47.360
Total		5553571	79624				100.000

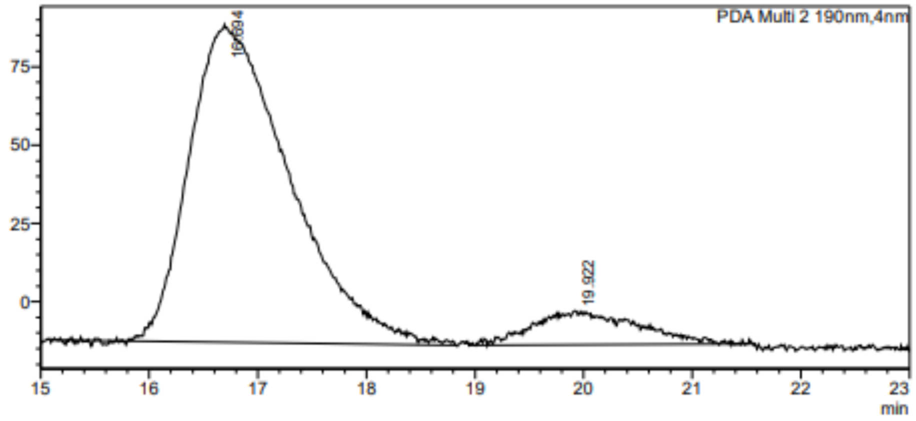

PDA Ch2 190nm

Peak#	Ret. Time	Area	Height	Conc.	Unit	Mark	Area%
1	16.694	6510558	101370	0.000		M	89.952
2	19.922	727273	10857	0.000		M	10.048
Total		7237832	112228				100.000

UV Spectrum

Peak# : 1
Retention Time : 16.694 min
Compound Name :
Spectrum Operation : None

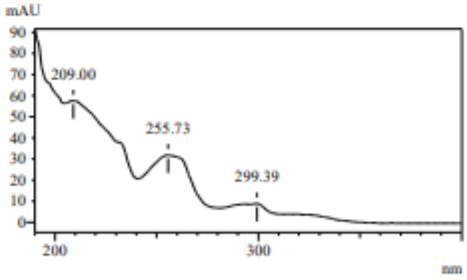

UV Spectrum

Peak# : 2
Retention Time : 19.922 min
Compound Name :
Spectrum Operation : None

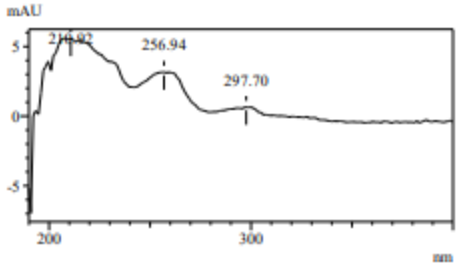

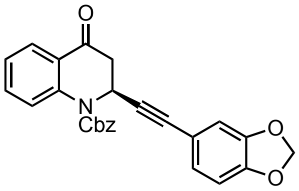

Benzyl (*S*)-2-(benzo[d][1,3]dioxol-5-ylethynyl)-4-oxo-3,4-dihydroquinoline-1(2H)-carboxylate (3j).

UPLC analysis (CHIRACEL AD, 90% heptane and 10% isopropanol, 1mL/min) t_R = 14.91 min (Major) and t_R = 17.21 min (Minor).

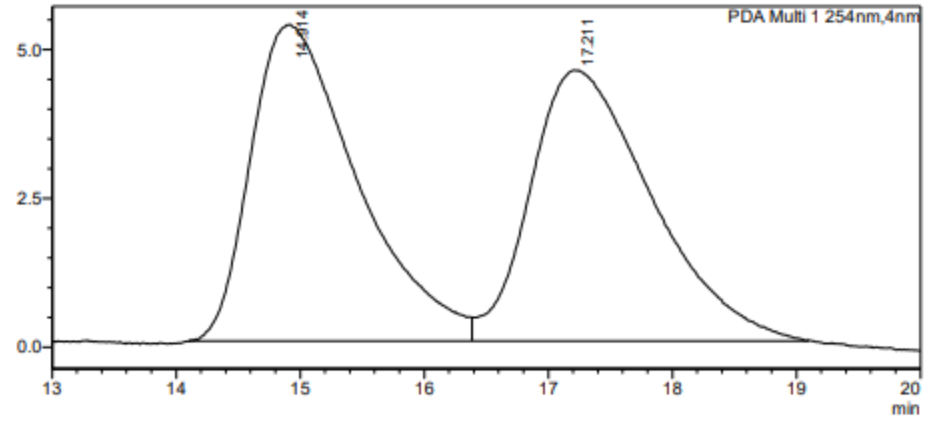

PDA Ch1 254nm

Peak#	Ret. Time	Area	Height	Conc.	Unit	Mark	Area%
1	14.914	316699	5314	0.000			50.670
2	17.211	308325	4550	0.000		V	49.330
Total		625024	9863				100.000

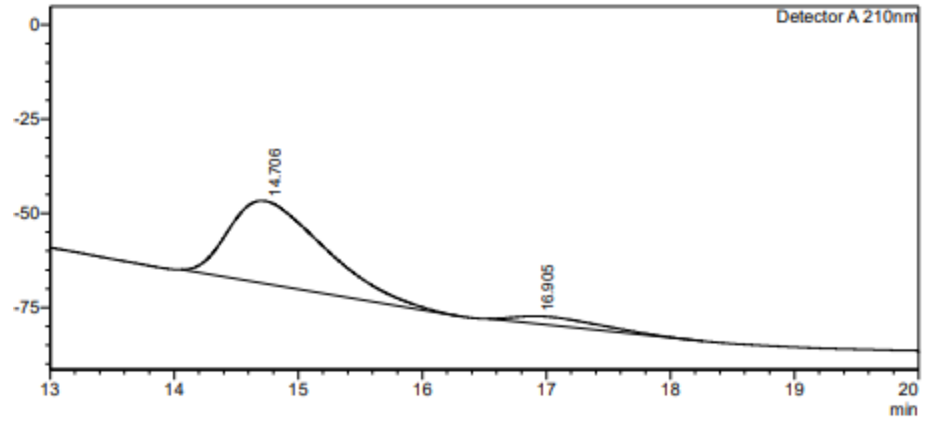

Detector A 210nm

Peak#	Ret. Time	Area	Height	Conc.	Unit	Mark	Area%
1	14.706	1186702	21872	91.741			91.741
2	16.905	106829	1973	8.259			8.259
Total		1293531	23845				100.000

UV Spectrum

Peak# : 1
Retention Time : 14.934 min
Compound Name :
Spectrum Operation : None

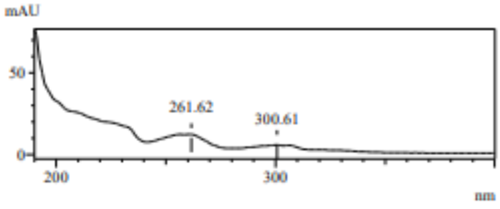

UV Spectrum

Peak# : 2
Retention Time : 17.250 min
Compound Name :
Spectrum Operation : None

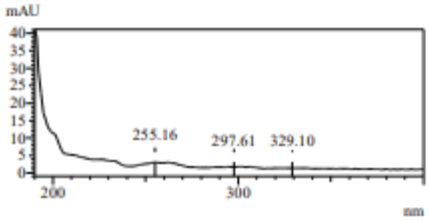

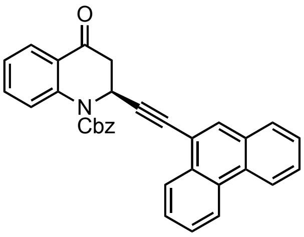

Benzyl (*S*)-4-oxo-2-(phenanthren-9-ylethynyl)-3,4-dihydroquinoline-1(2H)-carboxylate (3k).

UPLC analysis (CHIRACEL AD, 90% heptane and 10% isopropanol, 1mL/min) t_R = 7.12 min (Minor) and t_R = 9.87 min (Major).

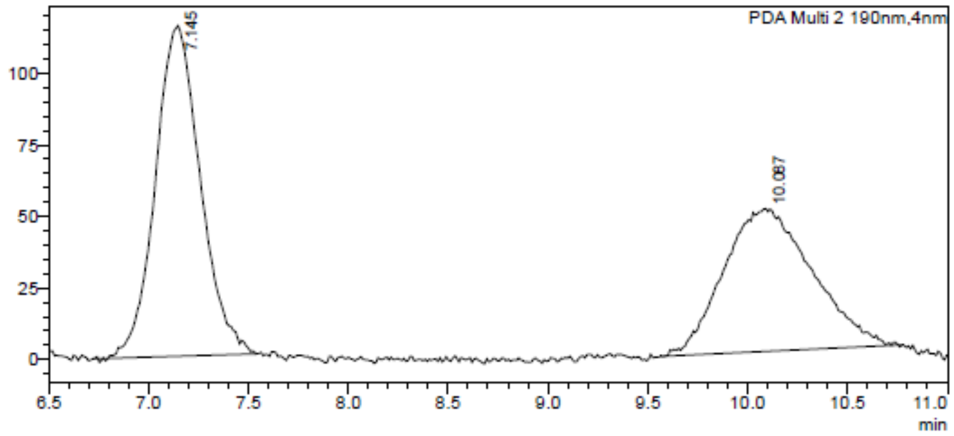

PDA Ch2 190nm

Peak#	Ret. Time	Area	Height	Unit	Mark	Name	Area%
1	7.145	1805586	115202		M		54.087
2	10.087	1532700	49742		M		45.913
Total		3338286	164944				100.000

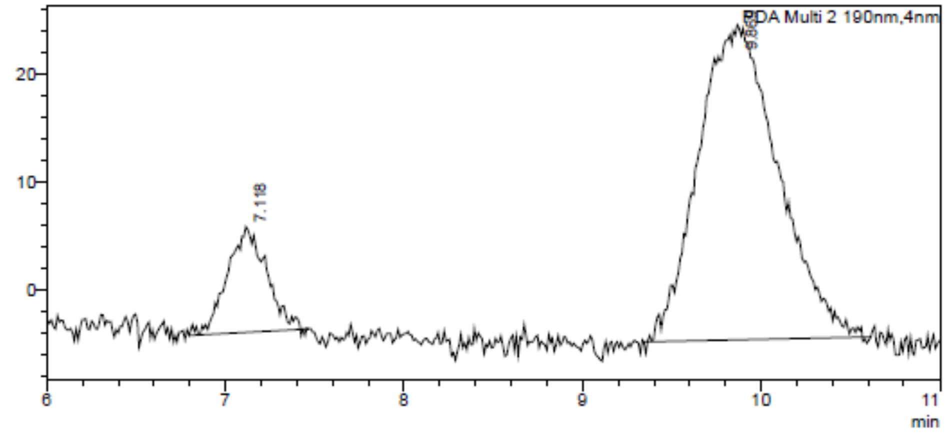

PDA Ch2 190nm

Peak#	Ret. Time	Area	Height	Unit	Mark	Name	Area%
1	7.118	149927	9838		M		14.165
2	9.869	908504	29273		M		85.835
Total		1058431	39111				100.000

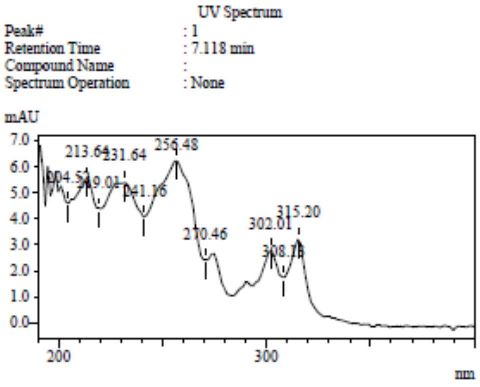

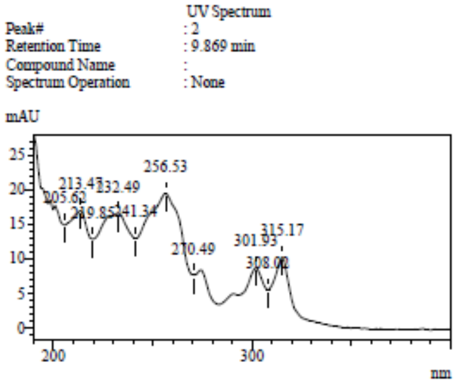

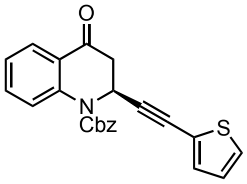

Benzyl (*S*)-4-oxo-2-(thiophen-2-ylethynyl)-3,4-dihydroquinoline-1(2H)-carboxylate (3l).

UPLC analysis (CHIRACEL AD, 90% heptane and 10% isopropanol, 1mL/min) t_R = 22.75 min (Major) and t_R = 24.37 min (Minor).

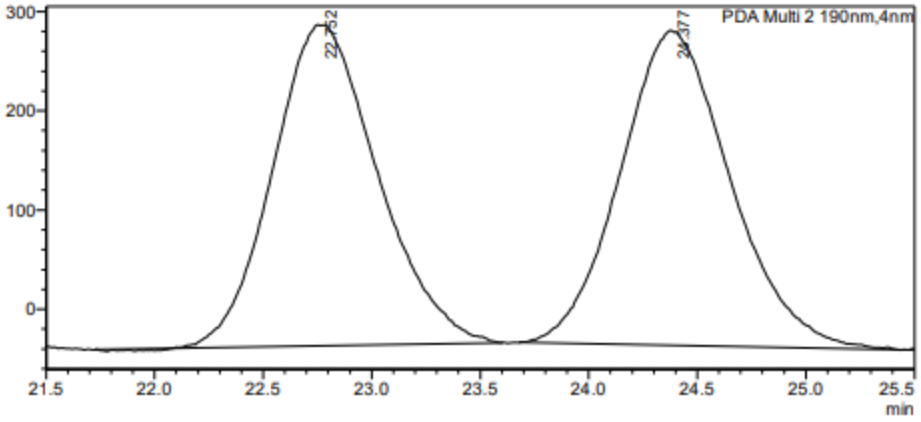

PDA Ch2 190nm

Peak#	Ret. Time	Area	Height	Conc.	Unit	Mark	Area%
1	22.752	10847915	323681	0.000		M	49.322
2	24.377	11146287	317187	0.000		M	50.678
Total		21994202	640867				100.000

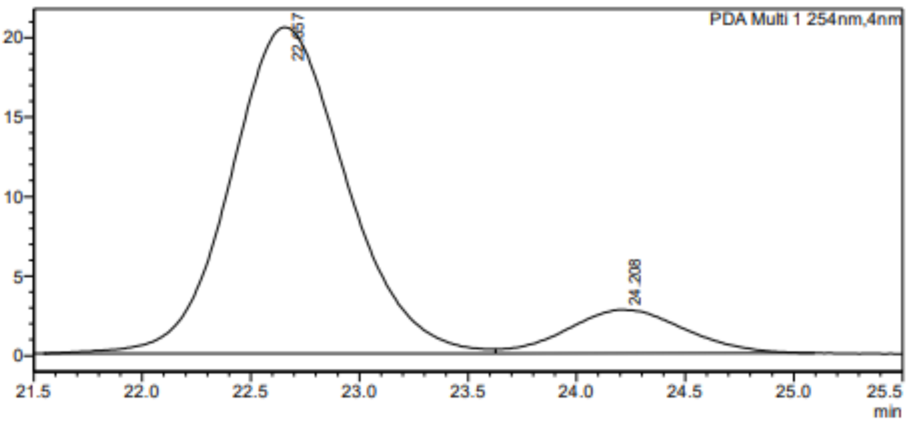

PDA Ch2 190nm

Peak#	Ret. Time	Area	Height	Conc.	Unit	Mark	Area%
1	22.685	1400950	40126	0.000		M	91.185
2	24.303	135434	4841	0.000		M	8.815
Total		1536385	44968				100.000

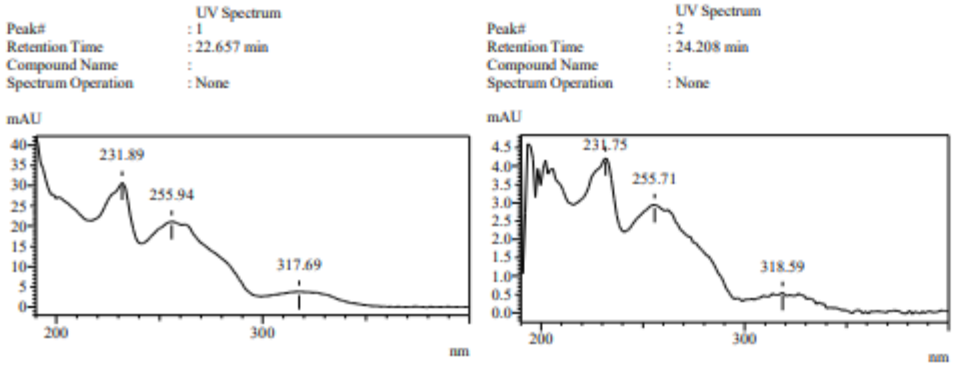

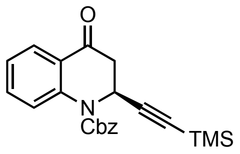

Benzyl (*S*)-4-oxo-2-((trimethylsilyl)ethynyl)-3,4-dihydroquinoline-1(2H)-carboxylate (3m).

UPLC analysis (CHIRALPAK OJ, 90% Heptane and 10% isopropanol, 0.5 mL/min) t_R = 7.26 min (Minor) and t_R = 9.18 min (Major)

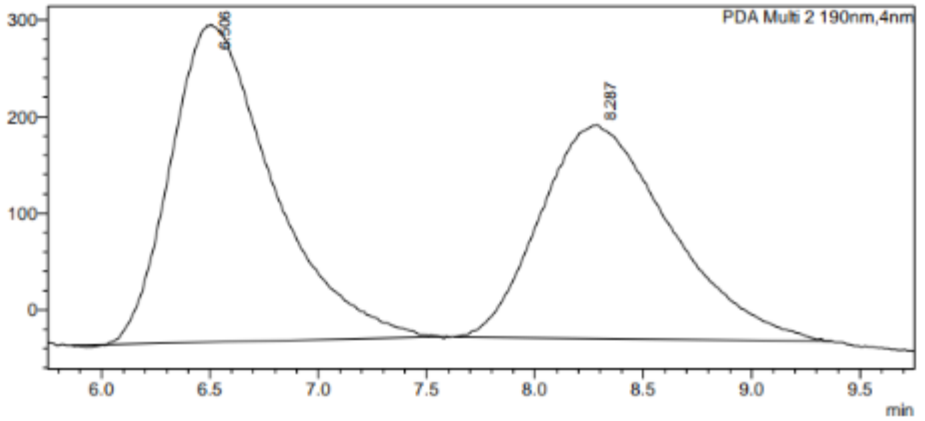

PDA Ch2 190nm

Peak#	Ret. Time	Area	Height	Unit	Mark	Name	Area%
1	6.506	10791873	328364		M		53.969
2	8.287	9204565	221007		M		46.031
Total		19996439	549371				100.000

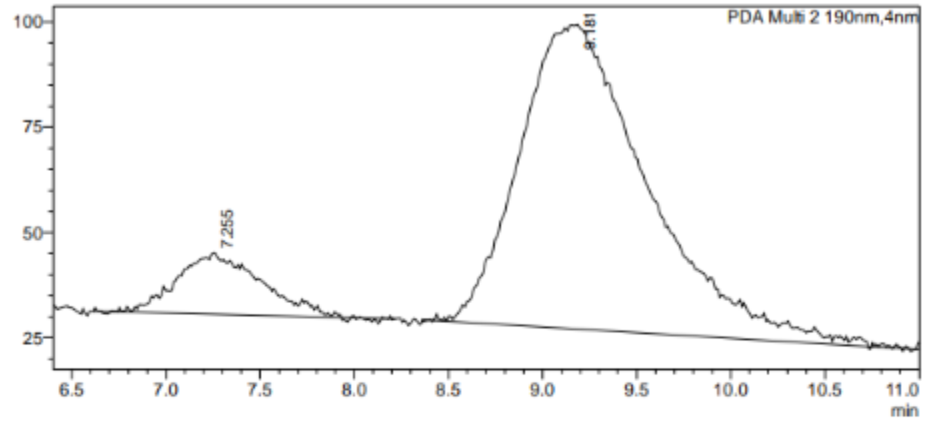

PDA Ch2 190nm

Peak#	Ret. Time	Area	Height	Unit	Mark	Name	Area%
1	7.255	445983	14557		M		11.520
2	9.181	3425480	72306		M		88.480
Total		3871464	86863				100.000

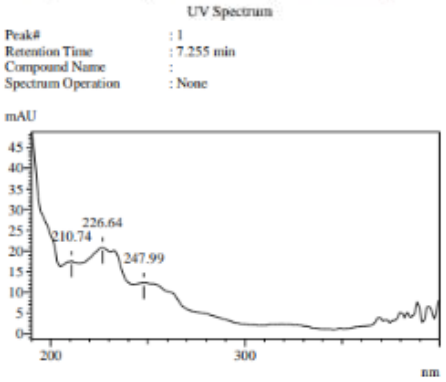

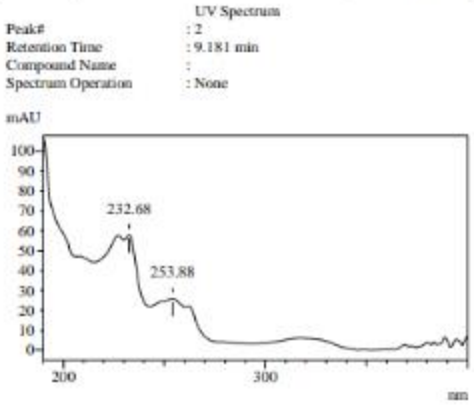

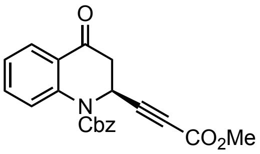

Benzyl (*S*)-2-(3-methoxy-3-oxoprop-1-yn-1-yl)-4-oxo-3,4-dihydroquinoline-1(2H)-carboxylate (3n).[1]

SFC analysis (CHIRALPAK OD, 90% Heptane and 10% IPA, 1 mL/min) t_R 8.07 min (Major) and t_R = 9.53 min (Minor).

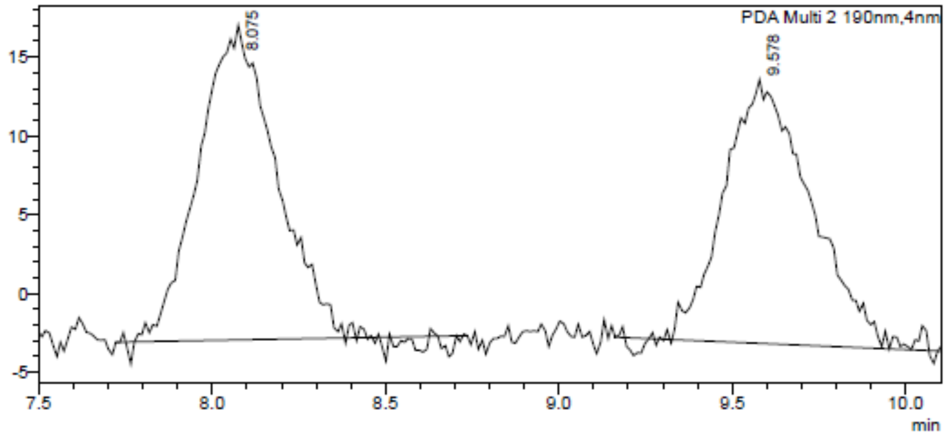

PDA Ch2 190nm

Peak#	Ret. Time	Area	Height	Conc.	Unit	Mark	Area%
1	8.075	304671	19940	0.000		M	51.083
2	9.578	291748	16716	0.000		M	48.917
Total		596418	36656				100.000

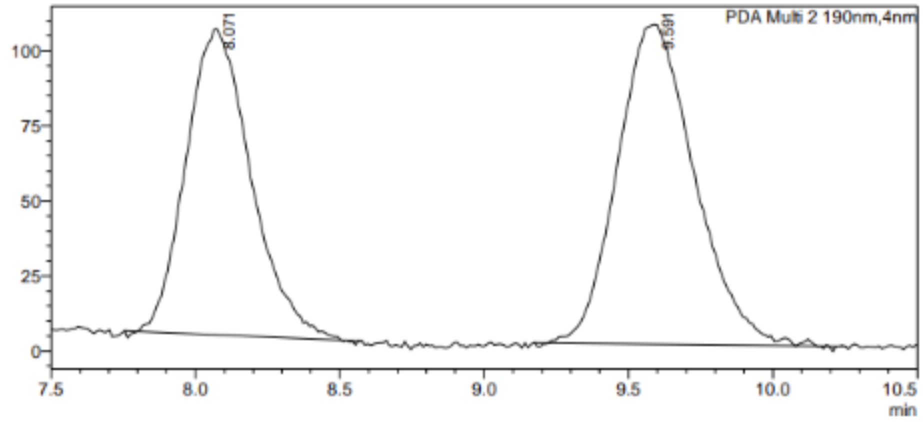

PDA Ch2 190nm

Peak#	Ret. Time	Area	Height	Unit	Mark	Name	Area%
1	8.071	1593577	101891		M		44.566
2	9.591	1982180	106409		M		55.434
Total		3575757	208300				100.000

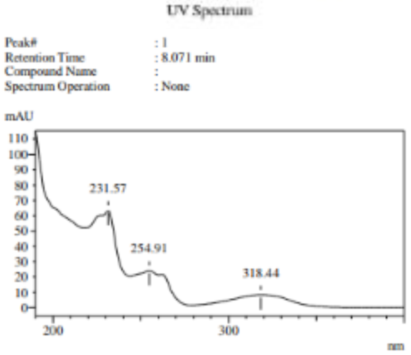

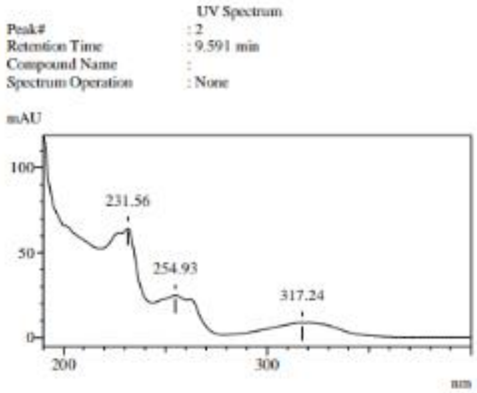

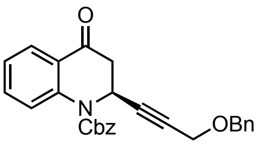

Benzyl (*S*)-2-(3-(benzyloxy)prop-1-yn-1-yl)-4-oxo-3,4-dihydroquinoline-1(2H)-carboxylate (3o).

UPLC analysis (CHIRACEL OD, 90% heptane and 10% isopropanol, 1mL/min) t_R = 15.6 min (Major) and t_R = 17.3 min (Minor).

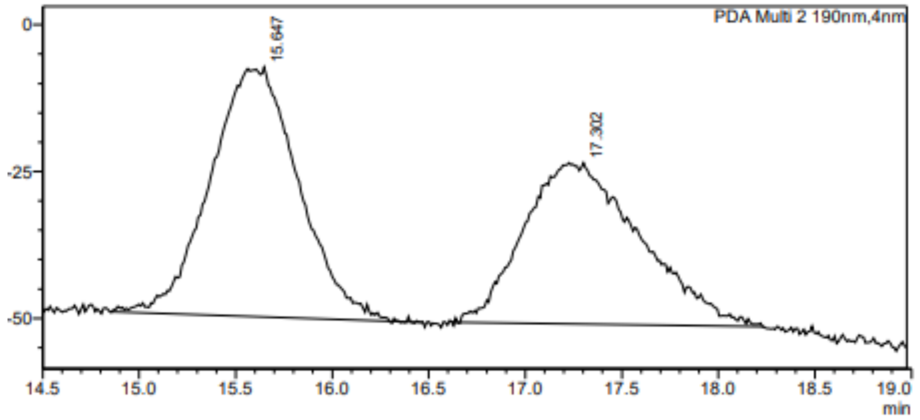

PDA Ch2 190nm

Peak#	Ret. Time	Area	Height	Conc.	Unit	Mark	Area%
1	15.647	1353380	42509	0.000		M	54.003
2	17.302	1152757	27467	0.000		M	45.997
Total		2506138	69976				100.000

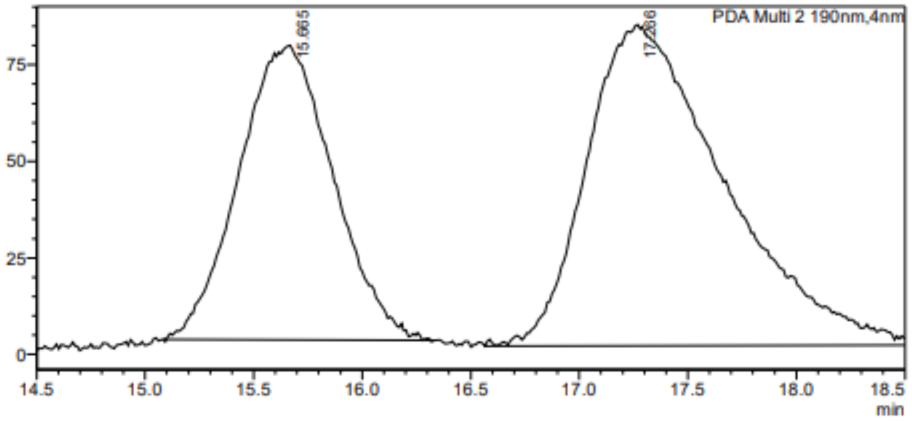

PDA Ch2 190nm

Peak#	Ret. Time	Area	Height	Conc.	Unit	Mark	Area%
1	15.665	2307200	76236	0.000		M	38.402
2	17.266	3700890	83049	0.000		M	61.598
Total		6008090	159285				100.000

UV Spectrum

Peak# : 1
Retention Time : 15.665 min
Compound Name :
Spectrum Operation : None

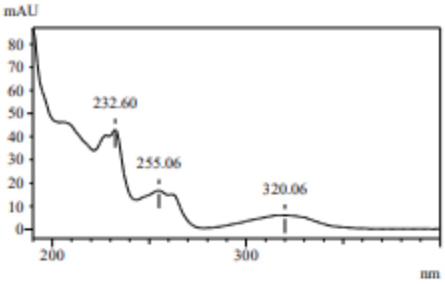

UV Spectrum

Peak# : 2
Retention Time : 17.266 min
Compound Name :
Spectrum Operation : None

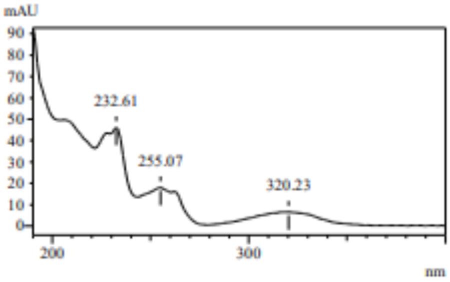

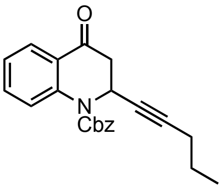

Benzyl (*S*)-4-oxo-2-(pent-1-yn-1-yl)-3,4-dihydroquinoline-1(2*H*)-carboxylate (3p).

UPLC analysis (CHIRACEL OD, 90% heptane and 10% isopropanol, 1mL/min) t_R = 7.07 min (Major) and t_R = 7.64 min (Minor).

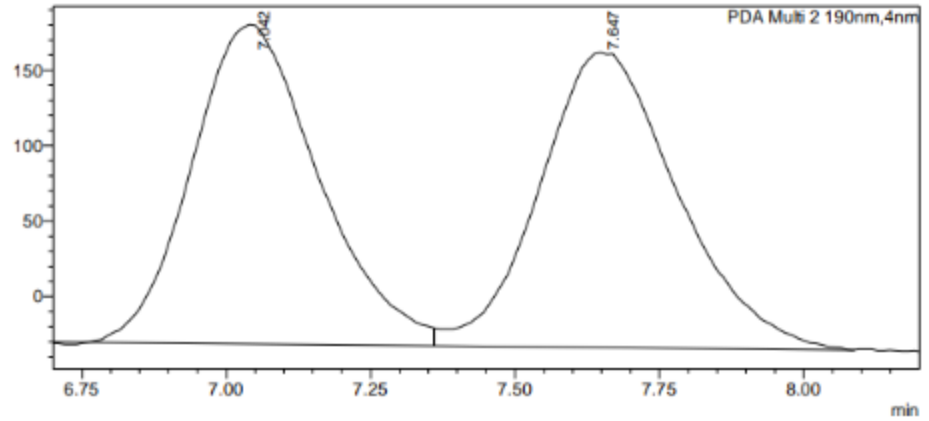

PDA Ch2 190nm

Peak#	Ret. Time	Area	Height	Unit	Mark	Name	Area%
1	7.042	3226477	211508		M		49.854
2	7.647	3245328	195334		V M		50.146
Total		6471805	406842				100.000

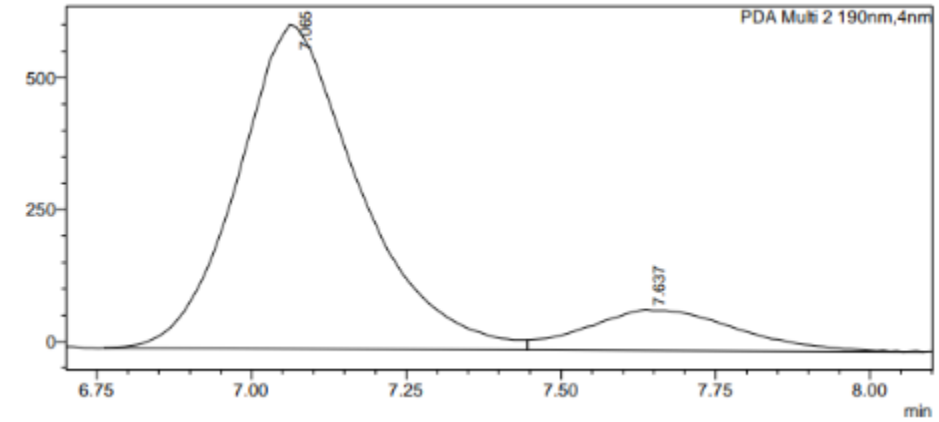

PDA Ch2 190nm

Peak#	Ret. Time	Area	Height	Unit	Mark	Name	Area%
1	7.065	8413445	613922		M		86.613
2	7.637	1300347	77303		V M		13.387
Total		9713792	691224				100.000

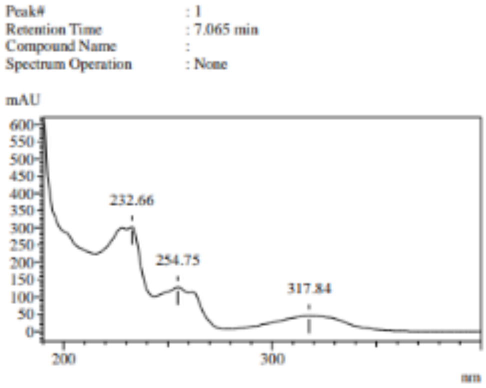

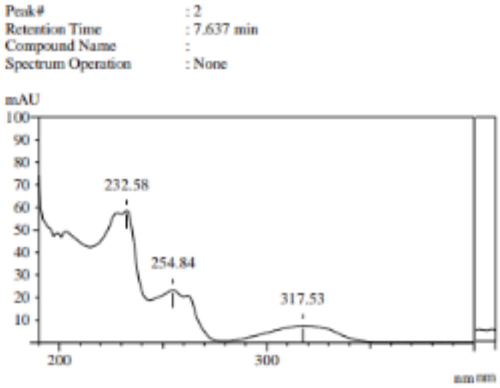

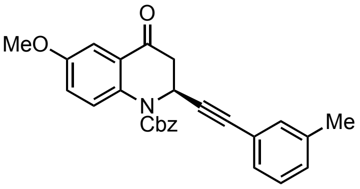

Benzyl (*S*)-6-methoxy-4-oxo-2-(*m*-tolylethynyl)-3,4-dihydroquinoline-1(2H)-carboxylate (3q).[1]

UPLC analysis (CHIRALPAK OD, 90% heptane and 10% isopropanol, 1 mL/min) t_R 9.15 min (Major) and t_R = 11.18 min (Minor).

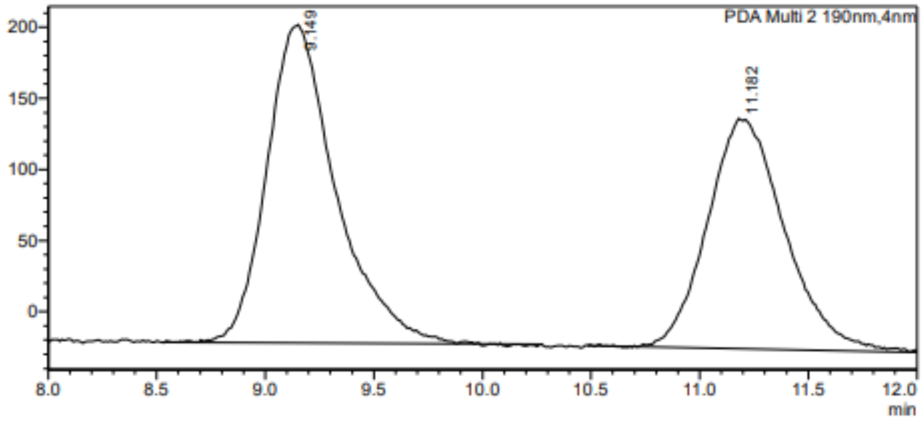

PDA Ch2 190nm

Peak#	Ret. Time	Area	Height	Conc.	Unit	Mark	Area%
1	9.149	4959156	223809	0.000		M	55.254
2	11.182	4015997	161847	0.000		M	44.746
Total		8975153	385656				100.000

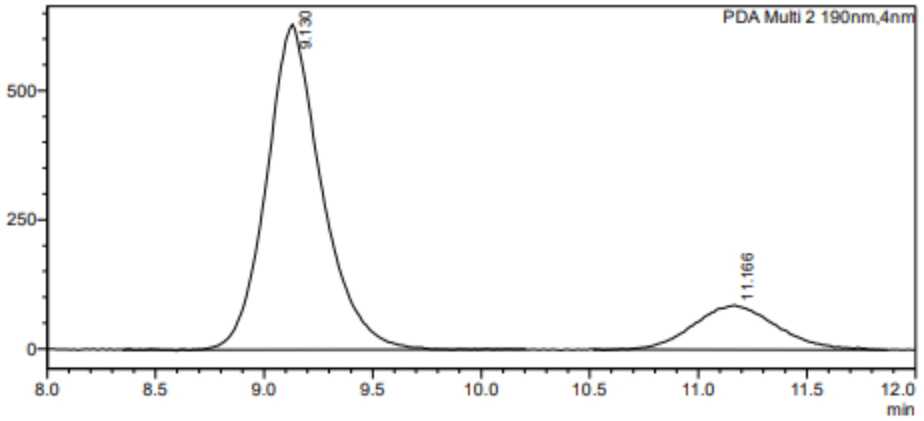

PDA Ch2 190nm

Peak#	Ret. Time	Area	Height	Conc.	Unit	Mark	Area%
1	9.130	11120190	629522	0.000		M	83.025
2	11.166	2273632	85556	0.000		M	16.975
Total		13393822	715078				100.000

UV Spectrum
Peak# : 1
Retention Time : 9.130 min
Compound Name :
Spectrum Operation : None

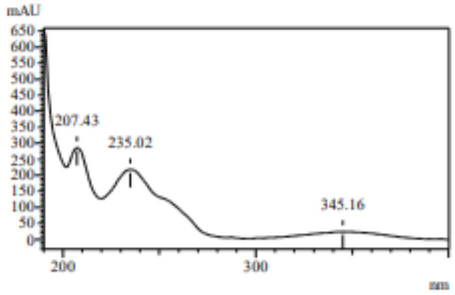

UV Spectrum
Peak# : 2
Retention Time : 11.166 min
Compound Name :
Spectrum Operation : None

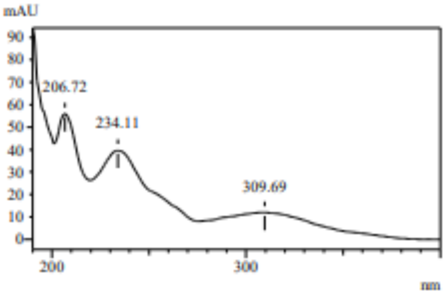

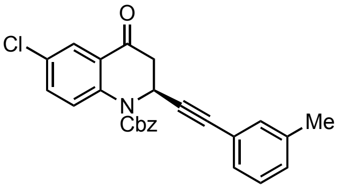

Benzyl (*S*)-6-chloro-4-oxo-2-(*m*-tolylethynyl)-3,4-dihydroquinoline-1(2H)-carboxylate (3r).

UPLC analysis (CHIRALPAK OD, 90% heptane and 10% isopropanol, 1 mL/min) t_R 8.42 min (Major) and t_R = 10.38 min (Minor).

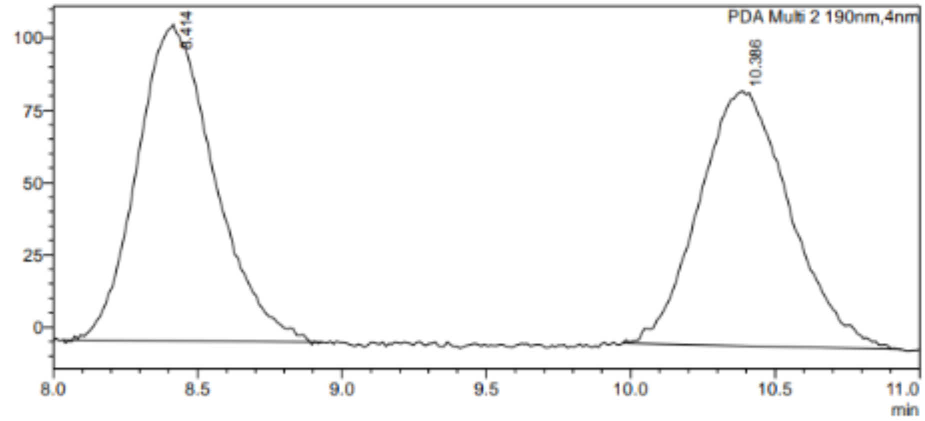

PDA Ch2 190nm

Peak#	Ret. Time	Area	Height	Unit	Mark	Name	Area%
1	8.414	2035722	109385		M		51.310
2	10.386	1931750	88024		M		48.690
Total		3967472	197409				100.000

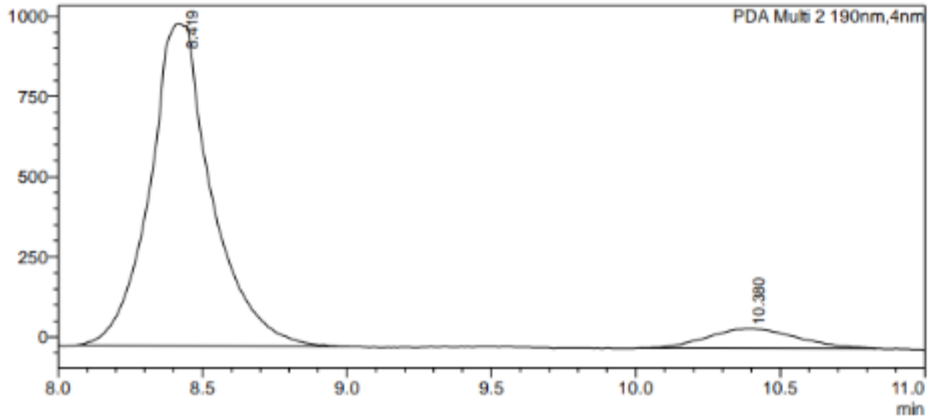

PDA Ch2 190nm

Peak#	Ret. Time	Area	Height	Unit	Mark	Name	Area%
1	8.419	14998496	1003155		M		91.972
2	10.380	1309114	61123		M		8.028
Total		16307610	1064278				100.000

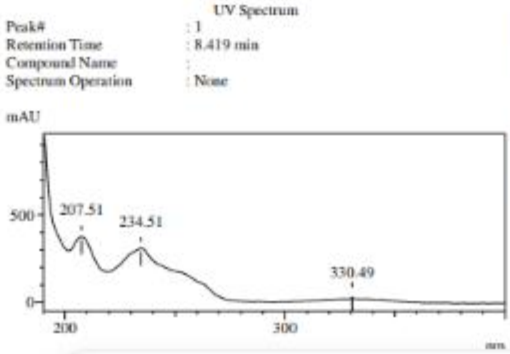

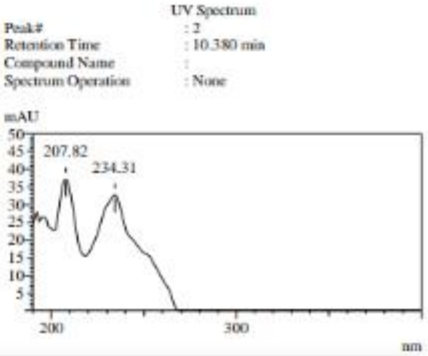

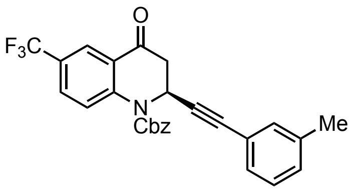

Benzyl (*S*)- 4-oxo-2-(*m*-tolylethynyl)-6-(trifluoromethyl)-3,4-dihydroquinoline-1(2H)-carboxylate (3s).[1]

UPLC analysis (CHIRALPAK OD, 90% heptane and 10% isopropanol, 1 mL/min) t_R 8.69 min (Major) and t_R = 10.51 min (Minor).

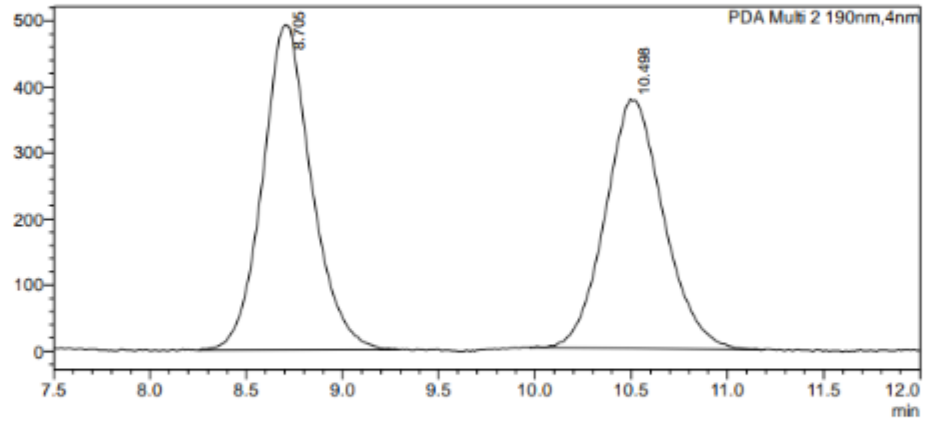

PDA Ch2 190nm

Peak#	Ret. Time	Area	Height	Unit	Mark	Name	Area%
1	8.705	8740965	491917		M		52.277
2	10.498	7979361	377829		M		47.723
Total		16720326	869746				100.000

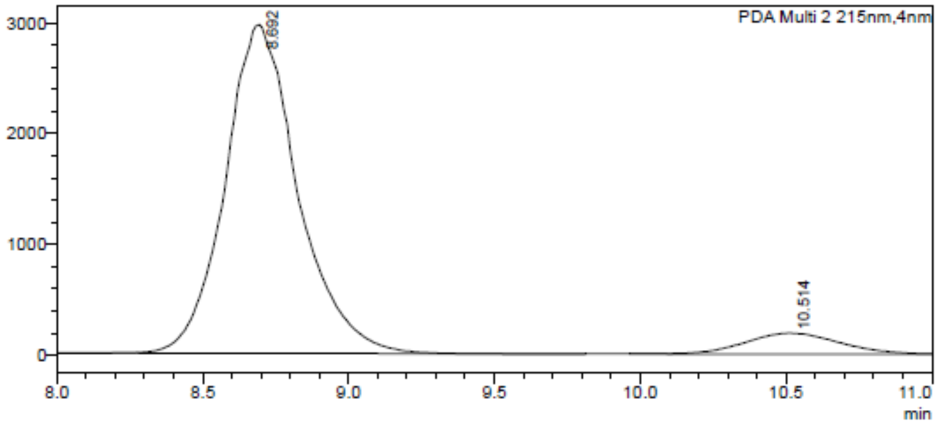

PDA Ch2 215nm

Peak#	Ret. Time	Area	Height	Conc.	Unit	Mark	Area%
1	8.692	51484845	2966058	0.000		M	92.238
2	10.514	4332482	189264	0.000		M	7.762
Total		55817327	3155322				100.000

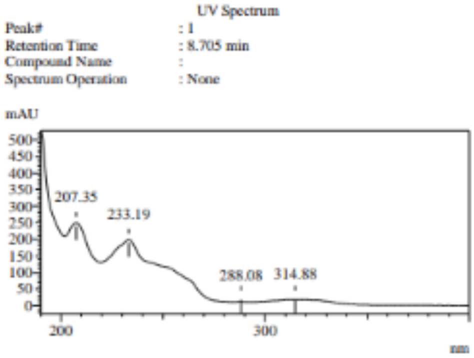

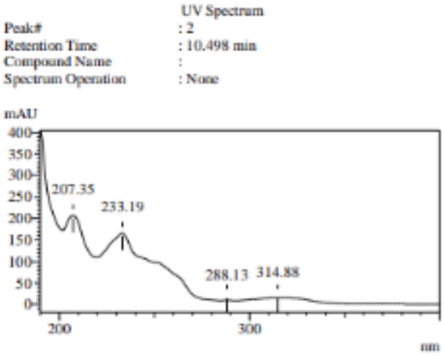

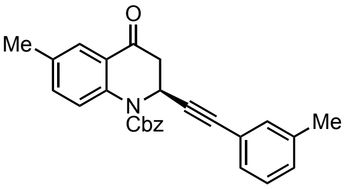

Benzyl (*S*)-6-methyl-4-oxo-2-(m-tolylethynyl)-3,4-dihydroquinoline-1(2H)-carboxylate (3t).[1]

UPLC analysis (CHIRALPAK OD, 90% heptane and 10% isopropanol, 1 mL/min) t_R 8.11 min (Major) and t_R = 10.06 min (Minor).

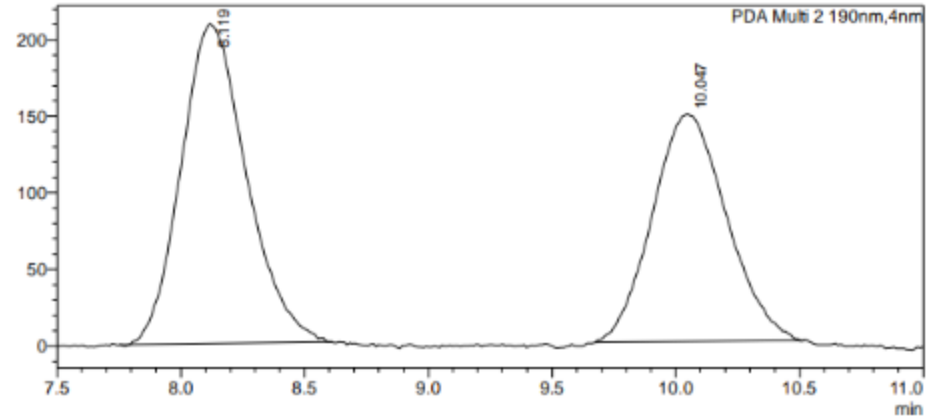

PDA Ch2 190nm

Peak#	Ret. Time	Area	Height	Unit	Mark	Name	Area%
1	8.119	3830760	208509		M		55.239
2	10.047	3104062	148579		M		44.761
Total		6934822	357089				100.000

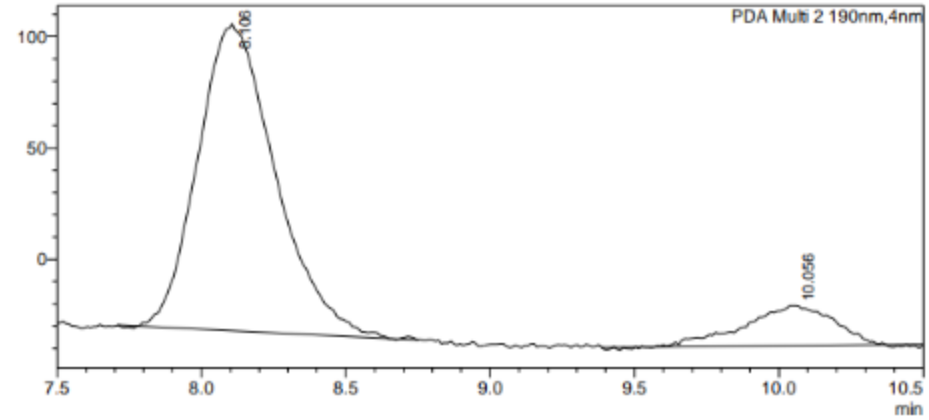

PDA Ch2 190nm

Peak#	Ret. Time	Area	Height	Unit	Mark	Name	Area%
1	8.106	2564839	137596		M		87.126
2	10.056	378984	17845		M		12.874
Total		2943823	155441				100.000

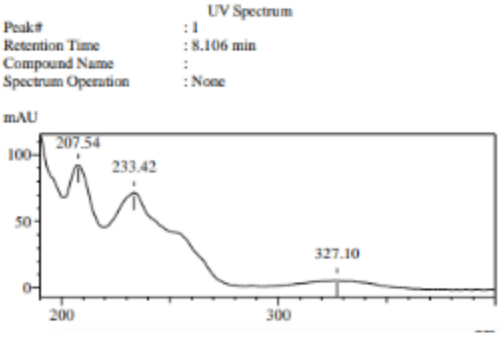

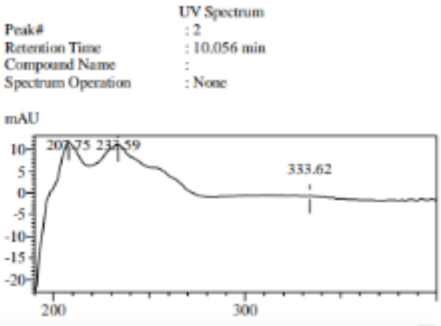

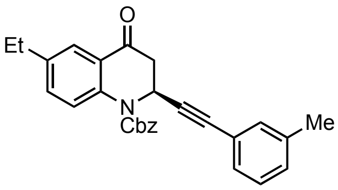

Benzyl (*S*)-6-ethyl-4-oxo-2-(*m*-tolylethynyl)-3,4-dihydroquinoline-1(2H)-carboxylate (3u).[1]

UPLC analysis (CHIRALPAK OD, 90% heptane and 10% isopropanol, 1 mL/min) t_R 7.80 min (Major) and t_R = 9.07 min (Minor).

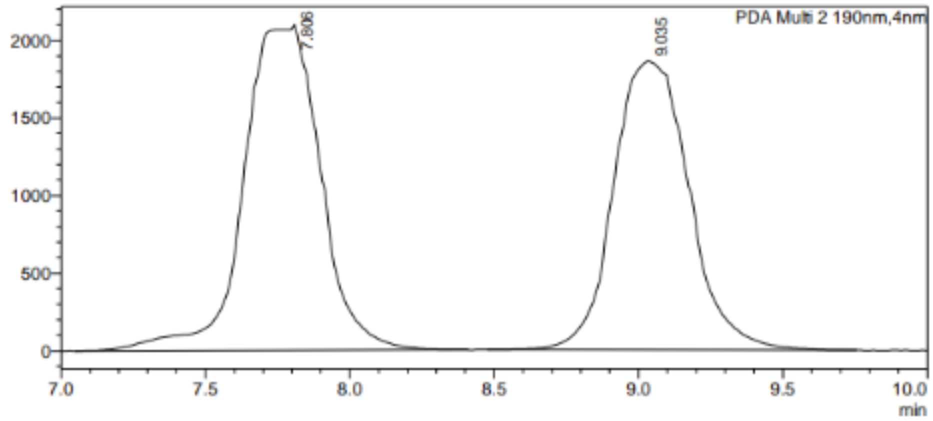

PDA Ch2 190nm

Peak#	Ret. Time	Area	Height	Unit	Mark	Name	Area%
1	7.806	38665018	2094954		M		53.259
2	9.035	33932585	1861036		M		46.741
Total		72597603	3955990				100.000

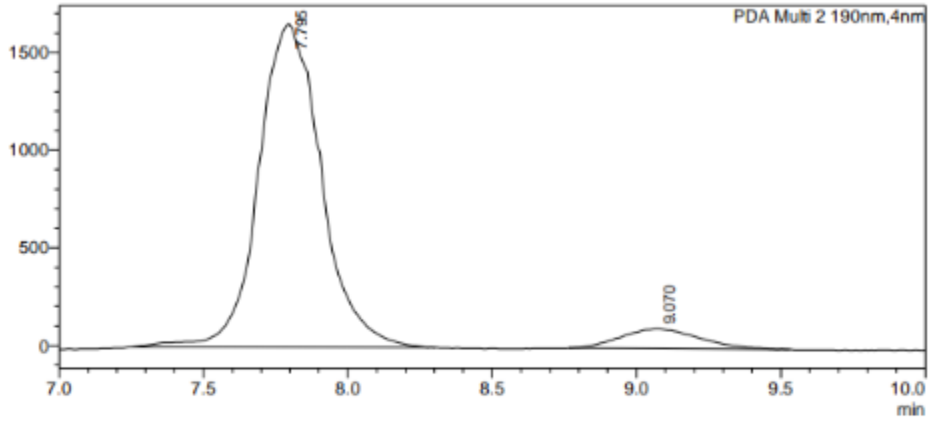

PDA Ch2 190nm

Peak#	Ret. Time	Area	Height	Unit	Mark	Name	Area%
1	7.795	25004659	1654253		M		92.880
2	9.070	1916795	100821		M		7.120
Total		26921454	1755074				100.000

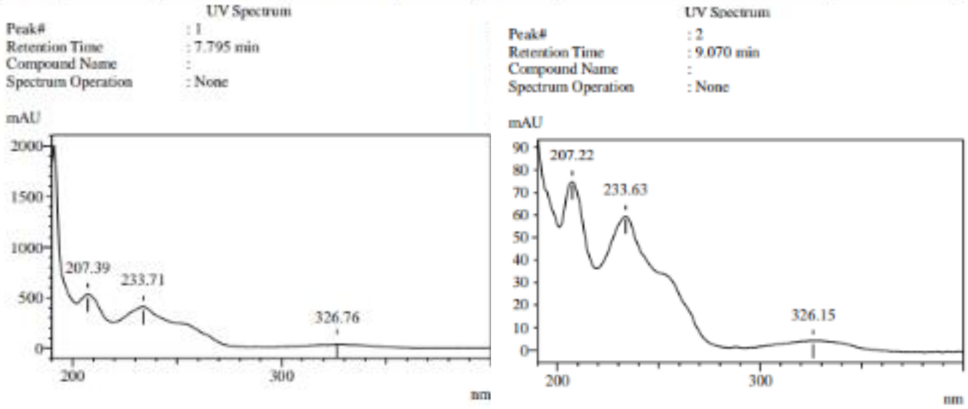

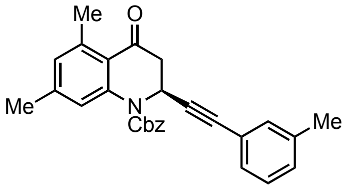

Benzyl (*S*)-5,7-dimethyl-4-oxo-2-(*m*-tolylethynyl)-3,4-dihydroquinoline-1(2H)-carboxylate (3v).[1]

UPLC analysis (CHIRACEL AD, 90% heptane and 10% isopropanol, 1mL/min) t_R = 7.0 min (Minor) and t_R = 9.4 min (Major).

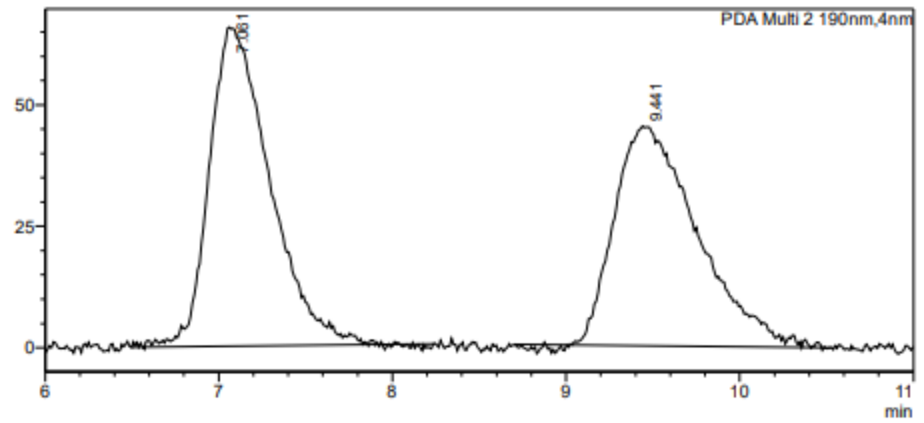

PDA Ch2 190nm

Peak#	Ret. Time	Area	Height	Conc.	Unit	Mark	Area%
1	7.061	1587419	65643	0.000		M	51.523
2	9.441	1493584	45247	0.000		M	48.477
Total		3081003	110891				100.000

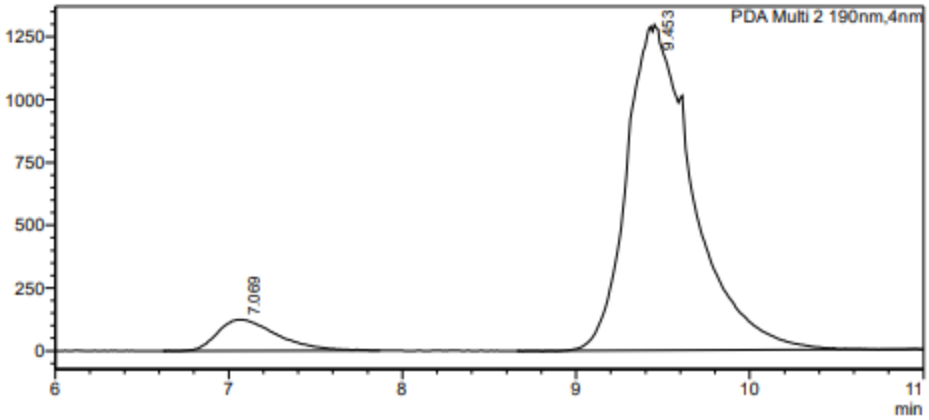

PDA Ch2 190nm

Peak#	Ret. Time	Area	Height	Conc.	Unit	Mark	Area%
1	7.069	2951435	123833	0.000		M	7.883
2	9.453	34487907	1296264	0.000		M	92.117
Total		37439342	1420097				100.000

UV Spectrum

Peak# : 1
Retention Time : 7.069 min
Compound Name :
Spectrum Operation : None

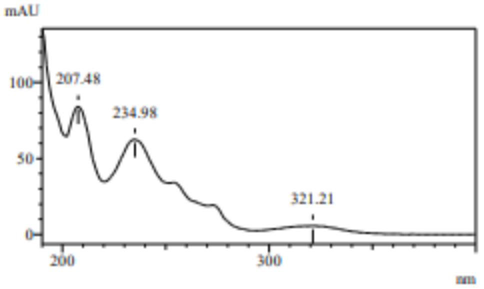

UV Spectrum

Peak# : 2
Retention Time : 9.453 min
Compound Name :
Spectrum Operation : None

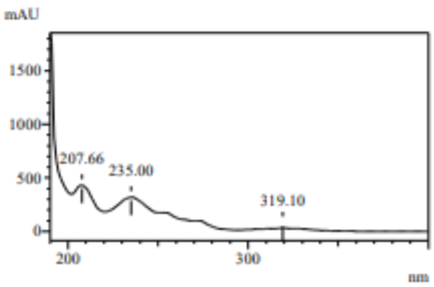

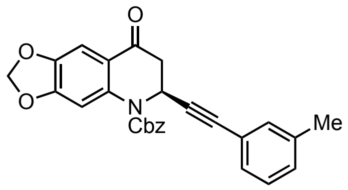

Benzyl (*S*)-8-oxo-6-(*m*-tolylethynyl)-7,8-dihydro-[1,3]dioxolo[4,5-g]quinoline-5(6H)-carboxylate (3w).[1]

UPLC analysis (CHIRACEL AD, 90% heptane and 10% isopropanol, 1mL/min) t_R = 12.73 min (Major) and t_R = 17.30 min (Minor).

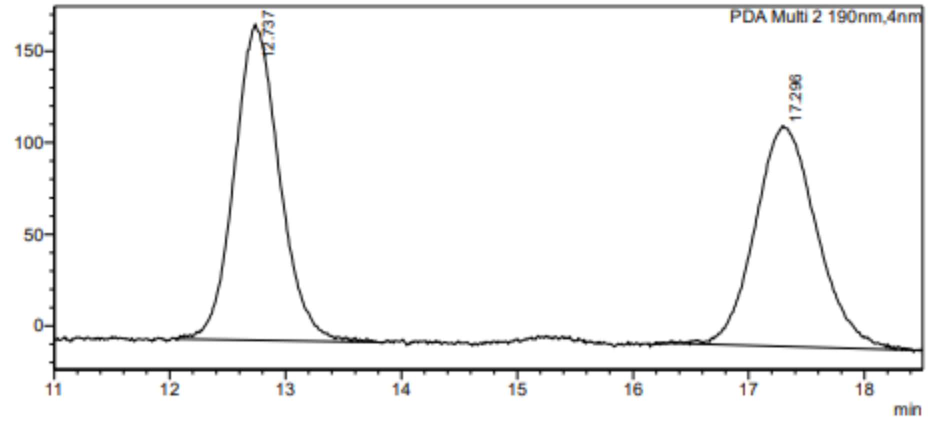

PDA Ch2 190nm

Peak#	Ret. Time	Area	Height	Conc.	Unit	Mark	Area%
1	12.737	4686521	172410	0.000		M	51.310
2	17.296	4447264	120328	0.000		M	48.690
Total		9133785	292738				100.000

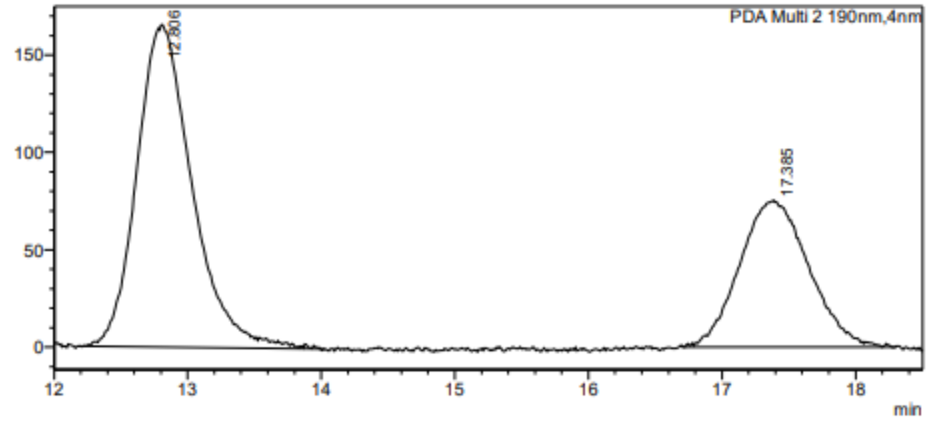

PDA Ch2 190nm

Peak#	Ret. Time	Area	Height	Conc.	Unit	Mark	Area%
1	12.806	4759381	165474	0.000		M	63.785
2	17.385	2702227	75165	0.000		M	36.215
Total		7461608	240639				100.000

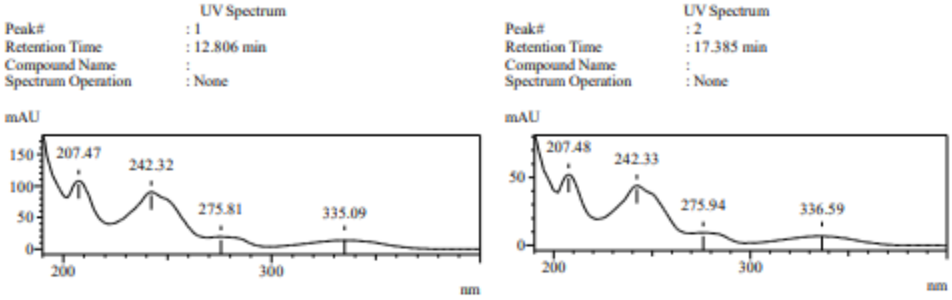

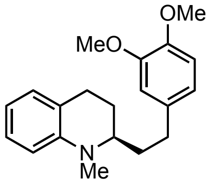

(+)-Cuspareine (4).[9] *Step 1:* Synthesised as per general procedure **C** on a 1 mmol scale using benzyl 4-oxoquinoline-1(4H)-carboxylate **1a** and 4-ethynyl-1,2-dimethoxybenzene. The product was isolated as a yellow solid (0.254 g, 56%, 77% *ee*). This product was then recrystallised using hot ethanol (94% *ee*)

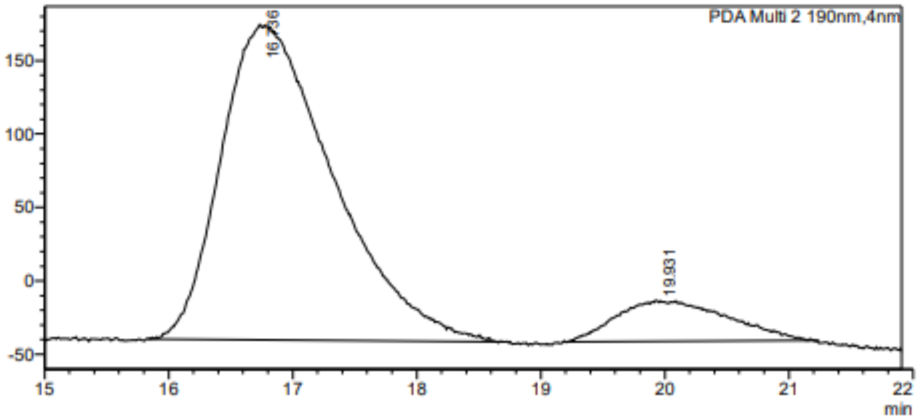

PDA Ch2 190nm

Peak#	Ret. Time	Area	Height	Conc.	Unit	Mark	Area%
1	16.736	13494151	214542	0.000			88.644
2	19.931	1728636	27832	0.000		M	11.356
Total		15222786	242374				100.000

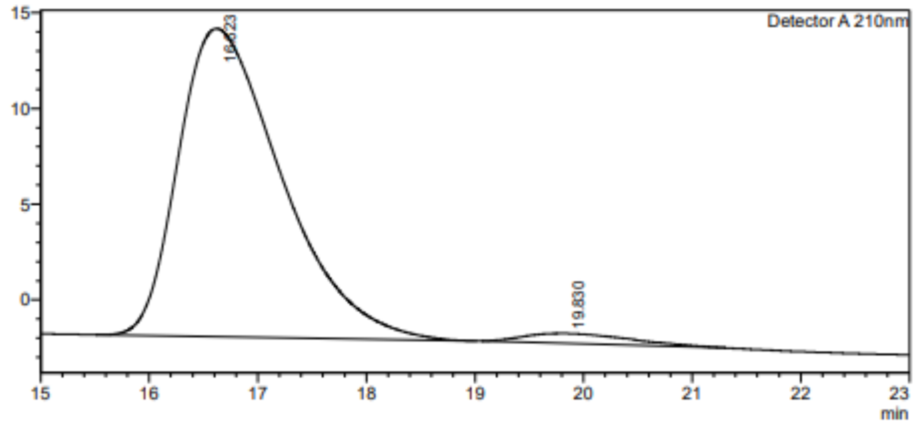

Peak#	Ret. Time	Area	Height	Conc.	Unit	Mark	Name
1	16.623	1063835	16099	96.781		M	
2	19.830	35387	519	3.219		M	
Total		1099222	16618				

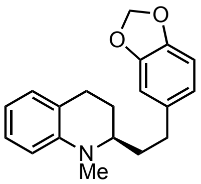

(−)-Galipinine (5).[9]

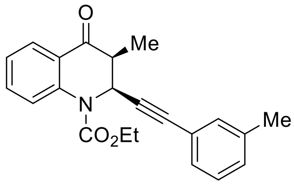

Benzyl (2S,3S)-3-methyl-4-oxo-2-(m-tolylethynyl)-3,4-dihydroquinoline-1(2H)-carboxylate (8).

UPLC analysis (CHIRACEL OD, 95% heptane and 5% isopropanol, 0.5 mL/min) t_R = 12.02 min (Major) and t_R = 13.16 min (Minor)

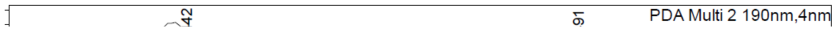

PDA Ch2 190nm

Peak#	Ret. Time	Area	Height	Unit	Mark	Name	Area%
1	12.018	13230505	755213		M		62.684
2	13.162	7876001	352464		M		37.316
Total		21106506	1107677				100.000

UV Spectrum

Peak# : 1
Retention Time : 12.018 min
Compound Name :
Spectrum Operation : None

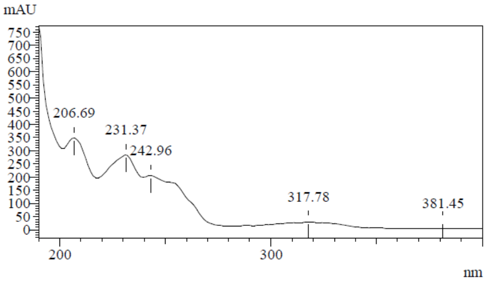

UV Spectrum

Peak# : 2
Retention Time : 13.162 min
Compound Name :
Spectrum Operation : None

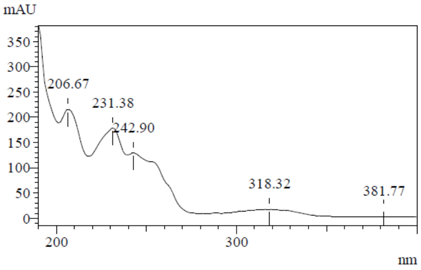

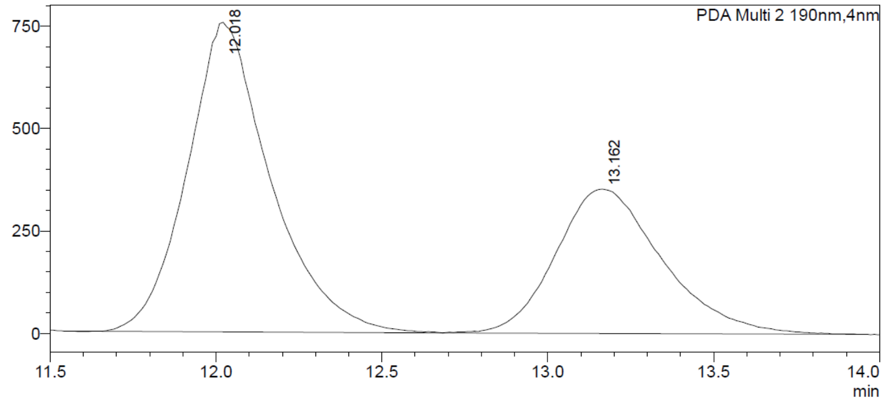

6. References

(1) Maestro, A.; Lemaire, S.; Harutyunyan, S. R. Cu(I)-Catalyzed Alkynylation of Quinolones. *Org. Lett.* **2022**, *24* (5), 1228–1231.

(2) Shintani, R.; Tokunaga, N.; Doi, H.; Hayashi, T. A New Entry of Nucleophiles in Rhodium-Catalyzed Asymmetric 1,4-Addition Reactions: Addition of Organozinc Reagents for the Synthesis of 2-Aryl-4-Piperidones. *J. Am. Chem. Soc.* **2004**, *126* (20), 6240–6241.

(3) Holtschulte, C.; Börgel, F.; Westphälinger, S.; Schepmann, D.; Civenni, G.; Laurini, E.; Marson, D.; Catapano, C. V.; Pricl, S.; Wünsch, B. Synthesis of Aminoethyl-Substituted Piperidine Derivatives as Σ1 Receptor Ligands with Antiproliferative Properties. *ChemMedChem* **2022**, *17* (7), 1–17.

(4) Bichovski, P.; Haas, T. M.; Kratzert, D.; Streuff, J. Synthesis of Bridged Benzazocines and Benzoxocines by a Titanium-Catalyzed Double-Reductive Umpolung Strategy. *Chem. - A Eur. J.* **2015**, *21* (6), 2339–2342.

(5) Chakraborty, B.; Dutta, D.; Mukherjee, S.; Das, S.; Maiti, N. C.; Das, P.; Chowdhury, C. Synthesis and Biological Evaluation of a Novel Betulinic Acid Derivative as an Inducer of Apoptosis in Human Colon Carcinoma Cells (HT-29). *Eur. J. Med. Chem.* **2015**, *102*, 93–105.

(6) Wang, S.; Larrosa, I.; Yorimitsu, H.; Perry, G. J. P. Carboxylic Acid Salts as Dual-Function Reagents for Carboxylation and Carbon Isotope Labeling. *Angew. Chemie - Int. Ed.* **2023**, *62* (14), 1–5.

(7) Pearson, W. H.; Postich, M. J. Approach 6a-Epipretazettine 6a-Epiprecriwelline Cycloaddition. *J. Org. Chem.* **1994**, 21, 5662–5671.

(8) Farran, D.; Slawin, A. M. Z.; Kirsch, P.; O'Hagan, D. Diastereoselective Synthesis of 2,3,4,5,6-Pentafluoroheptanes. *J. Org. Chem.* **2009**, *74* (18), 7168–7171.

(9) Davies, S. G.; Fletcher, A. M.; Houlsby, I. T. T.; Roberts, P. M.; Thomson, J. E.; Zimmer, D. The Hancock Alkaloids (-)-Cuspareine, (-)-Galipinine, (-)-Galipeine, and (-)-Angustureine: Asymmetric Syntheses and Corrected 1 H and 13 C NMR Data. *J. Nat. Prod.* **2018**, *81* (12), 2731–2742.

7. NMR Spectra

Benzyl 4-oxoquinoline-1(4H)-carboxylate (1a); ^{1}H NMR (400 MHz, $CDCl_3$)

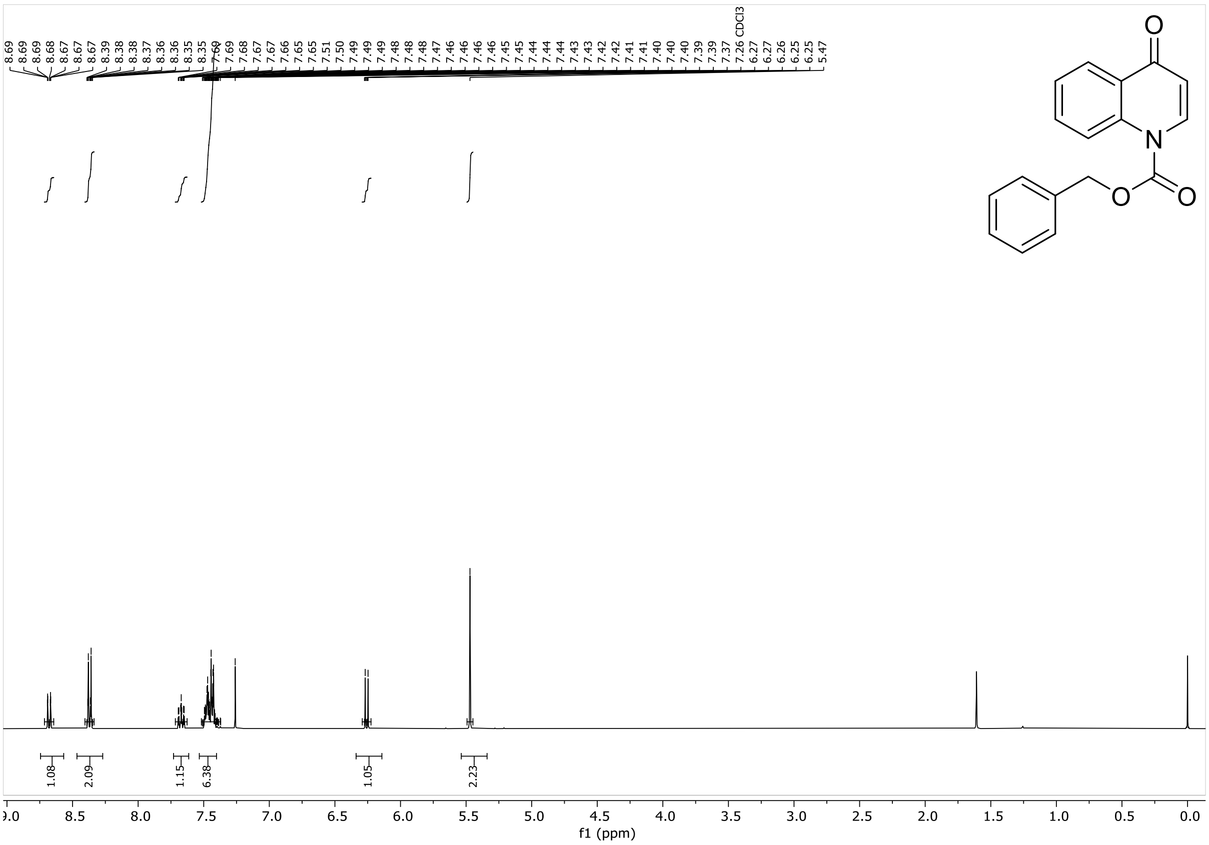

^{13}C{^{1}H} NMR (101 MHz, $CDCl_3$)

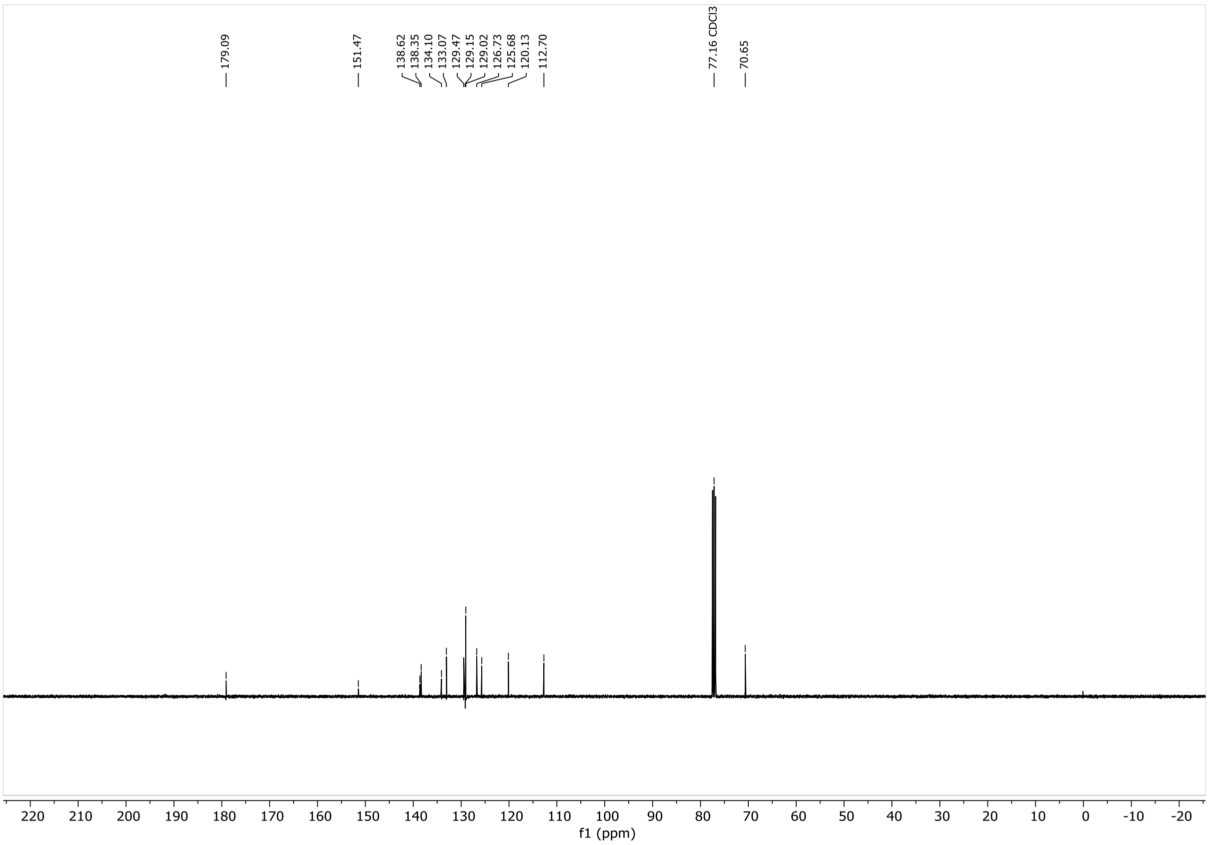

Benzyl 6-methoxy-4-oxoquinoline-1(4H)-carboxylate (1b); ^{1}H NMR (500 MHz, $CDCl_3$)

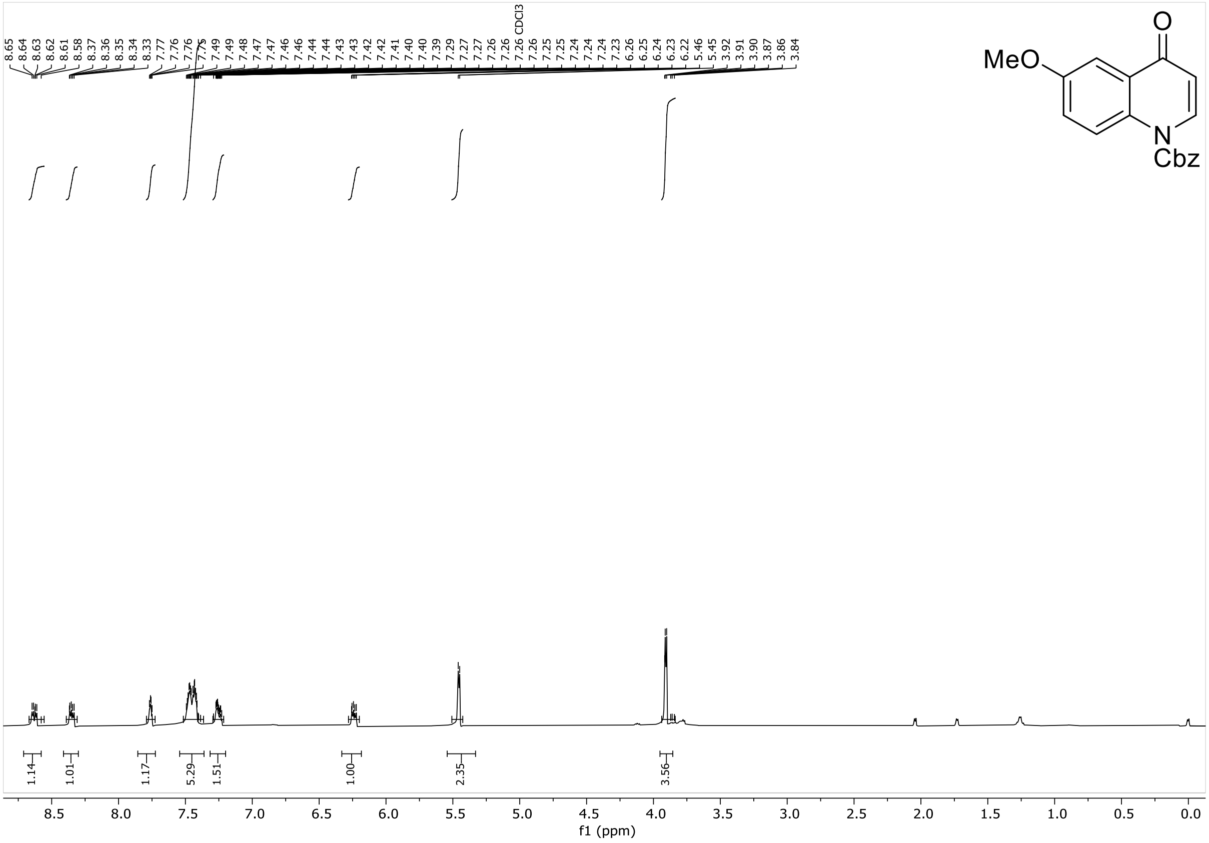

$^{13}C\{^{1}H\}$ NMR (101 MHz, $CDCl_3$)

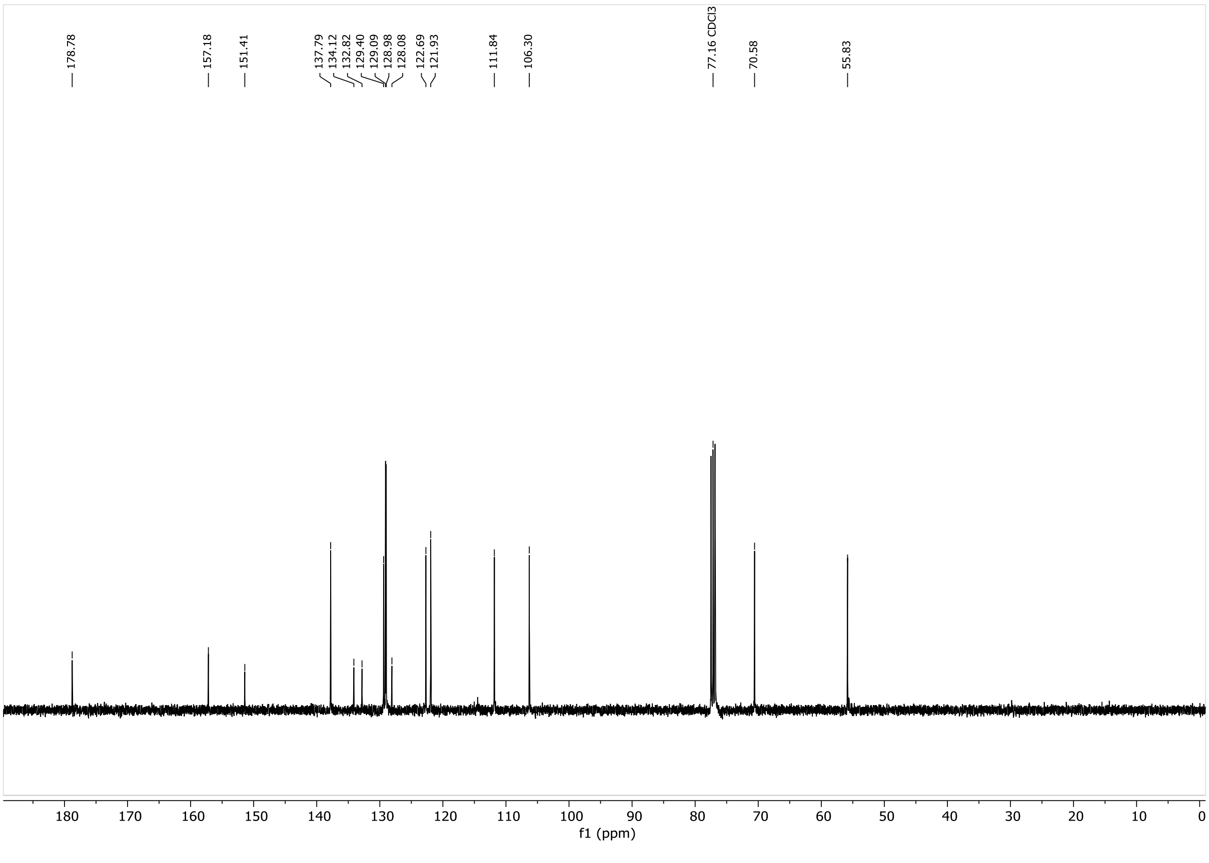

Benzyl 6-chloro-4-oxoquinoline-1(4H)-carboxylate (1c); ^{1}H NMR (400 MHz, $CDCl_3$)

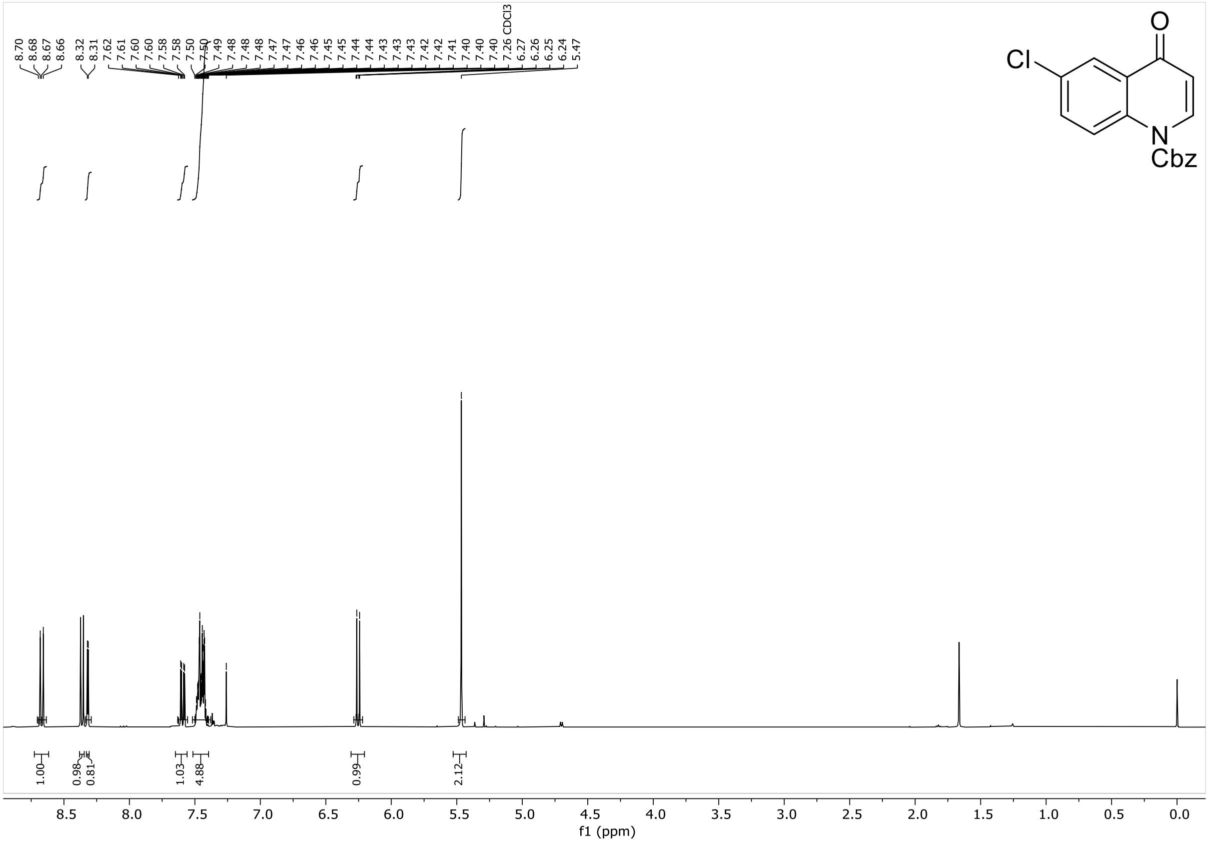

^{13}C{^{1}H} NMR (101 MHz, $CDCl_3$)

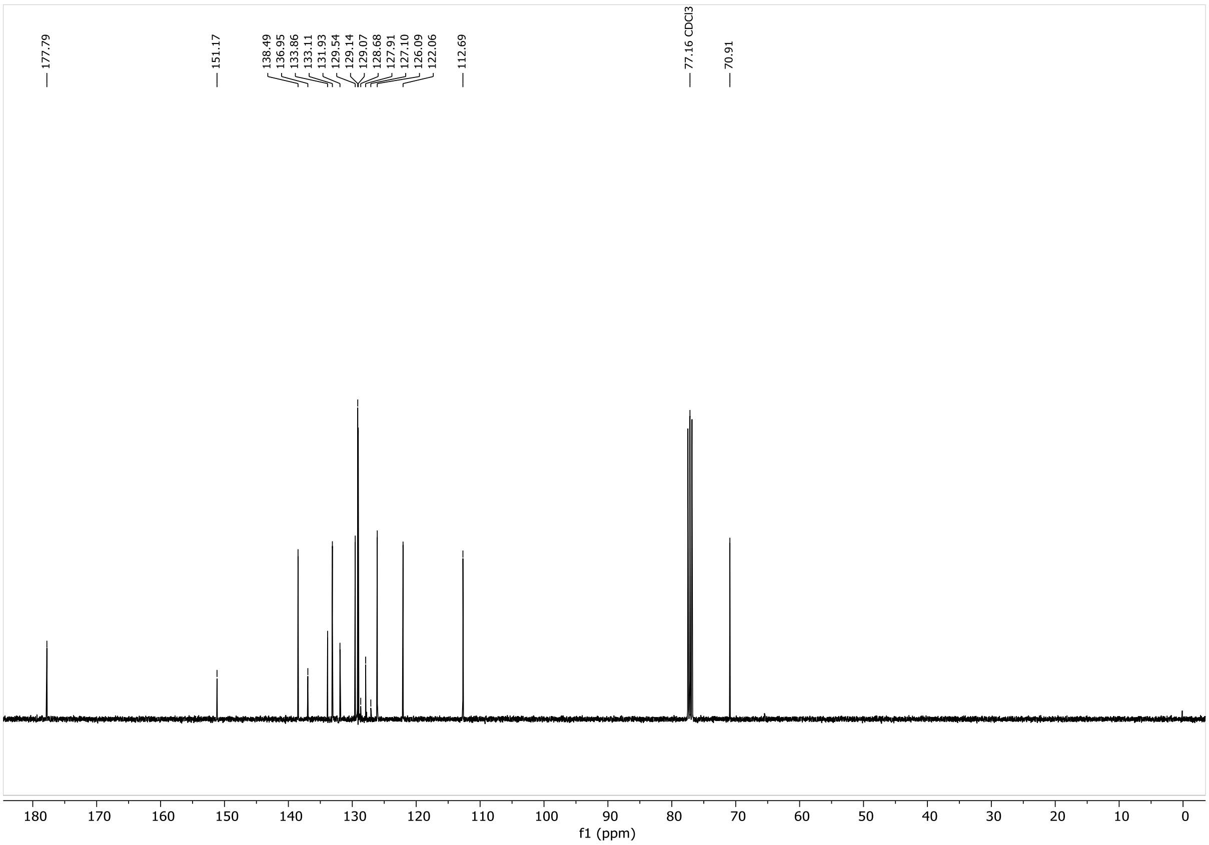

Benzyl 4-oxo-6-(trifluoromethyl)quinoline-1(4H)-carboxylate (1d); ^{1}H NMR (400 MHz, $CDCl_3$)

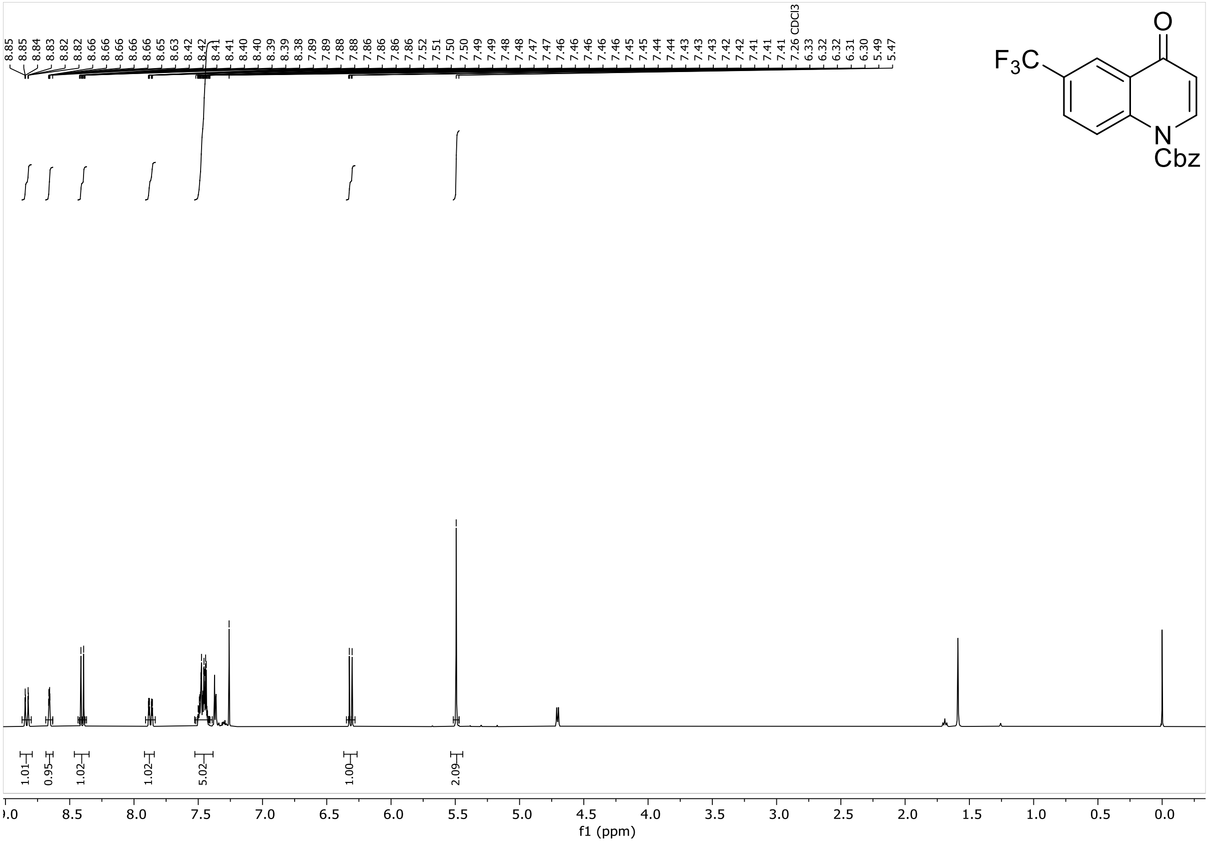

^{13}C{^{1}H} NMR (101 MHz, $CDCl_3$)

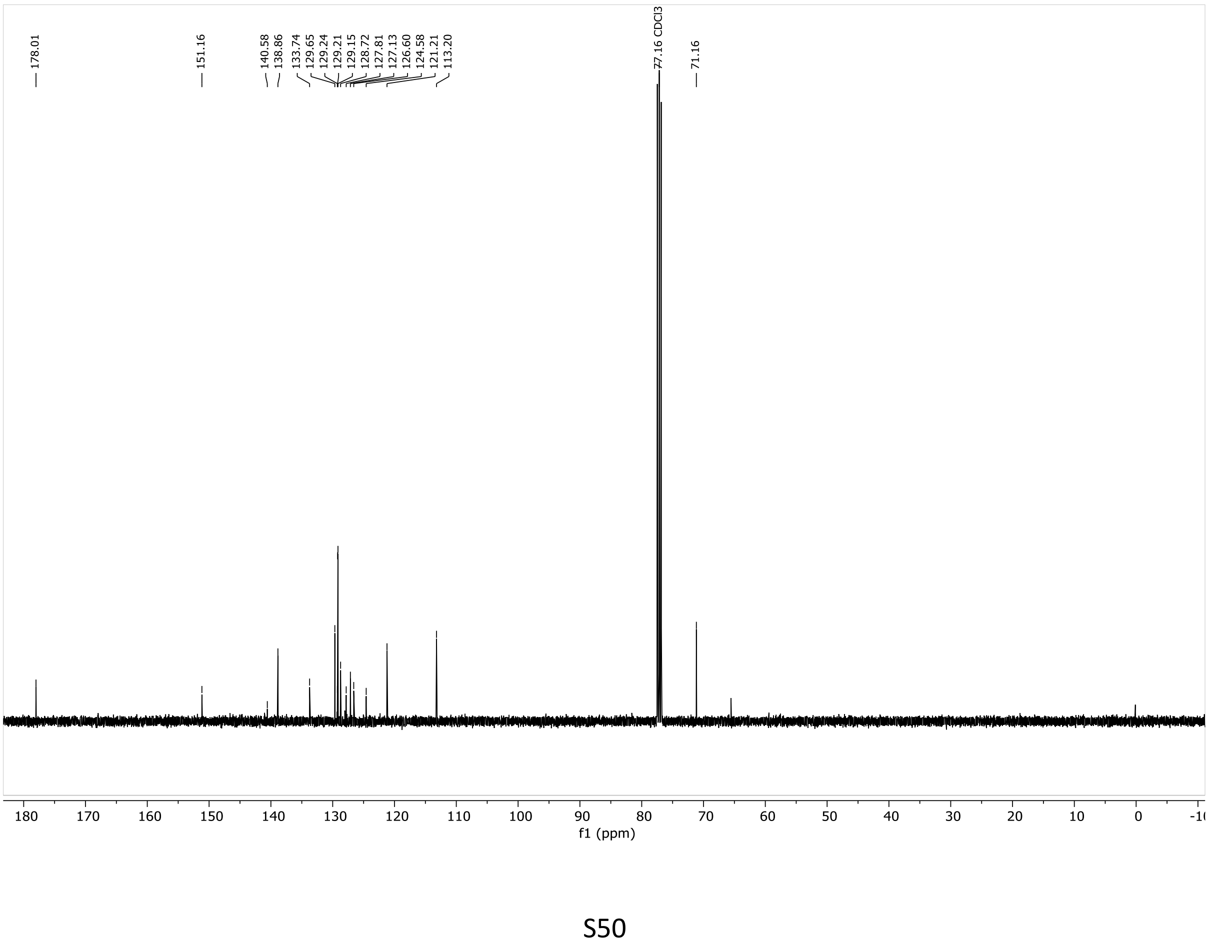

Benzyl 6-methyl-4-oxoquinoline-1(4H)-carboxylate (1e); ^{1}H NMR (400 MHz, $CDCl_3$)

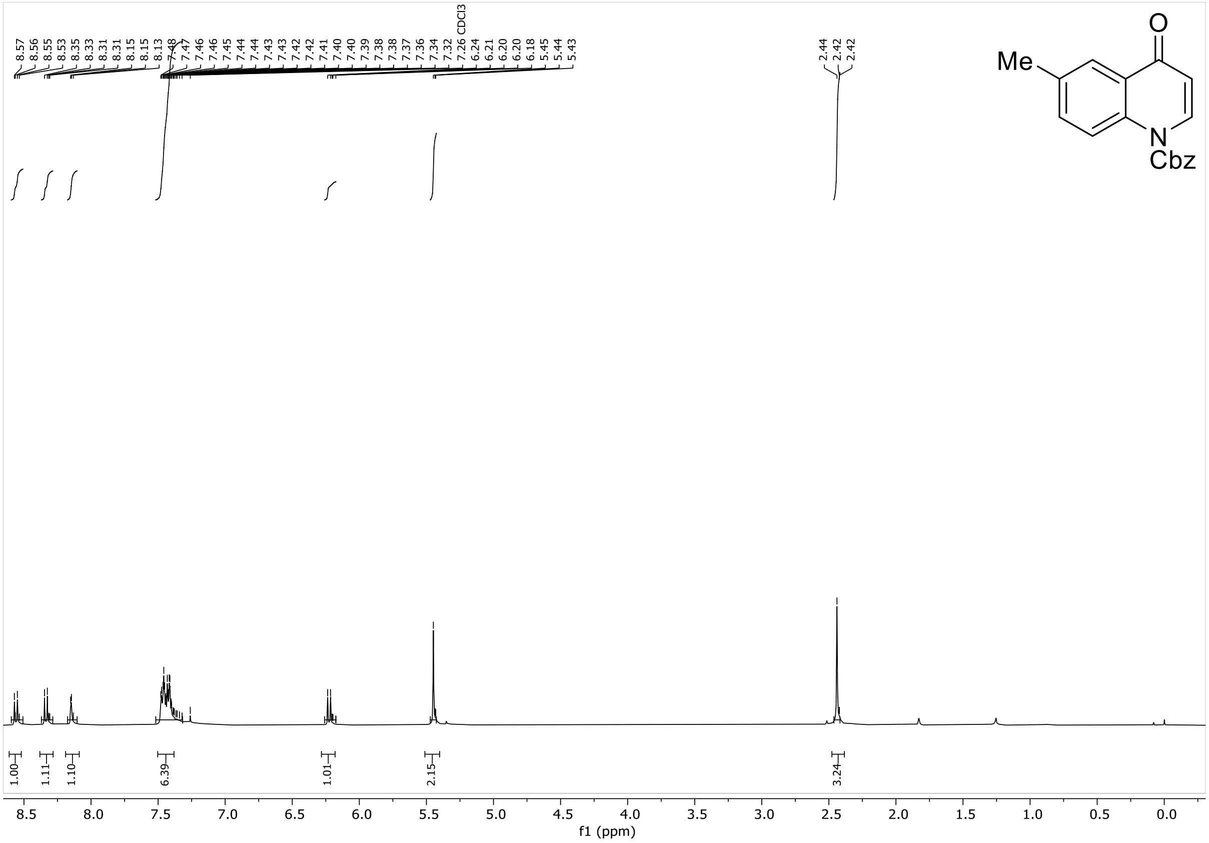

^{13}C{^{1}H} NMR (101 MHz, $CDCl_3$)

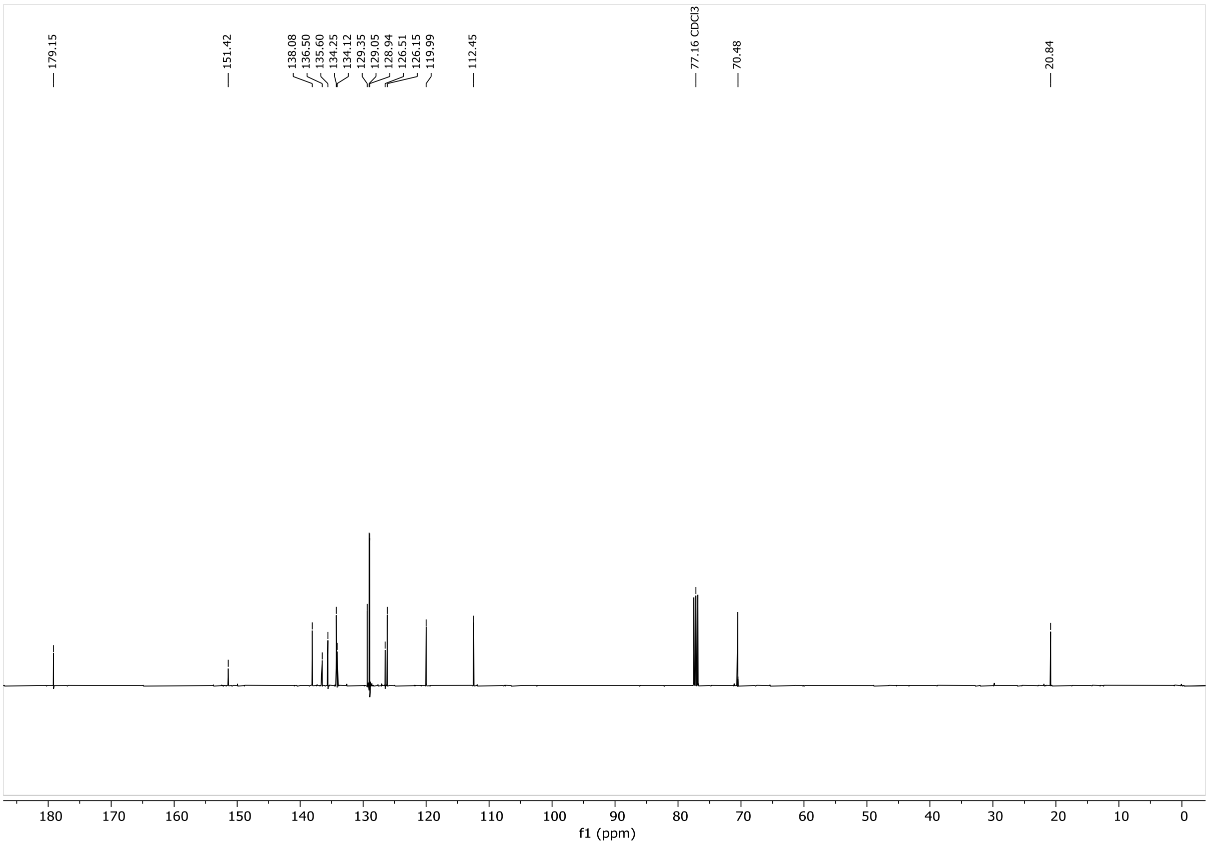

Benzyl 6-ethyl-4-oxoquinoline-1(4H)-carboxylate (1f); ^{1}H NMR (400 MHz, $CDCl_3$)

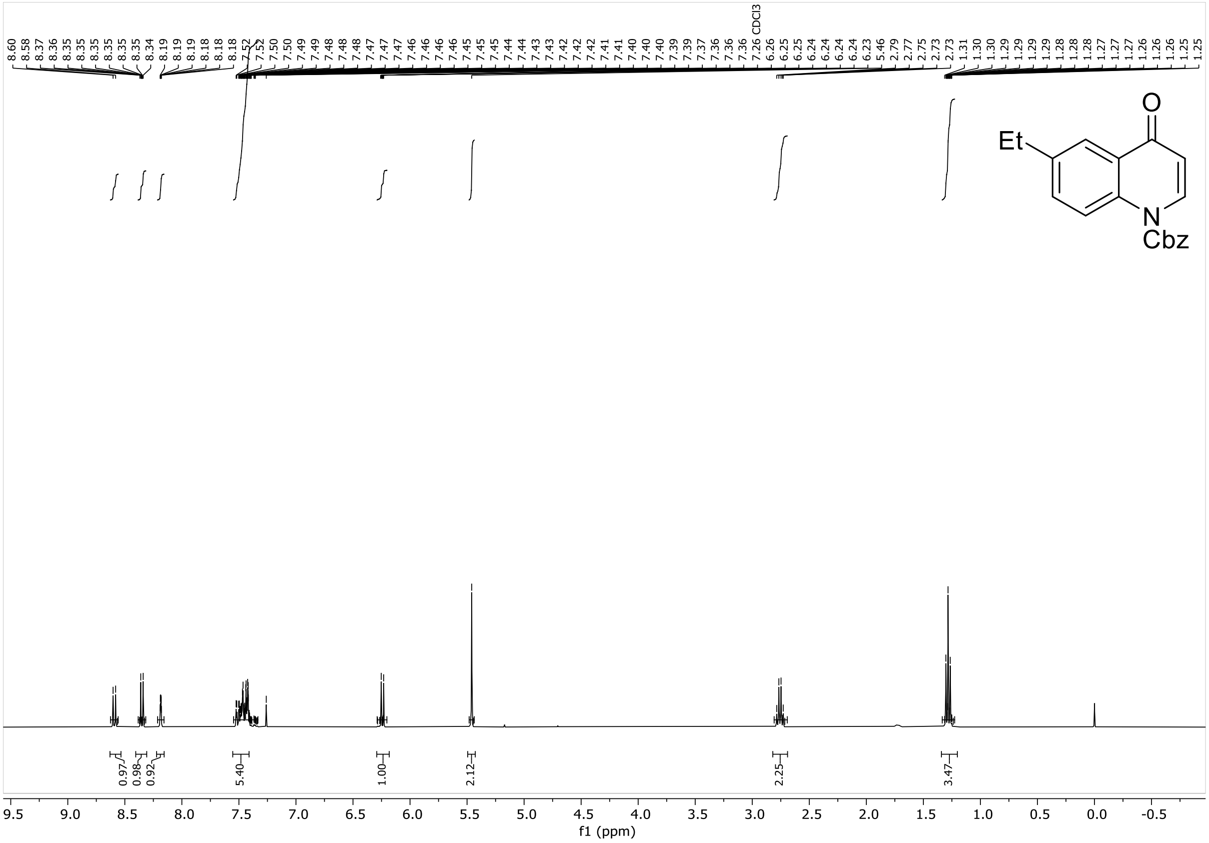

^{13}C{^{1}H} NMR (101 MHz, $CDCl_3$)

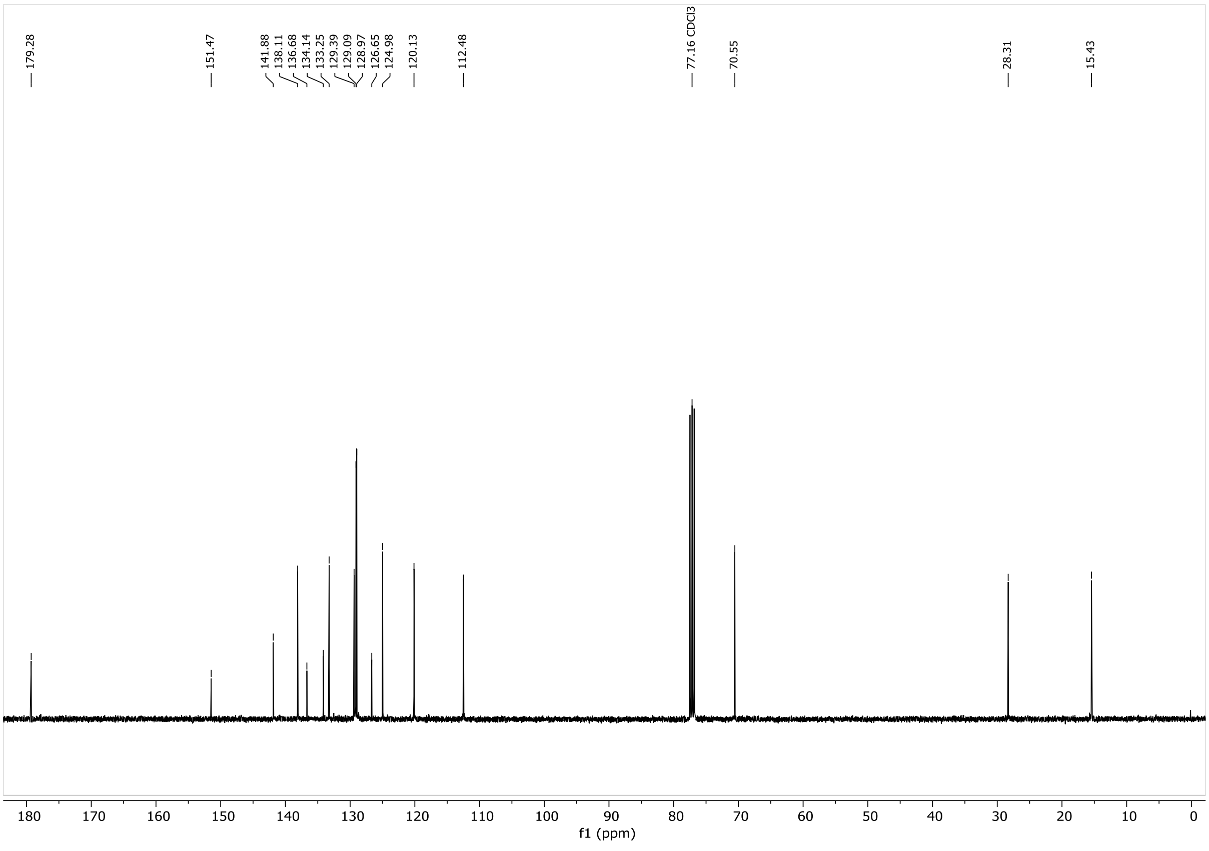

Benzyl 5,7-dimethyl-4-oxoquinoline-1(4H)-carboxylate (1g); ^{1}H NMR (400 MHz, $CDCl_3$)

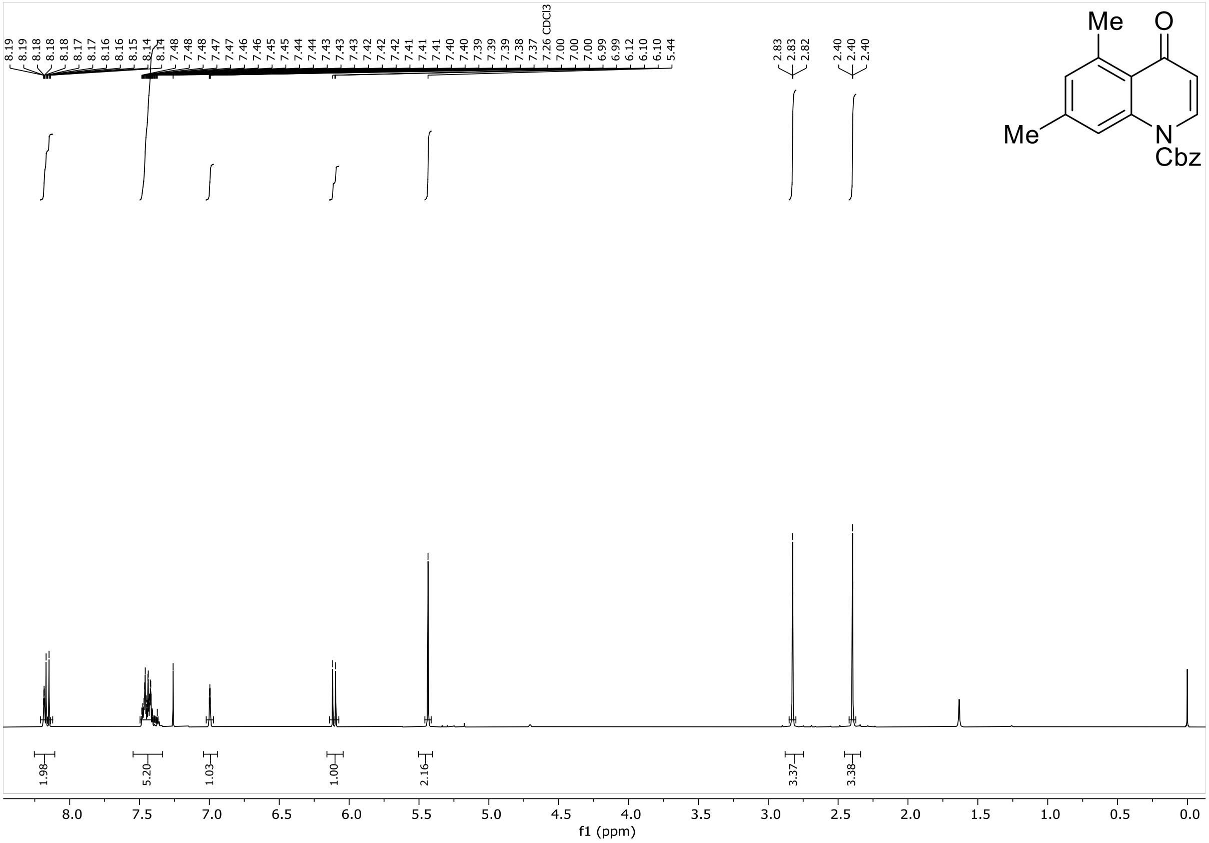

^{13}C{^{1}H} NMR (101 MHz, $CDCl_3$)

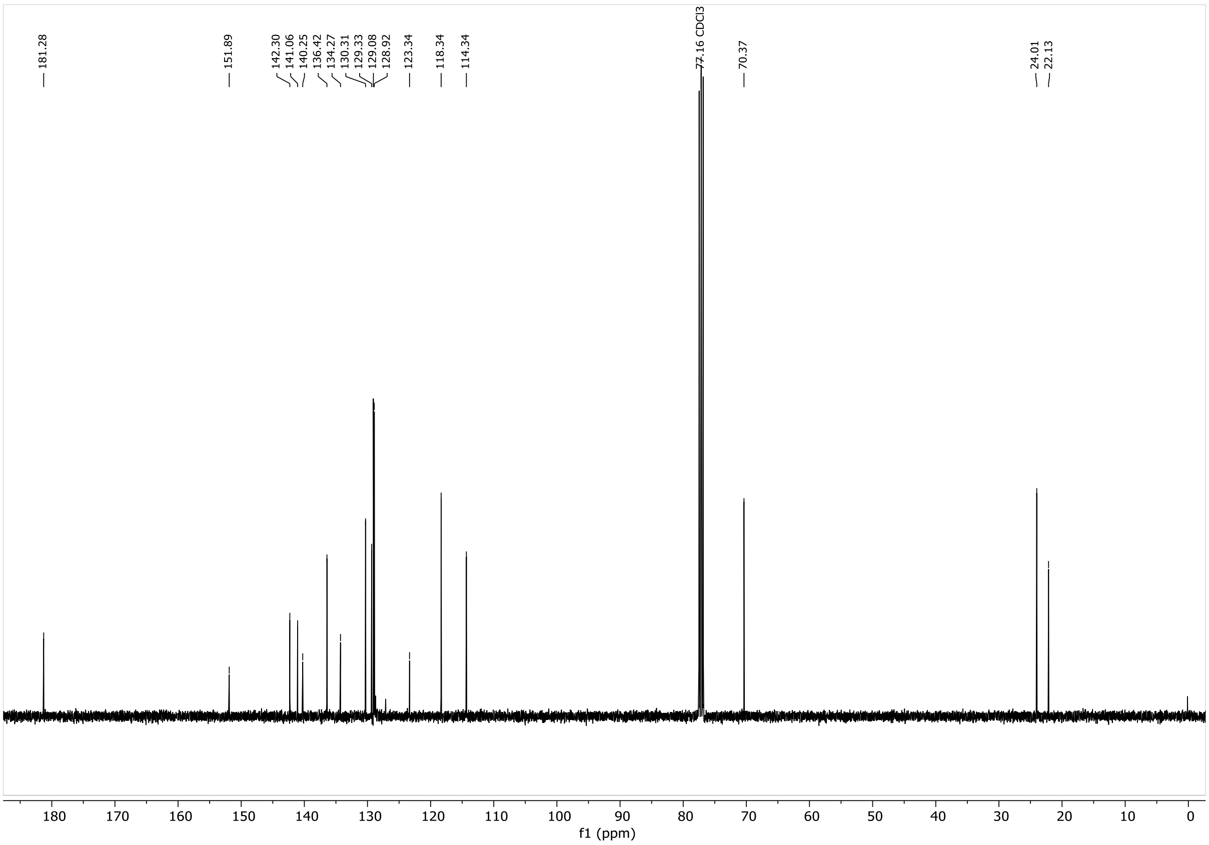

Benzyl 8-oxo-[1,3]dioxolo[4,5-g]quinoline-5(8H)-carboxylate (1h); ^{1}H NMR (400 MHz, $CDCl_3$)

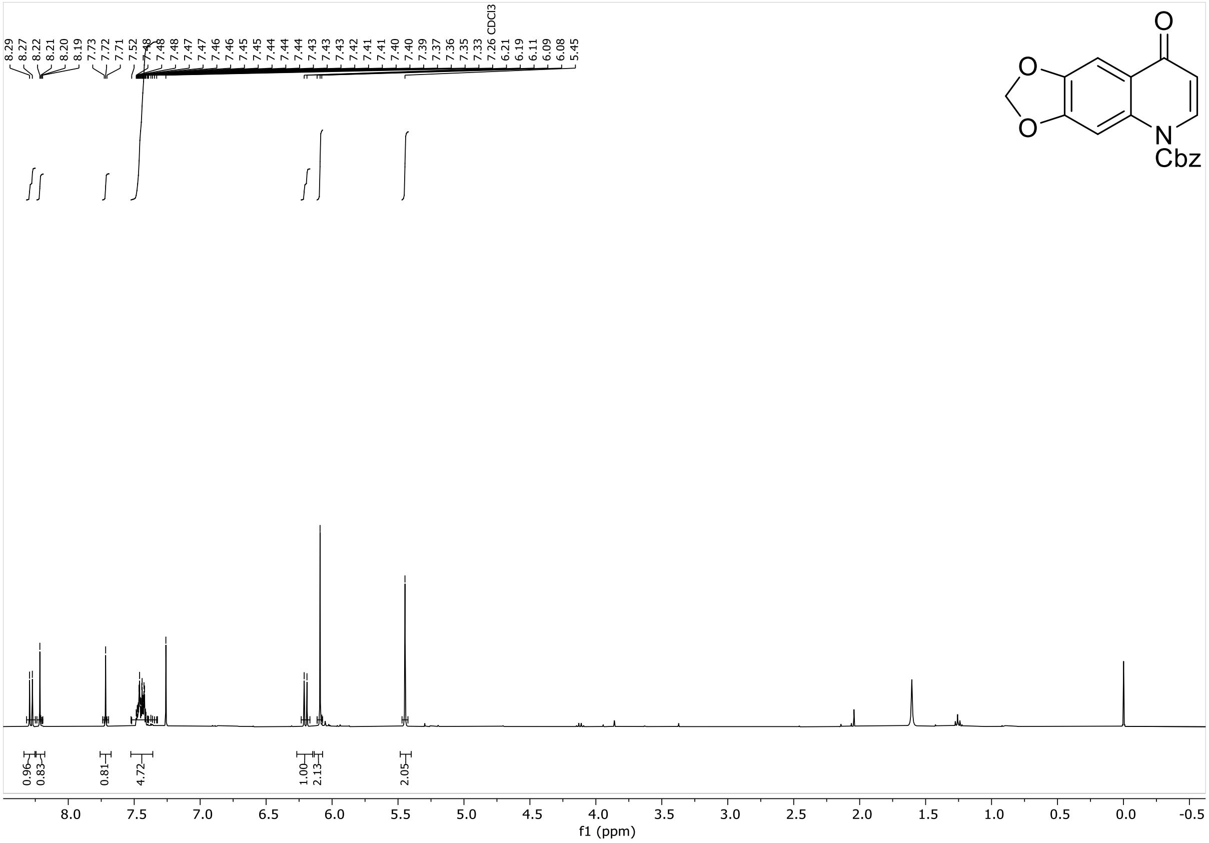

^{13}C{^{1}H} NMR (101 MHz, $CDCl_3$)

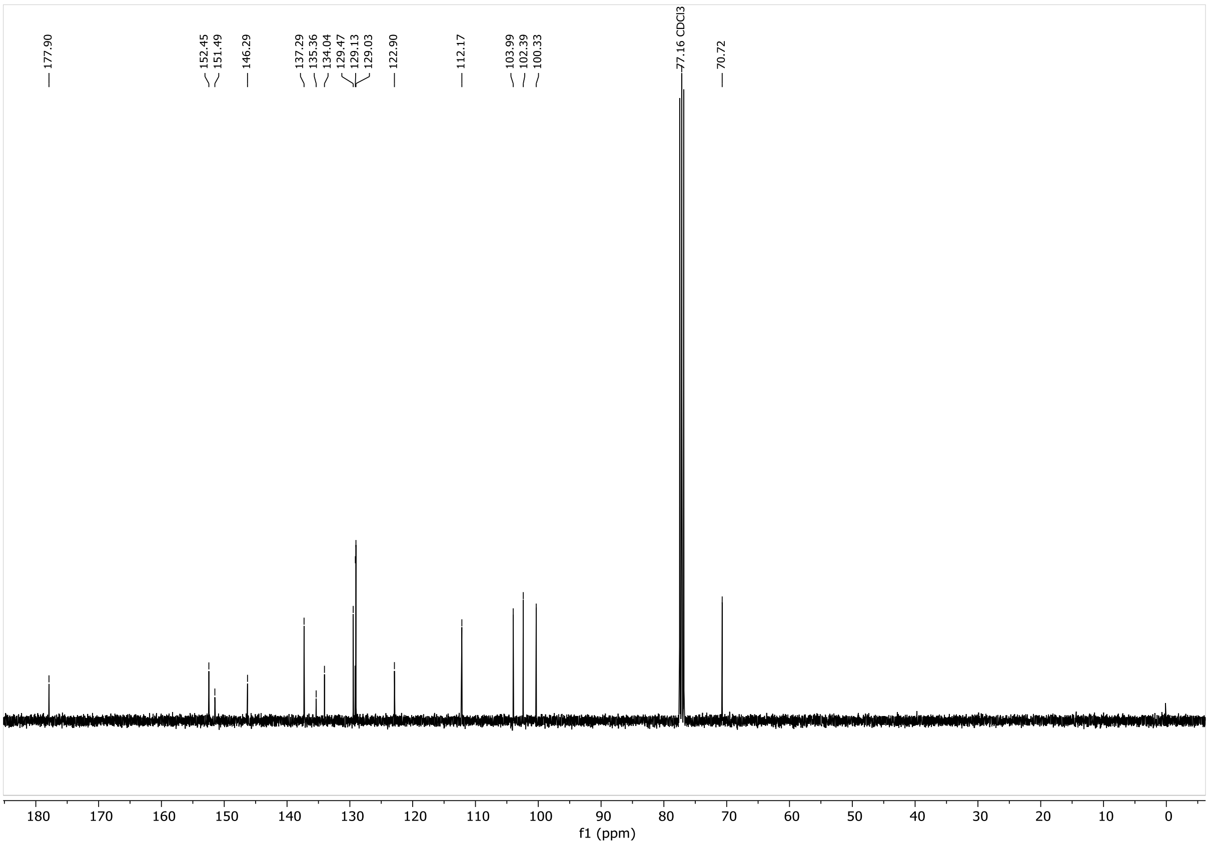

Benzyl 8-bromo-4-oxoquinoline-1(4H)-carboxylate (1i); ^{1}H NMR (400 MHz, $CDCl_3$)

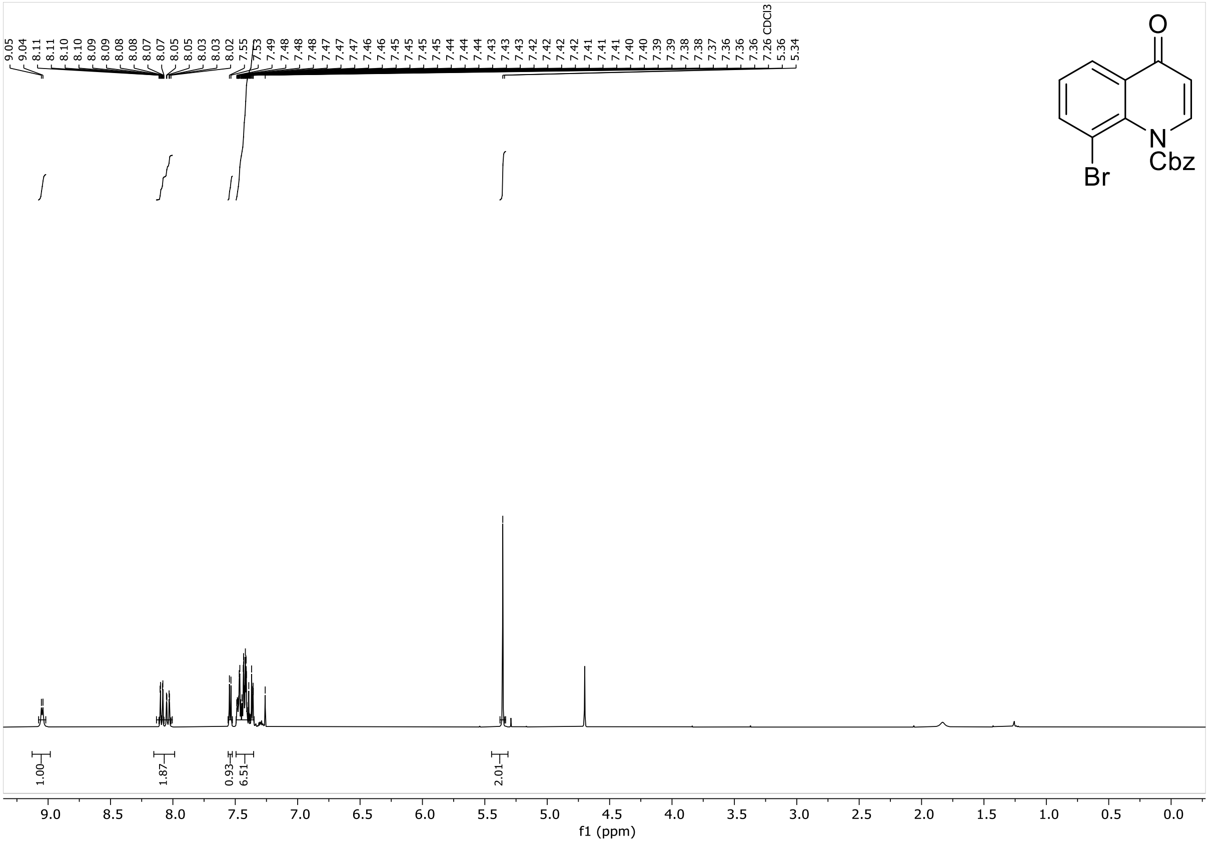

^{13}C{^{1}H} NMR (101 MHz, $CDCl_3$)

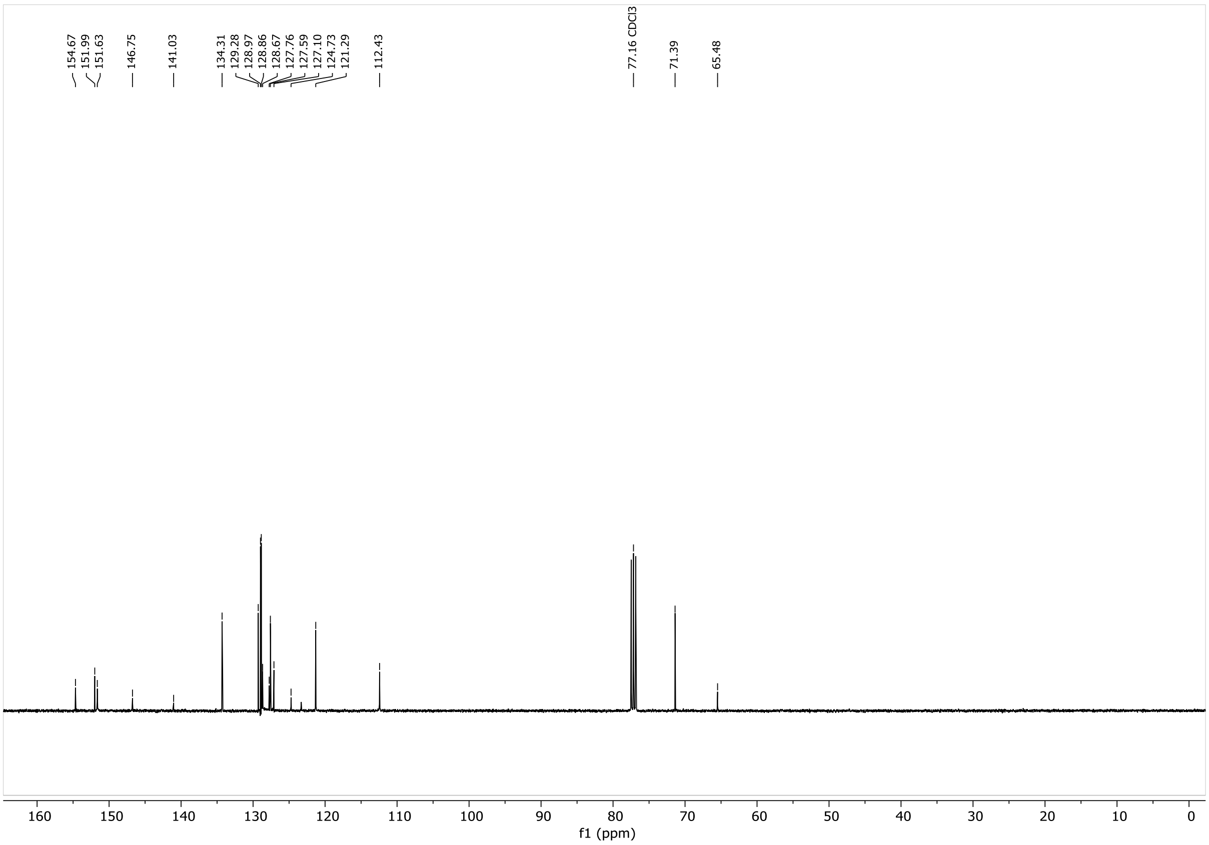

Benzyl 4-oxo-3,4-dihydropyridine-1(2H)-carboxylate (6); ^{1}H NMR (400 MHz, $CDCl_3$)

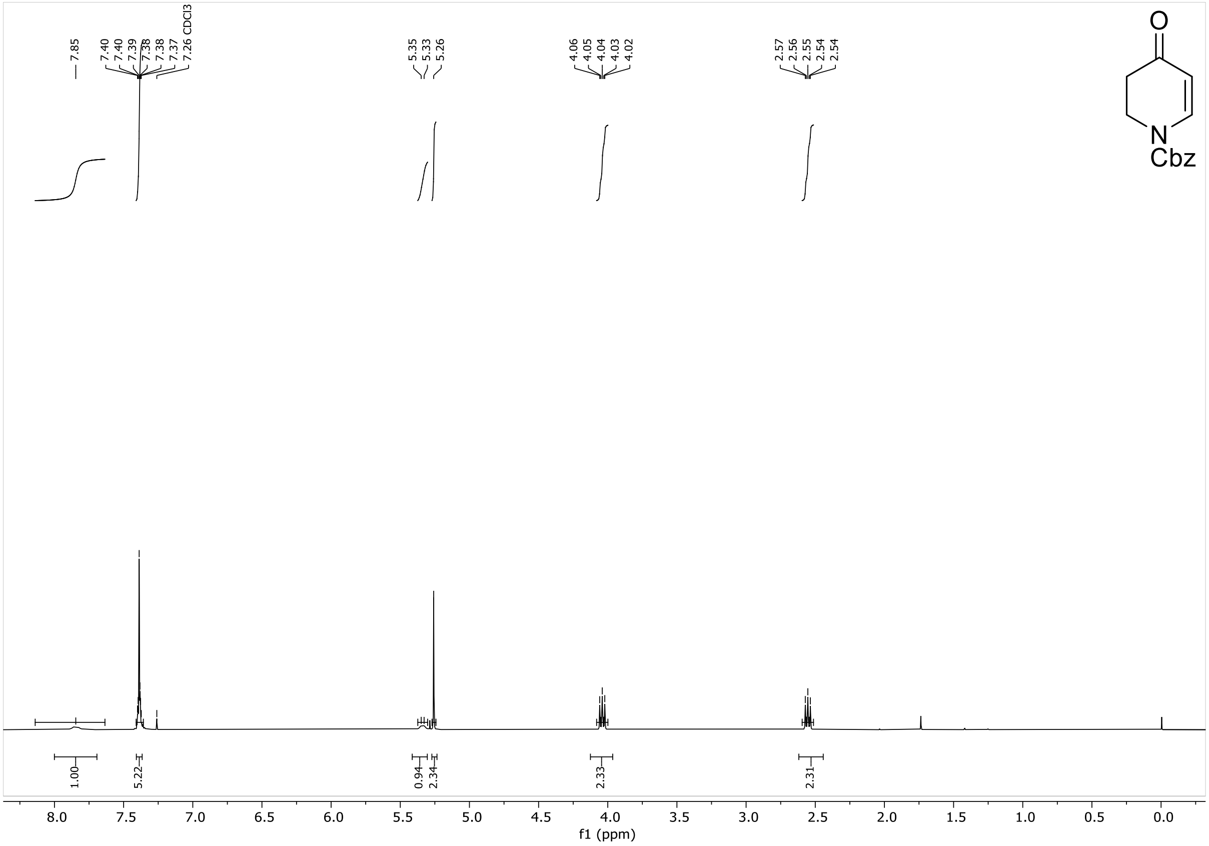

^{13}C{^{1}H} NMR (101 MHz, $CDCl_3$)

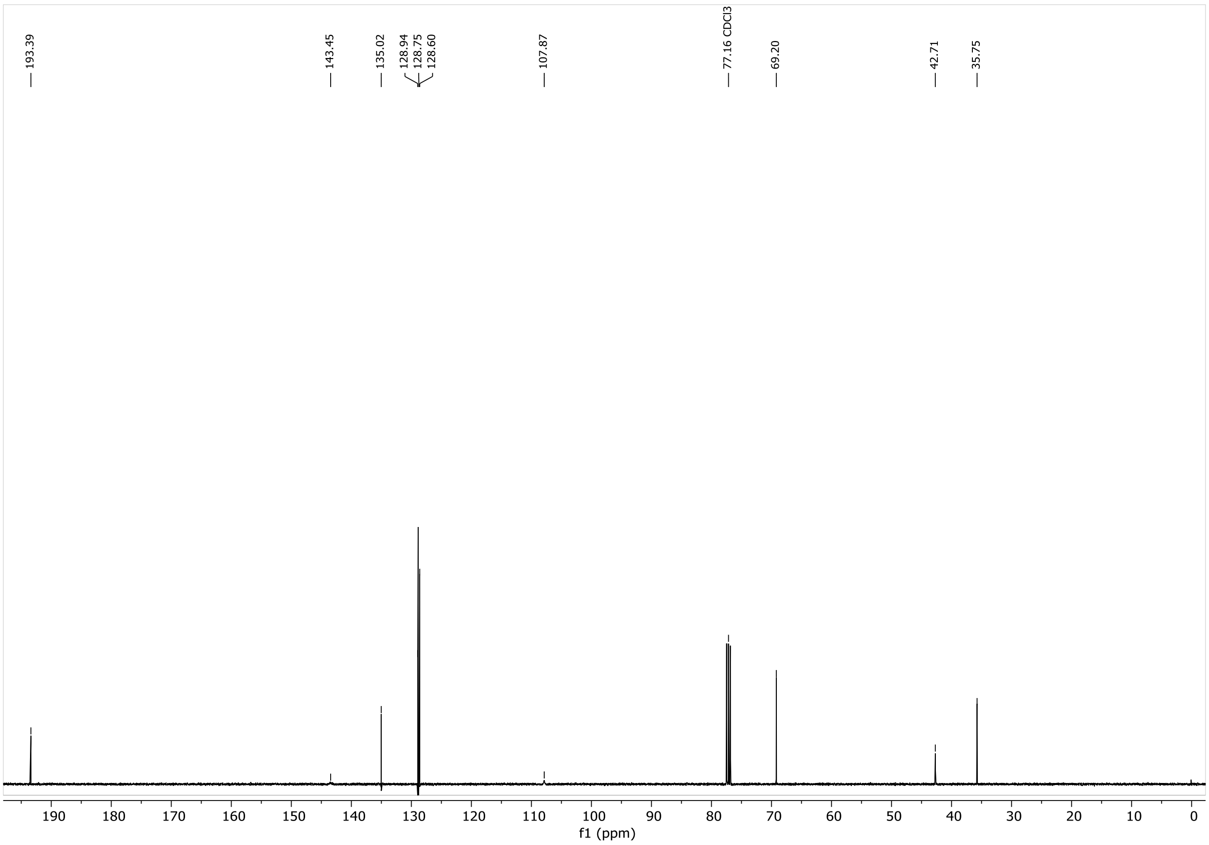

3-methylquinolin-4(1H)-one; ^{1}H NMR (400 MHz, DMSO-D$_6$)

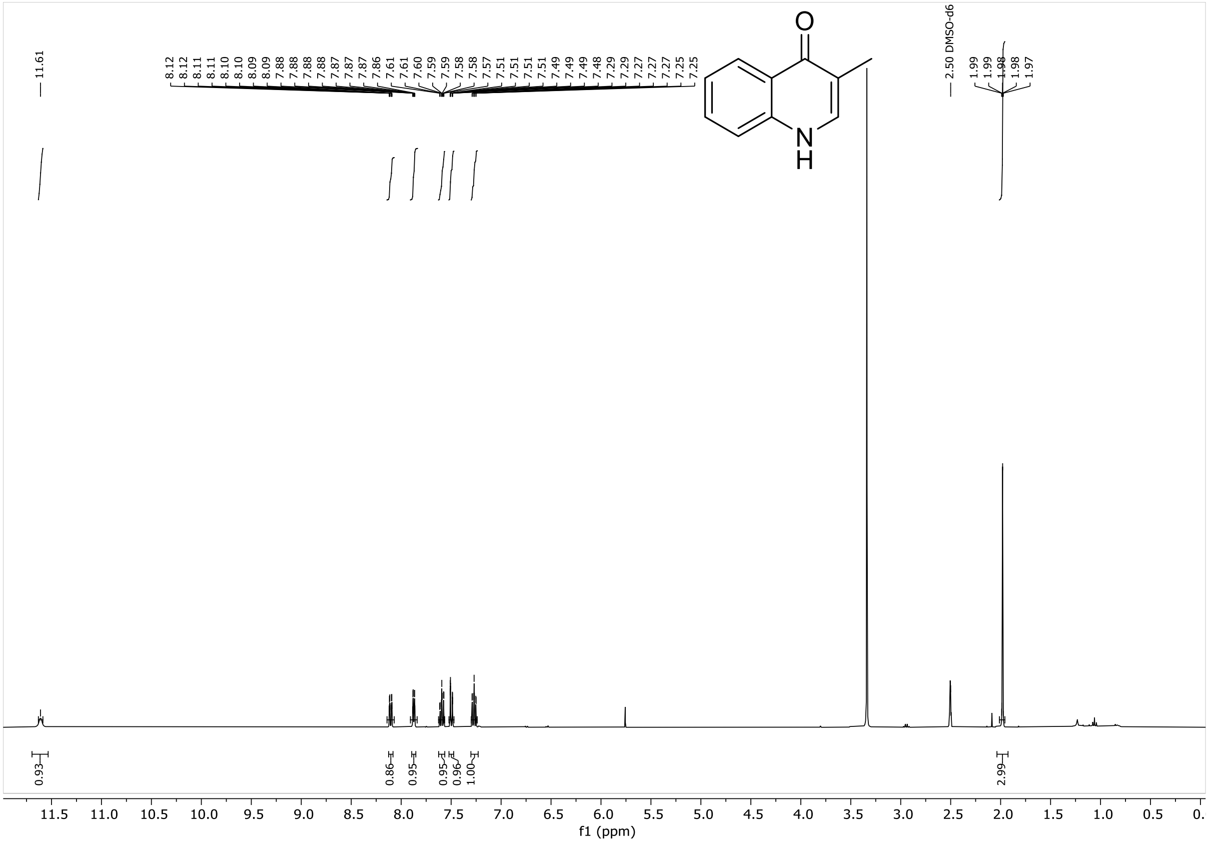

^{13}C{^{1}H} NMR (101 MHz, DMSO-D$_6$)

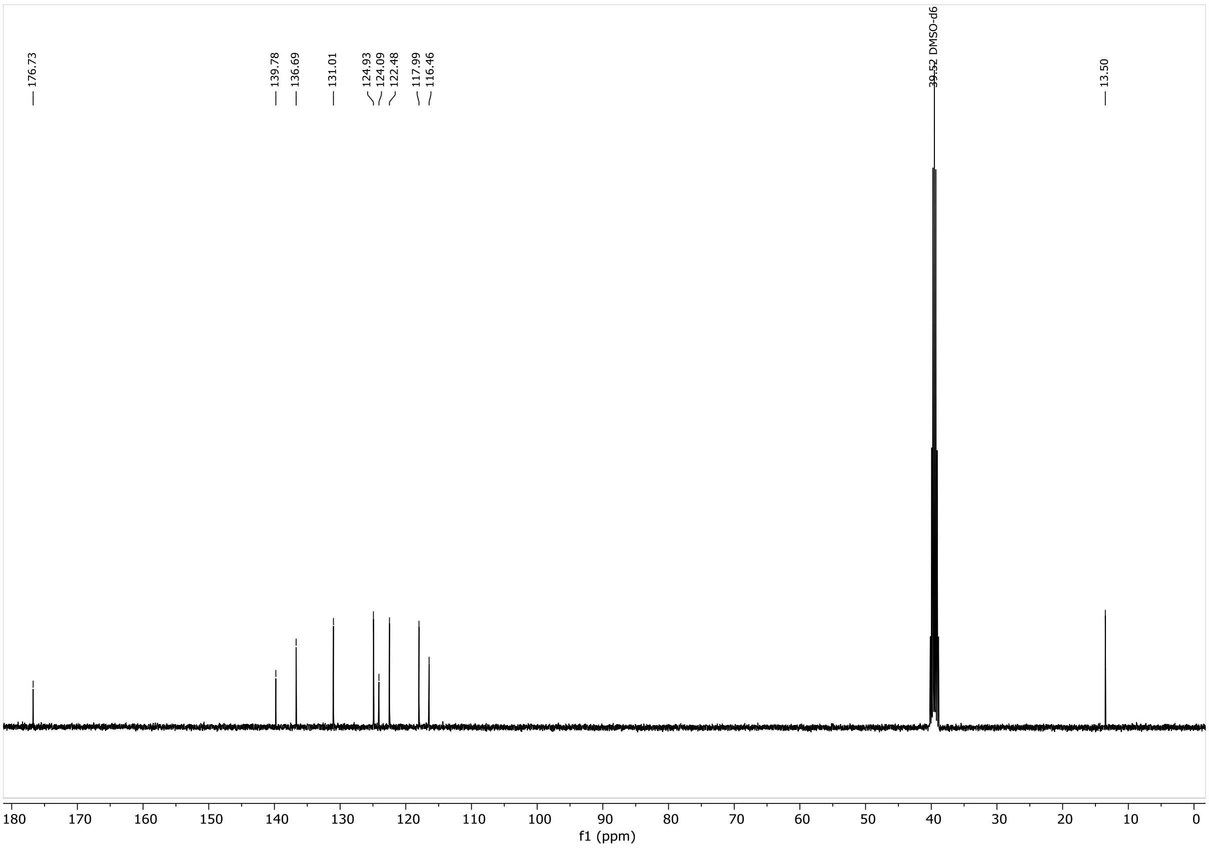

Ethyl 3-methyl-4-oxoquinoline-1(4H)-carboxylate (7); ^{1}H NMR (400 MHz, $CDCl_3$)

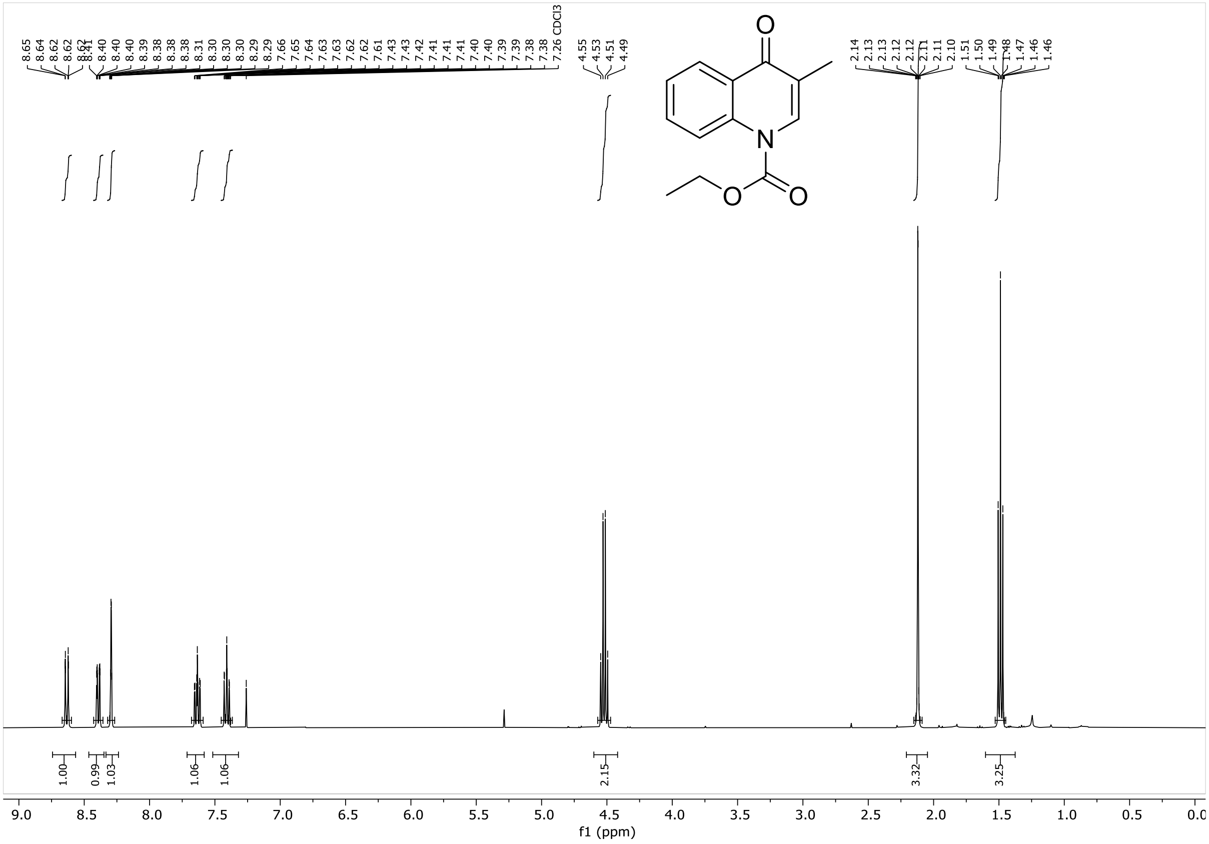

^{13}C{^{1}H} NMR (101 MHz, $CDCl_3$)

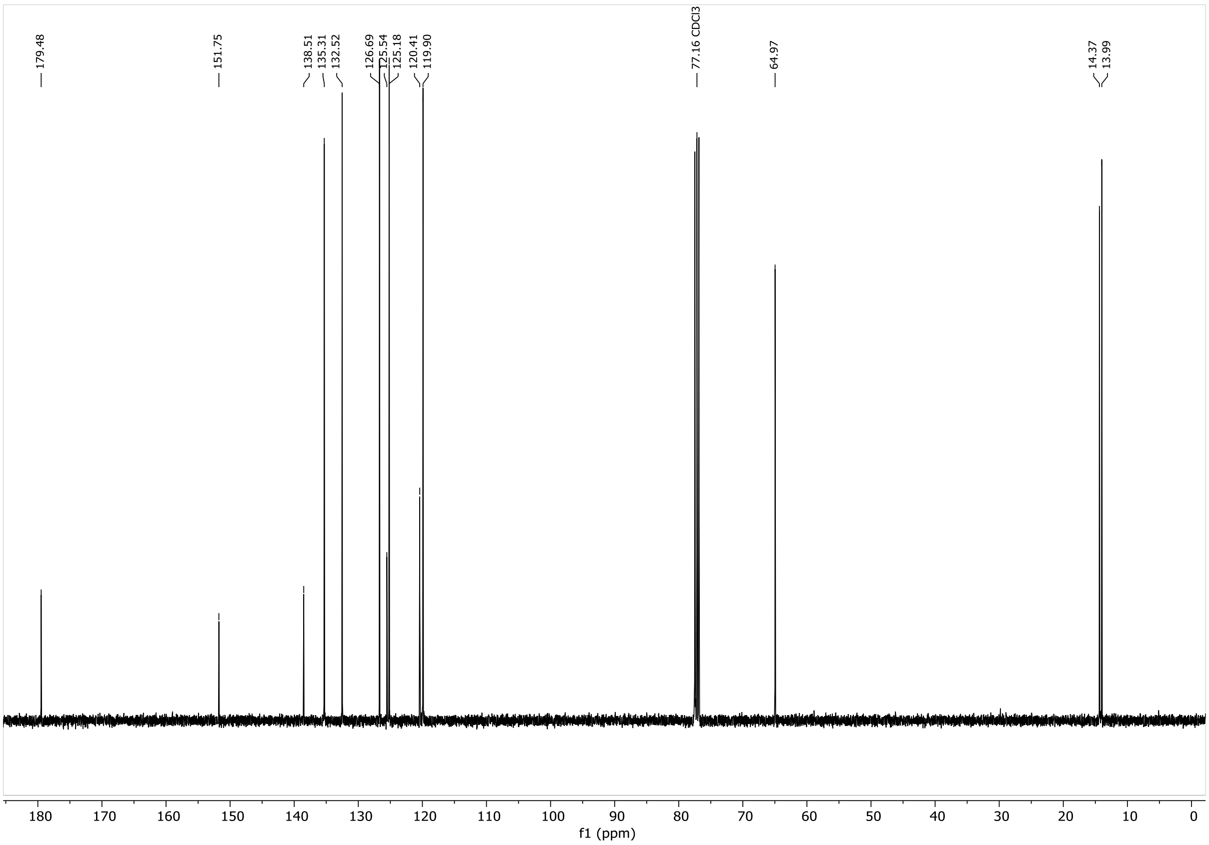

1-ethynyl-4-nitrobenzene (2h); ^{1}H NMR (400 MHz, $CDCl_3$)

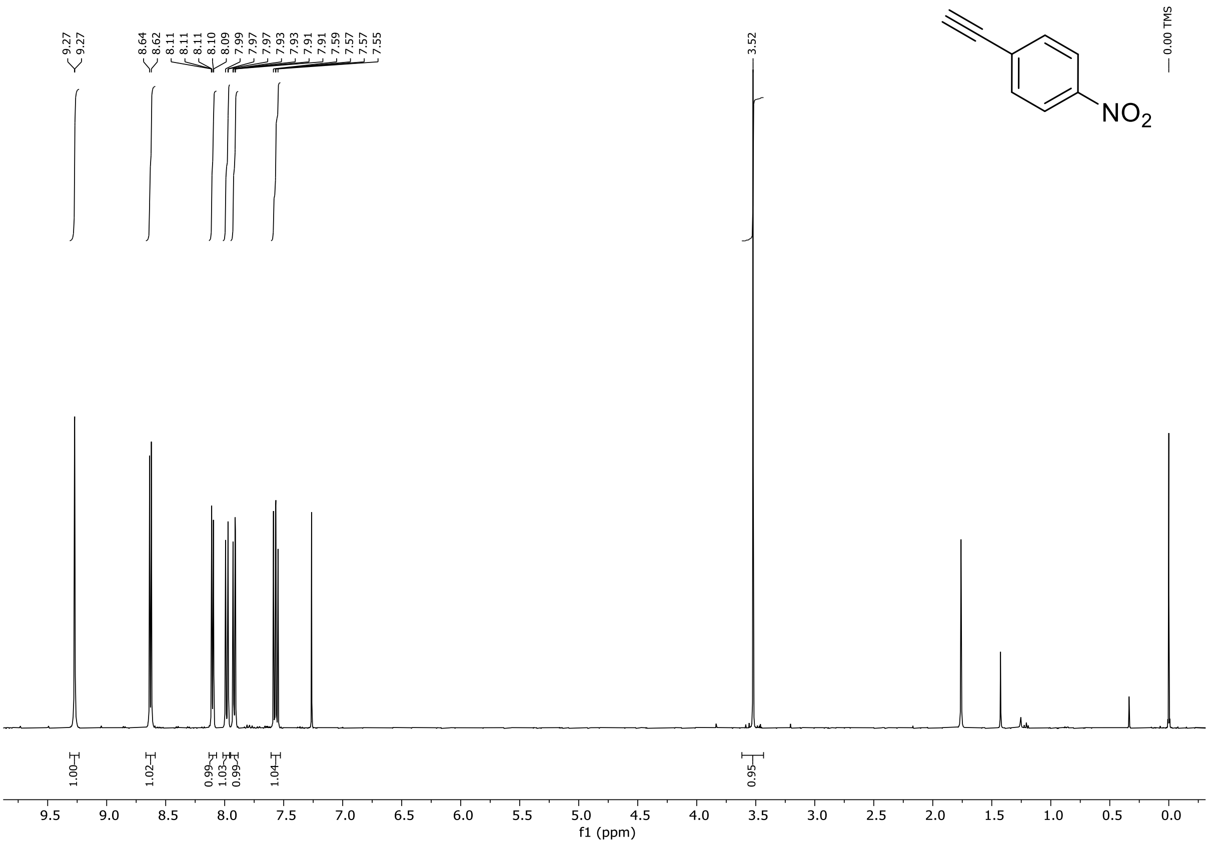

^{13}C{^{1}H} NMR (101 MHz, $CDCl_3$)

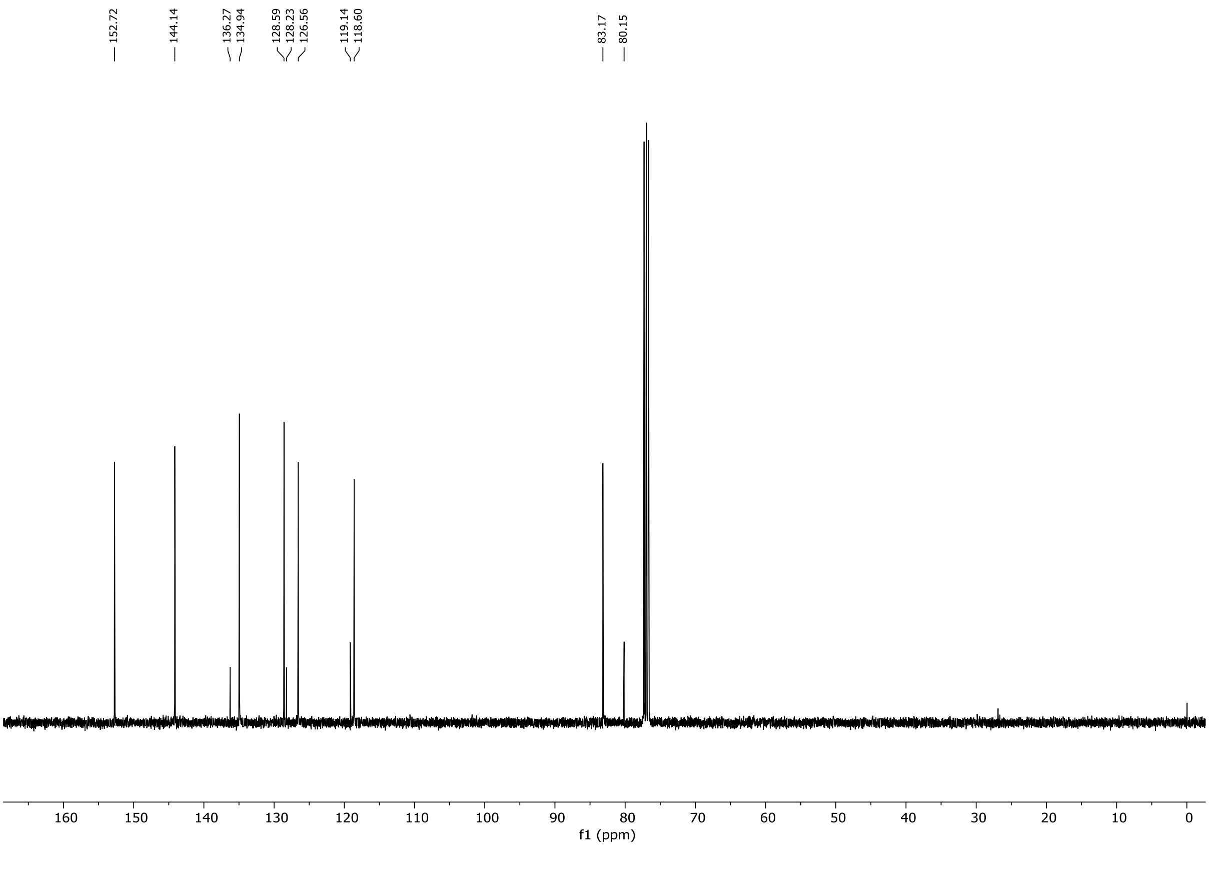

4-ethynyl-1,2-dimethoxybenzene (2i); ^{1}H NMR (400 MHz, $CDCl_3$)

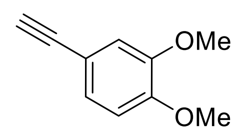

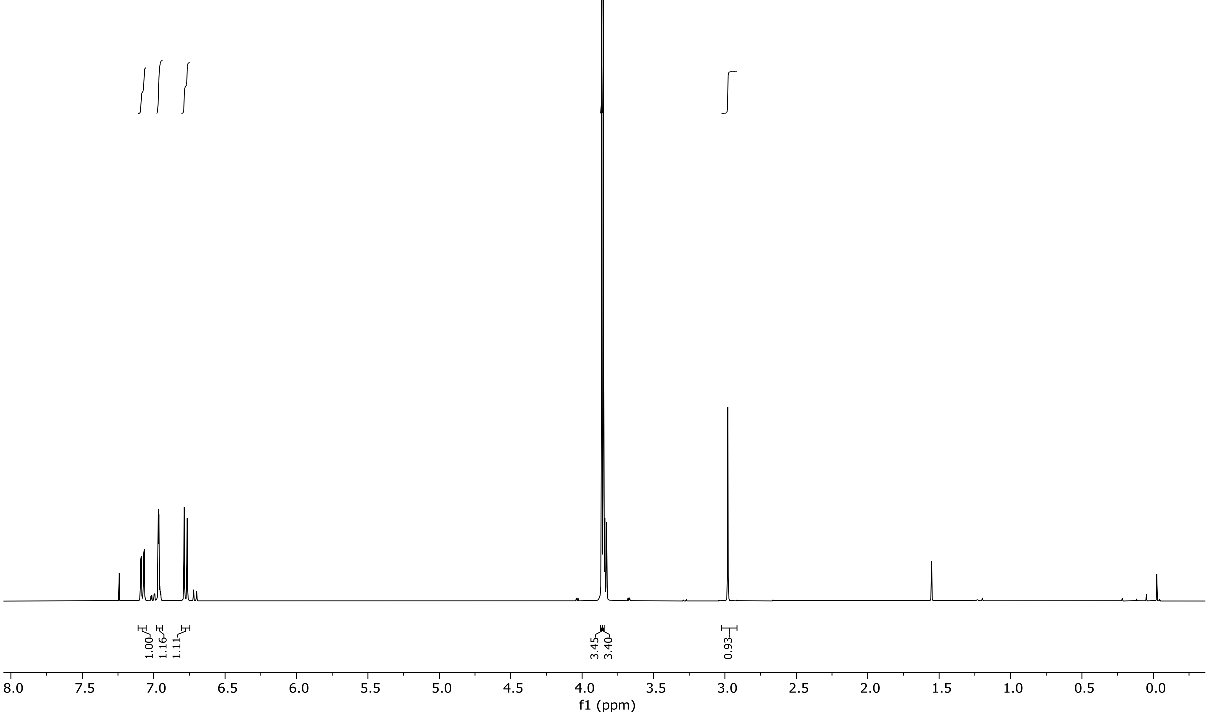

^{13}C{^{1}H} NMR (101 MHz, $CDCl_3$)

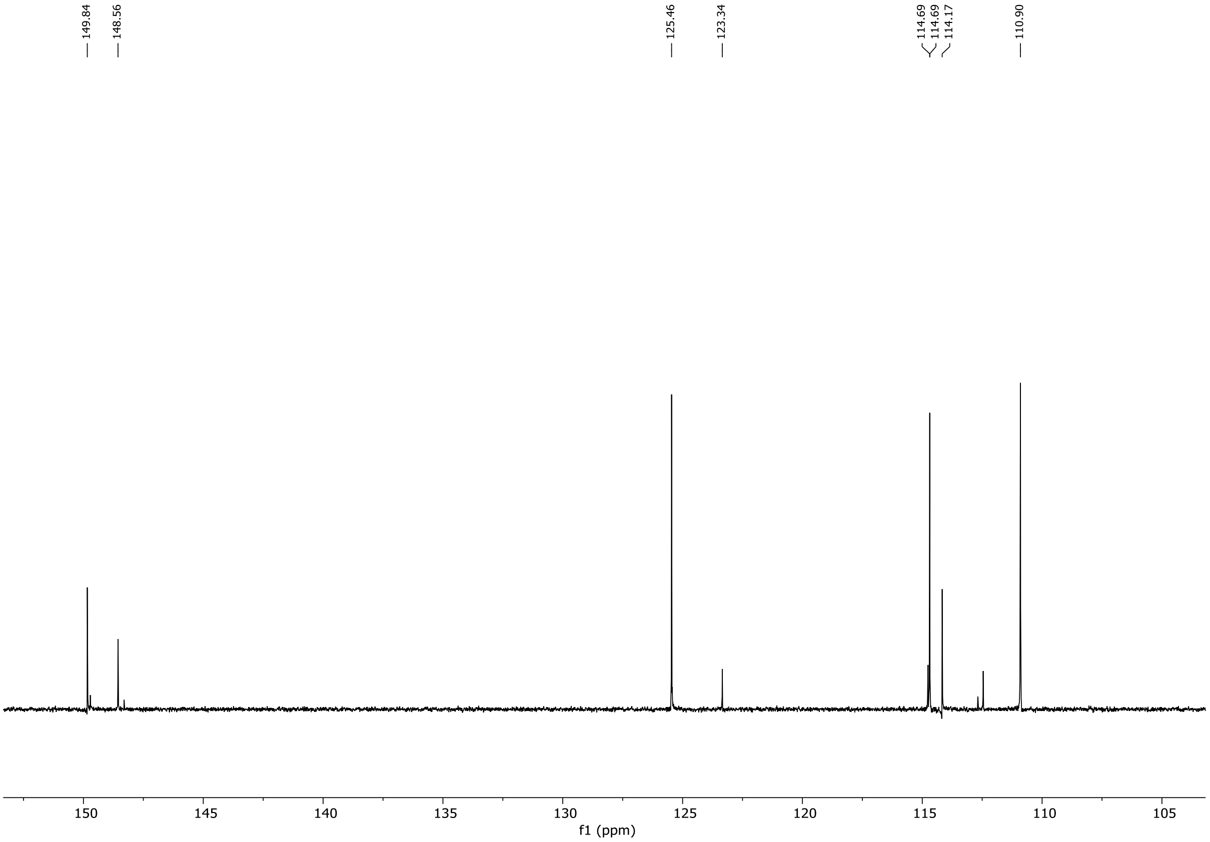

3,4-methylenedioxyphenylethyne (2j); ^{1}H NMR (400 MHz, $CDCl_3$)

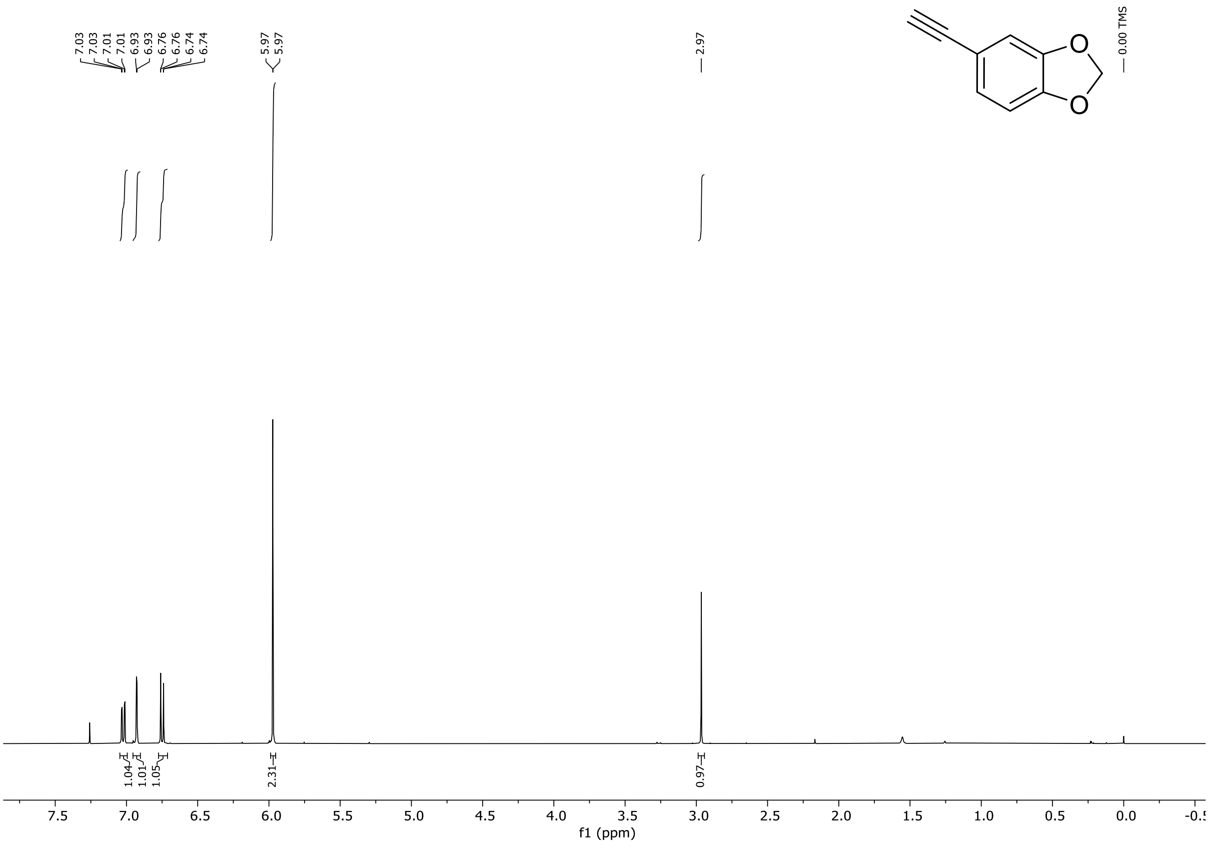

^{13}C{^{1}H} NMR (101 MHz, $CDCl_3$)

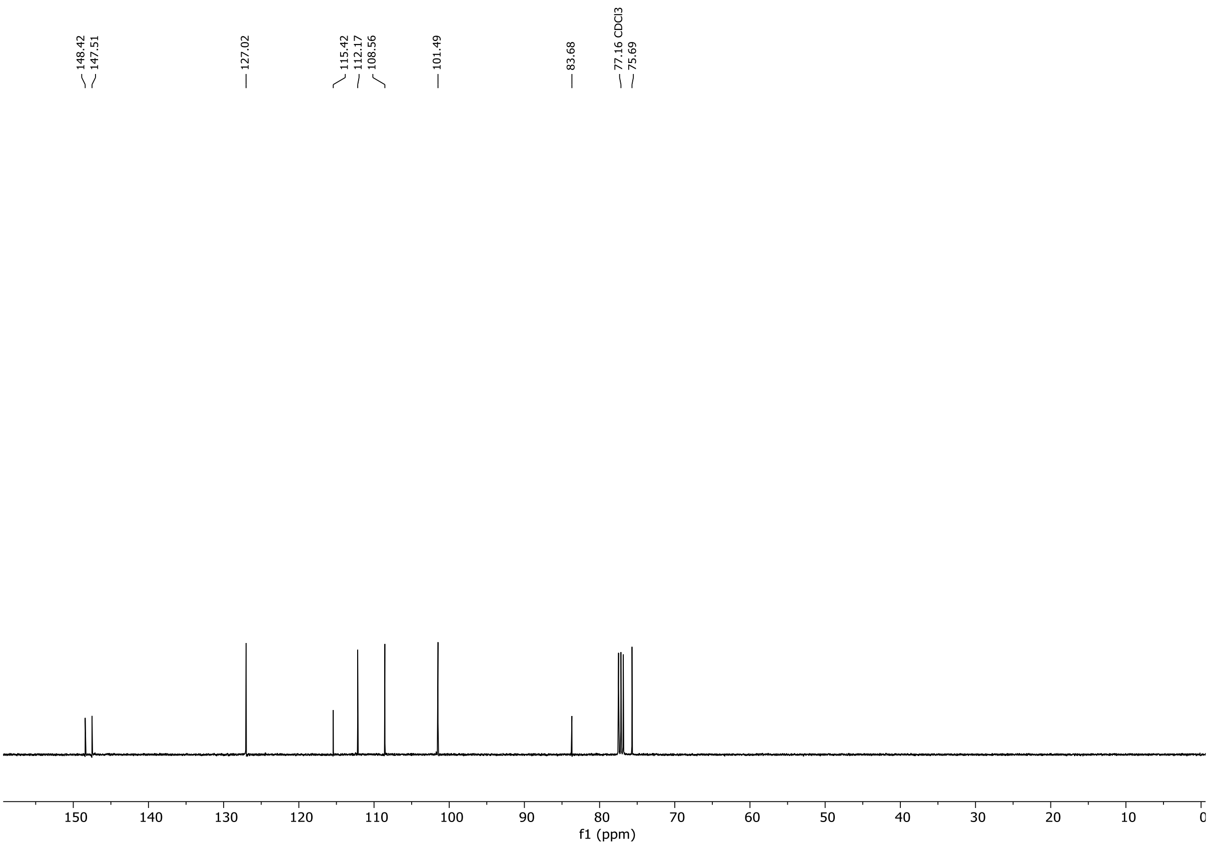

((prop-2-yn-1-yloxy)methyl)benzene (2o); ^{1}H NMR (400 MHz, $CDCl_3$)

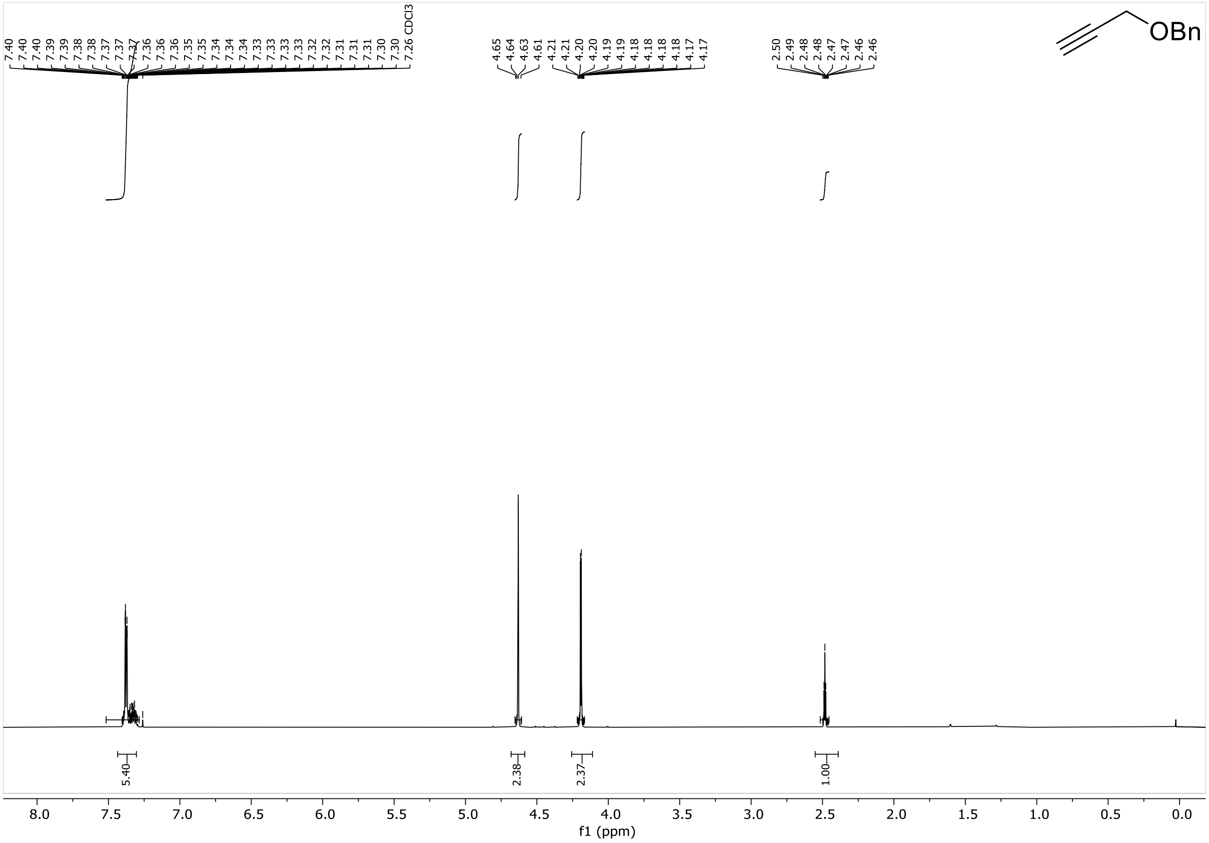

^{13}C{^{1}H} NMR (101 MHz, $CDCl_3$)

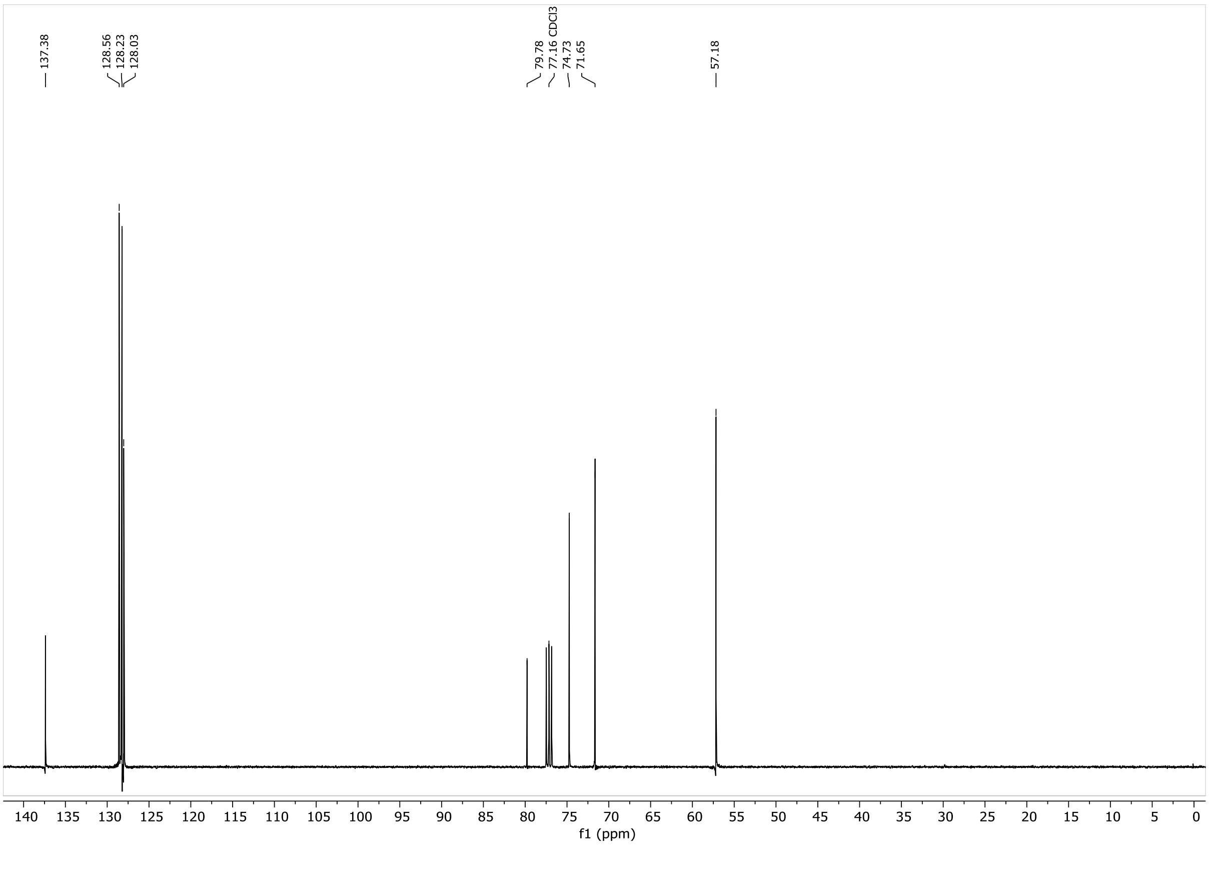

Benzyl (*S*)-4-oxo-2-(phenylethynyl)-3,4-dihydroquinoline-1(2H)-carboxylate (3a); ^{1}H NMR (400 MHz, $CDCl_3$)

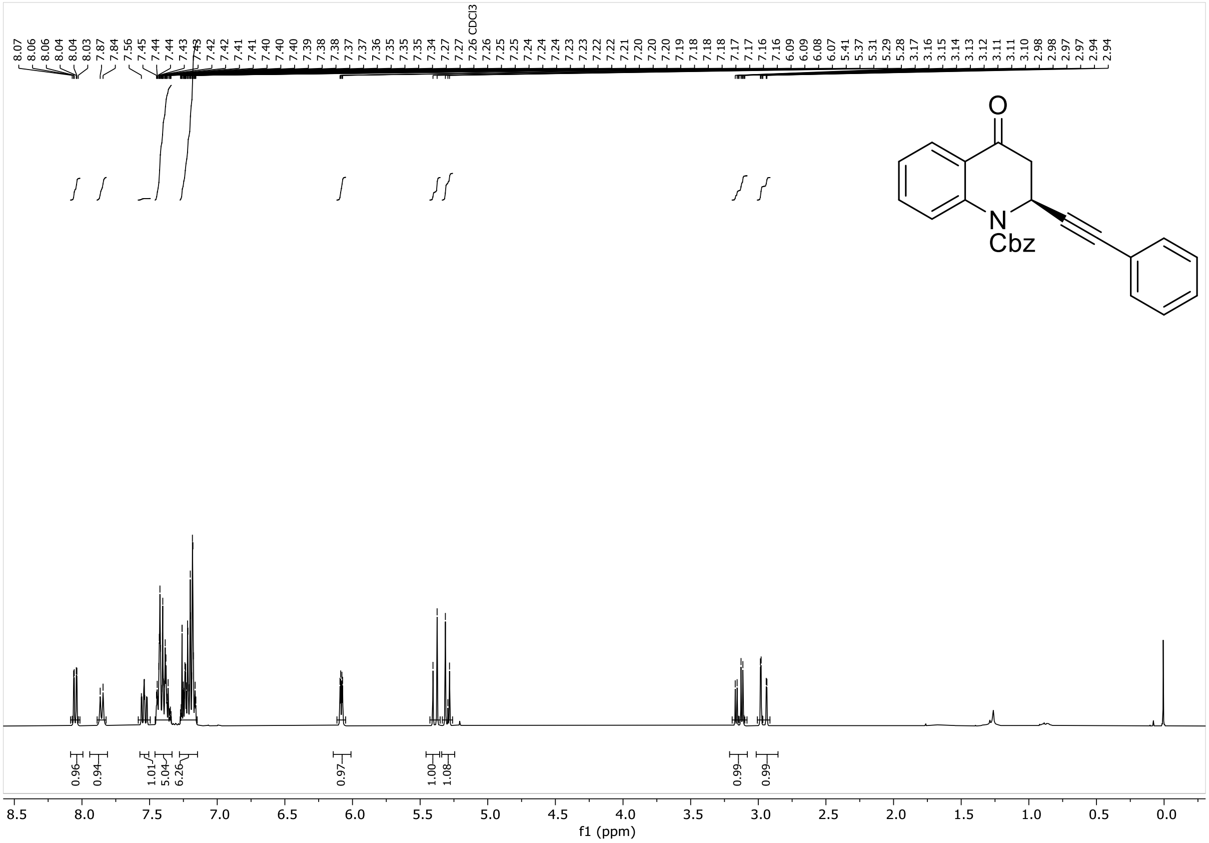

$^{13}C\{^{1}H\}$ NMR (101 MHz, $CDCl_3$)

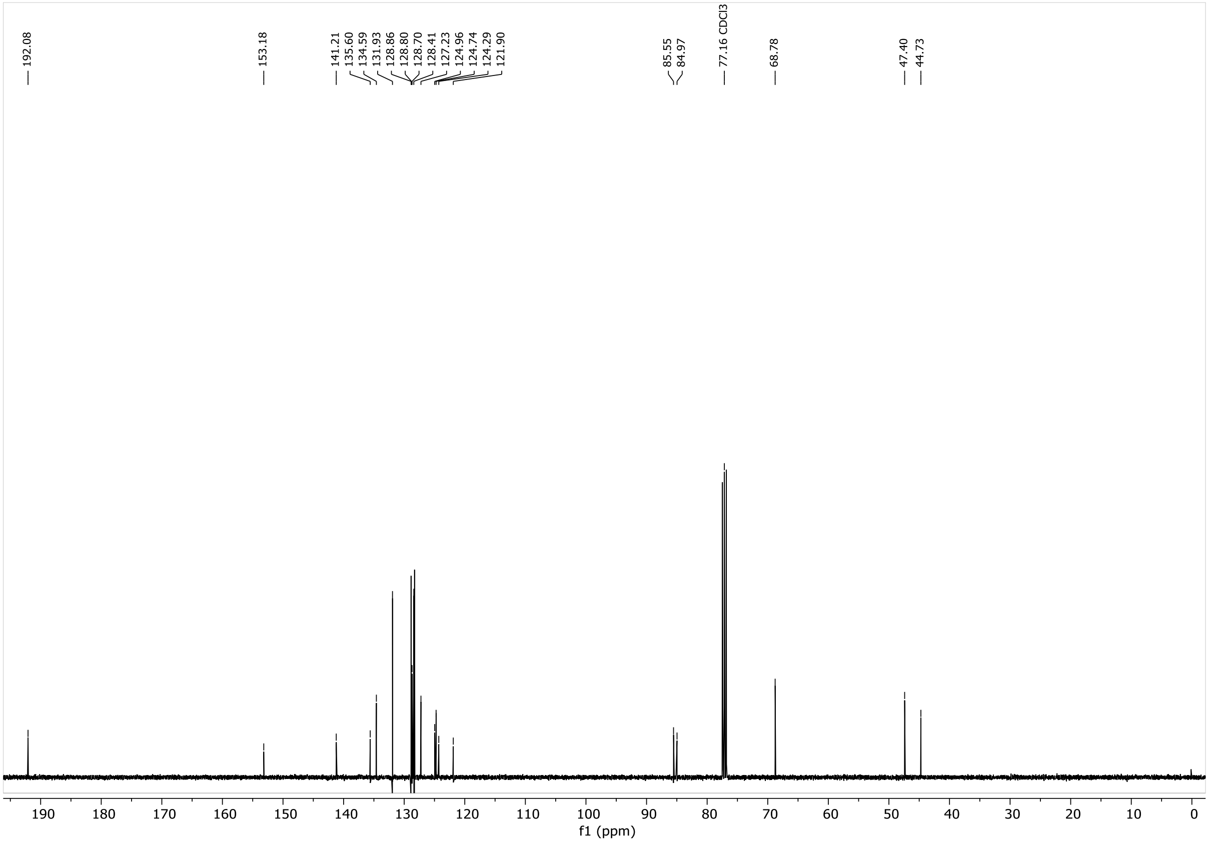

Benzyl (*S*)-4-oxo-2-(*p*-tolylethynyl)-3,4-dihydroquinoline-1(2H)-carboxylate (3b); ^{1}H NMR (400 MHz, $CDCl_3$)

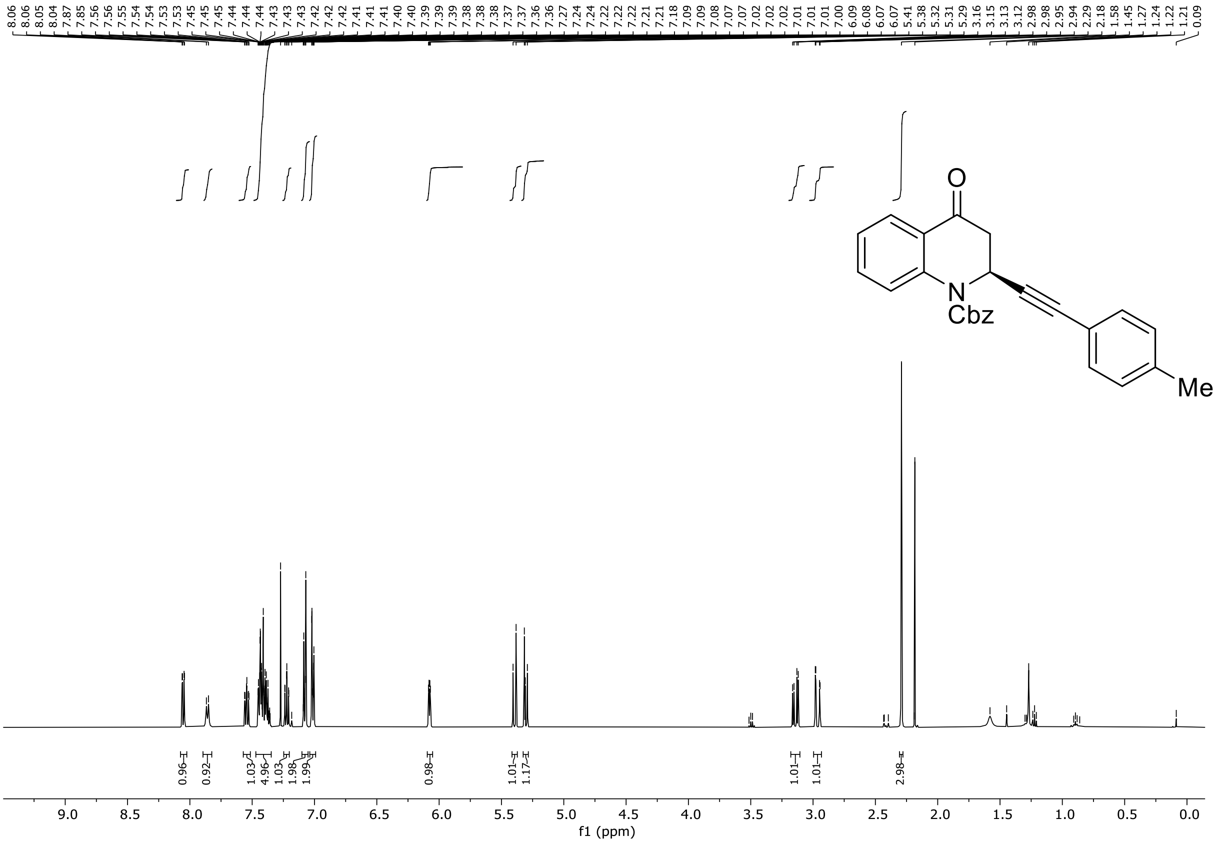

^{13}C{^{1}H} NMR (101 MHz, $CDCl_3$)

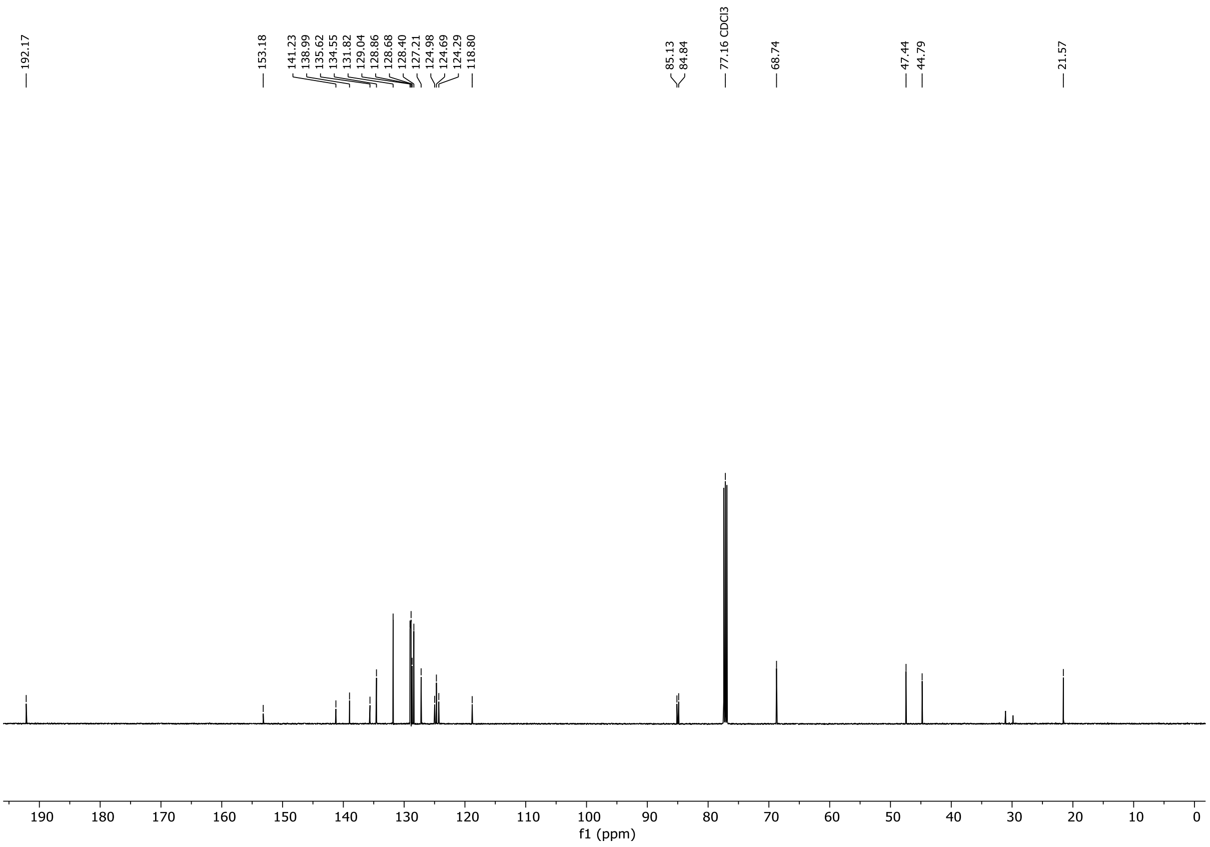

Benzyl (*S*)-4-oxo-2-(*m*-tolylethynyl)-3,4-dihydroquinoline-1(2H)-carboxylate (3c); ^{1}H NMR (400 MHz, $CDCl_3$)

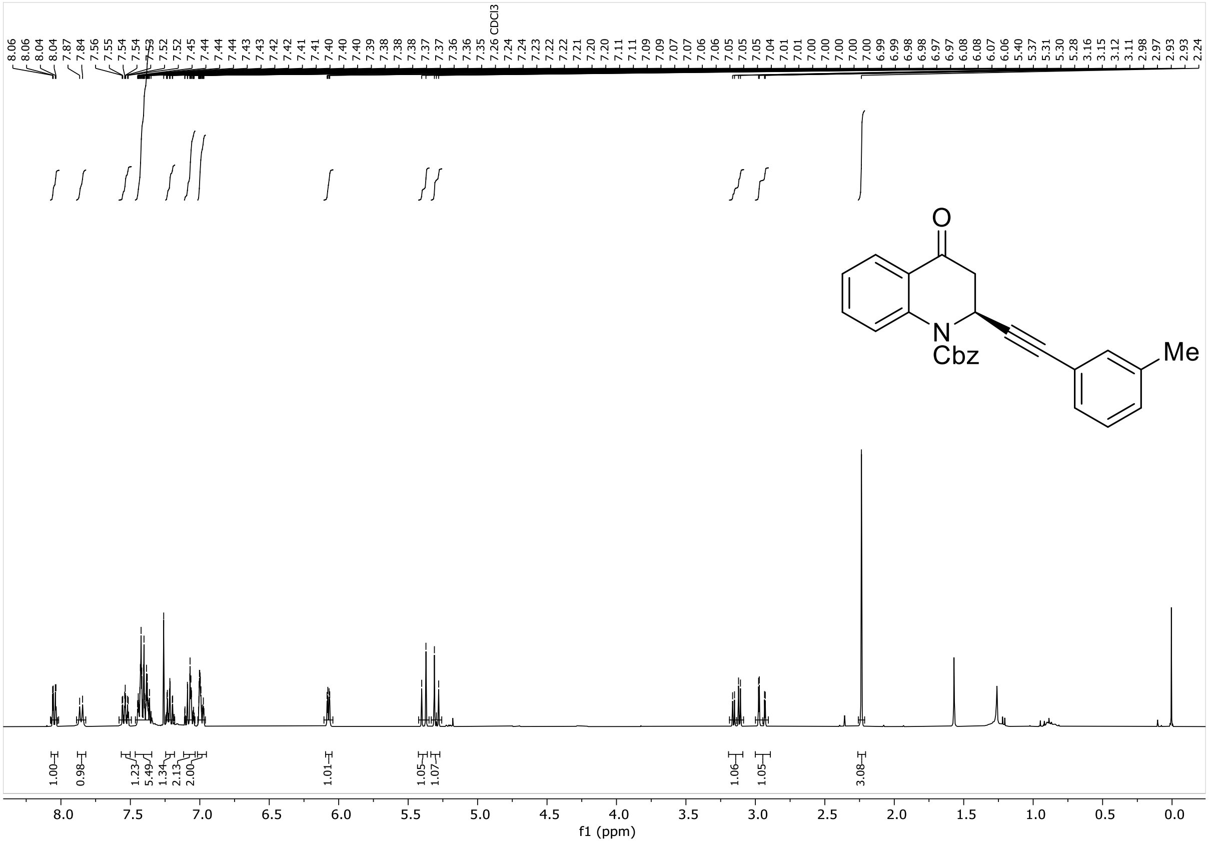

^{13}C{^{1}H} NMR (101 MHz, $CDCl_3$)

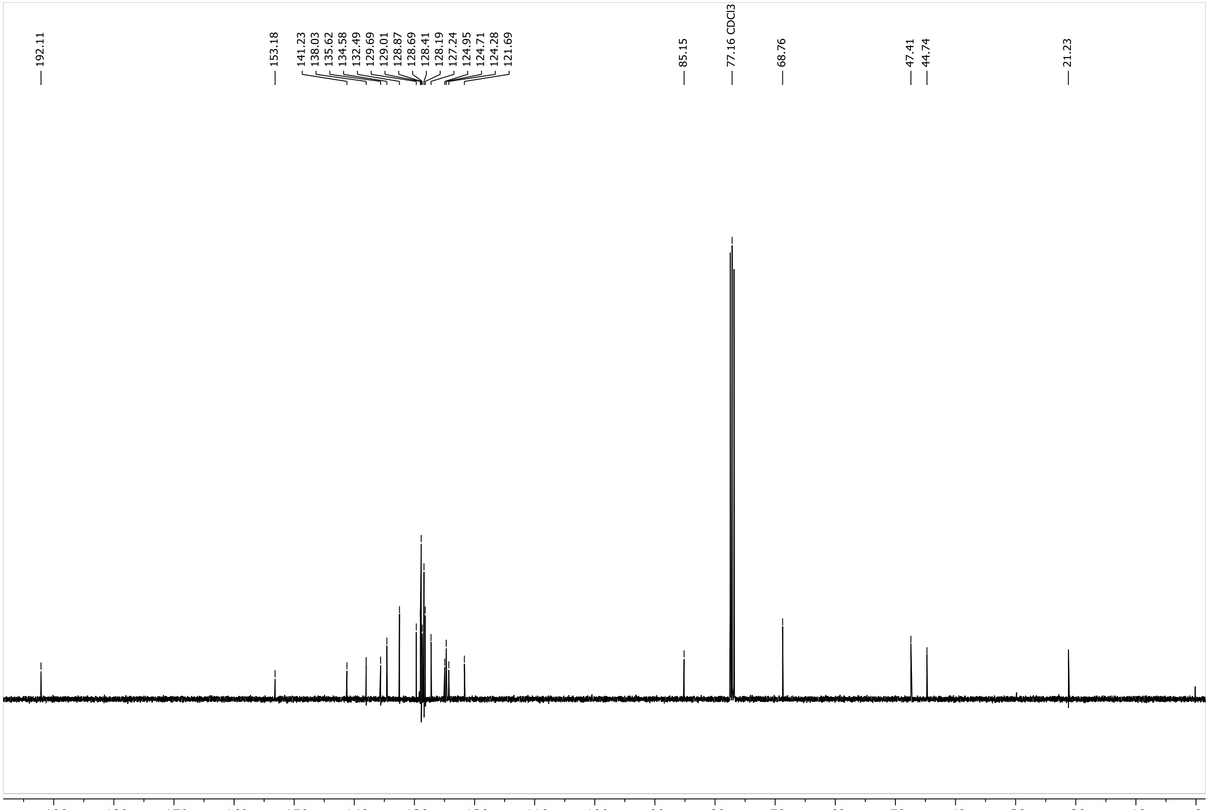

Benzyl (*S*)-4-oxo-2-(*o*-tolylethynyl)-3,4-dihydroquinoline-1(2H)-carboxylate (3d); ^{1}H NMR (400 MHz, $CDCl_3$)

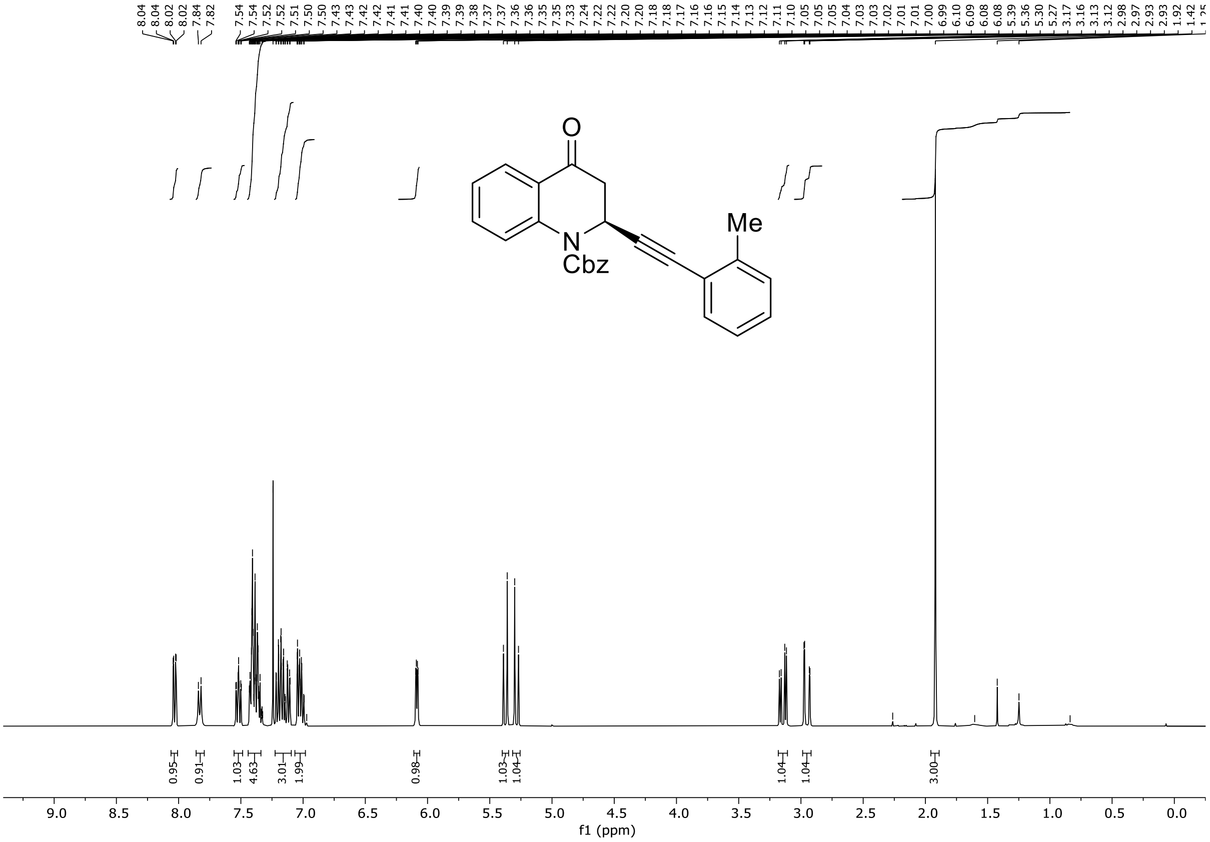

^{13}C{^{1}H} NMR (101 MHz, $CDCl_3$)

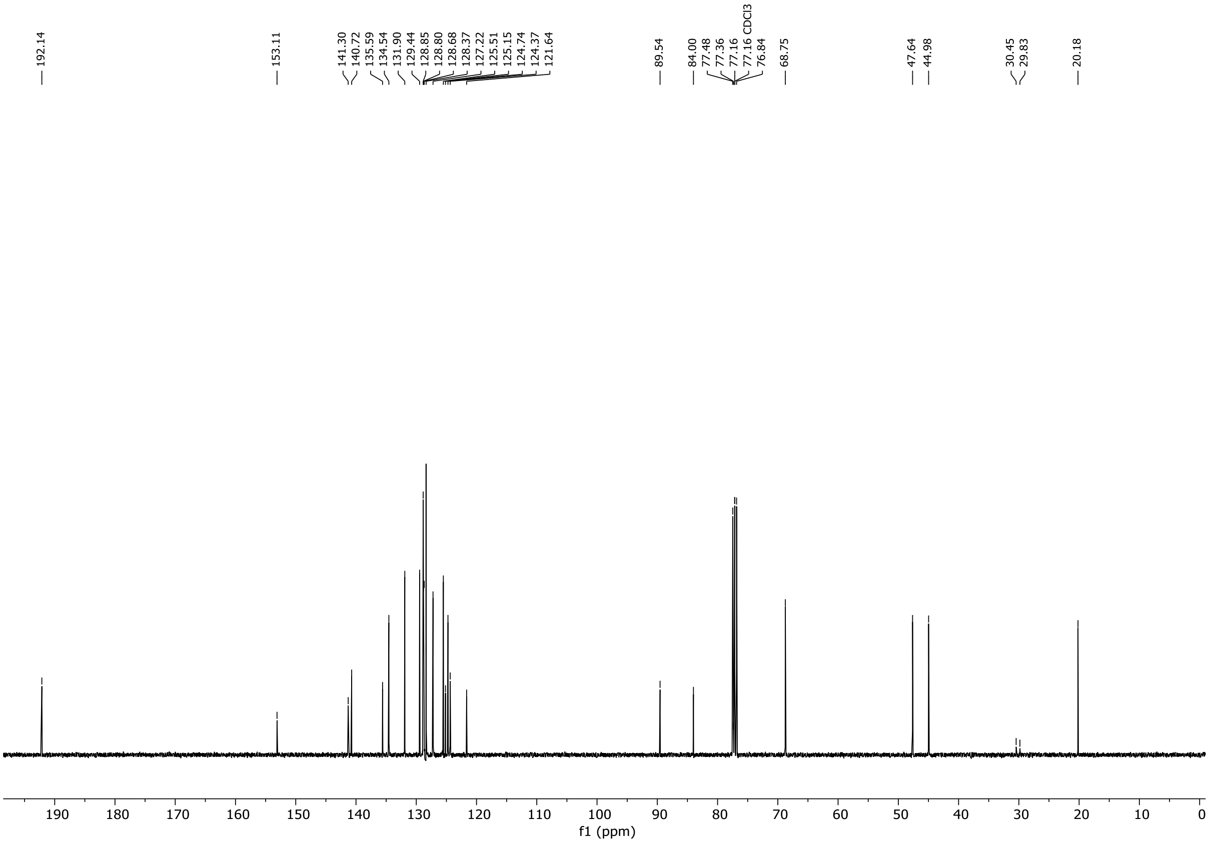

Benzyl (*S*)-2-((3-methoxyphenyl)ethynyl)-4-oxo-3,4-dihydroquinoline-1(2H)-carboxylate (3e); ^{1}H NMR (400 MHz, $CDCl_3$)

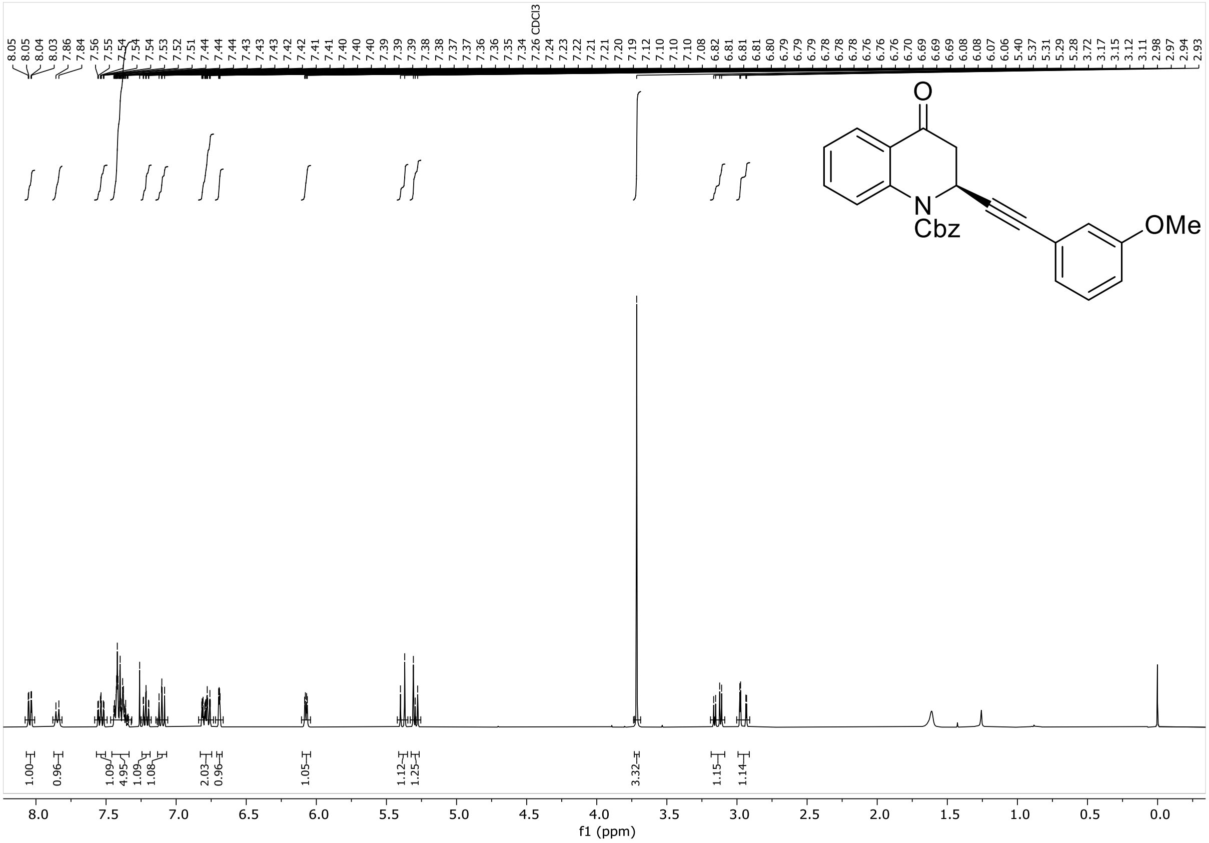

^{13}C{^{1}H} NMR (101 MHz, $CDCl_3$)

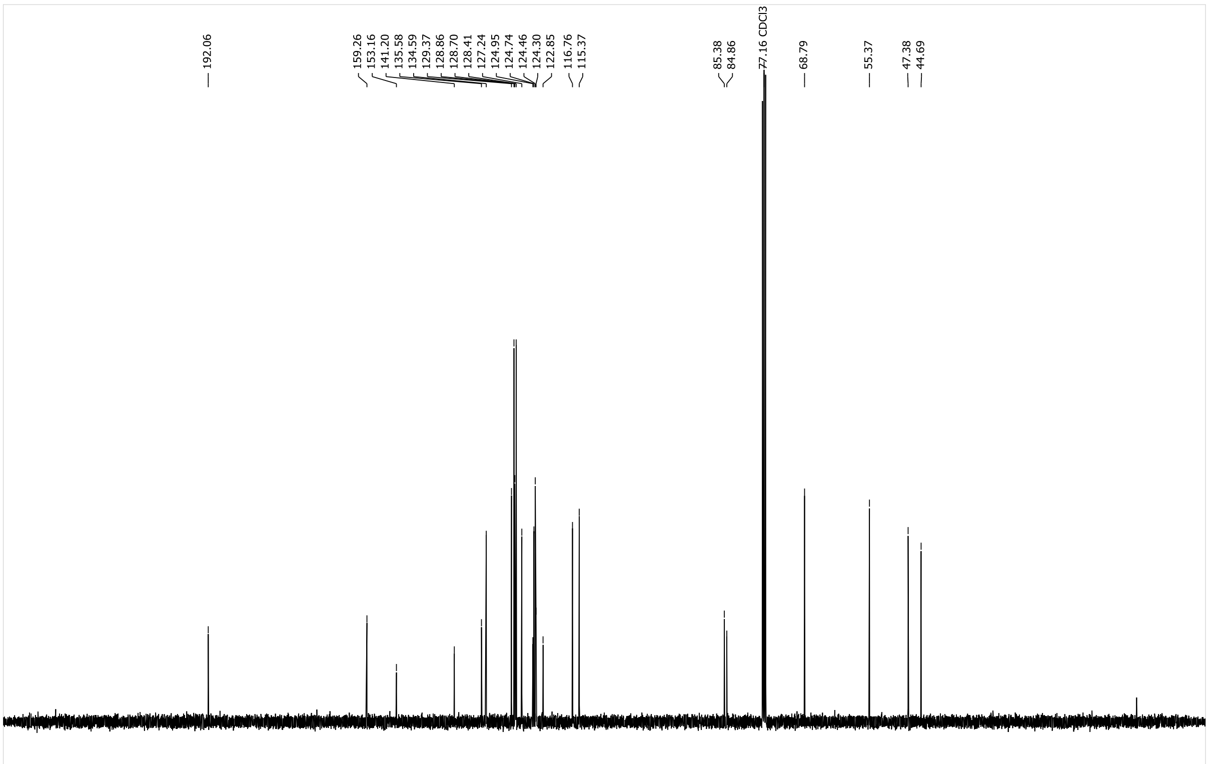

Benzyl (*S*)-2-((3-chlorophenyl)ethynyl)-4-oxo-3,4-dihydroquinoline-1(2H)-carboxylate (3f); **^{1}H NMR** (400 MHz, $CDCl_3$)

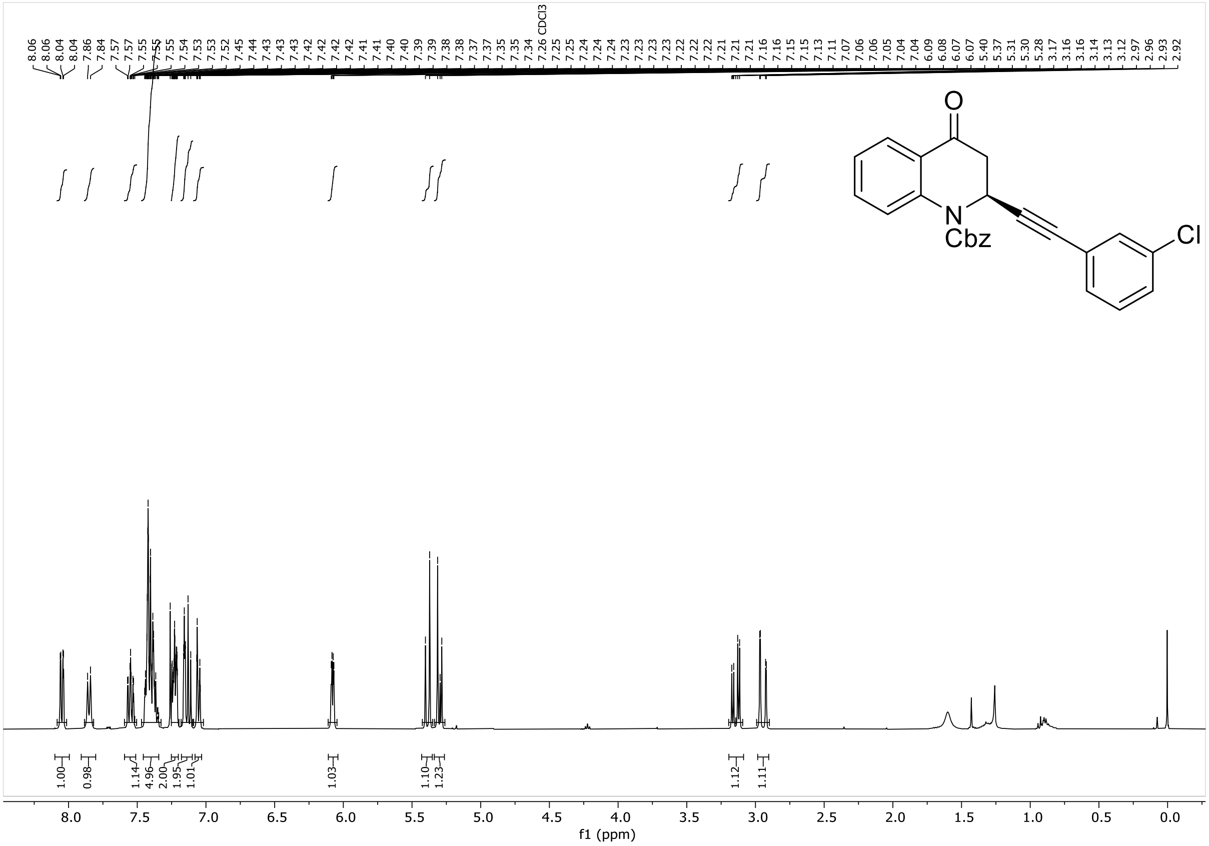

^{13}C{^{1}H} NMR (101 MHz, $CDCl_3$)

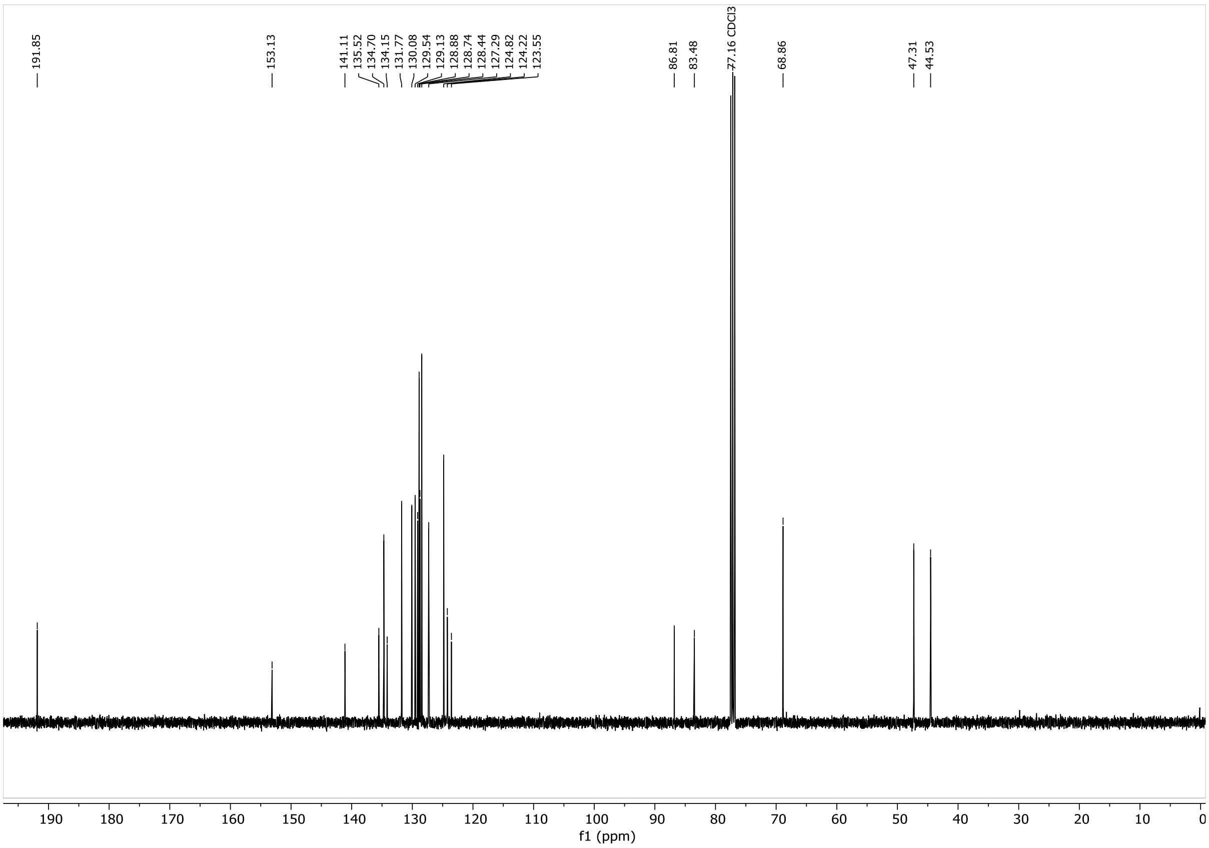

Benzyl (*S*)-2-((4-bromophenyl)ethynyl)-4-oxo-3,4-dihydroquinoline-1(2H)-carboxylate (3g); ^{1}H NMR (400 MHz, $CDCl_3$)

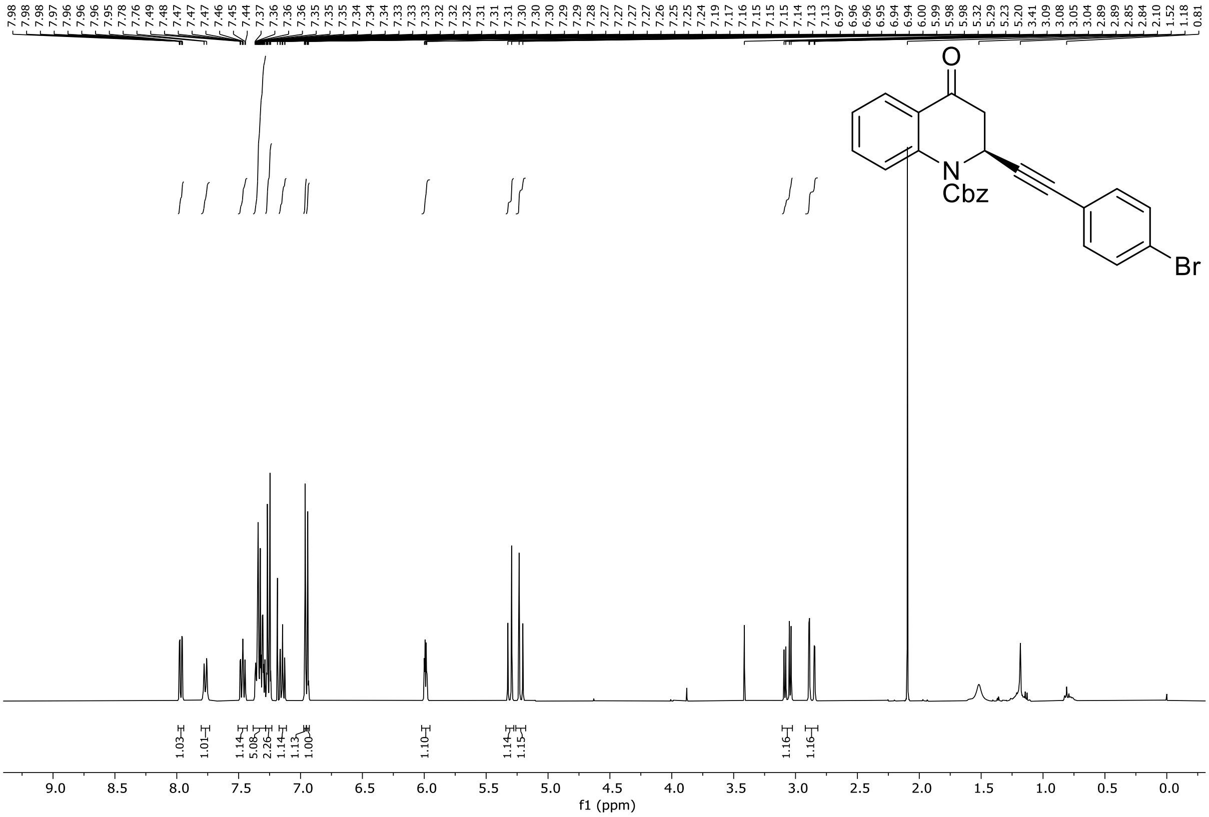

^{13}C{^{1}H} NMR (101 MHz, $CDCl_3$)

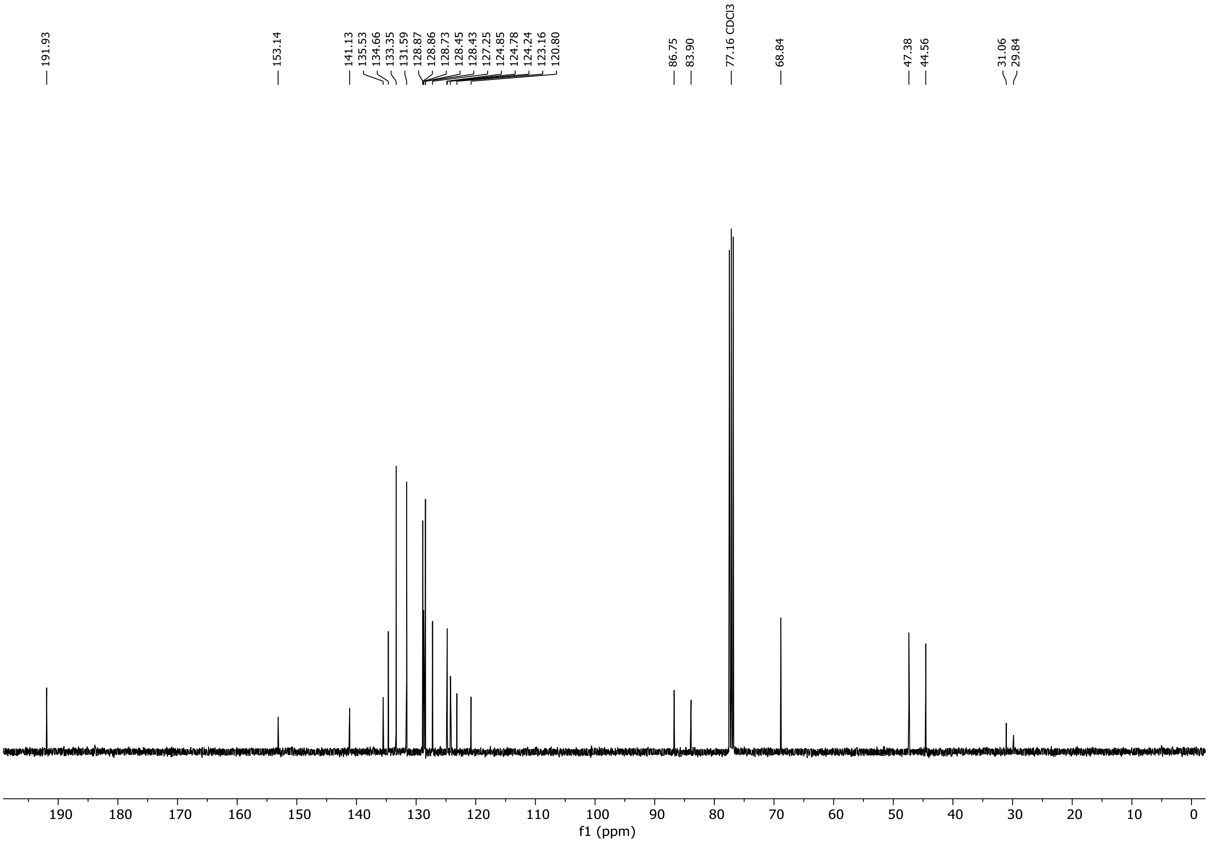

Benzyl (*S*)-2-((4-nitrophenyl)ethynyl)-4-oxo-3,4-dihydroquinoline-1(2H)-carboxylate (3h); ^{1}H NMR (400 MHz, $CDCl_3$)

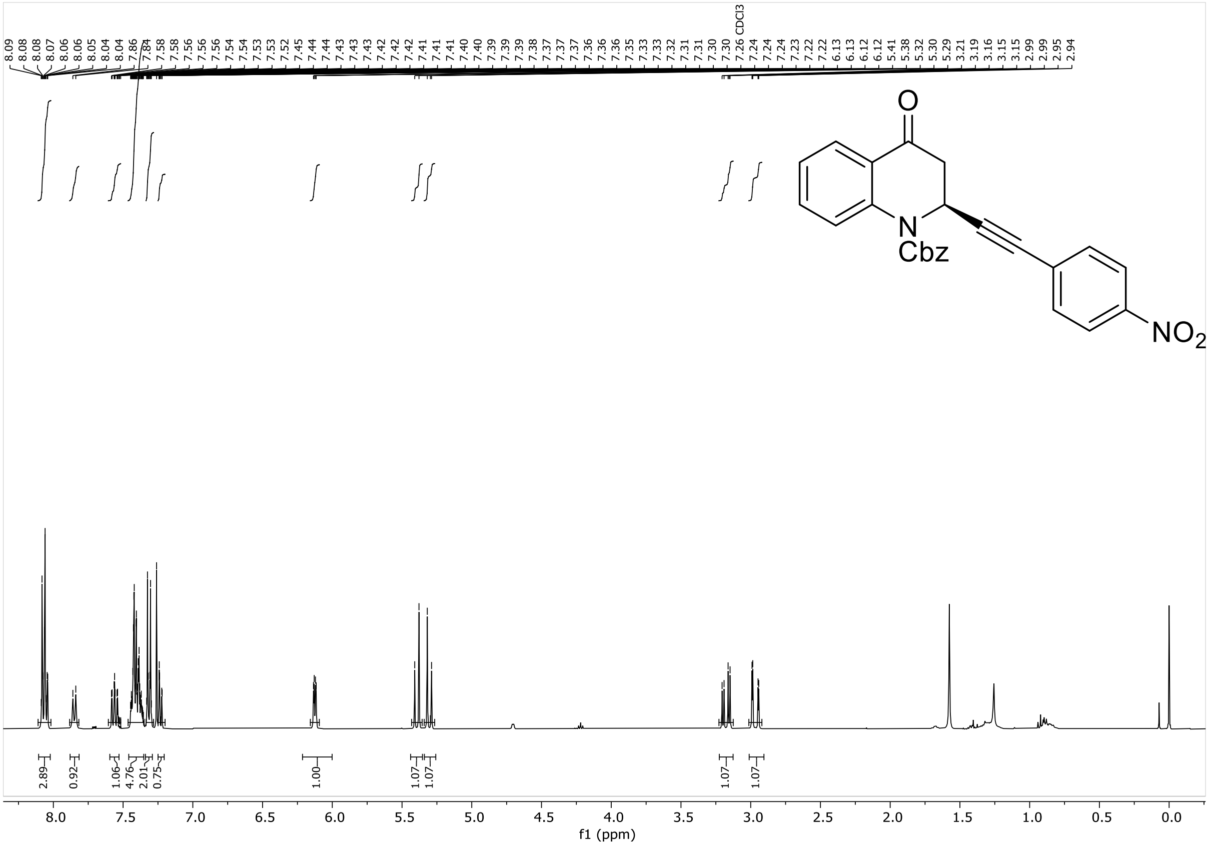

^{13}C{^{1}H} NMR (101 MHz, $CDCl_3$)

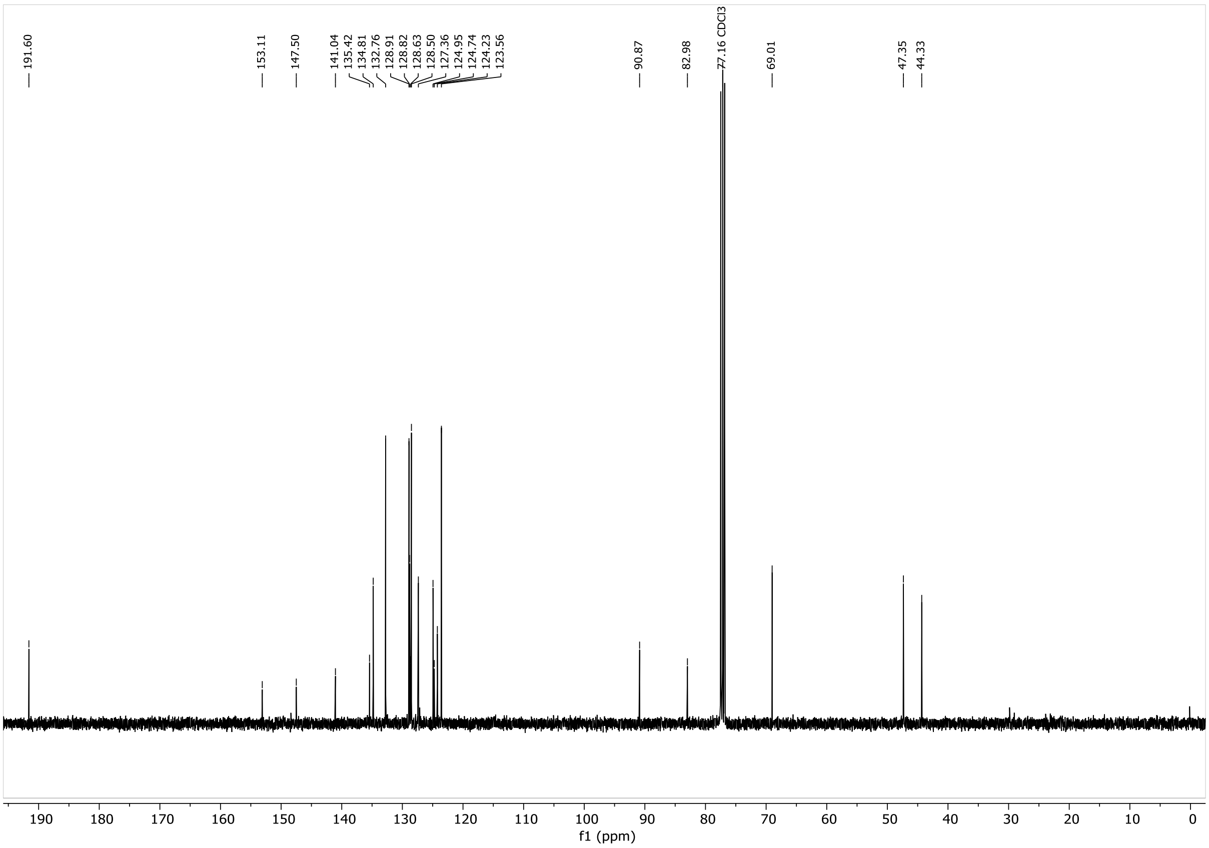

Benzyl (*S*)-2-((3,4-dimethoxyphenyl)ethynyl)-4-oxo-3,4-dihydroquinoline-1(2H)-carboxylate (3i); ^{1}**H NMR** (400 MHz, $CDCl_3$)

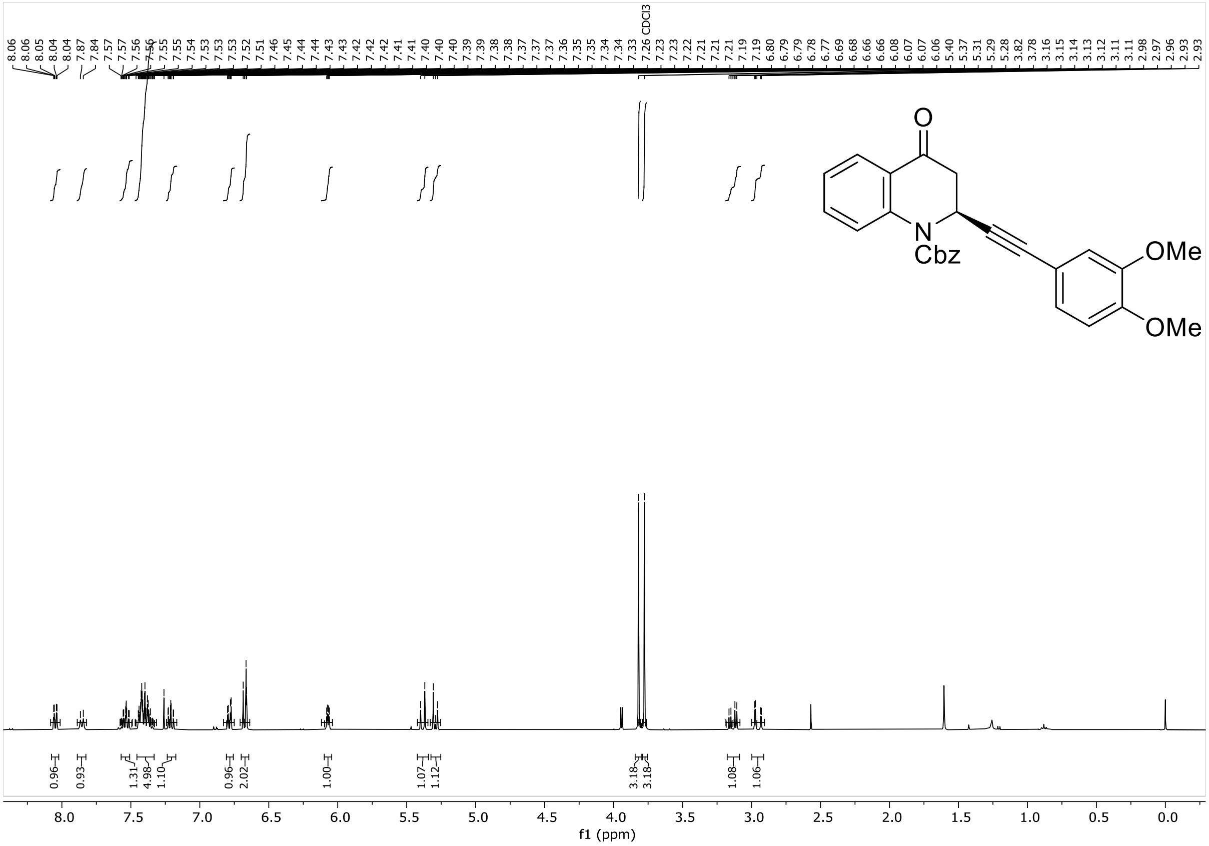

^{13}**C{^{1}H} NMR** (101 MHz, $CDCl_3$)

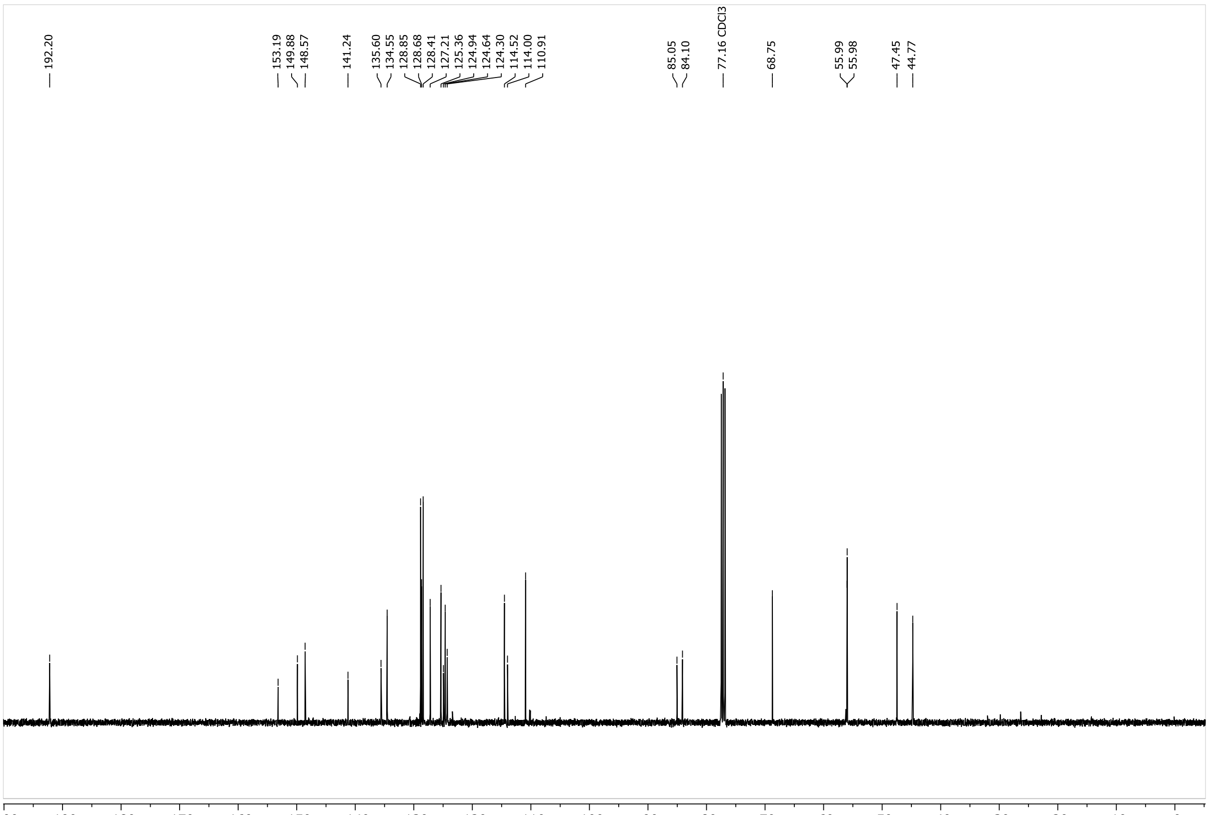

Benzyl (*S*)-2-(benzo[d][1,3]dioxol-5-ylethynyl)-4-oxo-3,4-dihydroquinoline-1(2H)-carboxylate (3j);
^{1}H NMR (400 MHz, $CDCl_3$)

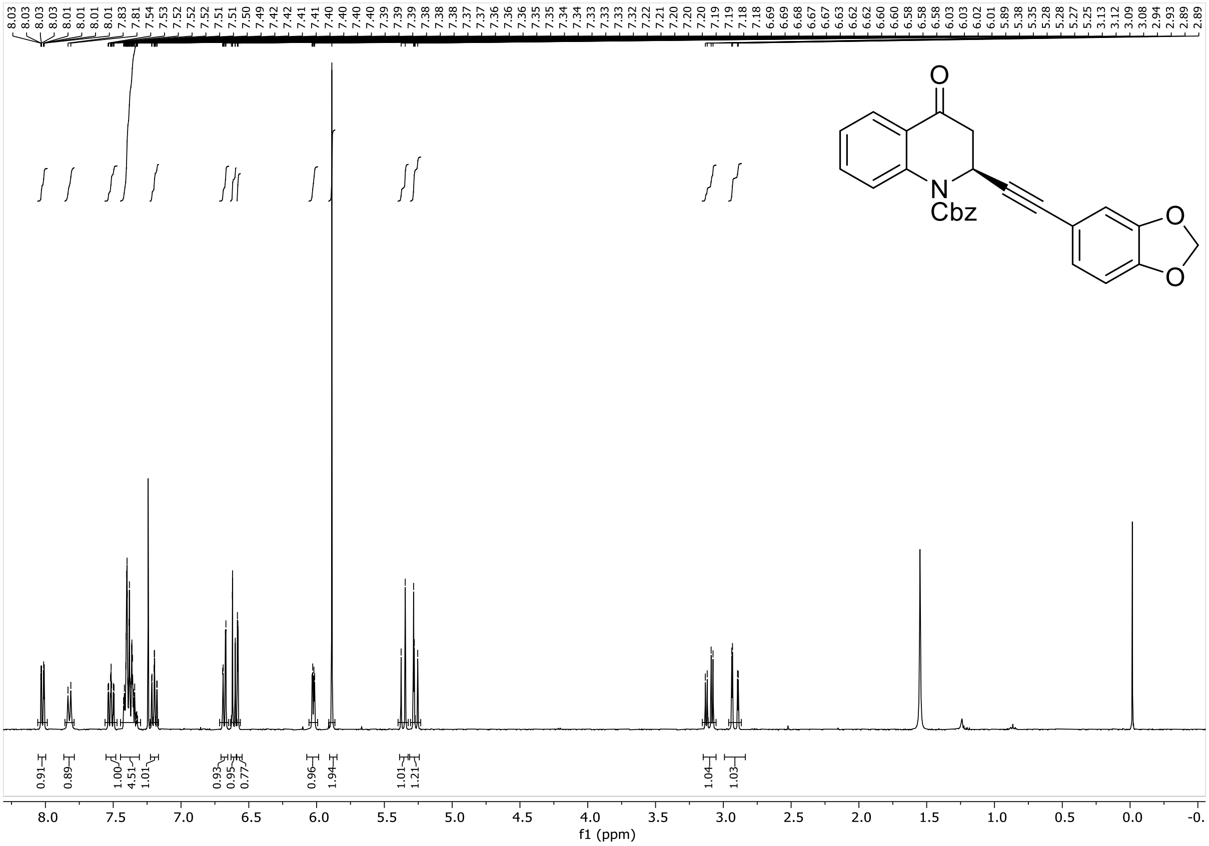

^{13}C{^{1}H} NMR (101 MHz, $CDCl_3$)

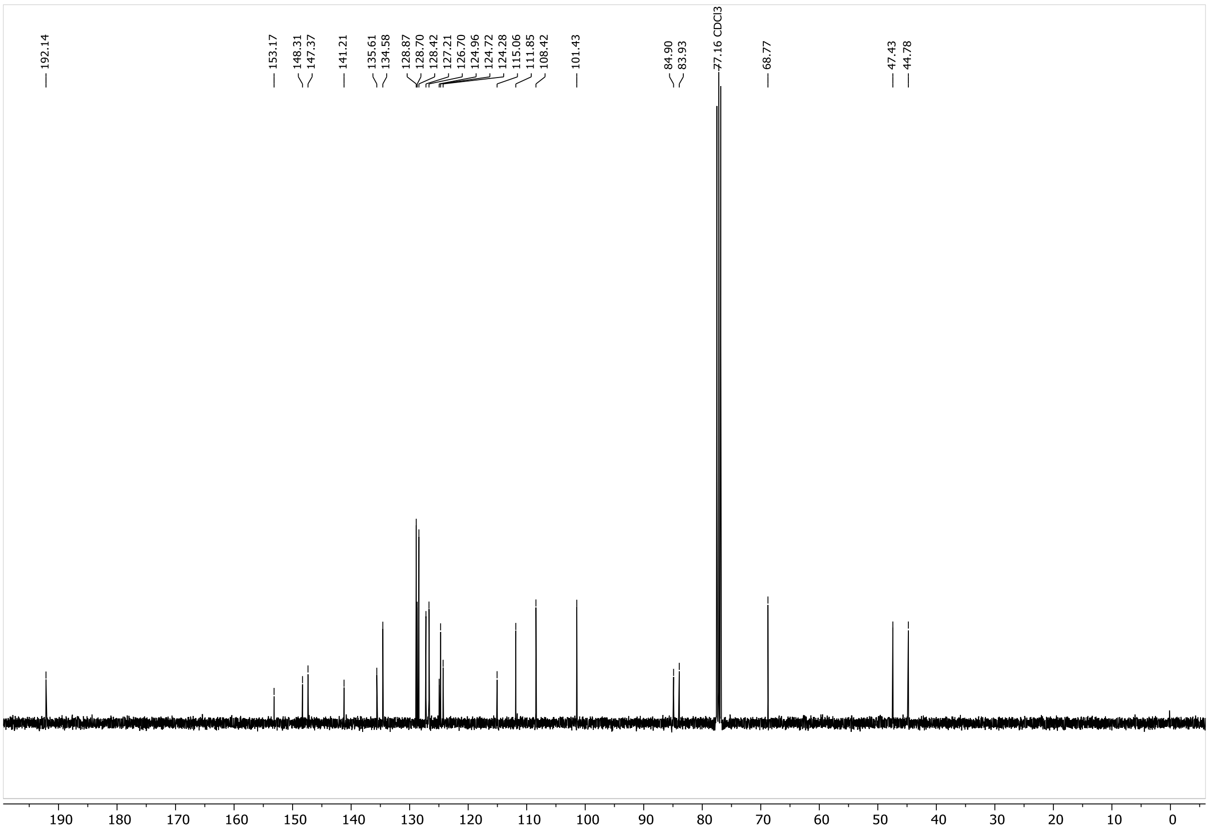

Benzyl (*S*)-4-oxo-2-(phenanthren-9-ylethynyl)-3,4-dihydroquinoline-1(2H)-carboxylate (3k); ^{1}H NMR (400 MHz, $CDCl_3$)

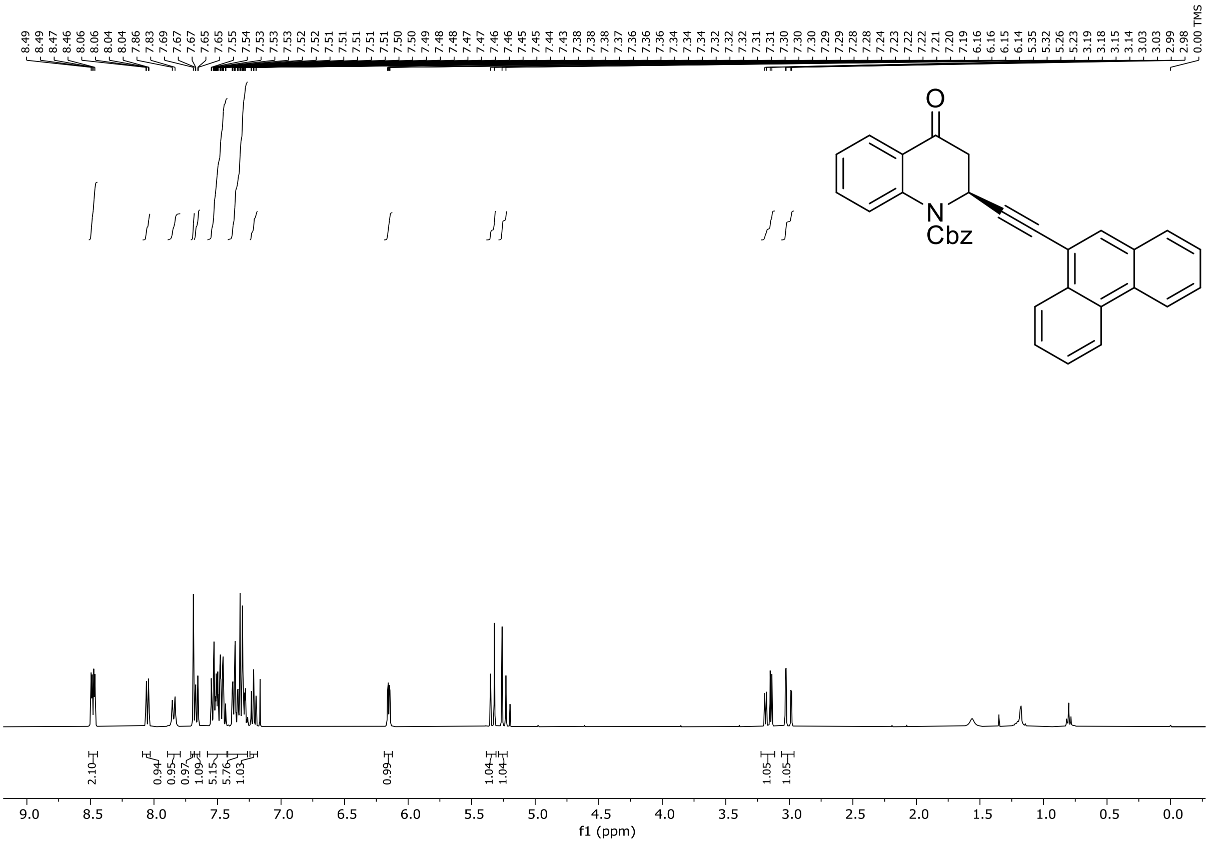

^{13}C{^{1}H} NMR (101 MHz, $CDCl_3$)

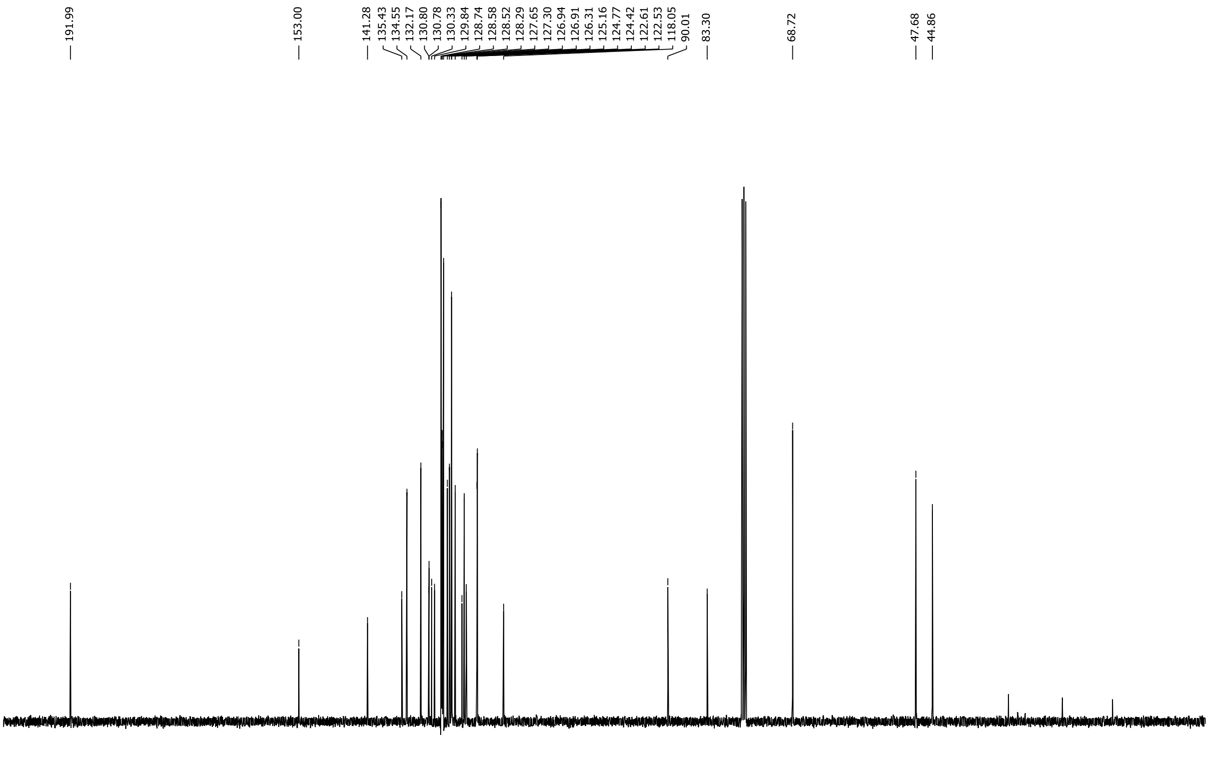

Benzyl (*S*)-4-oxo-2-(thiophen-2-ylethynyl)-3,4-dihydroquinoline-1(2H)-carboxylate (3l); ^{1}H NMR (400 MHz, $CDCl_3$)

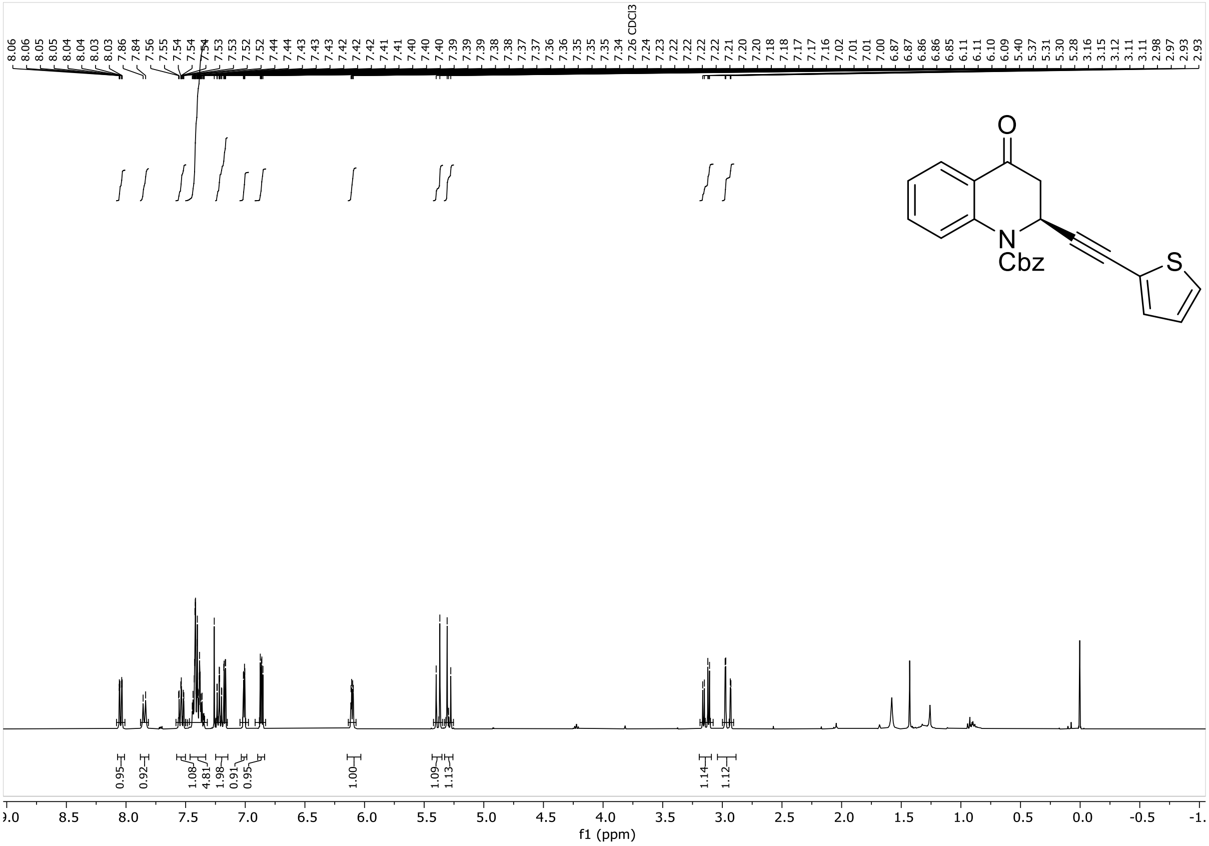

^{13}C{^{1}H} NMR (101 MHz, $CDCl_3$)

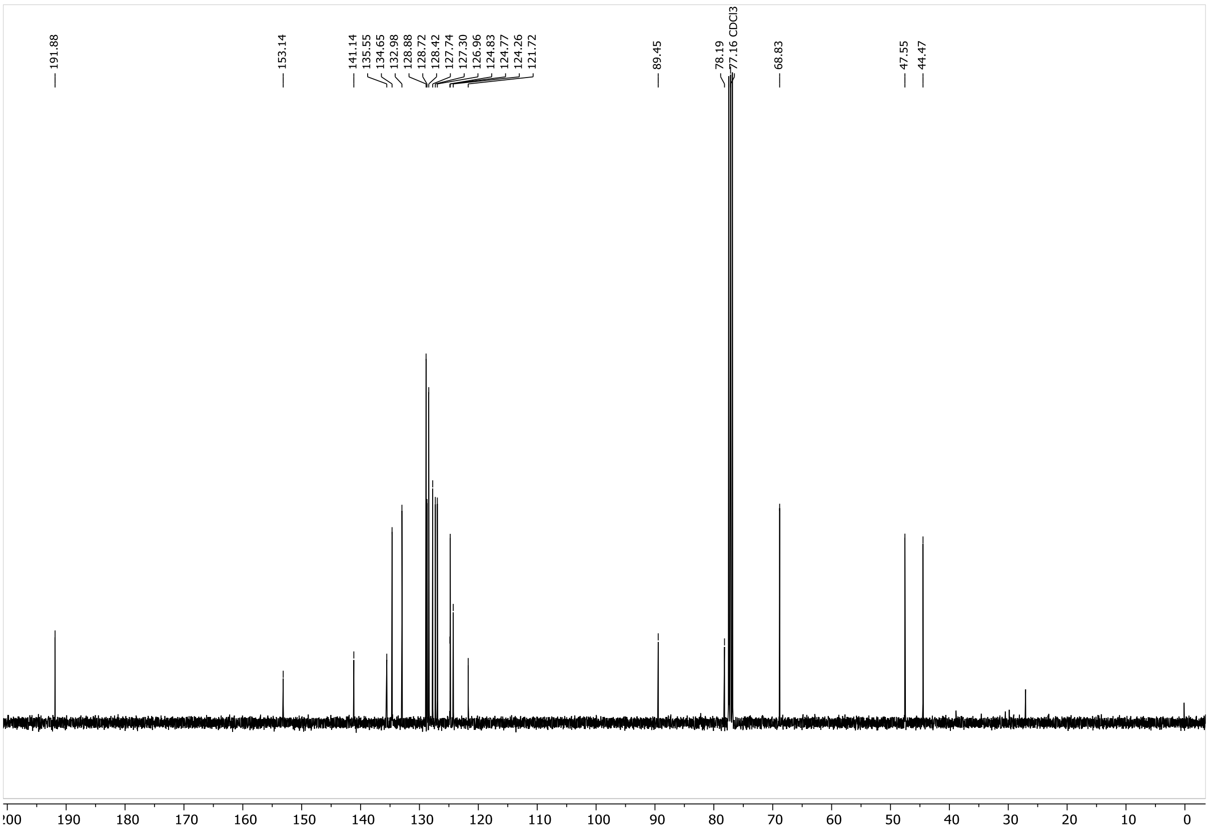

Benzyl (*S*)-4-oxo-2-((trimethylsilyl)ethynyl)-3,4-dihydroquinoline-1(2H)-carboxylate (3m); ^{1}H NMR (400 MHz, $CDCl_3$)

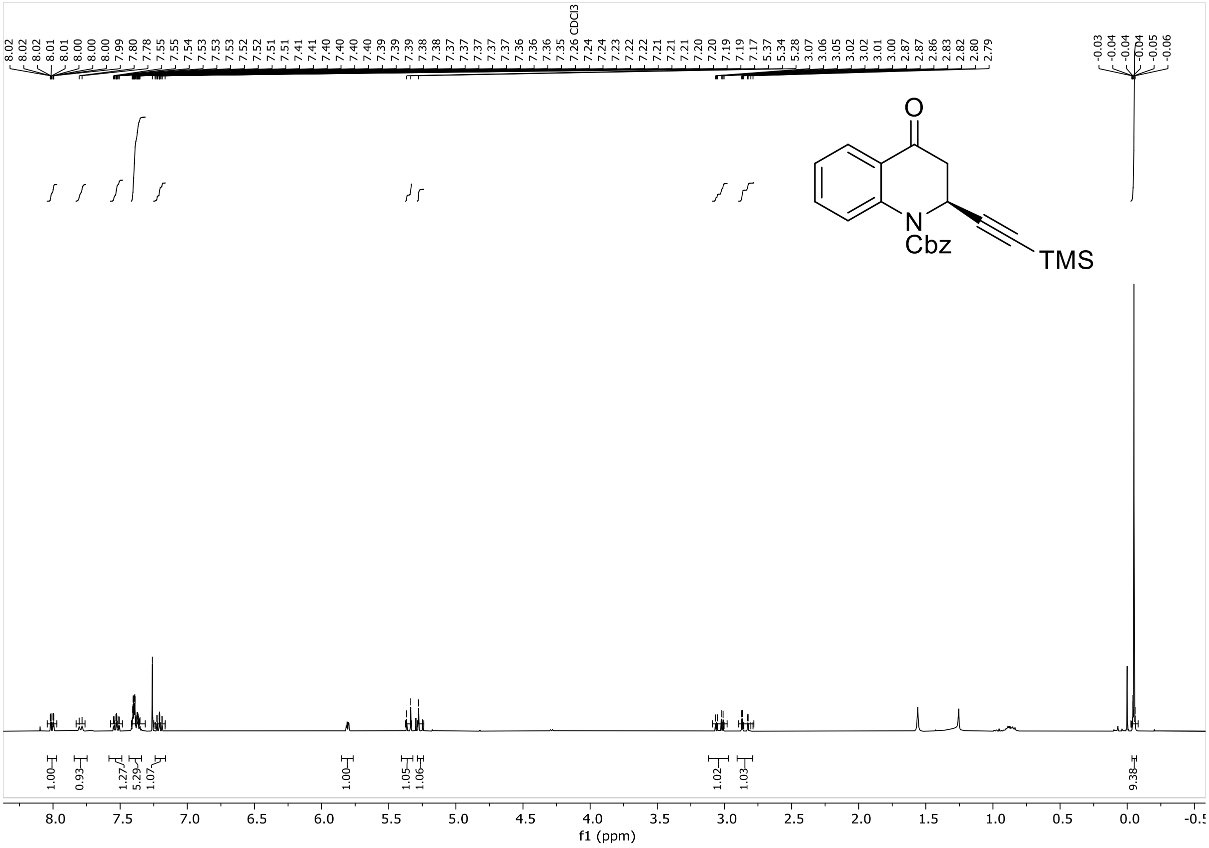

$^{13}C\{^1H\}$ NMR (101 MHz, $CDCl_3$)

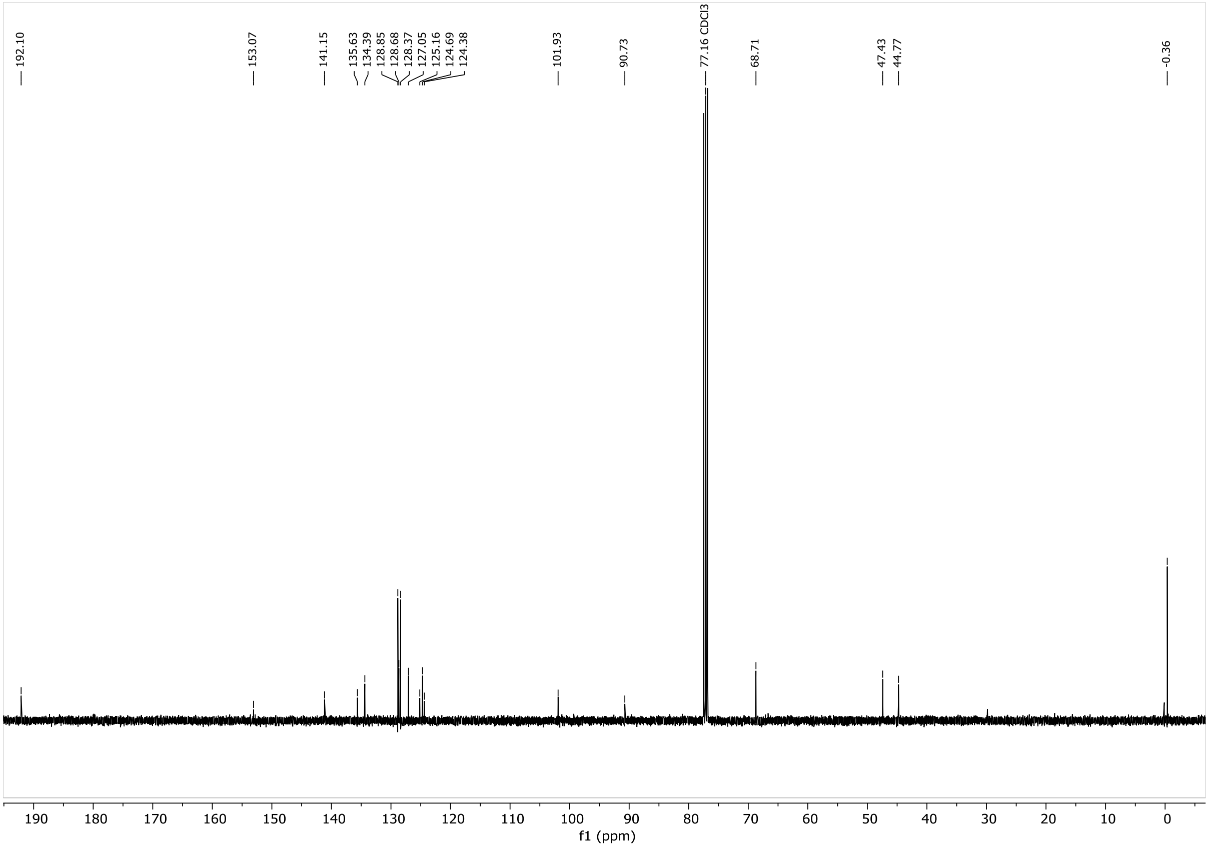

Benzyl (*S*)-2-(3-methoxy-3-oxoprop-1-yn-1-yl)-4-oxo-3,4-dihydroquinoline-1(2H)-carboxylate (3n);
^{1}**H NMR** (400 MHz, $CDCl_3$)

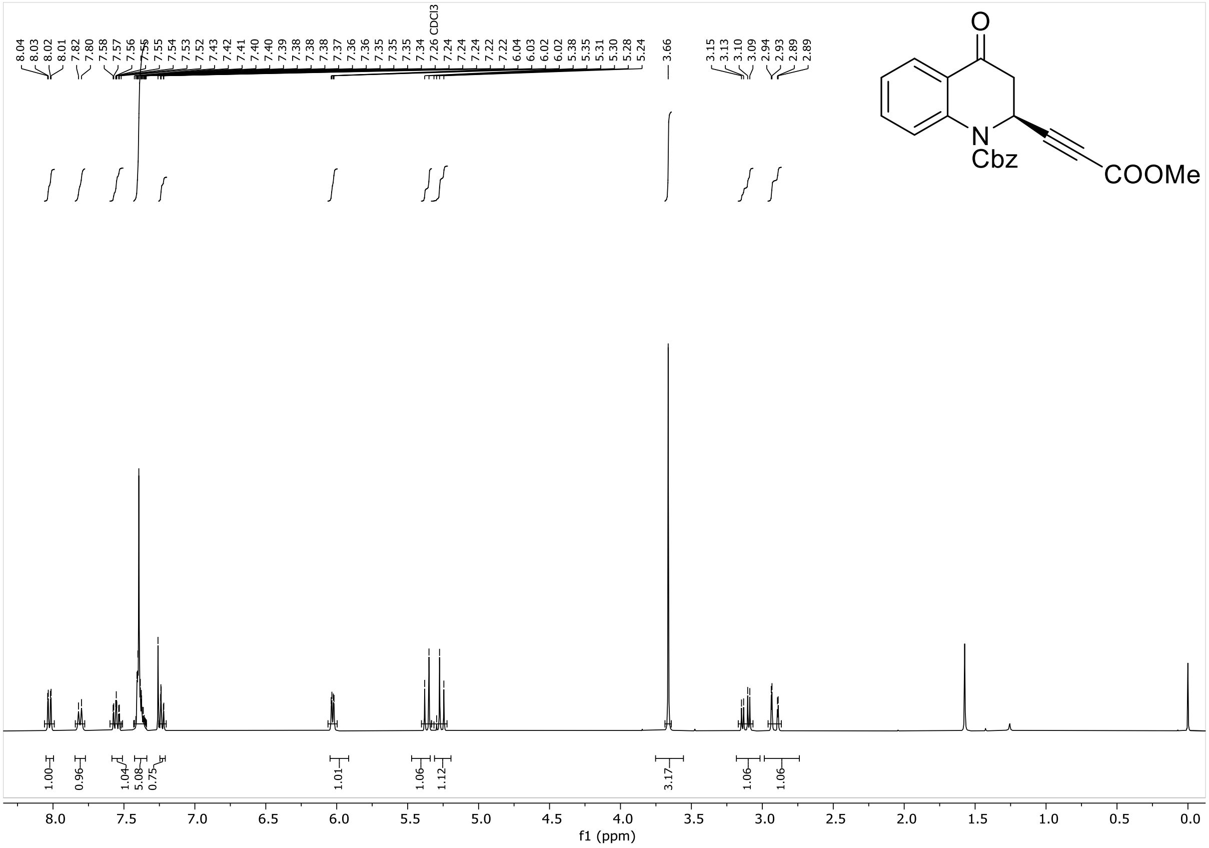

^{13}**C{**^{1}**H} NMR** (101 MHz, $CDCl_3$)

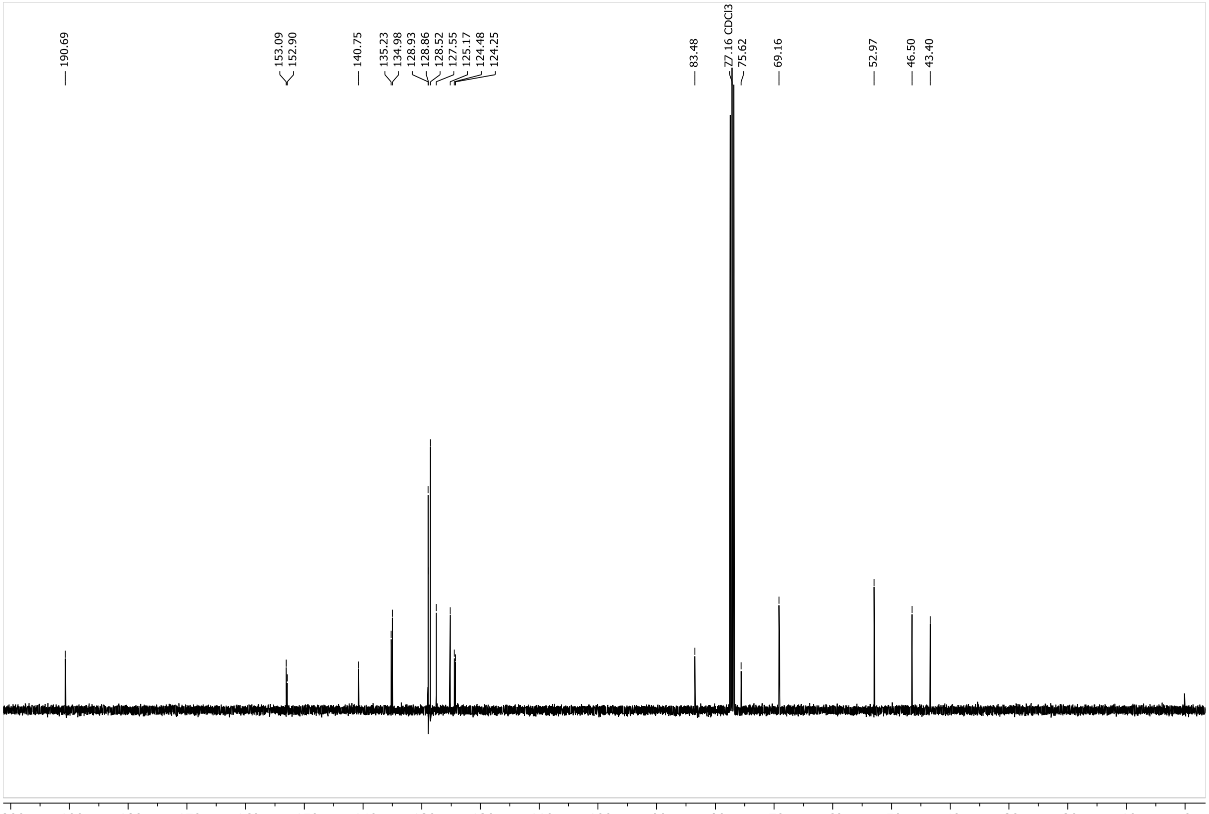

Benzyl (*S*)-2-(3-(benzyloxy)prop-1-yn-1-yl)-4-oxo-3,4-dihydroquinoline-1(2H)-carboxylate (3o); ^{1}H NMR (400 MHz, $CDCl_3$)

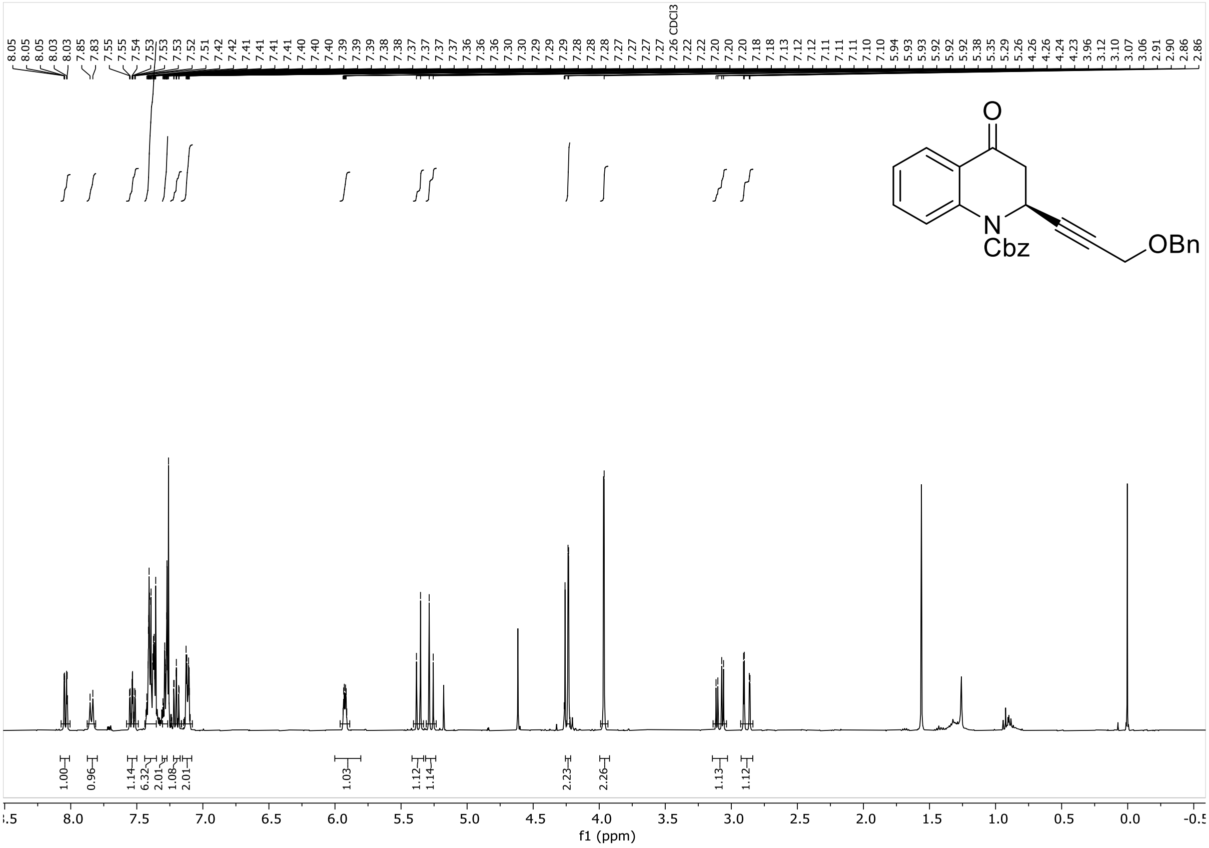

$^{13}C\{^{1}H\}$ NMR (101 MHz, $CDCl_3$)

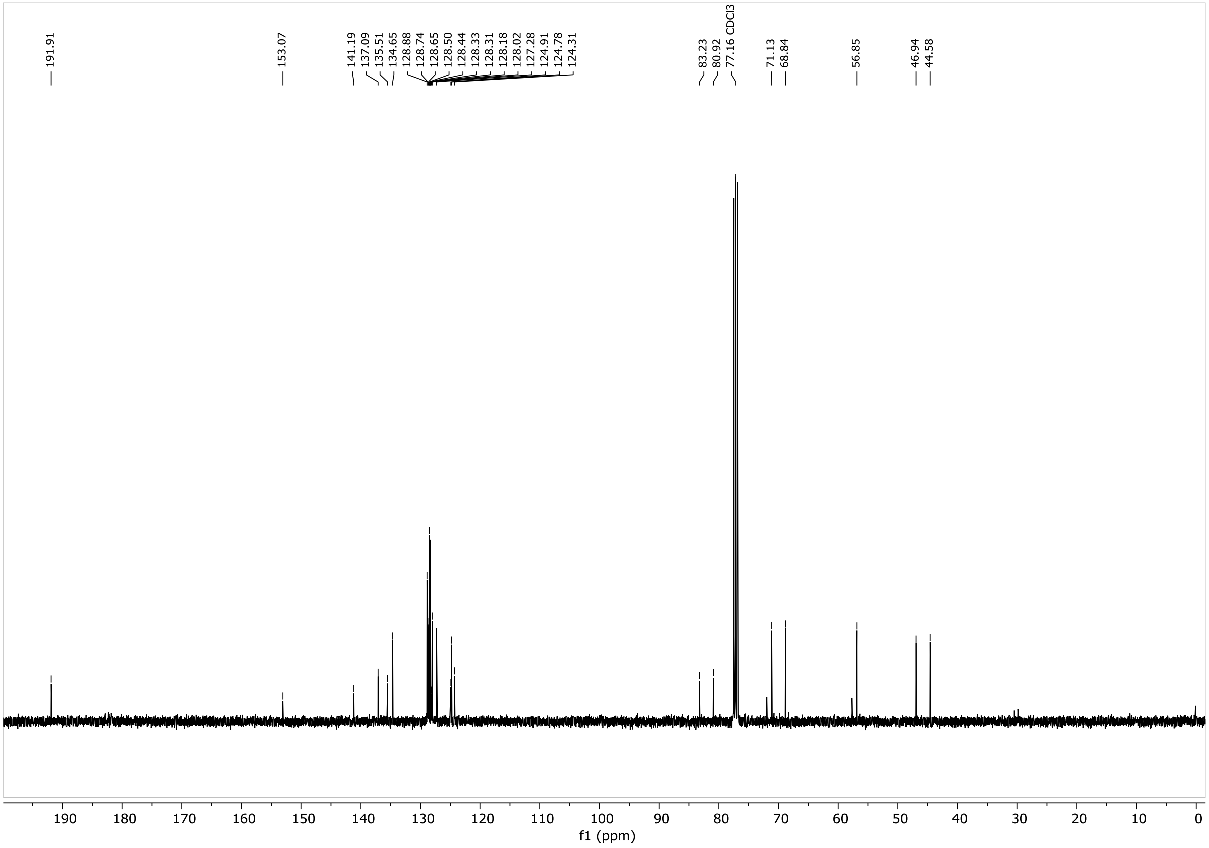

Benzyl (*S*)-4-oxo-2-(pent-1-yn-1-yl)-3,4-dihydroquinoline-1(2*H*)-carboxylate (3p); ^{1}H NMR (400 MHz, $CDCl_3$)

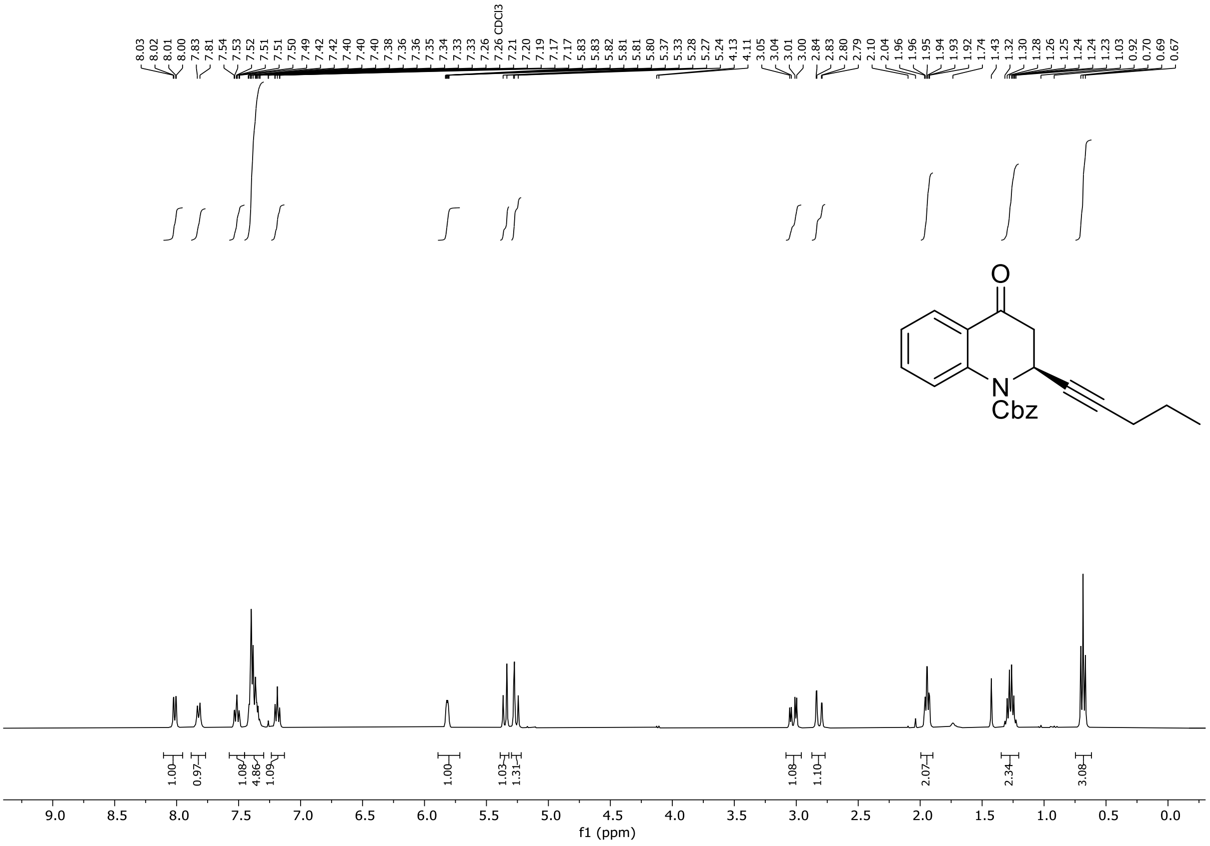

^{13}C{^{1}H} NMR (101 MHz, $CDCl_3$)

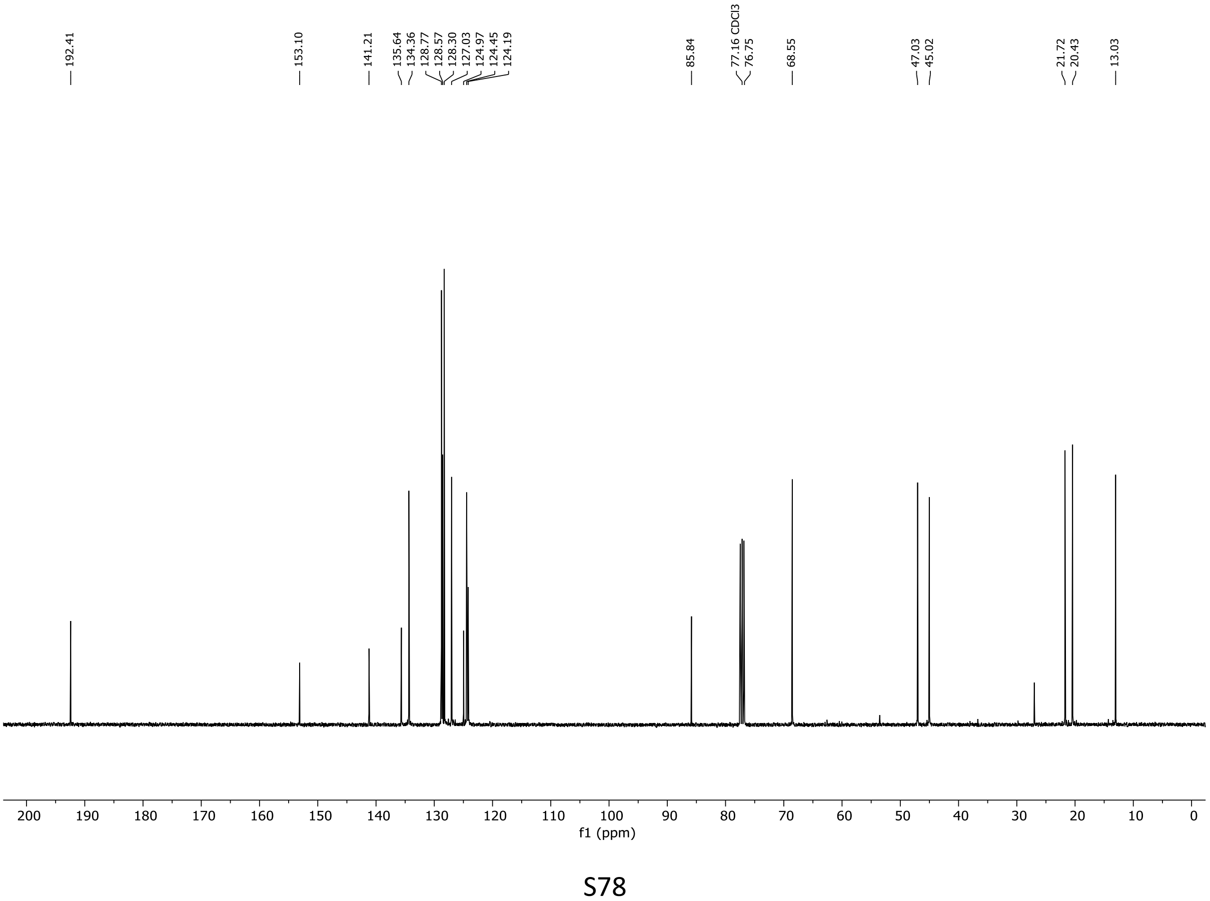

Benzyl (*S*)-6-methoxy-4-oxo-2-(*m*-tolylethynyl)-3,4-dihydroquinoline-1(2H)-carboxylate (3q); ^{1}H NMR (400 MHz, $CDCl_3$)

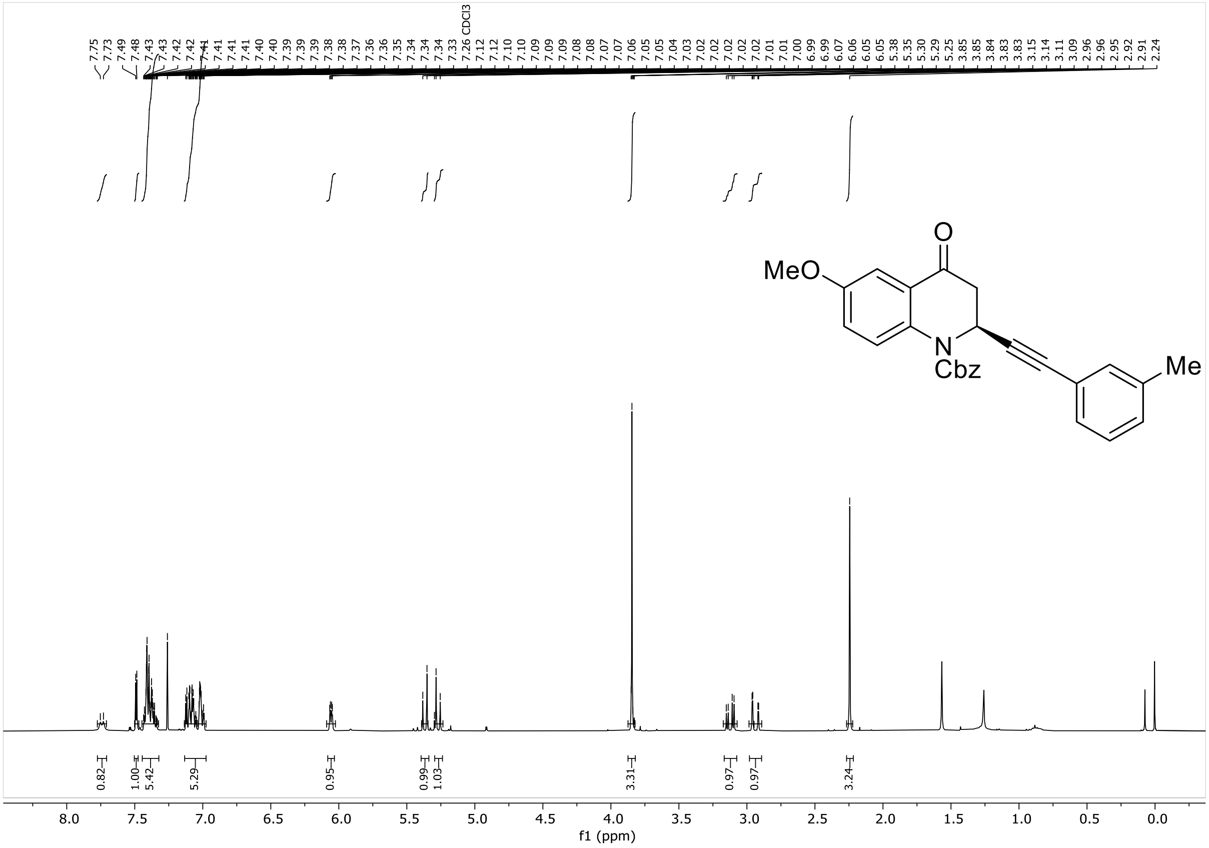

^{13}C{^{1}H} NMR (101 MHz, $CDCl_3$)

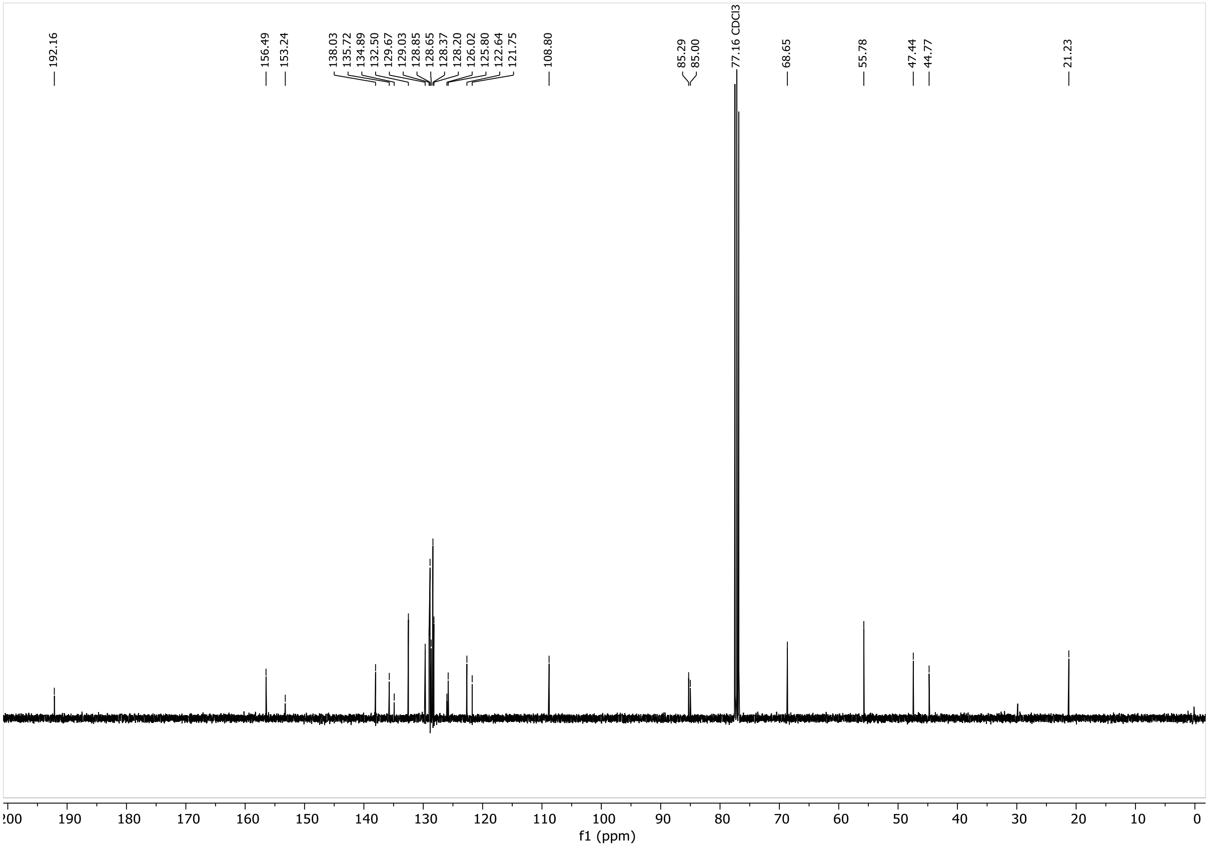

Benzyl (*S*)-6-chloro-4-oxo-2-(*m*-tolylethynyl)-3,4-dihydroquinoline-1(2H)-carboxylate (3r); ^{1}H NMR (400 MHz, $CDCl_3$)

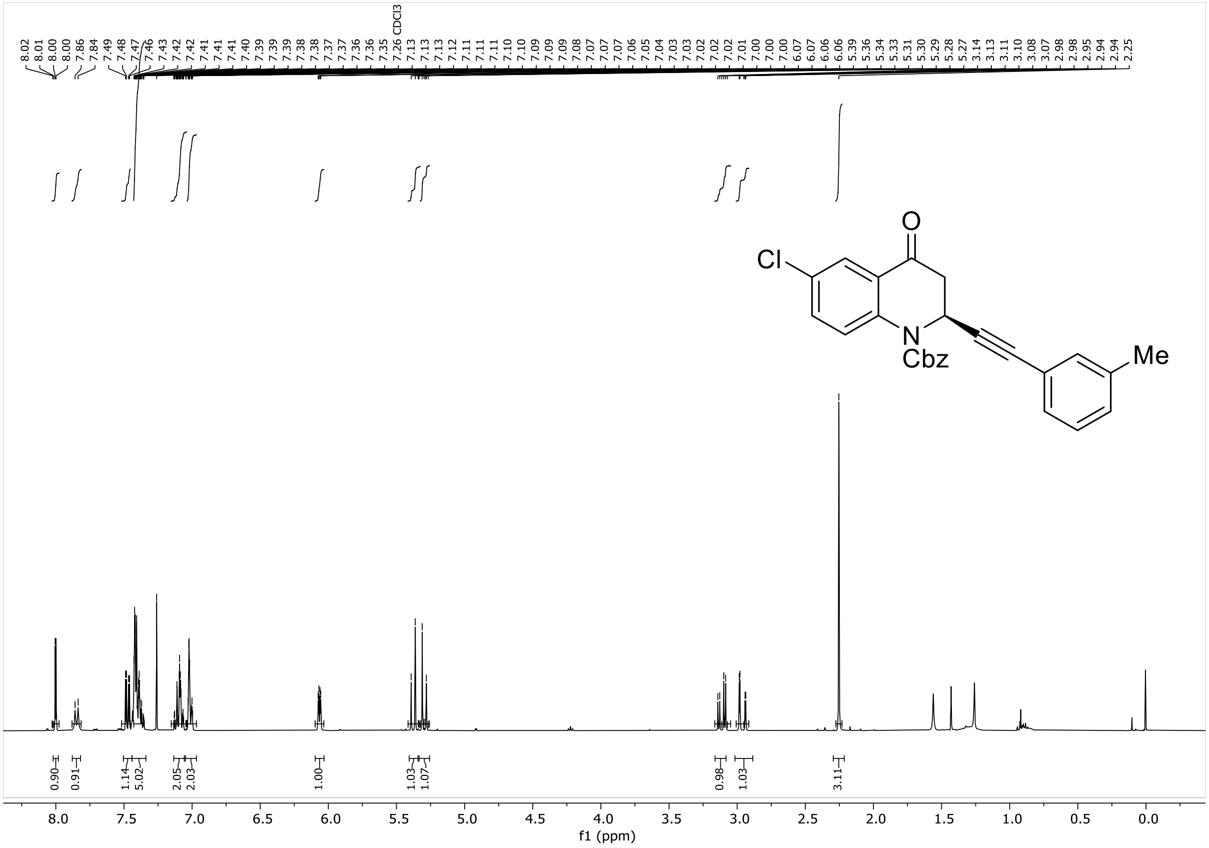

^{13}C{^{1}H} NMR (101 MHz, $CDCl_3$)

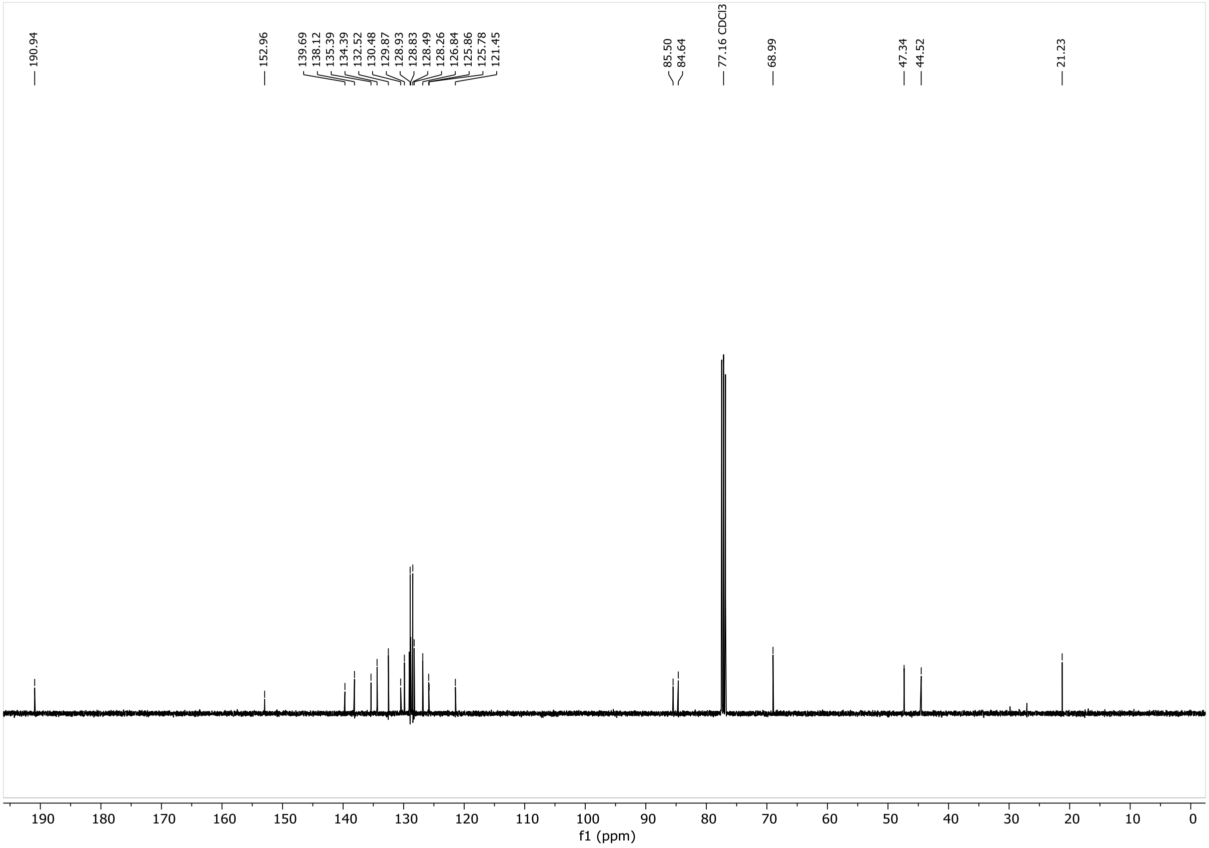

Benzyl (*S*)- 4-oxo-2-(*m*-tolylethynyl)-6-(trifluoromethyl)-3,4-dihydroquinoline-1(2H)-carboxylate (3s); ^{1}H NMR (400 MHz, $CDCl_3$)

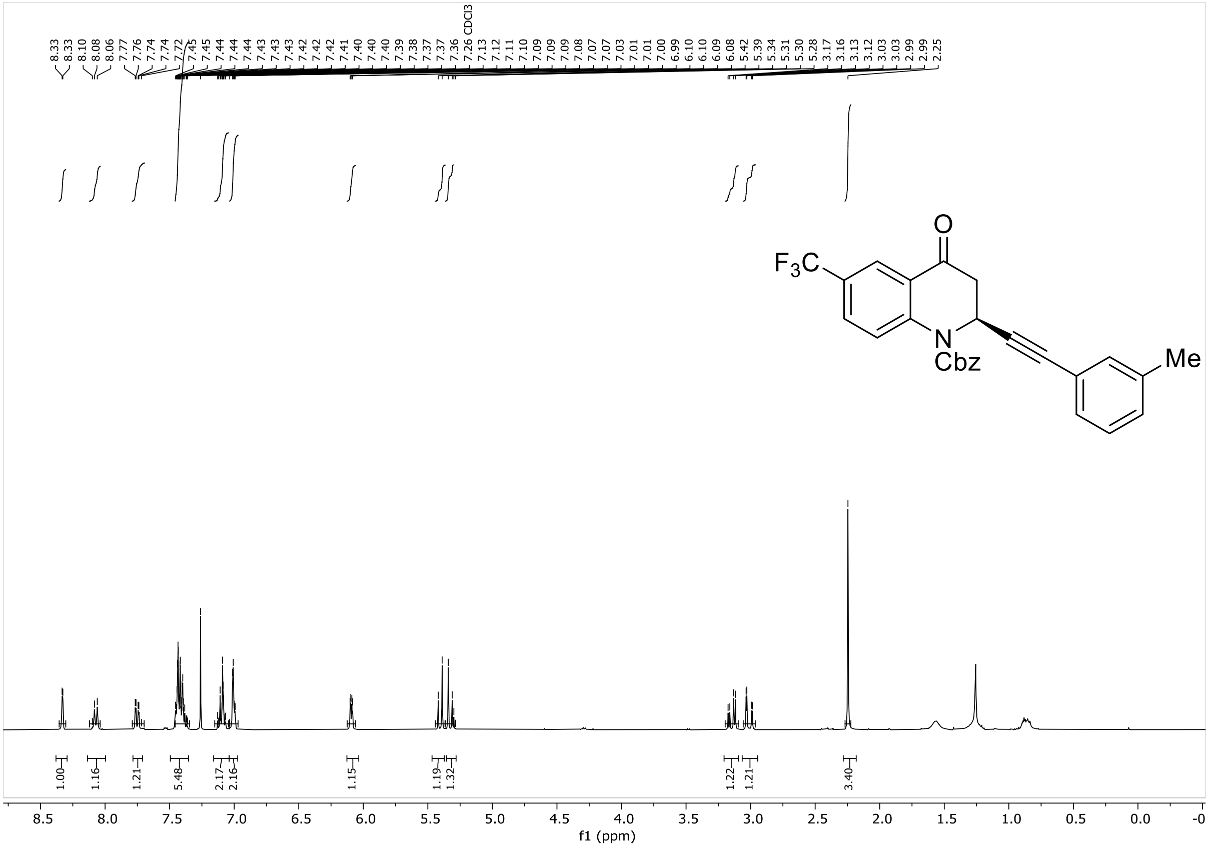

$^{13}C\{^1H\}$ NMR (101 MHz, $CDCl_3$)

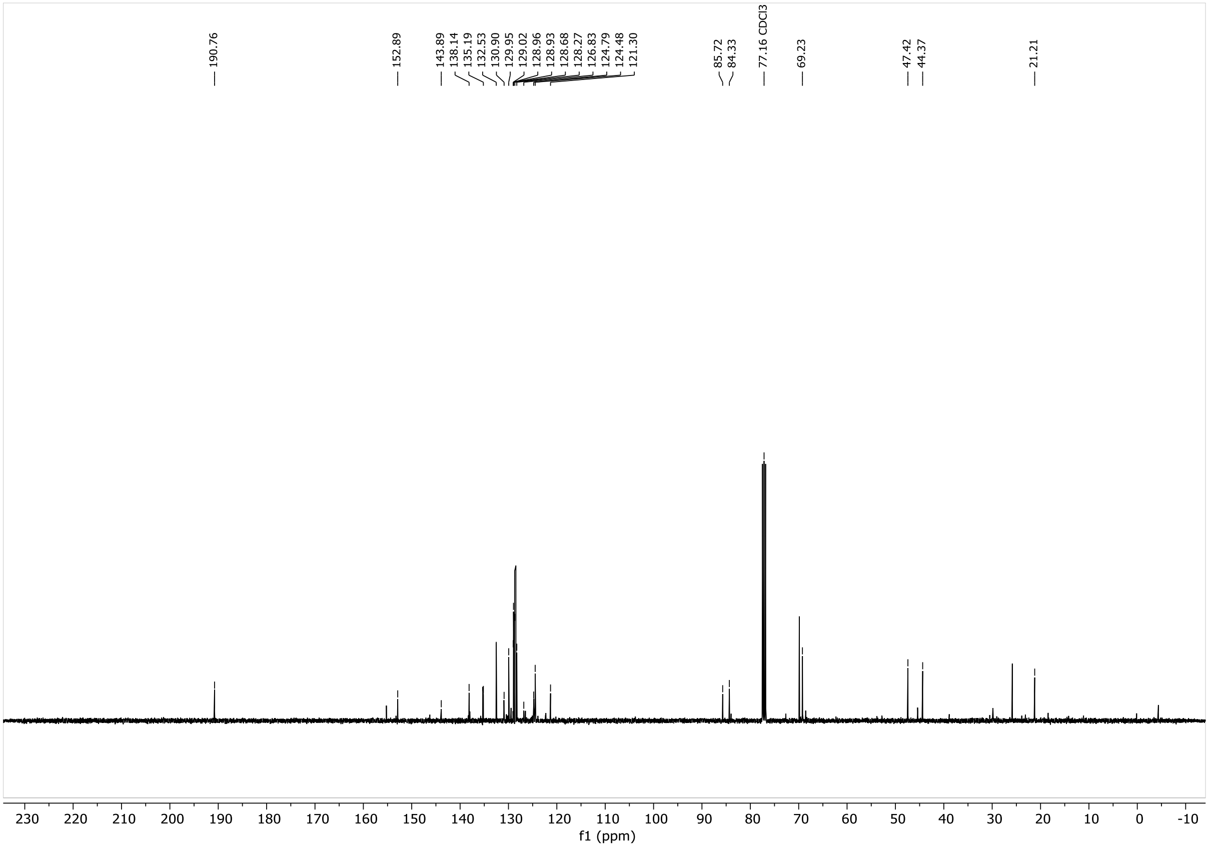

Benzyl (*S*)-6-methyl-4-oxo-2-(m-tolylethynyl)-3,4-dihydroquinoline-1(2H)-carboxylate (3t); ^{1}H NMR (400 MHz, $CDCl_3$)

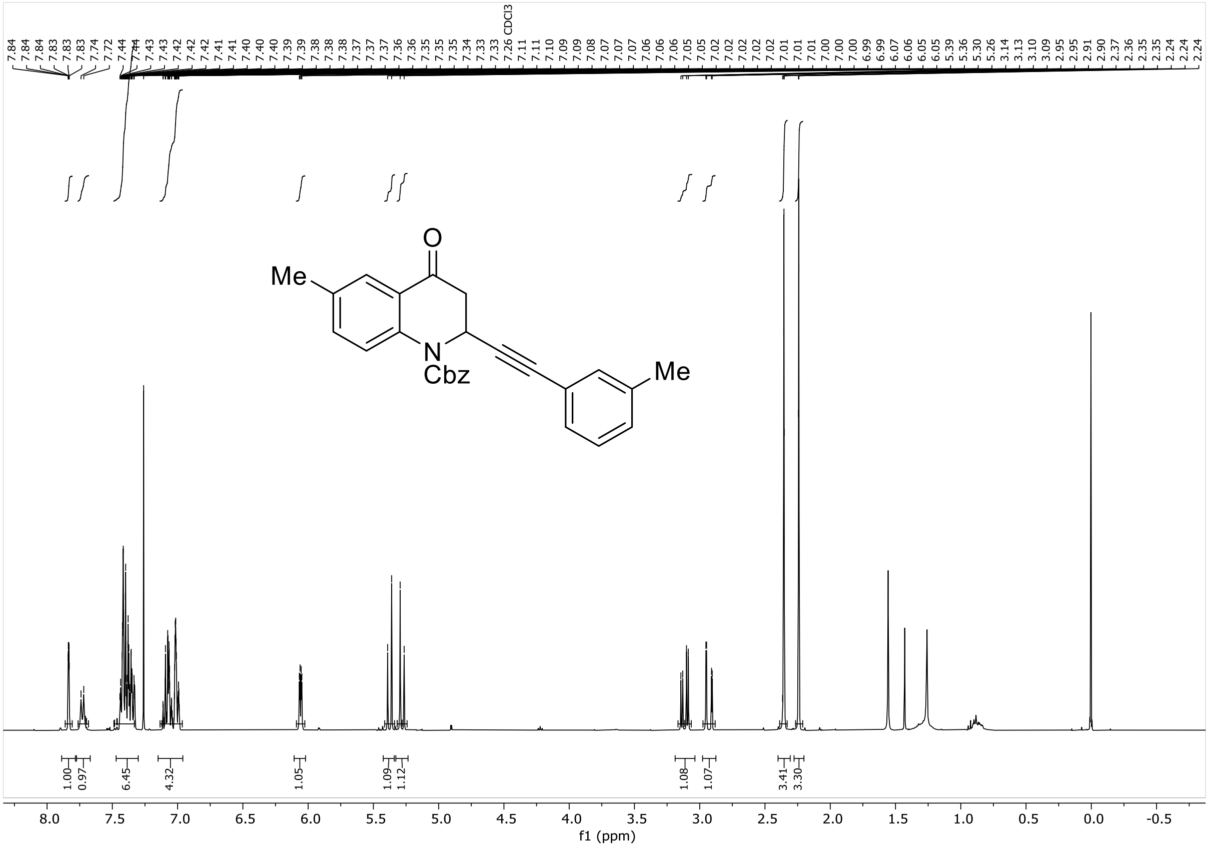

$^{13}C\{^{1}H\}$ NMR (101 MHz, $CDCl_3$)

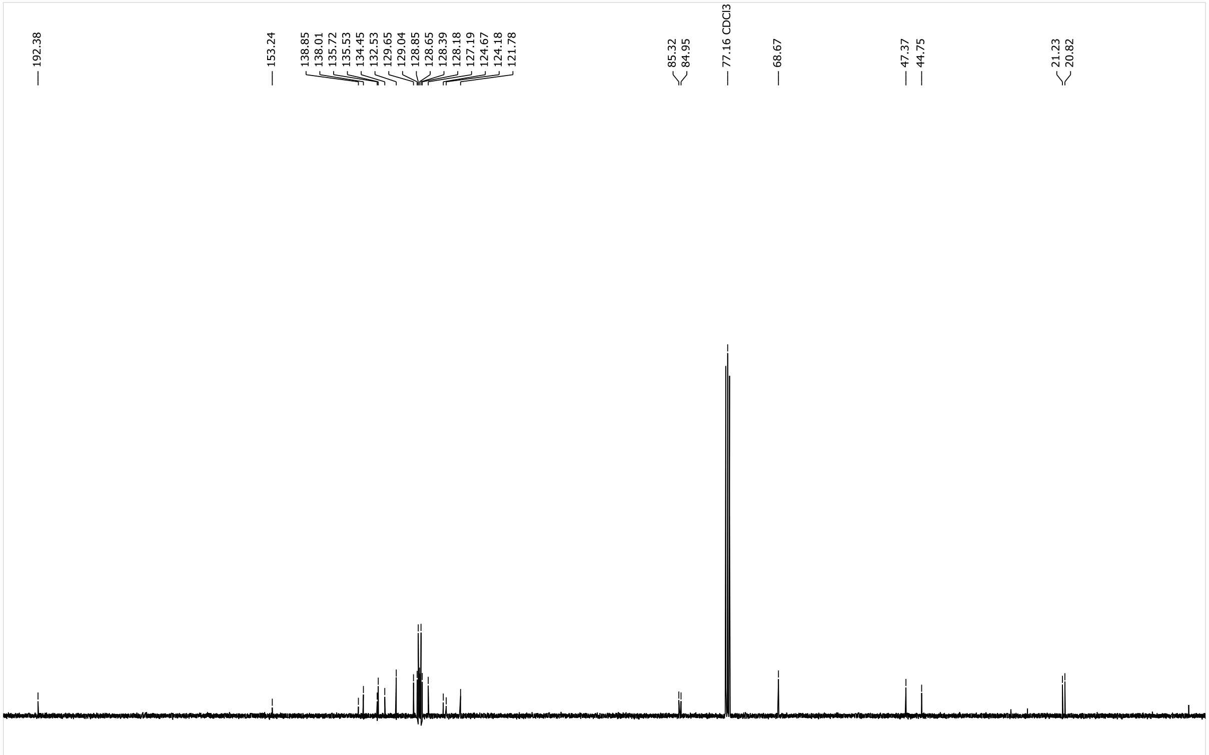

Benzyl (*S*)-6-ethyl-4-oxo-2-(*m*-tolylethynyl)-3,4-dihydroquinoline-1(2H)-carboxylate (3u); ^{1}H NMR (400 MHz, $CDCl_3$)

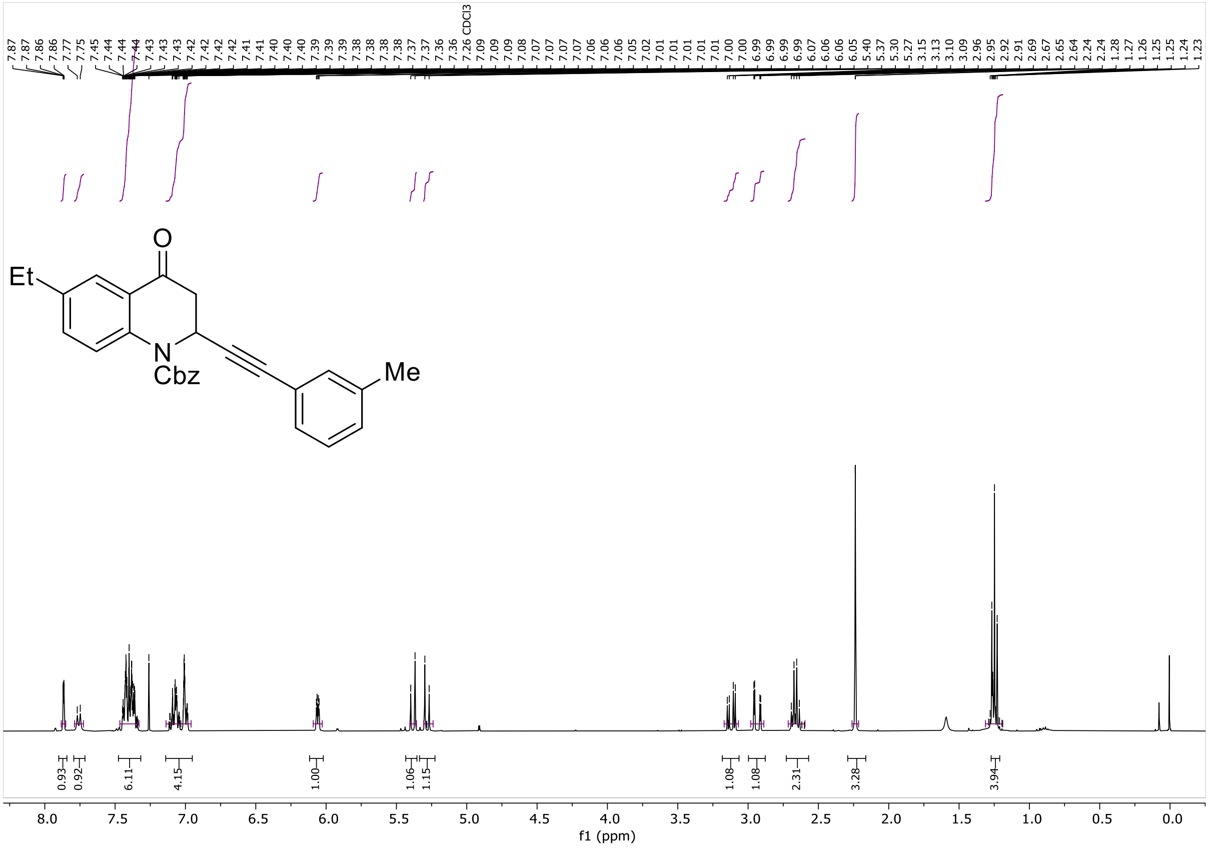

^{13}C{^{1}H} NMR (101 MHz, $CDCl_3$)

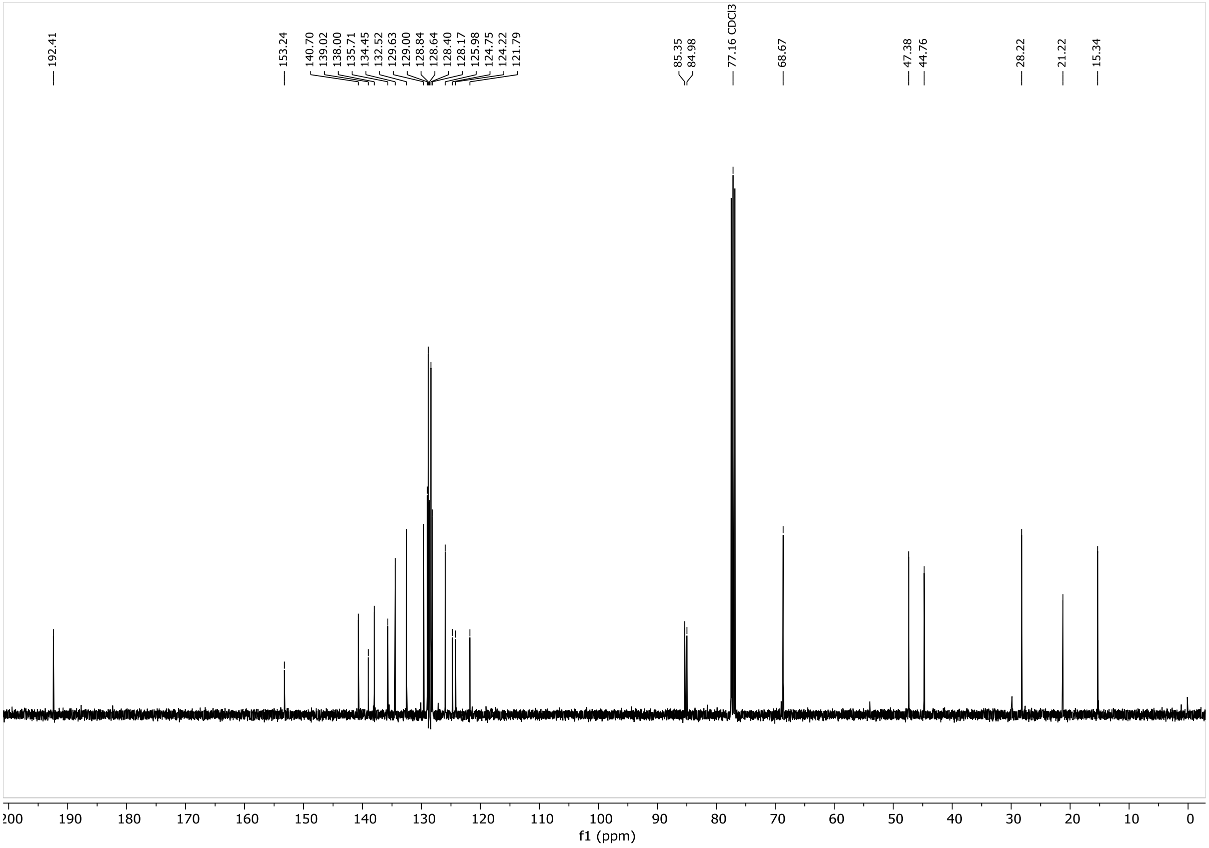

Benzyl (*S*)-5,7-dimethyl-4-oxo-2-(*m*-tolylethynyl)-3,4-dihydroquinoline-1(2H)-carboxylate (3v); ^{1}H NMR (400 MHz, CDCl$_3$)

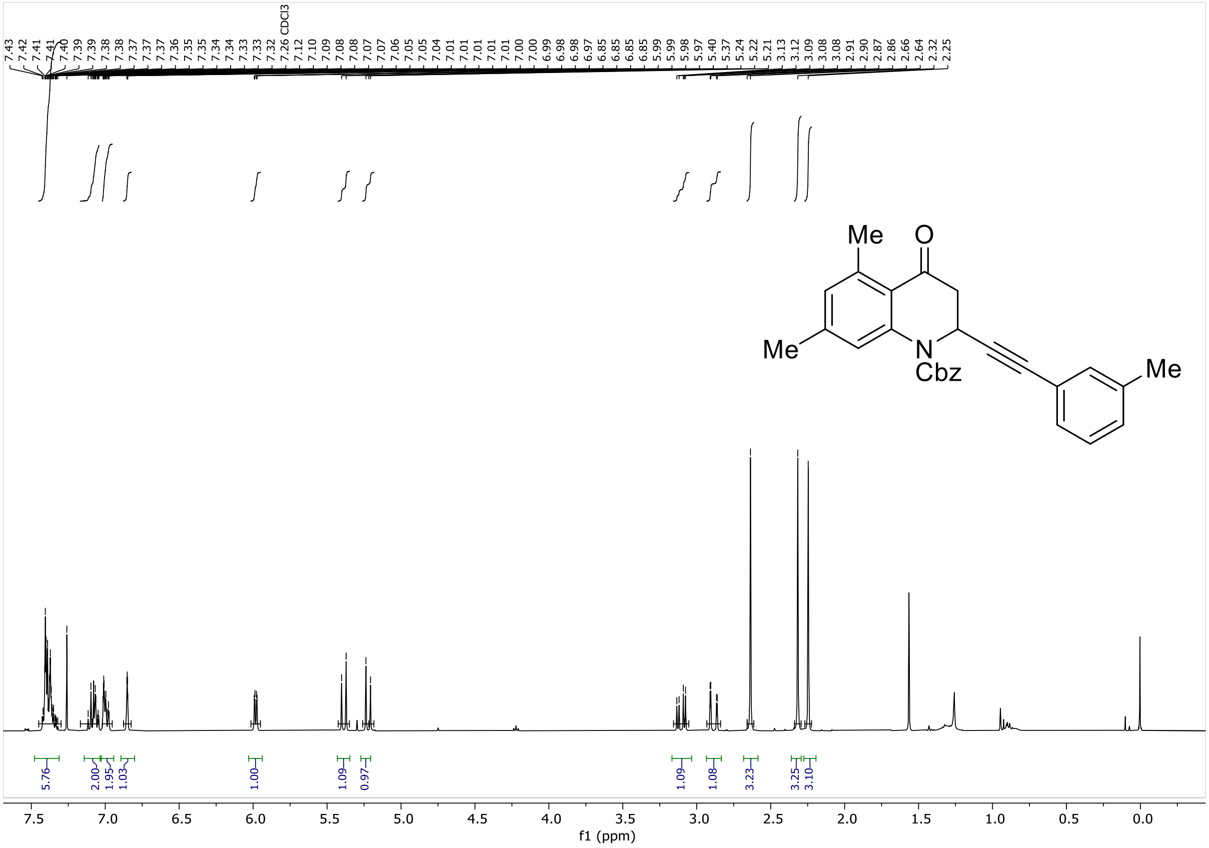

^{13}C{^{1}H} NMR (101 MHz, CDCl$_3$)

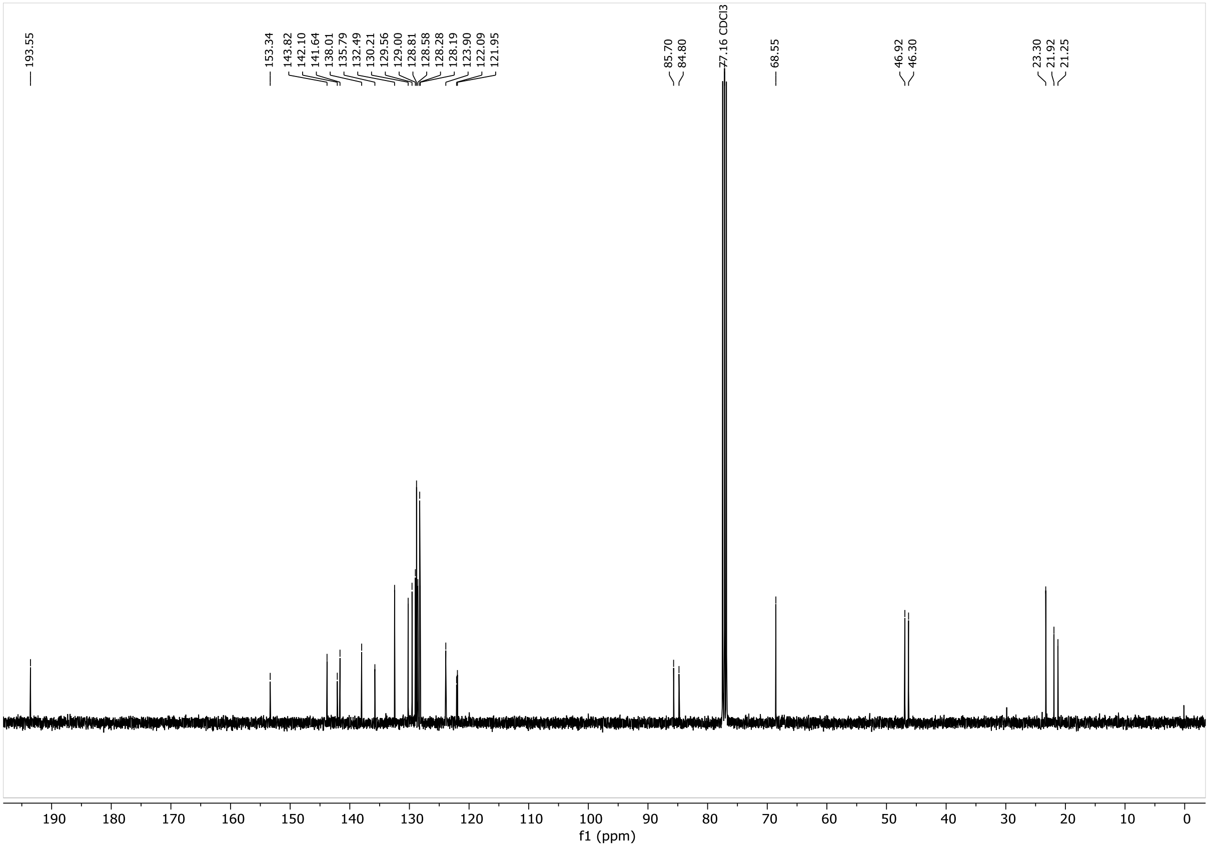

Benzyl (*S*)-8-oxo-6-(*m*-tolylethynyl)-7,8-dihydro-[1,3]dioxolo[4,5-g]quinoline-5(6H)-carboxylate (3w); ^{1}H NMR (400 MHz, CDCl$_3$)

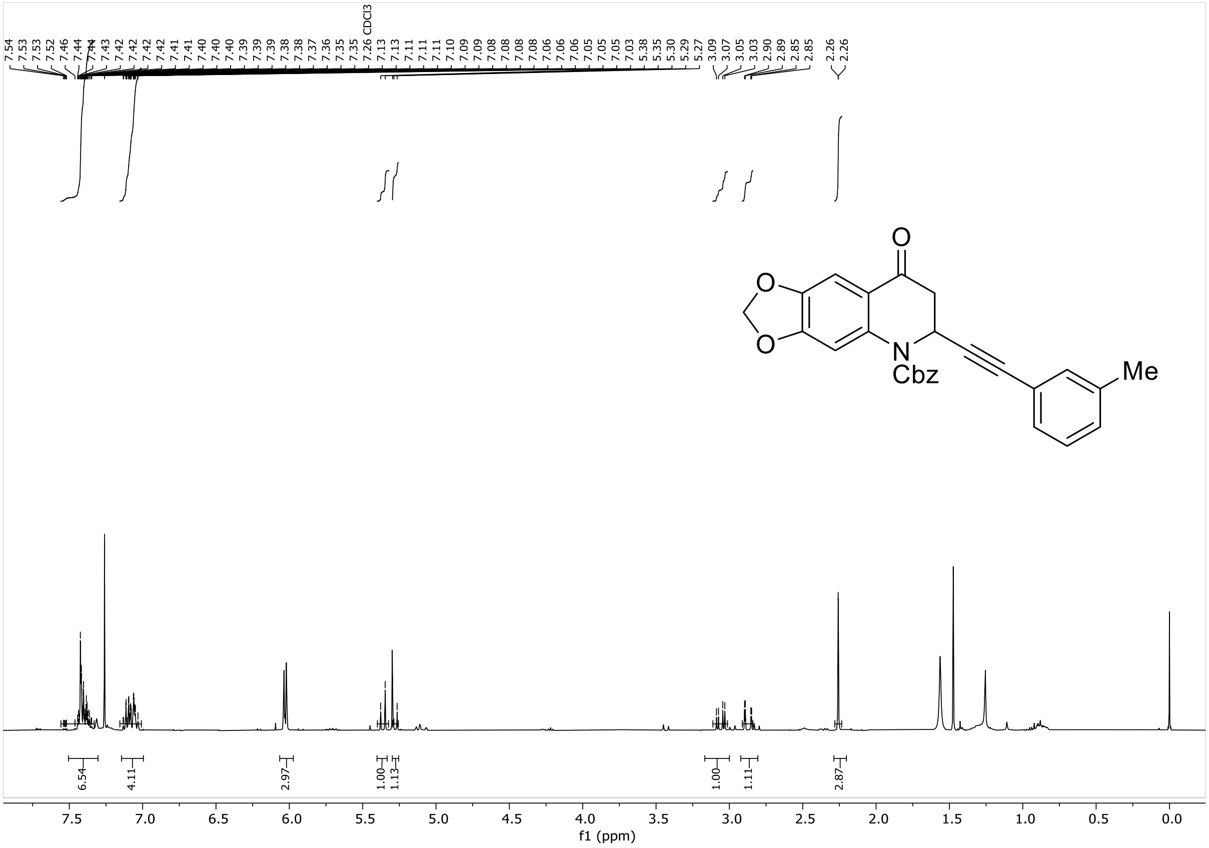

^{13}C{^{1}H} NMR (101 MHz, CDCl$_3$)

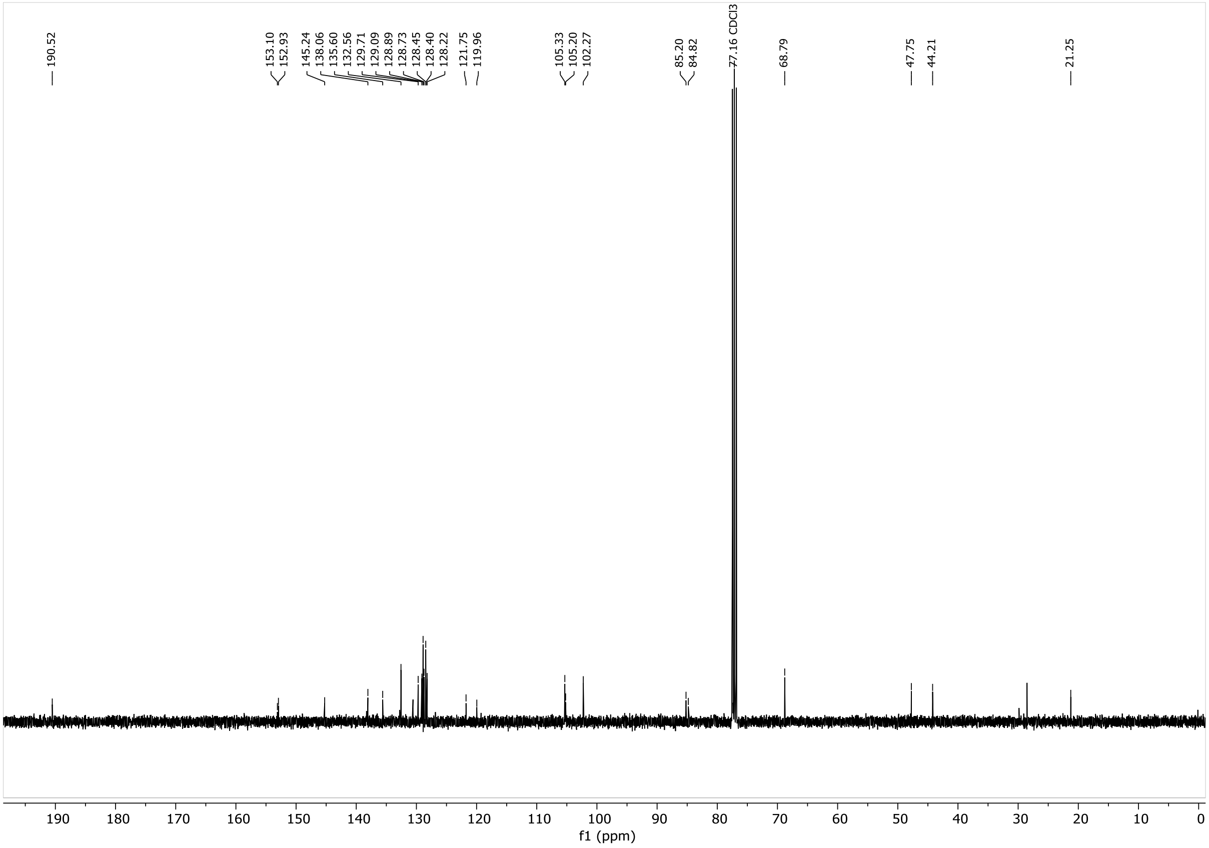

(+)-Cuspareine (4); ^{1}H NMR (400 MHz, $CDCl_3$)

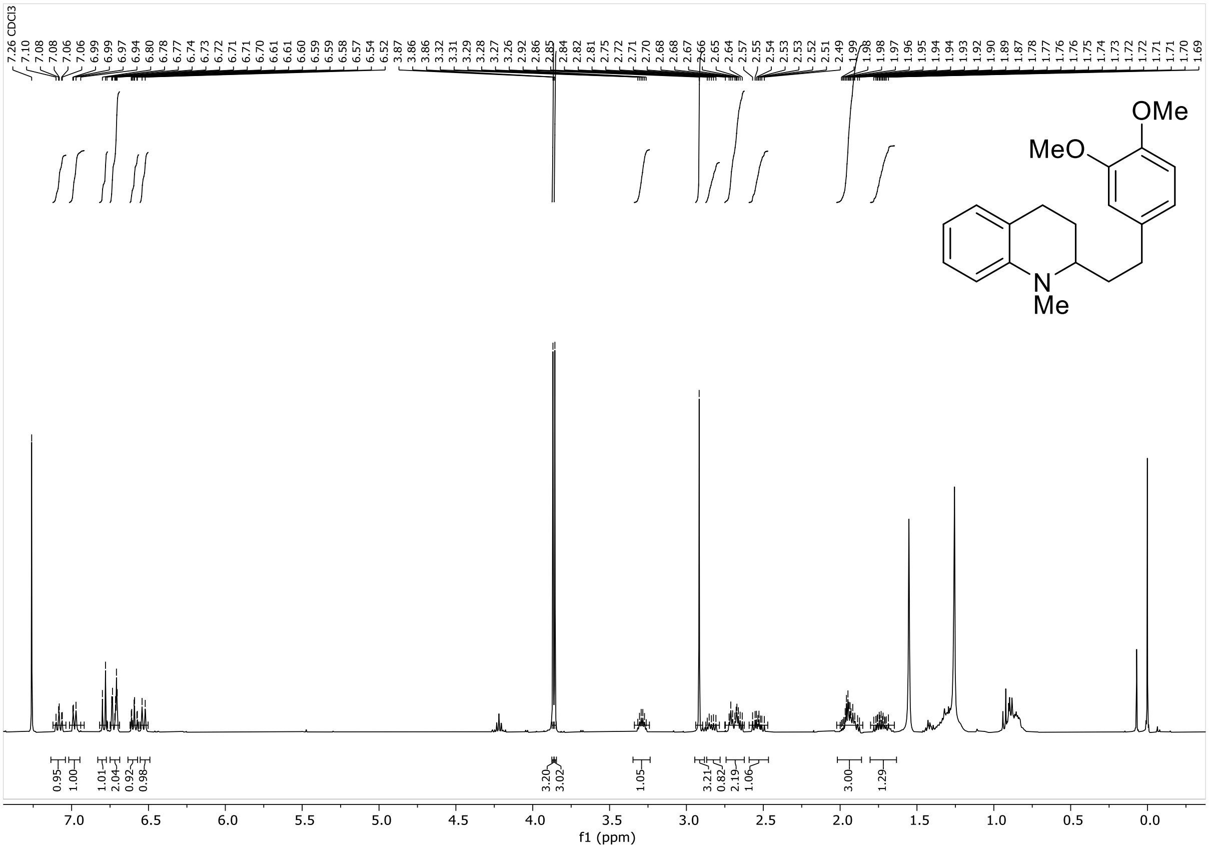

^{13}C{^{1}H} NMR (101 MHz, $CDCl_3$)

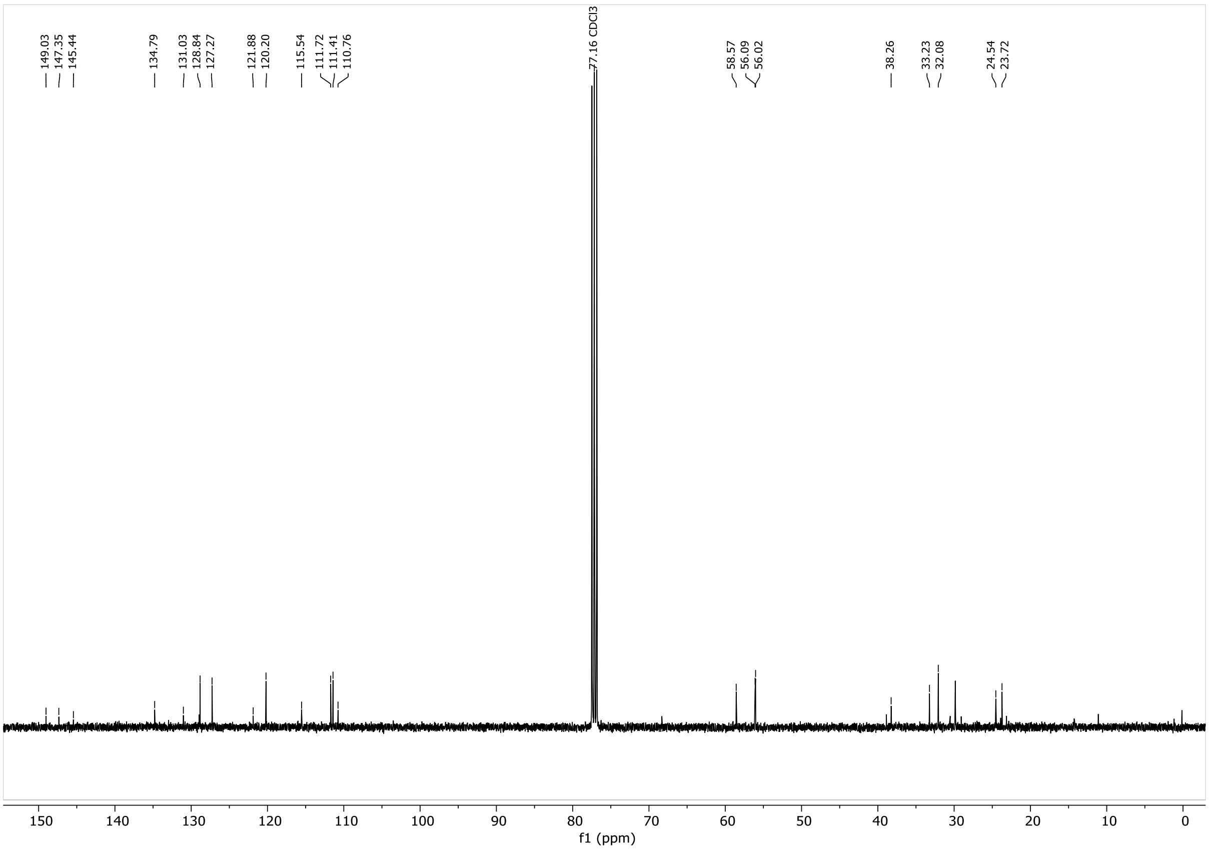

(−)-Galipinine (5); ^{1}H NMR (400 MHz, $CDCl_3$)

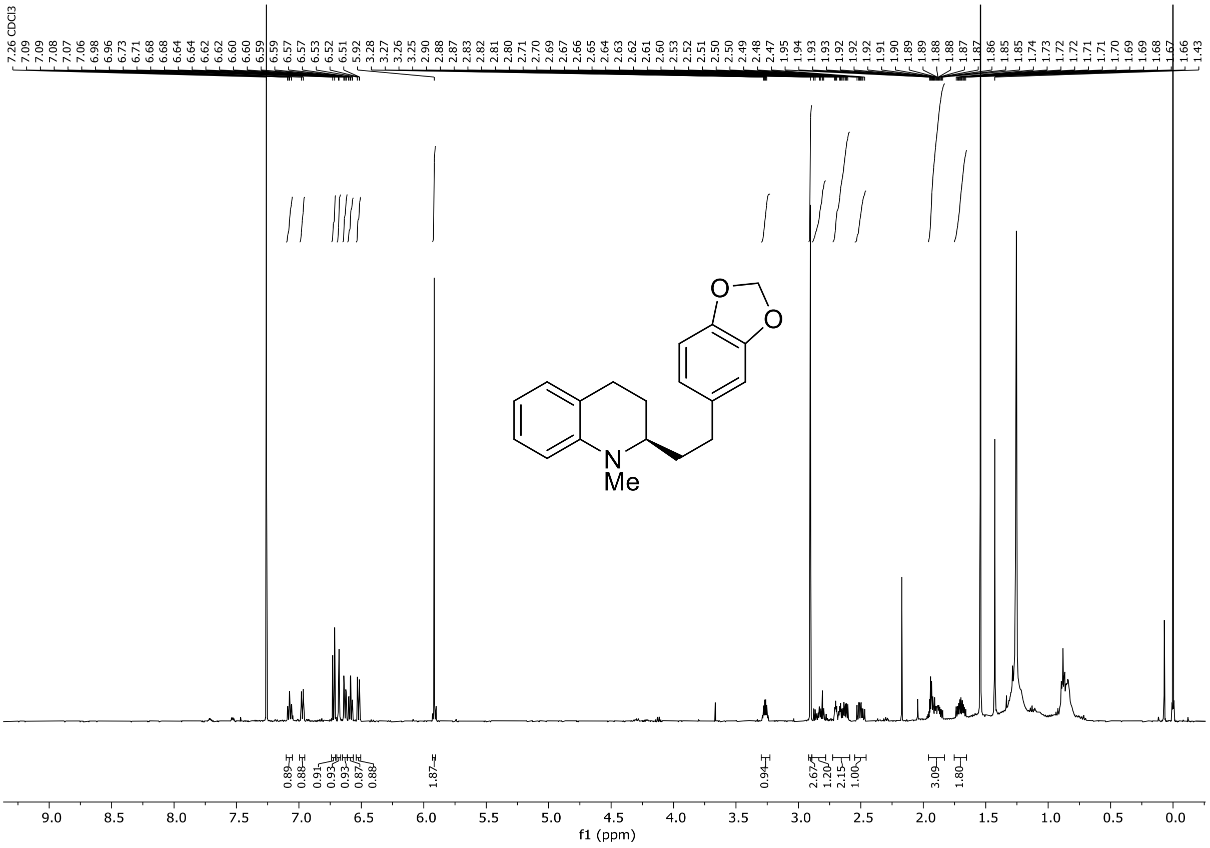

^{13}C{^{1}H} NMR (101 MHz, $CDCl_3$)

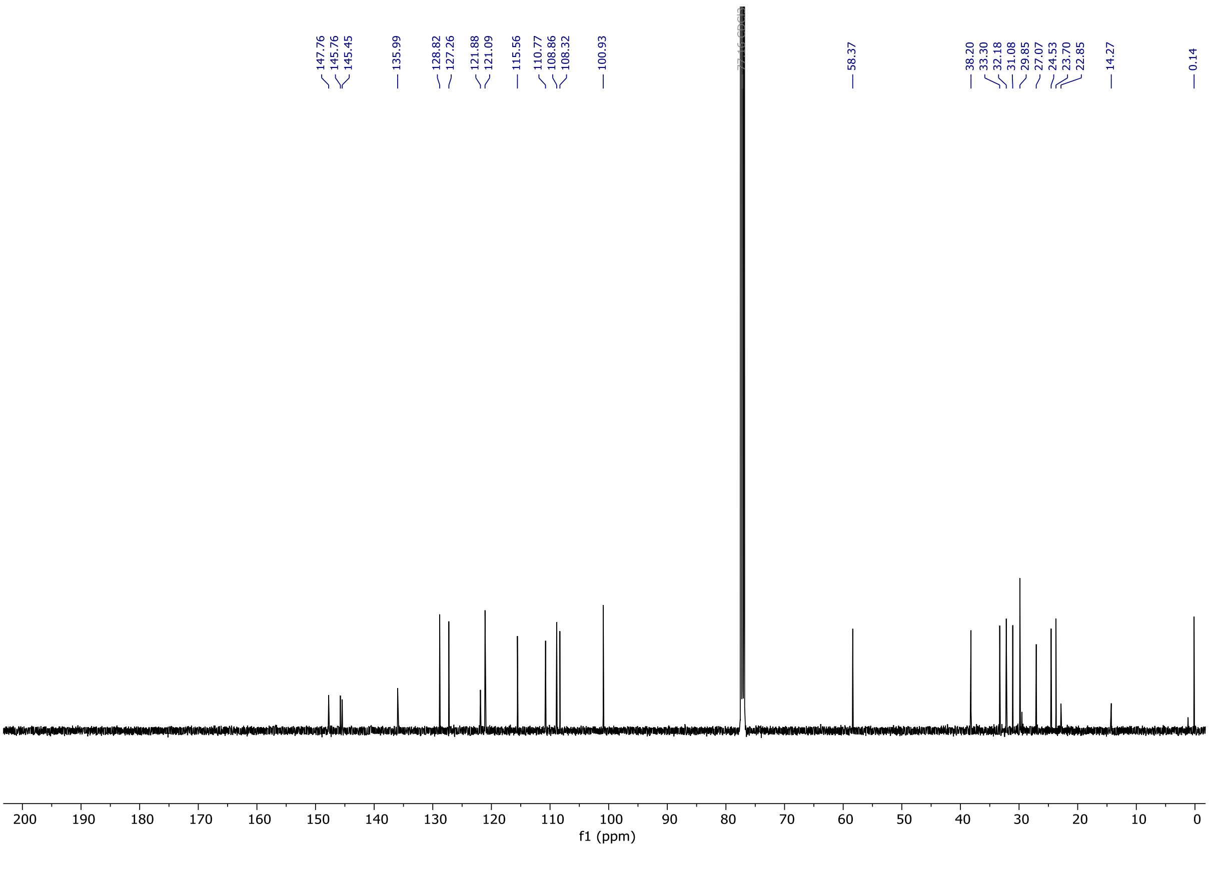

Benzyl (2S,3S)-3-methyl-4-oxo-2-(m-tolylethynyl)-3,4-dihydroquinoline-1(2H)-carboxylate (8); ^{1}H NMR (400 MHz, $CDCl_3$)

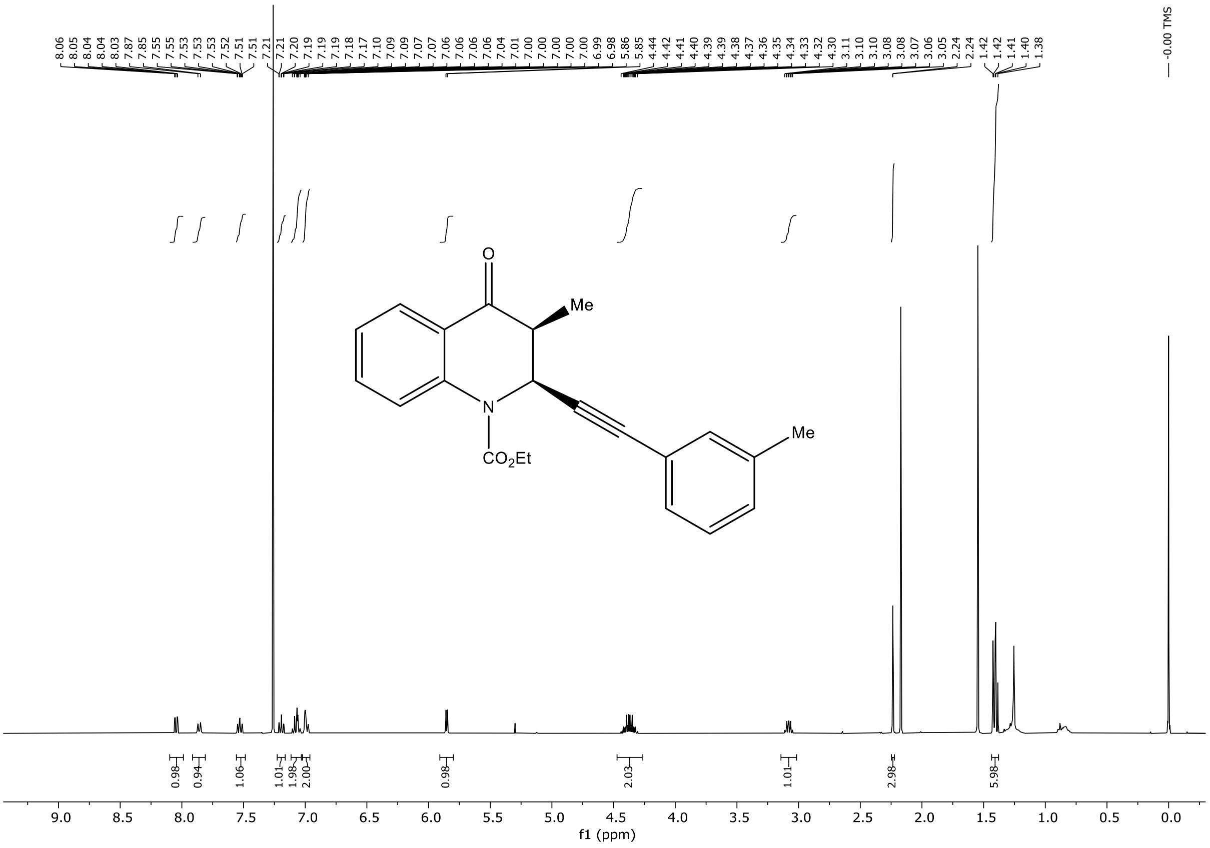

^{13}C{^{1}H} NMR (101 MHz, $CDCl_3$)

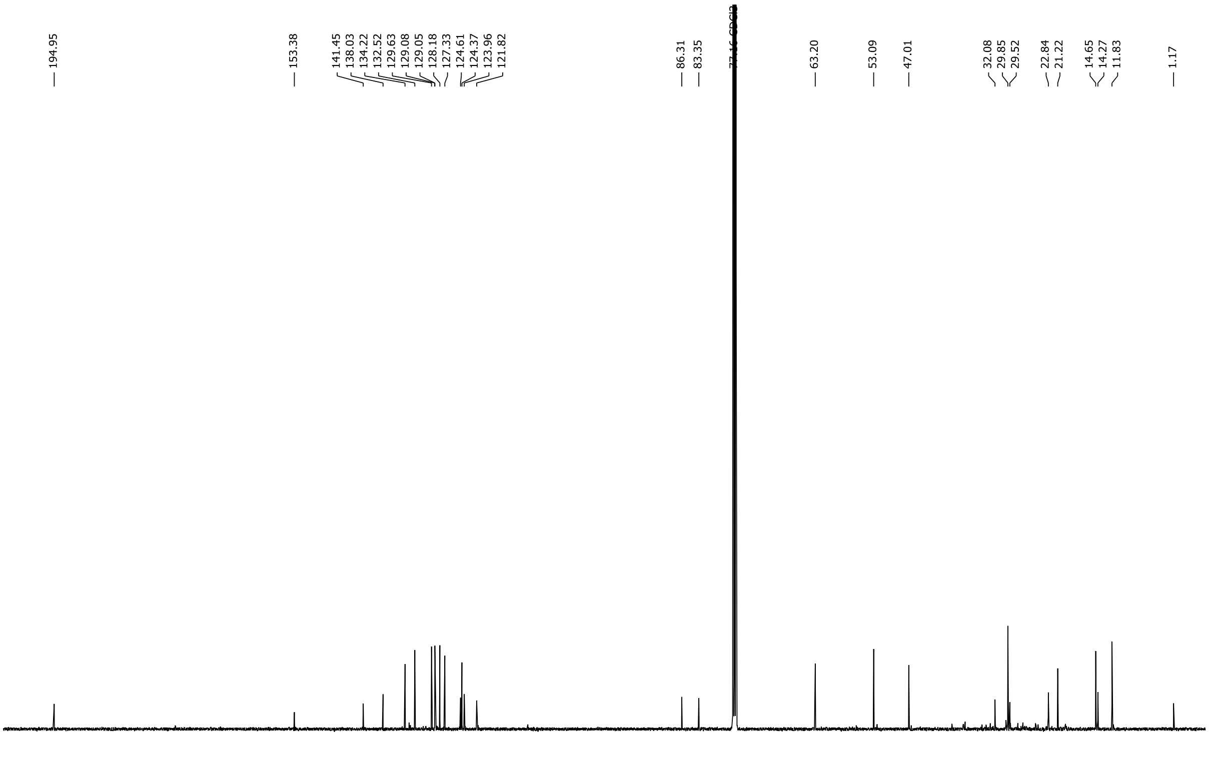

Supplement: Supplementary file 1 — jo3c01944_si_001.pdf [file jo3c01944_si_001.pdf]
